# Systematic Review to Inform a World Health Organization (WHO) Clinical Practice Guideline: Benefits and Harms of Needling Therapies for Chronic Primary Low Back Pain in Adults

**DOI:** 10.1007/s10926-023-10125-3

**Published:** 2023-11-22

**Authors:** Hainan Yu, Dan Wang, Leslie Verville, Danielle Southerst, André Bussières, Douglas P. Gross, Paulo Pereira, Silvano Mior, Andrea C. Tricco, Christine Cedraschi, Ginny Brunton, Margareta Nordin, Heather M. Shearer, Jessica J. Wong, Gaelan Connell, Danny Myrtos, Sophia da Silva-Oolup, James J. Young, Martha Funabashi, Andrew Romanelli, Joyce G. B. Lee, Kent Stuber, Brett Guist, Javier Muñoz Laguna, Léonie Hofstetter, Kent Murnaghan, Cesar A. Hincapié, Carol Cancelliere

**Affiliations:** 1 Institute for Disability and Rehabilitation Research and Faculty of Health Sciences, Ontario Tech University, Oshawa, Canada; 2 Département Chiropratique, Université du Québec à Trois-Rivières, Trois-Rivières, QC Canada; 3 School of Physical and Occupational Therapy, Faculty of Medicine and Health Sciences, McGill University, Montreal, Canada; 4 Department of Physical Therapy, University of Alberta, Edmonton, Canada; 5 Department of Neurosurgery, Centro Hospitalar Universitário São João, Faculty of Medicine, University of Porto, Porto, Portugal; 6 Department of Research and Innovation, Canadian Memorial Chiropractic College, Toronto, Canada; 7 Li Ka Shing Knowledge Institute, St. Michael’s Hospital, Unity Health Toronto, Toronto, Canada; 8 Epidemiology Division and Institute for Health Policy, Management, and Evaluation, Dalla Lana School of Public Health, University of Toronto, Toronto, Canada; 9 Queen’s Collaboration for Health Care Quality Joanna Briggs Institute Centre of Excellence, Queen’s University, Kingston, Canada; 10 Division of General Medical Rehabilitation, Geneva University and University Hospitals, Geneva, Switzerland; 11 Division of Clinical Pharmacology and Toxicology, Multidisciplinary Pain Centre, Geneva University Hospitals, Geneva, Switzerland; 12 EPPI-Centre, UCL Institute of Education, University College London, London, England, UK; 13 Department of Health Research Methods, Evidence and Impact, Faculty of Health Sciences, McMaster University, Hamilton, Canada; 14 Departments of Orthopedic Surgery and Environmental Medicine, NYU Grossman School of Medicine, New York University, New York, USA; 15 Bloorview Research Institute, Holland Bloorview Kids Rehabilitation Hospital, Toronto, Canada; 16 Department of Clinical Education, Canadian Memorial Chiropractic College, Toronto, Canada; 17 Department of Undergraduate Education, Canadian Memorial Chiropractic College, Toronto, Canada; 18 Department of Graduate Education, Canadian Memorial Chiropractic College, Toronto, Canada; 19 Schroeder Arthritis Institute, Krembil Research Institute, University Health Network, Toronto, Canada; 20 Center for Muscle and Joint Health, Department of Sports Science and Clinical Biomechanics, University of Southern Denmark, Odense, Denmark; 21 Parker University Research Center, Dallas, USA; 22 EBPI-UWZH Musculoskeletal Epidemiology Research Group, University of Zurich and Balgrist University Hospital, Zurich, Switzerland; 23 Epidemiology, Biostatistics and Prevention Institute (EBPI), University of Zurich, Zurich, Switzerland; 24 University Spine Centre Zurich (UWZH), Balgrist University Hospital and University of Zurich, Zurich, Switzerland; 25 Library and Information Services, Canadian Memorial Chiropractic College, Toronto, Canada

**Keywords:** Acupuncture, Dry needling, Low back pain, Pain, Function, Systematic review, Meta-analysis

## Abstract

**Purpose:**

Evaluate benefits and harms of needling therapies (NT) for chronic primary low back pain (CPLBP) in adults to inform a World Health Organization (WHO) standard clinical guideline.

**Methods:**

Electronic databases were searched for randomized controlled trials (RCTs) assessing NT compared with placebo/sham, usual care, or no intervention (comparing interventions where the attributable effect could be isolated). We conducted meta-analyses where indicated and graded the certainty of evidence.

**Results:**

We screened 1831 citations and 109 full text RCTs, yeilding 37 RCTs. The certainty of evidence was low or very low across all included outcomes. There was little or no difference between NT and comparisons across most outcomes; there may be some benefits for certain outcomes. Compared with sham, NT improved health-related quality of life (HRQoL) (physical) (2 RCTs; SMD = 0.20, 95%CI 0.07; 0.32) at 6 months. Compared with no intervention, NT reduced pain at 2 weeks (21 RCTs; MD = − 1.21, 95%CI − 1.50; − 0.92) and 3 months (9 RCTs; MD = − 1.56, 95%CI − 2.80; − 0.95); and reduced functional limitations at 2 weeks (19 RCTs; SMD = − 1.39, 95%CI − 2.00; − 0.77) and 3 months (8 RCTs; SMD = − 0.57, 95%CI − 0.92; − 0.22). In older adults, NT reduced functional limitations at 2 weeks (SMD = − 1.10, 95%CI − 1.71; − 0.48) and 3 months (SMD = − 1.04, 95%CI − 1.66; − 0.43). Compared with usual care, NT reduced pain (MD = − 1.35, 95%CI − 1.86; − 0.84) and functional limitations (MD = − 2.55, 95%CI − 3.70; − 1.40) at 3 months.

**Conclusion:**

Based on low to very low certainty evidence, adults with CPLBP experienced some benefits in pain, functioning, or HRQoL with NT; however, evidence showed little to no differences for other outcomes.

**Supplementary Information:**

The online version contains supplementary material available at 10.1007/s10926-023-10125-3.

## Introduction

Needling therapies are commonly used for the management of pain, including chronic primary low back pain (CPLBP) [1]. These interventions involve inserting fine, solid metallic needles through the skin at specific sites. One type of needling therapy is Traditional Chinese Medicine (TCM) acupuncture, whereby the clinician aims to alter the body’s vital energy *qi* flows along 12 primary and eight secondary meridians in the body [2]. According to TCM, pain occurs when *qi* is blocked; however, the proper flow of *qi* can be restored with the insertion of acupuncture needles at specific points along the meridians [2, 3]. A second type of needling therapy is myofascial acupuncture, or dry needling [4]. It is used to treat myofascial pain by inserting needles (acupuncture needle or other hypodermic needle) into myofascial trigger points and/or tender points. The needles used with both traditional and myofascial acupuncture can be further stimulated manually, with small electrical currents (electroacupuncture), moxibustion (burning the moxa herb at the needle handle), or heat lamps [5]. Various mechanisms have been proposed regarding how needling therapies may help relieve pain. Needle insertion may inhibit the perception of pain via the gate control theory of pain [6]; the release of endogenous opioids [7]; or local physiological and blood flow changes [8, 9].

Mu and colleagues published a Cochrane systematic review (2020) to assess the effects of needling therapies compared to sham intervention, no treatment, or usual care for chronic non-specific LBP (33 randomized controlled trials (RCTs); 8270 participants; search end date of August 2019) [10]. They found that compared with sham, needling therapies may not be more effective in reducing pain or improving back-specific functioning immediately after treatment, or quality of life in the short term. Compared with no treatment, needling therapies were not beneficial for pain relief and functional improvement immediately after treatment. Compared with usual care, needling therapies may improve function, but not pain, immediately after treatment; and did not improve quality of life in the short term. Adverse events related to needling therapies were minor or moderate, and the incidence was similar with sham and usual care. To develop clinical practice recommendations for the management of CPLBP in adults, the World Health Organization (WHO) commissioned the current systematic review to update the evidence and expand the aims of the Cochrane review [10]. The current review aimed to assess additional important outcomes, and subgroups.

The objectives of this systematic review of RCTs were to determine: (1) the benefits and harms (as reported in RCTs) of needling therapies compared with placebo/sham, usual care, or no intervention for the management of CPLBP in adults, including older adults (aged ≥ 60 years); and (2) whether the benefits and harms of needling therapies vary by age, gender/sex, presence of leg pain, race/ethnicity, or national economic development of the countries where the RCTs were conducted.

## Methods

This systematic review was conducted as part of a series of reviews to inform the WHO guideline on the management of CPLBP in adults. CPLBP is defined as pain of more than three months duration between the lower costal margin and the gluteal fold with no specific underlying cause. Guideline development was ongoing at the time of submission of this manuscript. The methods are detailed in the methodology article of this series [11].

Briefly, we updated and expanded the scope of the previously published high-quality Cochrane systematic review by Mu et al. [10]. We registered our review protocol with Prospero (CRD42022314824) on 7 March 2022. We searched the Cochrane Central Register of Controlled Trials (Wiley), MEDLINE (Ovid), Embase (Ovid), Cumulative Index to Nursing and Allied Health Literature (CINAHL) (EBSCO), China National Knowledge Infrastructure database (CNKI), WangFang database, and the World Health Organization International Clinical Trials Registry Platform (ICTRP) from the period of 1 August 2019 (end date of previous Cochrane review) to 9 March 2022 (see Online Resource 1). We also searched the reference lists of systematic reviews and included RCTs.

We included RCTs that compared needling therapies to placebo/sham (e.g., sham needling without skin penetration or at non-acupuncture points), usual care, no intervention (including comparison interventions where the attributable effect of needling therapies could be isolated e.g., needling therapy + medication vs. same medication alone) in adults (aged ≥ 20 years) with CPLBP. Eligible needling therapies included those conducted according to TCM theory or myofascial acupuncture methods (i.e., dry needling including neuroreflexotherapy, western medical acupuncture). Specifically, we included interventions where needles were inserted into classical meridian points, tender points, or trigger points through the skin. Manual stimulation, heating by moxa, heat lamps, cupping or electrical current stimulation could be further administered to maximize the treatment effect [10]. We excluded trials that did not use hypodermic needles (e.g., acupressure, laser acupuncture). Further details on the eligibility criteria can be found in the methodology article in this series [11]. In addition to the main critical outcomes requested by the WHO Guideline Development Group (GDG) and assessed for all reviews in this series (pain, function, health-related quality of life (HRQoL), harms/adverse events, psychological functioning, and social participation including work), we also assessed additional critical outcomes requested by the WHO GDG for this review—the change in use of medications and falls in older adults aged ≥ 60 years. These additional outcomes were deemed important for people and their caregivers and had the potential to inform future research. We reported outcomes based on post-intervention follow-up intervals including: (1) immediate term (closest to 2 weeks after the intervention period); (2) short term (closest to 3 months after the intervention period); (3) intermediate term (closest to 6 months after the intervention period); (4) long term (closest to 12 months after the intervention period); and (5) extra-long term (more than 12 months after the intervention period).

We assessed between-group differences to determine the magnitude of the effect of an intervention and to assess its effectiveness [12, 13] (details in the methodology article in this series) [11]. Briefly, we considered a mean difference (MD) of ≥ 10% of the scale range or ≥ 10% difference in risk for dichotomous outcomes to be a minimally important difference (MID) [14, 15]. If the standardized mean difference (SMD) was calculated, SMD ≥ 0.2 was considered a MID [16].

Pairs of reviewers independently screened studies for eligibility, and critically appraised risk of bias (ROB) using the Cochrane ROB 1 tool [17], modified from the Cochrane Back and Neck Methods Guidelines [18]. One reviewer extracted data for all included RCTs, which was then verified by a second reviewer. Any disagreements were resolved by consensus between paired reviewers or with a third reviewer when necessary. Forms and guidance for screening, ROB assessment, and data extraction were adapted from those used by Hayden et al. in the conduct of the ‘exercise for chronic low back pain’ collaborative review, in which members of our team participated [19]. The forms were modified and completed using a web-based electronic systematic review software DistillerSR Inc. [20].

In addition to the main sub-group analyses conducted for all reviews in this series (age, gender/sex, presence of leg pain, race/ethnicity, national economic development of country where RCT was conducted), we conducted the following pre-specified sub-group analyses: needling therapy type (i.e., TCM or myofascial acupuncture), and stimulation used with needling therapies (e.g., electrical, manual, moxibustion). We conducted sensitivity analyses by excluding RCTs judged to have high risk of bias.

We performed random-effects meta-analyses and narrative synthesis where meta-analysis was not appropriate [21], and graded the certainty of evidence using Grading of Recommendations Assessment, Development and Evaluation (GRADE) [22]. Comparisons to no intervention and sets of interventions where the specific attributable effect of needling therapies could be isolated (e.g., needling therapy + treatment B versus treatment B alone) were combined in meta-analyses. Meta-analyses were conducted using R [23, 24]. GRADE Evidence Profiles and GRADE Summary of Findings tables were developed using GRADEpro software [25].

## Results

**Fig. 1:**
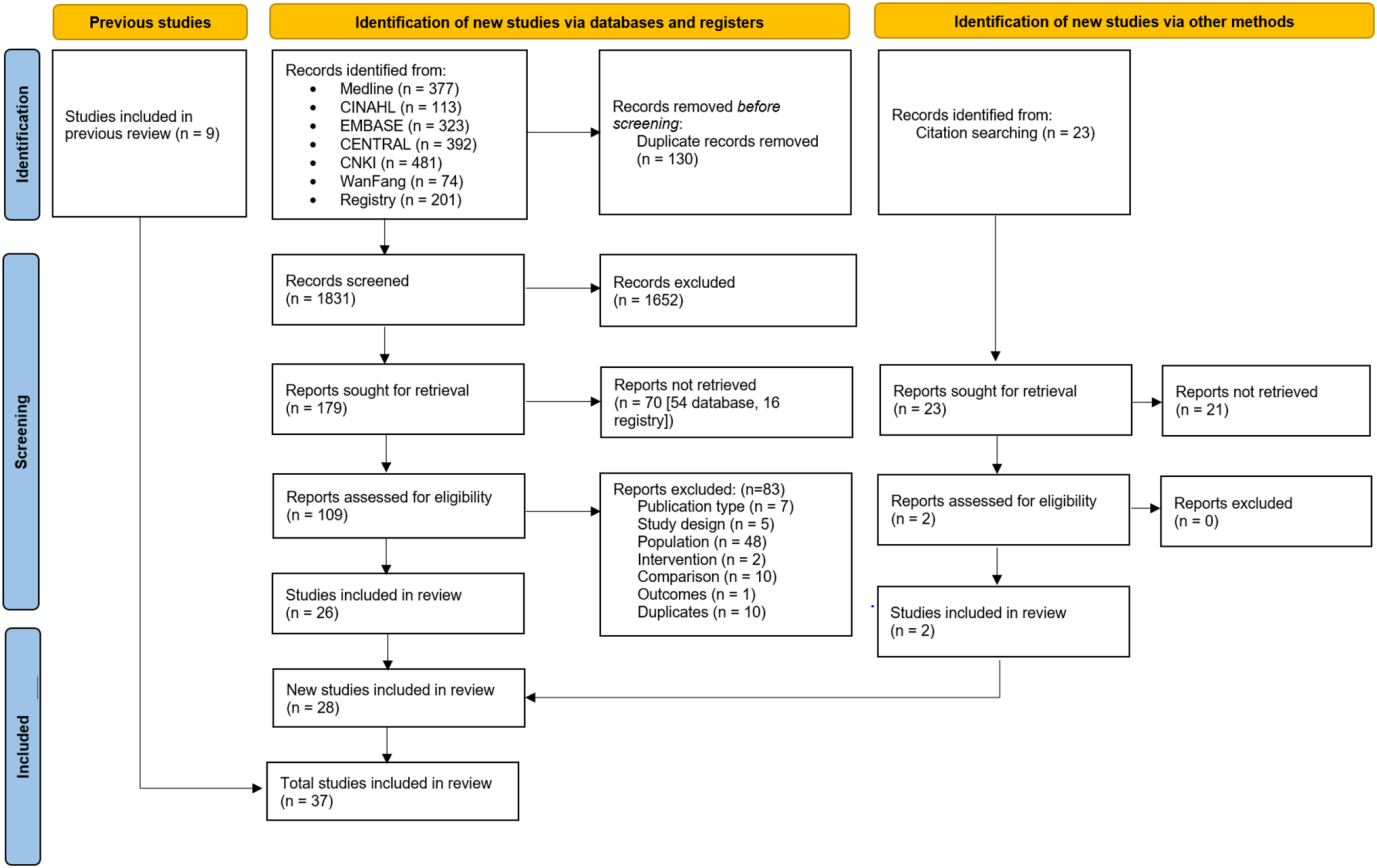
Flow diagram of literature search

We screened 1831 records and 109 full-text reports (Fig. 1). We identified 16 unpublished RCTs in the WHO ICTRP, of which we contacted the authors who had contact information listed (2 of 16). Both authors responded to inform us that the RCTs were ongoing. Thus, none of the 16 unpublished RCTs identified in the WHO ICTRP were included. We included 37 published RCTs [26–62] with a total of 7573 adults ranging from 32 to 3093 adults per RCT (see Online Resources 2, 3).

The RCTs were conducted in high-income economies [63]: Germany (5 RCTs) [26, 32, 41, 52, 53], Ireland (1 RCT) [34], Korea (2 RCTs) [28, 47], Spain (1 RCT) [39], and the United States (6 RCTs) [27, 36, 40, 55, 62, 64]; upper-middle income economies: Brazil (4 RCTs) [29–31, 49] and China (15 RCTs) [33, 37, 38, 37–38, 37–38, 54, 37–38, 37–38]; and lower-middle income economies: Iran (3 RCTs) [42, 48, 58]. The mean age ranged from 30 to 72 years; one RCT assessed older adults (n = 55) [40]. The percentage of females within the trials ranged from 0 to 85%. Thirteen of 37 RCTs (35%) were published in a Chinese language.

In 7 RCTs, adults had CPLBP with radicular leg pain [33, 43, 50, 51, 56, 50–51]; in 15 RCTs, adults had CPLBP without leg pain [26, 31, 35–38, 35–38, 47, 49, 53, 35–38, 35–38]; in 3 RCTs, adults had CPLBP either with or without leg pain (radicular or non-radicular) [27, 48, 54]; and presence of leg pain was not reported in 12 RCTs [28–30, 32, 34, 39, 42, 28–30, 52, 55]. Where reported by authors, CPLBP duration ranged from 4 months to 15 years.

Most RCTs assessed needling therapies based on TCM (31/37, 84%); while 6 of 37 RCTs (16%) assessed mysofascial acupuncture [26, 36, 39, 42, 48, 62]. Different stimulation types were used. Seven RCTs included electrical stimulation [31, 36, 40, 42, 54, 56, 58], 16 RCTs manual stimulation [26, 28, 34, 35, 38, 41, 34–35, 50, 55, 57, 34–35, 62], 2 RCTs moxibustion [44, 61], 1 RCT heated needles [51], and 1 RCT electromagnetic wave therapy device (1 RCT) [60]. The number of sessions delivered ranged from 1 to 40, with the duration of each session ranging from 10 to 45 min. Needling therapies were compared to sham (sham acupuncture or sham electrotherapy [no needles penetrating skin and no electronic stimulation]) (15 RCTs) [26–28, 30, 26–28, 39, 41, 49, 55, 57, 62]; usual care (1 RCT) [27]; no intervention (6 RCTs) [26, 30, 48, 53, 57, 58]; or comparison interventions where the attributable effect of needling therapies could be isolated (20 RCTs) [31, 37, 38, 37–38, 37–38, 37–38, 54, 56, 37–38, 65].

The outcomes were assessed in the immediate term (31 RCTs) [29, 31–34, 31–34, 31–34], short term (19 RCTs) [26–33, 26–33, 26–33, 50, 26–33, 26–33], intermediate term (5 RCTs) [26–28, 26–28], and long term (2 RCTs) [26, 27]. The majority of RCTs assessed pain and function in the immediate and short terms (Table 1). Most of the RCTs (30, 81%) were rated as high overall ROB, five (13.5%) were rated as unclear overall ROB, and two (5.5%) were rated as low overall ROB (see Online Resource 4). The agreement on overall ROB ratings was high (weighted overall kappa score 0.94).

**Table 1:**
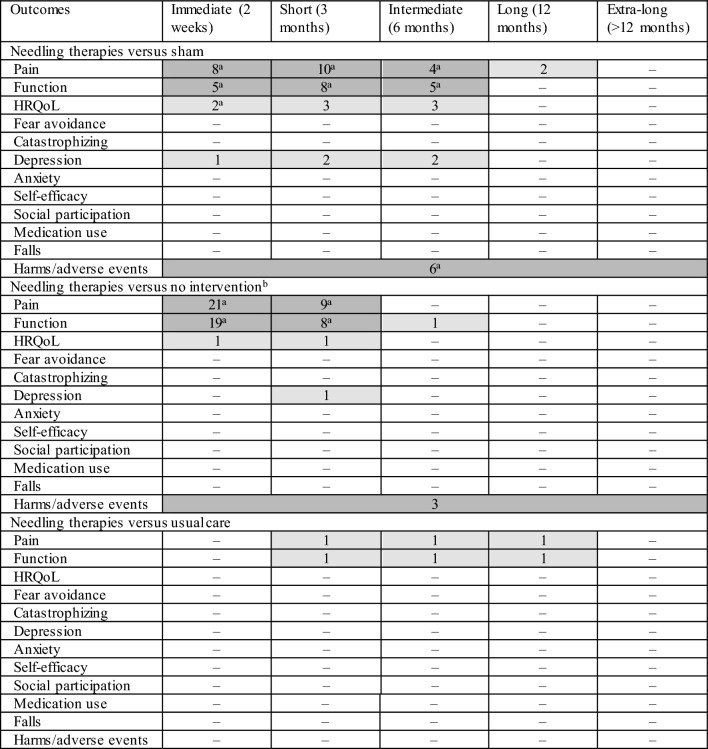
Number of included RCTs by comparison and outcome

Dark-shade grey: majority of studies are in this category; medium-shade grey: some studies; white: zero studies

*HRQoL* health-related quality of life

^a^Includes 1 RCT of older adults (aged ≥ 60 years)

^b^Includes comparison interventions where the attributable effect of needling therapies could be isolated

### Certainty of Evidence

The certainty of the evidence for all outcomes was low or very low, downgraded due to risk of bias, inconsistency, indirectness, and imprecision of the effect estimates (see Online Resources 5, 6 and 7).

### Needling Therapies Versus Sham

#### All Adults

With low certainty evidence, needling therapies may reduce pain (scale 0 to 10, 0 = *no pain)* in the immediate term (7 RCTs; MD = − 0.41, 95% CI − 0.72 to − 0.10) (effect estimate did not reach the threshold for a minimally important between-group difference, MD = − 1) (see Online Resource 7, plot 1.1.1) [32, 34, 39, 41, 57, 62, 66]. Needling therapies may make little or no difference to pain in the intermediate (4 RCTs; MD − 0.21, 95% CI − 0.58 to 0.16) (plot 1.1.3) [26–28, 32], or long term (2 RCTs; MD = − 0.02, 95% CI − 0.51 to 0.47) (plot 1.1.4) [26, 27]. Due to very low certainty evidence, it is uncertain whether needling therapies make little or no difference to pain in the short term (9 RCTs; MD = − 0.42, 95% CI − 0.88 to 0.05) (plot 1.1.2) [26–28, 30, 32, 35, 39, 41, 57].

Due to very low certainty evidence, it is uncertain whether needling therapies make little or no difference to ***functional limitations*** *(benefit indicated by lower values)* in the immediate (4 RCTs; SMD = − 0.22, 95% CI − 0.54 to 0.11) (plot 1.2.1) [32, 39, 57, 62], or intermediate term (4 RCTs; SMD = − 0.10, 95% CI − 0.22 to 0.02) (plot 1.2.3) [26–28, 32]. Due to low certainty evidence, needling therapies may make little or no difference to function in the short term (7 RCTs; SMD = − 0.03, 95% CI − 0.17 to 0.11) (plot 1.2.2) [26–28, 30, 32, 39, 57].

Due to very low certainty evidence, it is uncertain whether needling therapies make little or no difference to ***HRQoL*** *(scale 0 to 100, 0* = *poor HRQoL, benefit indicated by higher values; PCS: physical component summary, MCS: mental compenent summary)* in the immediate term (1 RCT; MD = 6.40, 95% CI − 6.42 to 19.22) (plot 1.3.1) [34]. In the short term, it is uncertain whether needling therapies improve HRQoL (1 RCT; MD = 7.78, 95% CI 1.41 to 14.15) (plot 1.3.2) (effect estimate did not reach the threshold for a minimally important between-group difference, MD = 10) [34], or make little or no difference to HRQoL (PCS) (2 RCTs; SMD = 0.25, 95% CI − 0.07 to 0.56) (plot 1.3.2.1) [26, 32]. In the intermediate term, it is uncertain whether needling therapies make little or no difference to HRQoL (1 RCT; MD = 3.39, 95% CI − 2.98 to 9.76) (plot 1.3.3) [28], or to HRQoL (MCS) (2 RCTs; SMD = 0.10, 95% CI − 0.18 to 0.39) (plot 1.3.3.2) [26, 32]. Due to low certainty evidence, needling therapies may make little or no difference to HRQoL (MCS) in the short term (2 RCTs; SMD = 0.01, 95% CI − 0.12 to 0.14) (plot 1.3.2.2) [26, 32]; and may improve HRQoL (PCS) in the intermediate term (2 RCTs; SMD = 0.20, 95% CI 0.07 to 0.32) (plot 1.3.3.1) [26, 32].

Due to very low certainty evidence, it is uncertain whether needling therapies make little or no difference to ***depression*** *(scale 0 to 60, benefit indicated by lower values)* in the immediate (1 RCT; MD = − 2.50, 95% CI − 5.23 to 0.23) ( plot 1.4.1) [26], short (2 RCTs; SMD = − 0.17, 95% CI − 0.44 to 0.10) (plot 1.4.2) [26, 28], or intermediate term (2 RCTs; SMD = − 0.10, 95% CI − 0.33 to 0.12) (plot 1.4.3) [26, 28].

Due to very low certainty evidence, it is uncertain whether needling therapies make little or no difference to ***adverse events/harms*** (6 RCTs; odds ratio (OR) = 1.62, 95% CI 0.67 to 3.90) (plot 1.5) [26, 27, 33, 36, 57, 62].

#### Older Adults (Aged ≥ 60 years)

Based on one RCT [33] and reported as a narrative synthesis of the authors’ findings (no corresponding plots), there is very low certainty evidence for all outcomes.

It is uncertain whether needling therapy makes little or no difference to: ***pain*** *(scale 0 to 100, 0* = *no pain)* in the immediate (MD = − 6.85, 95% CI − 16.82 to 3.11), short (MD = − 6.06, 95% CI − 18.50 to 6.38), or intermediate term (MD = − 7.01, 95% CI − 17.50 to 3.48); ***function*** *(scale 0 to 100, 0* = *no disability)* in the immediate (MD = − 4.52, 95% CI − 13.05 to 4.01), short (MD = − 3.04, 95% CI − 12.34 to 6.25), or intermediate term (MD = 0.09, 95% CI − 10.80 to 10.98); or ***HRQoL*** in the immediate term (effect estimates not provided). Authors reported no serious ***harms/adverse events*** occurred during the 4-week RCT; 2 of 46 participants total (4.3%) had subcutaneous hematoma after needling (both from the needling therapy group) [33].

### Needling Therapies Versus no Intervention or Comparison Interventions Where the Attributable Effect of Needling Therapies Could be Isolated

#### All Adults

**Fig. 2:**
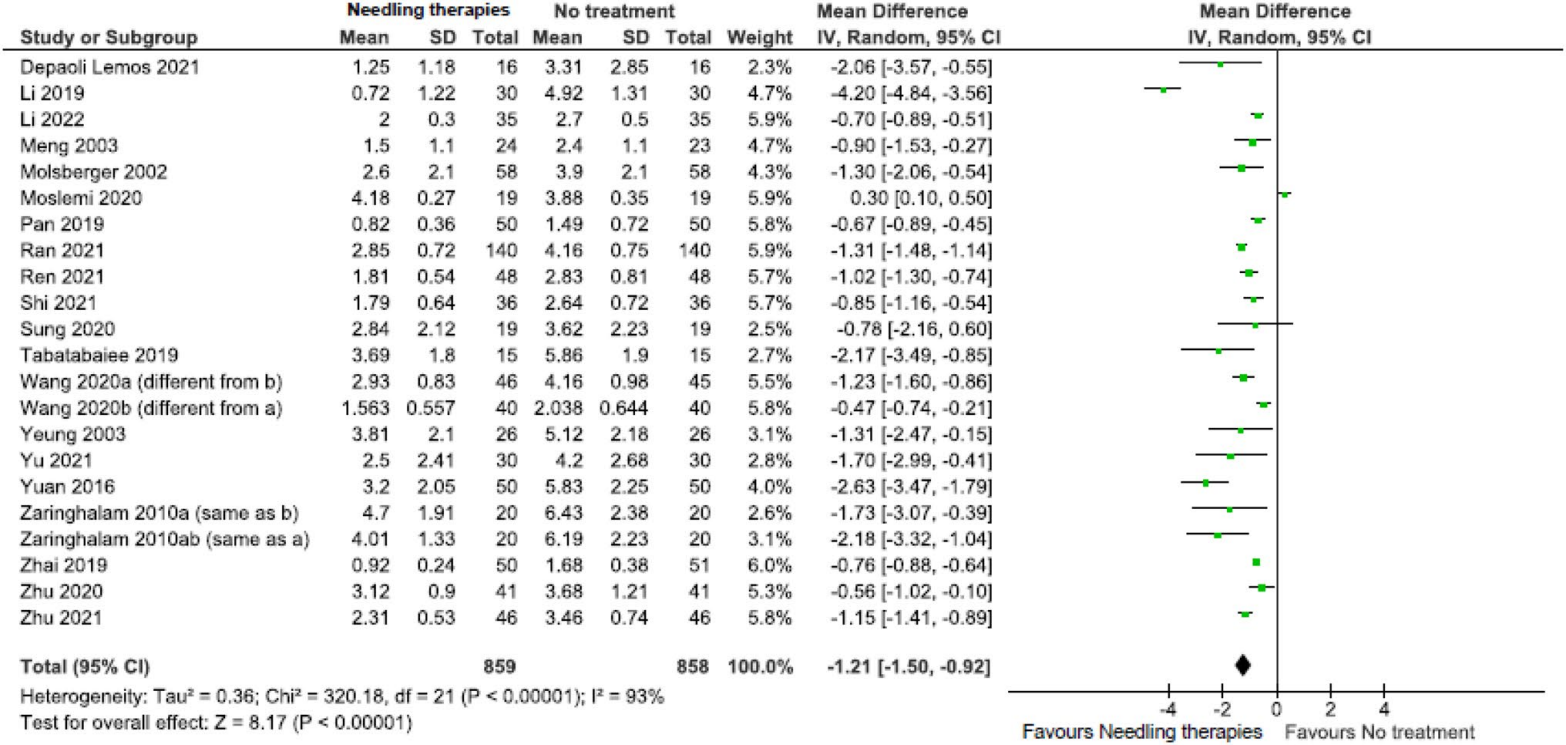
Needling therapies versus no intervention, and needling therapies versus additional comparison interventions where the attributable effect of could be isolated for pain in the immediate term (closest to 2 weeks); scale range is 0 to 10

**Fig. 3:**
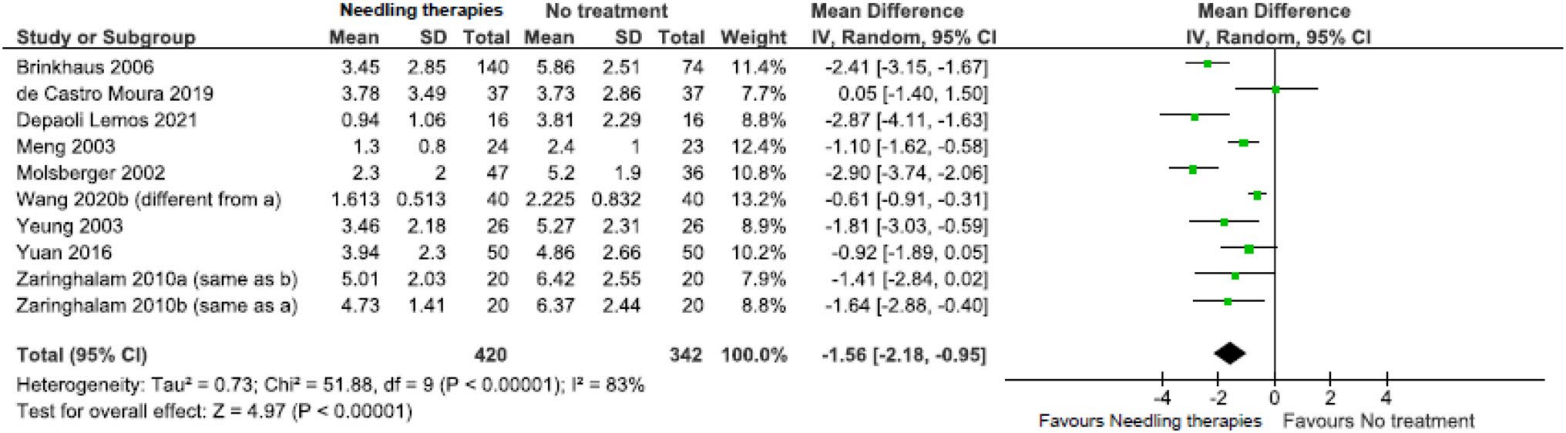
Needling therapies versus no intervention, and needling therapies versus additional comparison interventions where the attributable effect could be isolated for pain in the short term (closest to 3 months); scale range is 0 to 10

Due to low certainty evidence, needling therapies may reduce ***pain*** *(scale 0 to 10, 0* = *no pain)* in the immediate (21 RCTs; MD = − 1.21, 95% CI − 1.50 to − 0.92) (Fig. 2, Online Resource 7, plot 2.1.1) [31, 37, 38, 37–38, 37–38, 54, 37–38], and short term (9 RCTs; MD = − 1.56, 95% CI − 2.80 to − 0.95) (Fig. 3, plot 2.1.2) [26, 30, 31, 30–31, 50, 54, 57].

**Fig. 4:**
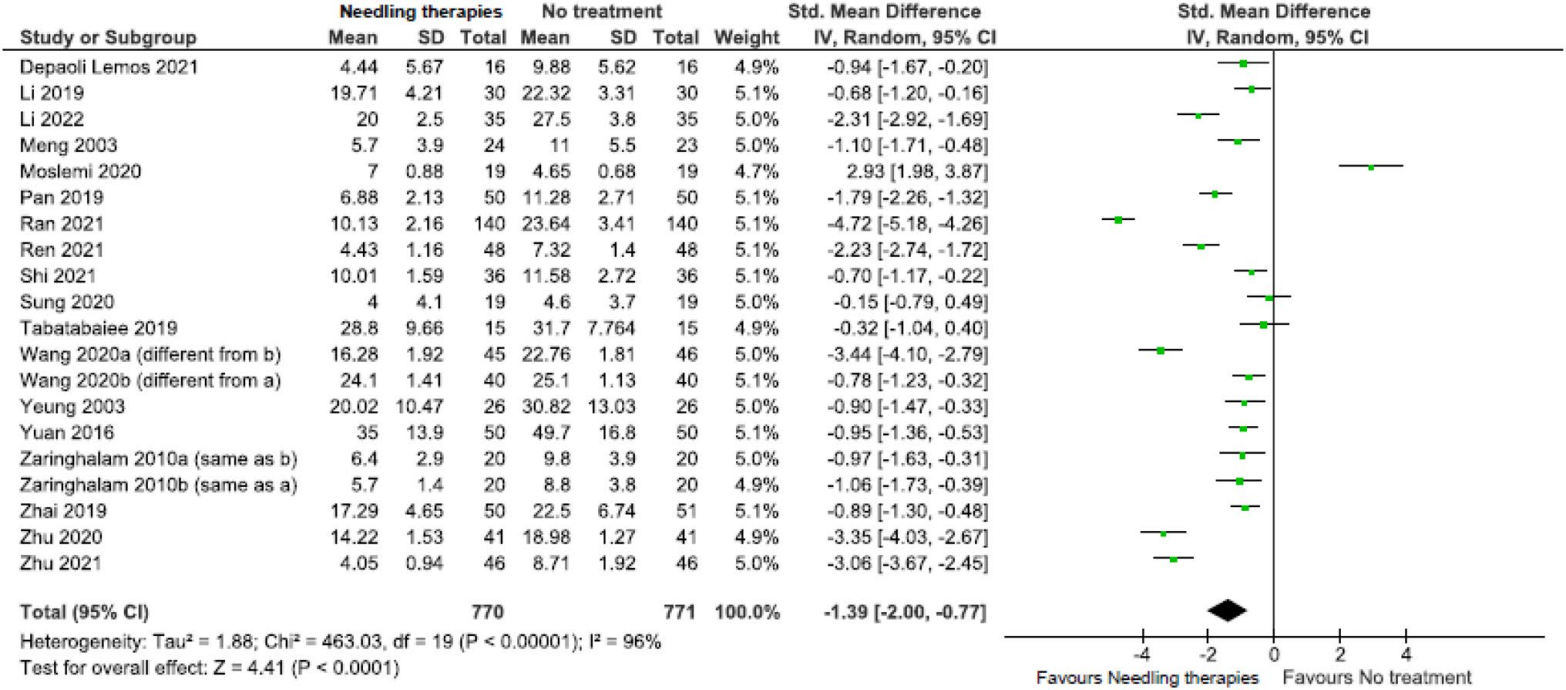
Needling therapies versus no intervention, and needling therapies versus additional comparison interventions where the attributable effect could be isolated for function in the immediate term (closest to 2 weeks)

Due to low certainty evidence, needling therapies may reduce ***functional limitations*** *(scale 0 to 100, 0* = *no functional limitations; benefit indicated by lower values)* in the immediate (19 RCTs; SMD = − 1.39, 95% CI − 2.00 to − 0.77) (Fig. 4, Online Resource 7, plot 2.2.1) [31, 37, 38, 40, 37–38, 37–38, 54, 57, 37–38], and short term (8 RCTs; SMD = − 0.57, 95% CI − 0.92 to − 0.22) (plot 2.2.2) [26, 30, 31, 40, 50, 54, 30–31]. Due to very low certainty evidence, it is uncertain whether needling therapy reduces functional limitations in the intermediate term (1 RCT; MD = − 8.30, 95% CI − 13.93 to − 2.67) (effect estimate did not reach the thresholds for a minimally important between-group difference, MD = − 10) (plot 2.2.3) [26].

Due to very low certainty evidence, it is uncertain whether needling therapy makes little or no difference to ***HRQoL*** *(scale 0 to 1, 0* = *poor HRQoL)* in the immediate term (1 RCT; MD = 0.02, 95% CI − 0.09 to 0.14) (plot 2.3.1) [47]. In the short term, it is uncertain whether needling therapy improves ***HRQoL*** *(scale 0 to 100, 0* = *poor quality of life; PCS: physical component summary, MCS: mental component summary)* with respect to the PCS (1 RCT; MD = 6.60, 95% CI 3.90 to 9.30) (effect estimate did not reach the threshold for a minimally important between-group difference, MD = 10) (plot 2.3.2.1) [26]. It is uncertain whether needling therapy makes little or no difference to HRQoL (MCS) (1 RCT; MD = 1.20; 95% CI − 1.86 to 4.26) (plot 2.3.2.2) [26].

Due to very low certainty evidence, it is uncertain whether needling therapy makes little or no difference to ***depression*** *(scale 0 to 60, 0* = *no depression)* in the short term (1 RCT; MD = − 0.80, 95% CI − 3.60 to 2.00) (plot 2.4.1) [26].

Due to very low certainty evidence, it is uncertain whether needling therapies make little or no difference to ***adverse events/harms*** (3 RCTs; OR = 3.12, 95% CI 0.42 to 23.44) (plot 2.5) [34, 49, 57].

#### Older Adults (Aged ≥ 60 Years)

One RCT assessed older adults [40]. Due to very low certainty evidence, it is uncertain if needling therapy reduces: ***pain*** *(scale 0 to 10, 0* = *no pain)* in the immediate (MD = − 0.90, 95% CI − 1.53 to − 0.27) (effect estimate did not reach the threshold for a minimally important between-group difference, MD = − 1) (plot.2.6.1.1), or short term (MD = − 1.10, 95% CI − 1.62 to − 0.58) (plot2.6.2.1); or ***functional limitations*** *(benefit indicated by lower values)* in the immediate (SMD = − 1.10, 95% CI − 1.71 to − 0.48) (plot 2.7.1.1), or short term (SMD = − 1.04, 95% CI − 1.66 to − 0.43) (plot 2.7.2.1). No RCTs assessed harms/adverse events.

### Needling Therapies Versus Usual Care

#### All Adults

One trial assessed benefits [27]. Due to very low certainty evidence, it is uncertain whether needling therapy reduces ***pain*** *(scale 0 to 10, 0* = *no pain)* in the short (MD = − 1.35, 95% CI − 1.86 to − 0.84) (plot 3.1.1), or intermediate term (MD = − 0.65, 95% CI − 1.17 to − 0.13) (effect estimate did not reach the threshold for a minimally important between-group difference, MD = − 1) (plot 3.2.1); or whether needling therapy makes little or no difference to pain in the long term (MD = − 0.50, 95% CI − 1.02 to 0.02) (plot 3.1.3). It is uncertain whether needling therapy reduces ***functional limitations*** *(scale 0 to 24, 0* = *no functional limitations)* in the short (MD = − 2.55, 95% CI − 3.70 to − 1.40) (plot 3.2.1), intermediate (MD = − 1.65, 95% CI − 2.83 to − 0.47) (plot 3.2.2), or long term (MD = − 1.90, 95% CI − 3.15 to − 0.65) (effect estimate did not reach the threshold for a minimally important between-group difference for the latter 2 timepoints, MD = − 2.4) (plot 3.2.3). No RCTs assessed harms/adverse events.

### Results of Subgroup and Sensitivity Analyses

The subgroups were small (consisting of 1 to 3 RCTs per group), yielding small, imprecise pooled effects (see Online Resource 7). In addition, there was inadequate reporting of some factors, such as gender/sex or presence of leg pain in participants, which further limited the usefulness of the pooled results. Despite limitations, subgroup effects were overall in line with those of the main analysis.

We examined publication bias using funnel plots when the number of RCTs per comparison was 10 or more. Two funnel plots (needling therapy versus no treatment for pain and function in the immediate term) showed no substantial publication bias (plots 4.1.1, 4.2.1).

## Discussion

Recent evidence regarding the benefits and harms of needling therapies for CPLBP in adults is based on 37 RCTs (n = 7573 total adults, n = 55 older adults). Most of the RCTs (30, 81%) were rated as having a high overall ROB and the certainty of the evidence for all outcomes ranged from low to very low. For most outcomes, there was little or no difference between needling therapies and comparison interventions. Evidence suggested the following clinically important benefits for certain outcomes (with low to very low certainty): (1) compared to sham, evidence suggested a small improvement in HRQoL (physical) in the intermediate term; (2) compared to no intervention (including comparison interventions where the attributable effect of needling therapies could be isolated), evidence suggested small reductions in pain in the immediate and short terms, and large and moderate reductions in functional limitations in the immediate and short term, respectively. In older adults, evidence suggested a larger reduction in functional limitations in the immediate and short terms; (3) compared to usual care, evidence suggested a small reduction in pain and functional limitations in the short term. Of nine RCTs reporting on harms/adverse events, 4.3% (2/46) older adults reported mild subcutaneous hematoma after needling compared with sham needling (1 RCT).

Our findings align with those by Mu et al. [10], as discussed at the onset. They also align with Asano et al. [67], who stated that acupuncture as an adjunct to usual care may provide some benefits in reducing immediate and short-term pain and disability among adults with CPLBP (|no other outcomes were assessed).

Our systematic review has several strengths. First, we had an expert review team that included international clinical and methodological experts in the fields of LBP, systematic reviews and evidence syntheses, and answering important policy questions from the WHO. Second, our review employed comprehensive and peer-reviewed literature search strategies that did not have any language restrictions. Third, we ensured that at least half of the screening and ROB pairs were formed by a member of the core team, who was the most trained and reliable in screening and ROB judgements. Fourth, unlike other systematic reviews that relied on the number of items at risk of bias or summary scores for ROB assessments, we developed and used adjunct guidance forms based on the ROB1 criteria, which allowed reviewers to consider important critical flaws [11]. Consequently, there was high agreement for overall ROB ratings. Finally, we maintained transparency in all review steps by providing detailed ROB assessments and footnotes for grading the certainty of the evidence (see Online Resources 4, 5). This approach allowed readers to understand how we came to our judgements, enabling them to make their own judgements and conclusions.

There are potential limitations to our systematic review. First, despite our rigorous literature search strategies, we may have missed some potentially relevant RCTs, especially as we were unable to retrieve and assess the eligibility of 54 full-text RCTs identified from our database search (mainly due to restricted international access). This may have increased or decreased our effect estimates. Second, our pre-specified eligibility criteria was restricted to published trials and did not search the grey literature. However, we do not have strong evidence to suggest our review was impacted by publication bias, based on our publication bias assessment. We searched for unpublished RCTs in the WHO ICTRP registry and contacted authors of unpublished RCTs. However, only two authors responded, and the reason given for non-publication was ongoing RCTs. While we are uncertain how publication bias may have impacted our findings, unpublished studies have been shown to represent a small proportion of studies and rarely impact the results and conclusions [68, 69]. Finally, the decision to combine different sets of comparison interventions in meta-analysis (i.e., no intervention and sets of interventions where the attributable effect of needling therapies could be isolated) may have contributed to inconsistent pooled effects.

We identified evidence gaps across all comparisons. First, there were some outcomes for which there were no RCTs, mainly psychological outcomes (including fear avoidance, catastrophizing, anxiety, and self-efficacy), social participation including work; or change in medication use or falls in older adults. Further research is needed to explore these outcomes comprehensively.

For the comparison of needling therapies to sham, we found no RCTs to inform the long-term benefits on function, or depression. For the comparison of needling therapies to no intervention or interventions where the attributable effect of needling therapies could be isolated, we found no RCTs to inform the intermediate- to long-term benefits on pain, HRQoL and depression and no RCTs to inform the long-term benefits on function. Finally, we identified a scarcity of pragmatic RCTs comparing needling therapies with usual care, which may limit our understanding of their comparative effectiveness in real-world settings.

In terms of assessing benefits and harms, subgroup analyses posed challenges, particularly when considering mode of delivery and spine-related leg pain. This limitation may prevent guideline developers from providing specific case mix recommendations for clinical practice. Moreover, the reporting of harms/adverse events across studies was inadequate and inconsistent. This is particularly concerning for older adults and individuals taking anti-coagulants, as it hampers our ability to assess potential risks accurately.

## Conclusion

Based on low or very low certainty evidence, adults with CPLBP experienced small improvements with respect to pain and HRQoL (physical), and both adults and older adults experienced larger reductions in functional limitations. Needling therapy was associated with a small increased risk of subcutaneous hematoma in older adults, compared to sham needling. The remaining evidence showed little to no difference in benefits between needling therapies and comparisons for other outcomes, such as depression. Patient care plans should be developed through a collaborative decision-making process, that carefully considers scientific evidence, cost-effectiveness, and relevant contextual factors, such as values and preferences of users of needling therapies. Adverse events should be investigated systematically.

### Supplementary Information

Below is the link to the electronic supplementary material.

Supplementary file1 (DOCX 6077 kb)

## Data Availability

The datasets generated during and/or analysed during the current study are available from the corresponding author on reasonable request.
