# Supplementary material for: Systematic Review to Inform a World Health Organization (WHO) Clinical Practice Guideline: Benefits and Harms of Needling Therapies for Chronic Primary Low Back Pain in Adults
Source: J Occup Rehabil. 2023 Nov 22;33(4):661–72. doi: 10.1007/s10926-023-10125-3 (PMC10684627; doi:10.1007/s10926-023-10125-3)
Supplement: Supplementary file 1 — Supplementary file1 (DOCX 6077 kb) [file 10926_2023_10125_MOESM1_ESM.docx]

Systematic review to inform a World Health Organization (WHO) clinical practice guideline: Benefits and harms of needling therapies for chronic primary low back pain in adults: Supplementary Information

Yu H, Wang D, Verville L, Southerst D, Bussières A, Gross DP, Pereira P, Mior S, Tricco AC, Cedraschi C, Brunton G, Nordin M, Shearer HM, Wong JJ, Connell G, Myrtos D, da Silva-Oolup S, Young J, Funabashi M, Romanelli A, Lee J, Stuber K, Guist B, Muñoz Laguna J, Hofstetter L, Murnaghan K, Hincapié CA, Cancelliere C

**Corresponding authors:**

Carol Cancelliere

Email: [carolina.cancelliere@ontariotechu.ca](mailto:carolina.cancelliere@ontariotechu.ca)

Cesar A. Hincapié

Email: [cesar.hincapie@uzh.ch](mailto:cesar.hincapie@uzh.ch)

Acknowledgments: This work was funded by the World Health Organization (Switzerland/Ageing and Health Unit)

**Online Resource 1.** Literature search strategies

**A. Database & Platform:**  MEDLINE (Ovid)

**Years of search:** August 1, 2019 – March 9, 2022

**Date search run:**  March 9, 2022 **Number of records retrieved:** 377

**Search Strategy:**

1. Low Back Pain/
2. exp Back Pain/
3. Back Injuries/
4. exp Back Muscles/in [Injuries]
5. Intervertebral Disc Degeneration/
6. Intervertebral Disc Displacement/
7. Intervertebral Disc/in [Injuries]
8. Lumbar Vertebrae/in [Injuries]
9. Lumbosacral Plexus/in [Injuries]
10. Lumbosacral Region/in [Injuries]
11. Coccyx/in [Injuries]
12. Osteoarthritis, Spine/
13. Osteoarthritis/
14. Piriformis Muscle Syndrome/
15. Polyradiculopathy/
16. Sacroiliac Joint/in [Injuries]
17. Sciatica/
18. Spinal Curvatures/
19. Spinal Diseases/
20. Spinal Injuries/
21. Spinal Stenosis/
22. exp Spondylolysis/
23. Spondylosis/
24. Synovial Cyst/
25. Zygaphophyseal Joint/in [Injuries]
26. ((low* adj2 (back adj2 pain*)) or (low-back* adj2 pain*) or (lower-back* adj2 pain*) or (low* adj2 back-pain*)).mp.
27. ((low* adj2 (back adj2 injur*)) or (low-back* adj2 injur*) or (lower-back* adj2 injur*) or (low* adj2 back-injur*)).mp.
28. ((low* adj2 (back adj2 trauma*)) or (low-back adj2 trauma*) or (lower-back* adj2 trauma*) or (low* adj2 back-trauma*)).mp.
29. ((low* adj2 (trunk adj2 pain*)) or (lower-trunk* adj2 pain*) or (low* adj2 trunk-pain*)).mp.
30. lumbar* adj3 (disc* adj3 (extru* or degenerat* or displac* or herniat* or prolaps* or sequestered or slipped or protru* or avuls*)).mp.
31. lumbar* adj3 (disk* adj3 (extru* or degenerat* or displac* or herniat* or prolaps* or sequestered or slipped or protru* or avuls*)).mp.
32. lumbar* adj3 (pain* or facet* or (nerve adj2 root*) or osteoarth* or radicul* or stenos* or spondylo* or zygapophys* or injur* or discomfort* or dysfunction* or sore* or herniat*).mp.
33. lumbo* adj3 (pain* or facet* or (nerve adj2 root*) or osteoarth* or radicul* or stenos* or spondylo* or zygapophys* or injur* or discomfort* or dysfunction* or sore* or herniat*).mp.
34. back adj3 (ach* or injur* or pain* or sprain* or strain* or disorder*).mp.
35. backach*.mp.
36. back-pain*.mp.
37. intervertebral* adj3 (disc* adj3 (extru* or degenerat* or displac* or herniat* or prolaps* or sequestered or slipped or protru* or avuls*)).mp.
38. intervertebral* adj3 (disk* adj3 (extru* or degenerat* or displac* or herniat* or prolaps* or sequestered or slipped or protru* or avuls*)).mp.
39. coccy* adj2 (ach* or injur* or pain* or sprain* or strain*).mp.
40. (coccygodyn* or coccalg* or coccygalg*).mp.
41. dorsalg*.mp.
42. lumbago*.mp.
43. lumboischialg*.mp.
44. (piriformis* adj2 syndrome*).mp.
45. sacral* adj3 (pain* or facet* or (nerve adj2 root*) or osteoarth* or radicul* or stenos* or spondylo* or zygapophys* or injur* or discomfort* or dysfunction* or sore* or herniat*).mp.
46. sacro* adj3 (pain* or facet* or (nerve adj2 root*) or osteoarth* or radicul* or stenos* or spondylo* or zygapophys* or injur* or discomfort* or dysfunction* or sore* or herniat*).mp.
47. "si" adj2 (joint* adj3 (pain* or facet* or (nerve adj2 root*) or osteoarth* or radicul* or stenos* or spondylo* or zygapophys* or injur* or discomfort* or dysfunction* or sore* or herniat*)).mp.
48. sacrococcy* adj2 (ach* or injur* or pain* or sprain* or strain*).mp.
49. sacrum* adj2 (ach* or injur* or pain* or sprain* or strain*).mp.
50. sciatic*.mp.
51. stenos* adj2 (spine* or spinal* or vertebral*).mp.
52. (spine* or spinal*) adj2 osteoarthr*.mp.
53. spine* adj3 (condition* or diseas* or disabilit* or disorder* or degenerat* or pain* or stenos*).mp.
54. spinal* adj3 (condition* or diseas* or disabilit* or disorder* or degenerat* or pain* or stenos*).mp.
55. spondylo*.mp.
56. tailbone* adj3 (ach* or injur* or pain* or sprain* or strain*).mp.
57. vertebr* adj3 (ach* or injur* or pain* or sprain* or strain*).mp
58. poly-radicul* or polyradicul*.mp.
59. neuropath* adj2 (lumbar* or lumbo* or sacral* or sacro* or (low* adj2 back) or low-back* or lower-back* or spine* or spinal* or L1 or L2 or L3 or L4 or L5).mp.
60. radiculopath* adj3 (lumbar* or lumbo* or sacral* or sacro* or (low* adj2 back) or low-back* or lower-back* or spine* or spinal* or L1 or L2 or L3 or L4 or L5).mp.
61. radiating* adj3 (lumbar* or lumbo* or sacral* or sacro* or (low* adj2 back) or low-back* or lower-back* or spine* or spinal* or L1 or L2 or L3 or L4 or L5).mp.
62. radicular* adj3 (lumbar* or lumbo* or sacral* or sacro* or (low* adj2 back) or low-back* or lower-back* or spine* or spinal* or L1 or L2 or L3 or L4 or L5).mp.
63. lumborum* adj3 (ach* or injur* or pain* or sprain* or strain*).mp.
64. longissimus* adj3 (ach* or injur* or pain* or sprain* or strain*).mp.
65. (erector adj2 spin*) adj3 (ach* or injur* or pain* or sprain* or strain*).mp.
66. synovial* adj2 cyst*.mp.
67. thoracolumbar* adj3 (pain* or facet* or (nerve* adj2 root*) or osteoarthr* or radicul* or stenos* or spondylo* or zygapohys* or injur* or trauma* or discomfort* or dysfunction* or sore* or herniat*).mp.
68. thoraco-lumbar* adj3 (pain* or facet* or (nerve* adj2 root*) or osteoarthr* or radicul* or stenos* or spondylo* or zygapohys* or injur* or trauma* or discomfort* or dysfunction* or sore* or herniat*).mp.
69. curvatur* adj2 (spine* or spinal*).mp.
70. (pathol* adj2 (lumbar* or (low* adj2 back) or low-back* or (lower* adj2 back) or lower-back* or thoracolumbar* or thoraco-lumbar* or intervertebral* or lumbosacral* or lumbo-sacral* or sacral* or sacro-iliac* or sacroiliac*)).mp.
71. or/1-70
72. Acupuncture/
73. Acupuncture Therapy/
74. Acupuncture Analgesia/
75. Acupuncture Points/
76. Acupuncture, Ear/
77. Acupressure/
78. Meridians/
79. Moxibustion/
80. Trigger Points/
81. Dry Needling/
82. Electroacupuncture/
83. Auriculotherapy/
84. acupunct*.mp.
85. electroacupunct*.mp.
86. electro-acupunct*.mp.
87. meridian*.mp.
88. moxibust*.mp.
89. dry adj2 needl*.mp.
90. trigger adj point*.mp.
91. pharmacopunct*.mp.
92. acupotomy*.mp.
93. acupotomies.mp
94. auriculotherap*.mp.
95. (needl* adj2 (subcutan* or body*)).mp.
96. acupress*.mp
97. (Ching Lo or Jing Luo or Jingluo).mp.
98. Korean Constitutional.mp.
99. French Energetic.mp.
100. artemisia vulgaris.mp.
101. Lemington Five Elements.mp.
102. (intramuscul* adj2 stimul*).mp.
103. (Shiatsu or Shiatzu or Zhi Ya or Chih Ya).mp.
104. or/ 73-104
105. exp Randomized Controlled Trial/
106. exp Randomized Controlled Trials as Topic/
107. Controlled Clinical Trial/
108. exp Controlled Clinical Trials as Topic/
109. exp Clinical Trials as Topic/
110. exp Clinical Trial/
111. Double-Blind Method/
112. Single-Blind Method/
113. Cross-Over Studies/
114. Placebos/
115. Placebo Effect/
116. Random Allocation/
117. random*.mp.
118. clinical* adj2 trial*mp.
119. controlled* adj2 (trial*).mp.
120. blind* adj2 (doubl* or singl*).mp.
121. placebo*.mp.
122. (crossover* or cross-over*).mp.
123. randomized controlled trial.pt.
124. controlled clinical trial.pt.
125. clinical trial.pt.
126. or/ 95-1115
127. 71 AND 94 AND 116
128. exp Animals/
129. exp Humans/
130. 118 NOT 119
131. Limit 117 NOT 120
132. (comment or clinical conference or congress or consensus development conference or editorial or letter or review or systematic review or guideline or practice guideline or case reports).pt.
133. Limit 121 NOT 122
134. Limit 123 to dt=20070701-20220301
135. Limit 123 to rd=20070701-20220309
136. 124 or 125

**B. Database & Platform:**  CINAHL (EBSCO)

**Years of search:** August 1, 2019 - March 9, 2022

**Date search run:**  March 9, 2022 **Number of records retrieved:**  113

**Search Strategy:**

1. MH Low Back Pain
2. MH Back Pain+
3. MH Back Injuries
4. MH Intervertebral Disc Displacement
5. MH Intervertebral Disc/IN
6. MH Lumbar Vertebrae/IN
7. MH Lumbosacral Plexus/IN
8. MH Coccyx/IN
9. MH Osteoarthritis, Spine
10. MH Osteoarthritis
11. MH Piriformis Muscles/IN
12. MH Polyradiculopathy/
13. MH Sacroiliac Joint/IN
14. MH Sciatica
15. MH Spinal Curvatures
16. MH Spinal Diseases
17. MH Spinal Injuries
18. MH Spinal Stenosis
19. MH Spondylolysis+
20. MH Spondylosis
21. MH Synovial Cyst
22. MH Zygaphophyseal Joint/IN
23. TI ((low* n2 (back n2 pain*)) or (low-back* n2 pain*) or (lower-back* n2 pain*) or (low* n2 back-pain*)) or AB ((low* n2 (back n2 pain*)) or (low-back* n2 pain*) or (lower-back* n2 pain*) or (low* n2 back-pain*))
24. TI ((low* n2 (back n2 injur*)) or (low-back* n2 injur*) or (lower-back* n2 injur*) or (low* n2 back-injur*)) or AB ((low* n2 (back n2 injur*)) or (low-back* n2 injur*) or (lower-back* n2 injur*) or (low* n2 back-injur*))
25. TI ((low* n2 (back n2 trauma*)) or (low-back n2 trauma*) or (lower-back* n2 trauma*) or (low* n2 back-trauma*)) or AB ((low* n2 (back n2 trauma*)) or (low-back n2 trauma*) or (lower-back* n2 trauma*) or (low* n2 back-trauma*))
26. TI ((low* n2 (trunk n2 pain*)) or (lower-trunk* n2 pain*) or (low* n2 trunk-pain*)) or AB ((low* n2 (trunk n2 pain*)) or (lower-trunk* n2 pain*) or (low* n2 trunk-pain*))
27. TI lumbar* n3 (disc* n3 (extru* or degenerat* or displac* or herniat* or prolaps* or sequestered or slipped or protru* or avuls*)) or AB lumbar* n3 (disc* n3 (extru* or degenerat* or displac* or herniat* or prolaps* or sequestered or slipped or protru* or avuls*))
28. TI lumbar* n3 (disk* n3 (extru* or degenerat* or displac* or herniat* or prolaps* or sequestered or slipped or protru* or avuls*)) or AB lumbar* n3 (disk* n3 (extru* or degenerat* or displac* or herniat* or prolaps* or sequestered or slipped or protru* or avuls*))
29. TI lumbar* n3 (pain* or facet* or (nerve n2 root*) or osteoarth* or radicul* or stenos* or spondylo* or zygapophys* or injur* or discomfort* or dysfunction* or sore* or herniat*) or AB lumbar* n3 (pain* or facet* or (nerve n2 root*) or osteoarth* or radicul* or stenos* or spondylo* or zygapophys* or injur* or discomfort* or dysfunction* or sore* or herniat*)
30. TI lumbo* n3 (pain* or facet* or (nerve n2 root*) or osteoarth* or radicul* or stenos* or spondylo* or zygapophys* or injur* or discomfort* or dysfunction* or sore* or herniat*) or AB lumbo* n3 (pain* or facet* or (nerve n2 root*) or osteoarth* or radicul* or stenos* or spondylo* or zygapophys* or injur* or discomfort* or dysfunction* or sore* or herniat*)
31. TI back n3 (ach* or injur* or pain* or sprain* or strain* or disorder*) or AB back n3 (ach* or injur* or pain* or sprain* or strain* or disorder
32. TI backach* or AB backach*
33. TI back-pain* or AB back-pain*
34. TI intervertebral* n3 (disc* n3 (extru* or degenerat* or displac* or herniat* or prolaps* or sequestered or slipped or protru* or avuls*)) or AB intervertebral* n3 (disc* n3 (extru* or degenerat* or displac* or herniat* or prolaps* or sequestered or slipped or protru* or avuls*))
35. TI intervertebral* n3 (disk* n3 (extru* or degenerat* or displac* or herniat* or prolaps* or sequestered or slipped or protru* or avuls*)) or AB intervertebral* n3 (disk* n3 (extru* or degenerat* or displac* or herniat* or prolaps* or sequestered or slipped or protru* or avuls*))
36. TI coccy* n2 (ach* or injur* or pain* or sprain* or strain*) or AB coccy* n2 (ach* or injur* or pain* or sprain* or strain*)
37. TI (coccygodyn* or coccalg* or coccygalg*) or AB (coccygodyn* or coccalg* or coccygalg*)
38. TI dorsalg* or AB dorsalg*
39. TI lumbago* or AB lumbago*
40. TI lumboischialg* or AB lumboischialg*
41. TI (piriformis* n2 syndrome*) or AB (piriformis* n2 syndrome*)
42. TI sacral* n3 (pain* or facet* or (nerve n2 root*) or osteoarth* or radicul* or stenos* or spondylo* or zygapophys* or injur* or discomfort* or dysfunction* or sore* or herniat*) or AB sacral* n3 (pain* or facet* or (nerve n2 root*) or osteoarth* or radicul* or stenos* or spondylo* or zygapophys* or injur* or discomfort* or dysfunction* or sore* or herniat*)
43. TI sacro* n3 (pain* or facet* or (nerve n2 root*) or osteoarth* or radicul* or stenos* or spondylo* or zygapophys* or injur* or discomfort* or dysfunction* or sore* or herniat*) or AB sacro* n3 (pain* or facet* or (nerve n2 root*) or osteoarth* or radicul* or stenos* or spondylo* or zygapophys* or injur* or discomfort* or dysfunction* or sore* or herniat*)
44. TI "si" n2 (joint* n3 (pain* or facet* or (nerve n2 root*) or osteoarth* or radicul* or stenos* or spondylo* or zygapophys* or injur* or discomfort* or dysfunction* or sore* or herniat*)) or AB "si" n2 (joint* n3 (pain* or facet* or (nerve n2 root*) or osteoarth* or radicul* or stenos* or spondylo* or zygapophys* or injur* or discomfort* or dysfunction* or sore* or herniat*))
45. TI sacrococcy* n2 (ach* or injur* or pain* or sprain* or strain*) or AB sacrococcy* n2 (ach* or injur* or pain* or sprain* or strain*)
46. TI sacrum* n2 (ach* or injur* or pain* or sprain* or strain*) or AB sacrum* n2 (ach* or injur* or pain* or sprain* or strain*)
47. TI sciatic* or sciatic*
48. TI stenos* n2 (spine* or spinal* or vertebral*) or AB stenos* n2 (spine* or spinal* or vertebral*)
49. TI (spine* or spinal*) n2 osteoarthr* or AB (spine* or spinal*) n2 osteoarthr*
50. TI spine* n3 (condition* or diseas* or disabilit* or disorder* or degenerat* or pain* or stenos*) or AB spine* n3 (condition* or diseas* or disabilit* or disorder* or degenerat* or pain* or stenos*)
51. TI spinal* n3 (condition* or diseas* or disabilit* or disorder* or degenerat* or pain* or stenos*) or AB spinal* n3 (condition* or diseas* or disabilit* or disorder* or degenerat* or pain* or stenos*)
52. TI spondylo* or AB spondylo*
53. TI tailbone* n3 (ach* or injur* or pain* or sprain* or strain*) or AB tailbone* n3 (ach* or injur* or pain* or sprain* or strain*)
54. TI vertebr* n3 (ach* or injur* or pain* or sprain* or strain*) or AB vertebr* n3 (ach* or injur* or pain* or sprain* or strain*)
55. TI (poly-radicul* or polyradicul*) or AB (poly-radicul* or polyradicul*)
56. TI neuropath* n2 (lumbar* or lumbo* or sacral* or sacro* or (low* n2 back) or low-back* or lower-back* or spine* or spinal* or L1 or L2 or L3 or L4 or L5) or AB neuropath* n2 (lumbar* or lumbo* or sacral* or sacro* or (low* n2 back) or low-back* or lower-back* or spine* or spinal* or L1 or L2 or L3 or L4 or L5)
57. TI radiculopath* n3 (lumbar* or lumbo* or sacral* or sacro* or (low* n2 back) or low-back* or lower-back* or spine* or spinal* or L1 or L2 or L3 or L4 or L5) or AB radiculopath* n3 (lumbar* or lumbo* or sacral* or sacro* or (low* n2 back) or low-back* or lower-back* or spine* or spinal* or L1 or L2 or L3 or L4 or L5)
58. TI radiating* n3 (lumbar* or lumbo* or sacral* or sacro* or (low* n2 back) or low-back* or lower-back* or spine* or spinal* or L1 or L2 or L3 or L4 or L5) or AB radiating* n3 (lumbar* or lumbo* or sacral* or sacro* or (low* n2 back) or low-back* or lower-back* or spine* or spinal* or L1 or L2 or L3 or L4 or L5)
59. TI radicular* n3 (lumbar* or lumbo* or sacral* or sacro* or (low* n2 back) or low-back* or lower-back* or spine* or spinal* or L1 or L2 or L3 or L4 or L5) or AB radicular* n3 (lumbar* or lumbo* or sacral* or sacro* or (low* n2 back) or low-back* or lower-back* or spine* or spinal* or L1 or L2 or L3 or L4 or L5)
60. TI lumborum* n3 (ach* or injur* or pain* or sprain* or strain*) or AB lumborum* n3 (ach* or injur* or pain* or sprain* or strain*)
61. TI longissimus* n3 (ach* or injur* or pain* or sprain* or strain*) or AB longissimus* n3 (ach* or injur* or pain* or sprain* or strain*)
62. TI (erector n2 spin*) n3 (ach* or injur* or pain* or sprain* or strain*) or AB erector n2 spin*) n3 (ach* or injur* or pain* or sprain* or strain*)
63. TI synovial* n2 cyst* or AB synovial* n2 cyst*
64. TI thoracolumbar* n3 (pain* or facet* or (nerve* n2 root*) or osteoarthr* or radicul* or stenos* or spondylo* or zygapohys* or injur* or trauma* or discomfort* or dysfunction* or sore* or herniat*) or AB thoracolumbar* n3 (pain* or facet* or (nerve* n2 root*) or osteoarthr* or radicul* or stenos* or spondylo* or zygapohys* or injur* or trauma* or discomfort* or dysfunction* or sore* or herniat*)
65. TI thoraco-lumbar* n3 (pain* or facet* or (nerve* n2 root*) or osteoarthr* or radicul* or stenos* or spondylo* or zygapohys* or injur* or trauma* or discomfort* or dysfunction* or sore* or herniat*) or AB thoraco-lumbar* n3 (pain* or facet* or (nerve* n2 root*) or osteoarthr* or radicul* or stenos* or spondylo* or zygapohys* or injur* or trauma* or discomfort* or dysfunction* or sore* or herniat*)
66. TI curvatur* n2 (spine* or spinal*) or AB curvatur* n2 (spine* or spinal*)
67. TI (pathol* n2 (lumbar* or (low* n2 back) or low-back* or (lower* n2 back) or lower-back* or thoracolumbar* or thoraco-lumbar* or intervertebral* or lumbosacral* or lumbo-sacral* or sacral* or sacro-iliac* or sacroiliac*)) or AB (pathol* n2 (lumbar* or (low* n2 back) or low-back* or (lower* n2 back) or lower-back* or thoracolumbar* or thoraco-lumbar* or intervertebral* or lumbosacral* or lumbo-sacral* or sacral* or sacro-iliac* or sacroiliac*))
68. or/1-67
69. MH Acupuncture+
70. MH Acupuncture Analgesia
71. MH Acupuncture Points
72. MH Acupuncture, Ear
73. MH Meridians+
74. MH Moxibustion
75. MH Trigger Point
76. MH Dry Needling
77. MH Electroacupuncture
78. MH Auriculotherapy
79. TI acupunct* or AB acupunct*
80. TI electroacupunct* or AB electroacupunct*
81. TI electro-acupunct* or AB electro-acupunct*
82. TI meridian* or AB meridian*
83. TI moxibust* or AB moxibust*
84. TI dry n2 needl* or AB dry n2 needl*
85. TI trigger n2 point* or AB trigger n2 point*
86. TI tender n2 point* or AB tender n2 point*
87. TI pharmacopunct* or AB pharmacopunct*
88. TI neuroreflexotherap* or AB neuroreflexotherap*
89. TI acupotomy* or AB acupotomy
90. TI acupotomies or AB acupotomies
91. TI auriculotherap* or AB auriculotherap*
92. TI (needl* n2 (subcutan* or body*)) or AB (needl* n2 (subcutan* or body*))
93. TI (Ching Lo or Jing Luo or Jingluo) or AB (Ching Lo or Jing Luo or Jingluo)
94. TI Korean Constitutional or AB Korean Constitutional
95. TI French Energetic or AB French Energetic
96. TI artemisia vulgaris or AB artemisia vulgaris
97. TI Lemington Five Elements or AB Lemington Five Elements
98. TI (intramuscul* n2 stimul*) or AB (intramuscul* n2 stimul*)
99. TI (Shiatsu or Shiatzu or Zhi Ya or Chih Ya) or AB (Shiatsu or Shiatzu or Zhi Ya or Chih Ya)
100. or/ 69-99
101. MH Randomized Controlled Trials+
102. MH Clinical Trials+
103. MH Double-Blind Studies
104. MH Single-Blind Studies
105. MH Crossover Design
106. MH Placebos
107. MH Placebo Effect
108. MH Random Assignment
109. TI random* or AB random*
110. TI clinical* n2 (trial* or study* or studies*) or AB clinical* n2 (trial* or study* or studies*)
111. TI controlled* n2 (trial* or study* or studies*) or AB controlled* n2 (trial* or study* or studies*)
112. TI blind* n2 (doubl* or singl*) or AB blind* n2 (doubl* or singl*)
113. TI placebo* or AB placebo*
114. TI (crossover* or cross-over*) or AB (crossover* or cross-over*)
115. PT randomized controlled trial
116. PT controlled clinical trial
117. PT clinical trial
118. or/ 101-117
119. 68 AND 100 AND 118
120. MH Animals+
121. MH Human
122. 120 NOT 121
123. Limit 119 NOT 122
124. 123 NOT PT (abstract or brief item or book review or case study or commentary or doctoral dissertation or editorial or letter or practice guidelines or proceedings or review or systematic review)
125. 124 AND EM=20190801-20220309
126. 124 AND RD=20190801-20220309
127. 125 OR 126

**C.** **Database & Platform:**  EMBASE (Ovid)

**Years of search:** August 1, 2019 - March 9, 2022

**Date search run:**  March 9, 2022 **Number of records retrieved:** 323

**Search Strategy:**

1. Low Back Pain/
2. exp Backache/
3. Intervertebral Disc Degeneration/
4. Intervertebral Disk Hernia/
5. Lumbar Disk Hernia/
6. Coccyx/
7. Spondylosis/
8. Spondylolysis/
9. Osteoarthritis/
10. Lumbar Spinal Stenosis/
11. Synovial Cyst/
12. Piriformis Syndrome/
13. Sciatica/
14. ((low* adj2 (back adj2 pain*)) or (low-back* adj2 pain*) or (lower-back* adj2 pain*) or (low* adj2 back-pain*)).mp.
15. ((low* adj2 (back adj2 injur*)) or (low-back* adj2 injur*) or (lower-back* adj2 injur*) or (low* adj2 back-injur*)).mp.
16. ((low* adj2 (back adj2 trauma*)) or (low-back adj2 trauma*) or (lower-back* adj2 trauma*) or (low* adj2 back-trauma*)).mp.
17. ((low* adj2 (trunk adj2 pain*)) or (lower-trunk* adj2 pain*) or (low* adj2 trunk-pain*)).mp.
18. lumbar* adj3 (disc* adj3 (extru* or degenerat* or displac* or herniat* or prolaps* or sequestered or slipped or protru* or avuls*)).mp.
19. lumbar* adj3 (disk* adj3 (extru* or degenerat* or displac* or herniat* or prolaps* or sequestered or slipped or protru* or avuls*)).mp.
20. lumbar* adj3 (pain* or facet* or (nerve adj2 root*) or osteoarth* or radicul* or stenos* or spondylo* or zygapophys* or injur* or discomfort* or dysfunction* or sore* or herniat*).mp.
21. lumbo* adj3 (pain* or facet* or (nerve adj2 root*) or osteoarth* or radicul* or stenos* or spondylo* or zygapophys* or injur* or discomfort* or dysfunction* or sore* or herniat*).mp.
22. back adj3 (ach* or injur* or pain* or sprain* or strain* or disorder*).mp.
23. backach*.mp.
24. back-pain*.mp.
25. intervertebral* adj3 (disc* adj3 (extru* or degenerat* or displac* or herniat* or prolaps* or sequestered or slipped or protru* or avuls*)).mp.
26. intervertebral* adj3 (disk* adj3 (extru* or degenerat* or displac* or herniat* or prolaps* or sequestered or slipped or protru* or avuls*)).mp.
27. coccy* adj2 (ach* or injur* or pain* or sprain* or strain*).mp.
28. (coccygodyn* or coccalg* or coccygalg*).mp.
29. dorsalg*.mp.
30. lumbago*.mp.
31. lumboischialg*.mp.
32. (piriformis* adj2 syndrome*).mp.
33. sacral* adj3 (pain* or facet* or (nerve adj2 root*) or osteoarth* or radicul* or stenos* or spondylo* or zygapophys* or injur* or discomfort* or dysfunction* or sore* or herniat*).mp.
34. sacro* adj3 (pain* or facet* or (nerve adj2 root*) or osteoarth* or radicul* or stenos* or spondylo* or zygapophys* or injur* or discomfort* or dysfunction* or sore* or herniat*).mp.
35. "si" adj2 (joint* adj3 (pain* or facet* or (nerve adj2 root*) or osteoarth* or radicul* or stenos* or spondylo* or zygapophys* or injur* or discomfort* or dysfunction* or sore* or herniat*)).mp.
36. sacrococcy* adj2 (ach* or injur* or pain* or sprain* or strain*).mp.
37. sacrum* adj2 (ach* or injur* or pain* or sprain* or strain*).mp.
38. sciatic*.mp.
39. stenos* adj2 (spine* or spinal* or vertebral*).mp.
40. (spine* or spinal*) adj2 osteoarthr*.mp.
41. spine* adj3 (condition* or diseas* or disabilit* or disorder* or degenerat* or pain* or stenos*).mp.
42. spinal* adj3 (condition* or diseas* or disabilit* or disorder* or degenerat* or pain* or stenos*).mp.
43. spondylo*.mp.
44. tailbone* adj3 (ach* or injur* or pain* or sprain* or strain*).mp.
45. vertebr* adj3 (ach* or injur* or pain* or sprain* or strain*).mp
46. poly-radicul* or polyradicul*.mp.
47. neuropath* adj2 (lumbar* or lumbo* or sacral* or sacro* or (low* adj2 back) or low-back* or lower-back* or spine* or spinal* or L1 or L2 or L3 or L4 or L5).mp.
48. radiculopath* adj3 (lumbar* or lumbo* or sacral* or sacro* or (low* adj2 back) or low-back* or lower-back* or spine* or spinal* or L1 or L2 or L3 or L4 or L5).mp.
49. radiating* adj3 (lumbar* or lumbo* or sacral* or sacro* or (low* adj2 back) or low-back* or lower-back* or spine* or spinal* or L1 or L2 or L3 or L4 or L5).mp.
50. radicular* adj3 (lumbar* or lumbo* or sacral* or sacro* or (low* adj2 back) or low-back* or lower-back* or spine* or spinal* or L1 or L2 or L3 or L4 or L5).mp.
51. lumborum* adj3 (ach* or injur* or pain* or sprain* or strain*).mp.
52. longissimus* adj3 (ach* or injur* or pain* or sprain* or strain*).mp.
53. (erector adj2 spin*) adj3 (ach* or injur* or pain* or sprain* or strain*).mp.
54. synovial* adj2 cyst*.mp.
55. thoracolumbar* adj3 (pain* or facet* or (nerve* adj2 root*) or osteoarthr* or radicul* or stenos* or spondylo* or zygapohys* or injur* or trauma* or discomfort* or dysfunction* or sore* or herniat*).mp.
56. thoraco-lumbar* adj3 (pain* or facet* or (nerve* adj2 root*) or osteoarthr* or radicul* or stenos* or spondylo* or zygapohys* or injur* or trauma* or discomfort* or dysfunction* or sore* or herniat*).mp.
57. curvatur* adj2 (spine* or spinal*).mp.
58. (pathol* adj2 (lumbar* or (low* adj2 back) or low-back* or (lower* adj2 back) or lower-back* or thoracolumbar* or thoraco-lumbar* or intervertebral* or lumbosacral* or lumbo-sacral* or sacral* or sacro-iliac* or sacroiliac*)).mp.
59. or/1-58
60. exp Acupuncture/
61. Acupuncture Analgesia/
62. exp Acupuncture Point/
63. Moxibustion/
64. Trigger Point/
65. Dry Needling/
66. Electroacupuncture/
67. Auricular Acupuncture/
68. acupunct*.mp.
69. electroacupunct*.mp.
70. electro-acupunct*.mp.
71. meridian*.mp.
72. moxibust*.mp.
73. dry adj2 needl*.mp.
74. trigger adj point*.mp.
75. tender adj point*.mp.
76. neuroreflexotherap*.mp.
77. pharmacopunct*.mp.
78. acupotomy*.mp.
79. acupotomies.mp
80. auriculotherap*.mp.
81. (needl* adj2 (subcutan* or body*)).mp.
82. (Ching Lo or Jing Luo or Jingluo).mp.
83. Korean Constitutional.mp.
84. French Energetic.mp.
85. artemisia vulgaris.mp.
86. Lemington Five Elements.mp.
87. (intramuscul* adj2 stimul*).mp.
88. (Shiatsu or Shiatzu or Zhi Ya or Chih Ya).mp.
89. or/ 60-88
90. exp Randomized Controlled Trial/
91. exp Randomized Controlled Trial (Topic)/
92. Controlled Clinical Trial/
93. exp Controlled Clinical Trial (Topic)/
94. exp Clinical Trial (Topic)/
95. exp Clinical Trial/
96. Double-Blind Procedure/
97. Single-Blind Procedure/
98. Crossover Procedure/
99. Placebo/
100. Placebo Effect/
101. Randomization/
102. random*.mp.
103. clinical* adj2 trial*mp.
104. controlled* adj2 (trial*).mp.
105. blind* adj2 (doubl* or singl*).mp.
106. placebo*.mp.
107. (crossover* or cross-over*).mp.
108. or/ 90-107
109. 59 AND 89 AND 108
110. exp Animal/
111. exp Human/
112. 110 NOT 111
113. Limit 109 NOT 112
114. (books or chapter or conference abstract or conference paper or conference review or review or editorial or letter).pt.
115. Limit 113 NOT 114
116. Limit 115 to dd=20190801-20220309
117. Limit 115 to rd=20190801-20220309
118. 116 or 117

**D. Database & Platform:**  Cochrane Central Register of Controlled Trials (Wiley)

**Years of search:** August 2019 – March 2022

**Date search run:**  March 9, 2022 **Number of records retrieved:** 392

**Search Strategy:**

#1 MeSH descriptor: [Back Injuries] explode all trees

#2 MeSH descriptor: [Back Pain] explode all trees

#3 MeSH descriptor: [Low Back Pain] this term only

#4 MeSH descriptor: [Osteoarthritis] this term only

#5 MeSH descriptor: [Osteoarthritis, Spine] this term only

#6 MeSH descriptor: [Piriformis Muscle Syndrome] this term only

#7 MeSH descriptor: [Polyradiculopathy] this term only

#8 MeSH descriptor: [Spinal Diseases] explode all trees

#9 MeSH descriptor: [Synovial Cyst] this term only

#10 MeSH descriptor: [Back Muscles] this term only and with qualifier(s): [injuries - IN]

#11 MeSH descriptor: [Intervertebral Disc Degeneration] this term only

#12 MeSH descriptor: [Intervertebral Disc Displacement] this term only

#13 MeSH descriptor: [Intervertebral Disc] this term only and with qualifier(s): [injuries - IN]

#14 MeSH descriptor: [Lumbar Vertebrae] this term only and with qualifier(s): [injuries - IN]

#15 MeSH descriptor: [Lumbosacral Plexus] this term only and with qualifier(s): [injuries - IN]

#16 MeSH descriptor: [Sacroiliac Joint] this term only and with qualifier(s): [injuries - IN]

#17 MeSH descriptor: [Sacrum] this term only and with qualifier(s): [injuries - IN]

#18 MeSH descriptor: [Lumbosacral Region] this term only and with qualifier(s): [injuries - IN]

#19 MeSH descriptor: [Zygapophyseal Joint] this term only and with qualifier(s): [injuries - IN]

#20 MeSH descriptor: [Sciatica] this term only

#21 MeSH descriptor: [Spinal Injuries] this term only

#22 MeSH descriptor: [Spinal Stenosis] this term only

#23 MeSH descriptor: [Spondylolysis] explode all trees

#24 (((low* near/2 (back near/2 pain*)) or (low-back* near/2 pain*) or (lower-back* near/2 pain*) or (low* near/2 back-pain*))):ti OR (((low* near/2 (back near/2 pain*)) or (low-back* near/2 pain*) or (lower-back* near/2 pain*) or (low* near/2 back-pain*))):ab

#25 (((low* near/2 (back near/2 injur*)) or (low-back* near/2 injur*) or (lower-back* near/2 injur*) or (low* near/2 back-injur*))):ti OR (((low* near/2 (back near/2 injur*)) or (low-back* near/2 injur*) or (lower-back* near/2 injur*) or (low* near/2 back-injur*))):ab

#26 (((low* near/2 (back near/2 trauma*)) or (low-back near/2 trauma*) or (lower-back* near/2 trauma*) or (low* near/2 back-trauma*))):ti OR (((low* near/2 (back near/2 trauma*)) or (low-back near/2 trauma*) or (lower-back* near/2 trauma*) or (low* near/2 back-trauma*))):ab

#27 (((low* near/2 (trunk near/2 pain*)) or (lower-trunk* near/2 pain*) or (low* near/2 trunk-pain*))):ti OR (((low* near/2 (trunk near/2 pain*)) or (lower-trunk* near/2 pain*) or (low* near/2 trunk-pain*))):ab

#28 (lumbar* near/3 (disc* near/3 (extru* or degenerat* or displac* or herniat* or prolaps* or sequestered or slipped or protru* or avuls*))):ti OR (lumbar* near/3 (disc* near/3 (extru* or degenerat* or displac* or herniat* or prolaps* or sequestered or slipped or protru* or avuls*))):ab

#29 (lumbar* near/3 (disk* near/3 (extru* or degenerat* or displac* or herniat* or prolaps* or sequestered or slipped or protru* or avuls*))):ti OR (lumbar* near/3 (disk* near/3 (extru* or degenerat* or displac* or herniat* or prolaps* or sequestered or slipped or protru* or avuls*))):ab

#30 (lumbar* near/3 (pain* or facet* or (nerve near/2 root*) or osteoarth* or radicul* or stenos* or spondylo* or zygapophys* or injur* or discomfort* or dysfunction* or sore* or herniat*)):ti OR (lumbar* near/3 (pain* or facet* or (nerve near/2 root*) or osteoarth* or radicul* or stenos* or spondylo* or zygapophys* or injur* or discomfort* or dysfunction* or sore* or herniat*)):ab

#31 (lumbo* near/3 (pain* or facet* or (nerve near/2 root*) or osteoarth* or radicul* or stenos* or spondylo* or zygapophys* or injur* or discomfort* or dysfunction* or sore* or herniat*)):ti OR (lumbo* near/3 (pain* or facet* or (nerve near/2 root*) or osteoarth* or radicul* or stenos* or spondylo* or zygapophys* or injur* or discomfort* or dysfunction* or sore* or herniat*)):ab

#32 (back near/3 (ach* or injur* or pain* or sprain* or strain* or disorder*)):ti OR (back near/3 (ach* or injur* or pain* or sprain* or strain* or disorder*)):ab

#33 (backach*):ti OR (backach*):ab

#34 (back-pain*):ti OR (back-pain*):ab

#35 (intervertebral* near/3 (disc* near/3 (extru* or degenerat* or displac* or herniat* or prolaps* or sequestered or slipped or protru* or avuls*))):ti OR (intervertebral* near/3 (disc* near/3 (extru* or degenerat* or displac* or herniat* or prolaps* or sequestered or slipped or protru* or avuls*))):ab

#36 (intervertebral* near/3 (disk* near/3 (extru* or degenerat* or displac* or herniat* or prolaps* or sequestered or slipped or protru* or avuls*))):ti OR (intervertebral* near/3 (disk* near/3 (extru* or degenerat* or displac* or herniat* or prolaps* or sequestered or slipped or protru* or avuls*))):ab

#37 (coccy* near/2 (ach* or injur* or pain* or sprain* or strain*)):ti OR (coccy* near/2 (ach* or injur* or pain* or sprain* or strain*)):ab

#38 ((coccygodyn* or coccalg* or coccygalg*)):ti OR ((coccygodyn* or coccalg* or coccygalg*)):ab

#39 (dorsalg* or lumbago* or lumboischialg*):ti OR (dorsalg* or lumbago* or lumboischialg*):ab

#40 ((piriformis* near/2 syndrome*)):ti OR ((piriformis* near/2 syndrome*)):ab

#41 (sacral* near/3 (pain* or facet* or (nerve near/2 root*) or osteoarth* or radicul* or stenos* or spondylo* or zygapophys* or injur* or discomfort* or dysfunction* or sore* or herniat*)):ti OR (sacral* near/3 (pain* or facet* or (nerve near/2 root*) or osteoarth* or radicul* or stenos* or spondylo* or zygapophys* or injur* or discomfort* or dysfunction* or sore* or herniat*)):ab

#42 (sacro* near/3 (pain* or facet* or (nerve near/2 root*) or osteoarth* or radicul* or stenos* or spondylo* or zygapophys* or injur* or discomfort* or dysfunction* or sore* or herniat*)):ti OR (sacro* near/3 (pain* or facet* or (nerve near/2 root*) or osteoarth* or radicul* or stenos* or spondylo* or zygapophys* or injur* or discomfort* or dysfunction* or sore* or herniat*)):ab

#43 ("si" near/2 (joint* near/3 (pain* or facet* or (nerve near/2 root*) or osteoarth* or radicul* or stenos* or spondylo* or zygapophys* or injur* or discomfort* or dysfunction* or sore* or herniat*))):ti OR ("si" near/2 (joint* near/3 (pain* or facet* or (nerve near/2 root*) or osteoarth* or radicul* or stenos* or spondylo* or zygapophys* or injur* or discomfort* or dysfunction* or sore* or herniat*))):ab

#44 (sacrococcy* near/2 (ach* or injur* or pain* or sprain* or strain*)):ti OR (sacrococcy* near/2 (ach* or injur* or pain* or sprain* or strain*)):ab

#45 (sacrum* near/2 (ach* or injur* or pain* or sprain* or strain*)):ti OR (sacrum* near/2 (ach* or injur* or pain* or sprain* or strain*)):ab

#46 (sciatic*):ti OR (sciatic*):ab ˙

#47 (stenos* near/2 (spine* or spinal* or vertebral*)):ti OR (stenos* near/2 (spine* or spinal* or vertebral*)):ab

#48 ((spine* or spinal*) near/2 osteoarthr*):ti OR ((spine* or spinal*) near/2 osteoarthr*):ab

#49 (spine* near/3 (condition* or diseas* or disabilit* or disorder* or degenerat* or pain* or stenos*)):ti OR (spine* near/3 (condition* or diseas* or disabilit* or disorder* or degenerat* or pain* or stenos*)):ab

#50 (spinal* near/3 (condition* or diseas* or disabilit* or disorder* or degenerat* or pain* or stenos*)):ti OR (spinal* near/3 (condition* or diseas* or disabilit* or disorder* or degenerat* or pain* or stenos*)):ab

#51 (spondylo*):ti OR (spondylo*):ab

#52 (tailbone* near/3 (ach* or injur* or pain* or sprain* or strain*)):ti OR (tailbone* near/3 (ach* or injur* or pain* or sprain* or strain*)):ab

#53 (vertebr* near/3 (ach* or injur* or pain* or sprain* or strain*)):ti OR (vertebr* near/3 (ach* or injur* or pain* or sprain* or strain*)):ab

#54 (poly-radicul* or polyradicul*):ti OR (poly-radicul* or polyradicul*):ab

#55 (neuropath* near/2 (lumbar* or lumbo* or sacral* or sacro* or (low* near/2 back) or low-back* or lower-back* or spine* or spinal* or L1 or L2 or L3 or L4 or L5)):ti OR (neuropath* near/2 (lumbar* or lumbo* or sacral* or sacro* or (low* near/2 back) or low-back* or lower-back* or spine* or spinal* or L1 or L2 or L3 or L4 or L5)):ab

#56 (radiculopath* near/3 (lumbar* or lumbo* or sacral* or sacro* or (low* near/2 back) or low-back* or lower-back* or spine* or spinal* or L1 or L2 or L3 or L4 or L5)):ti OR (radiculopath* near/3 (lumbar* or lumbo* or sacral* or sacro* or (low* near/2 back) or low-back* or lower-back* or spine* or spinal* or L1 or L2 or L3 or L4 or L5)):ab

#57 (radiating* near/3 (lumbar* or lumbo* or sacral* or sacro* or (low* near/2 back) or low-back* or lower-back* or spine* or spinal* or L1 or L2 or L3 or L4 or L5)):ti OR (radiating* near/3 (lumbar* or lumbo* or sacral* or sacro* or (low* near/2 back) or low-back* or lower-back* or spine* or spinal* or L1 or L2 or L3 or L4 or L5)):ab

#58 (radicular* near/3 (lumbar* or lumbo* or sacral* or sacro* or (low* near/2 back) or low-back* or lower-back* or spine* or spinal* or L1 or L2 or L3 or L4 or L5)):ti OR (radicular* near/3 (lumbar* or lumbo* or sacral* or sacro* or (low* near/2 back) or low-back* or lower-back* or spine* or spinal* or L1 or L2 or L3 or L4 or L5)):ab

#59 (lumborum* near/3 (ach* or injur* or pain* or sprain* or strain*)):ti OR (lumborum* near/3 (ach* or injur* or pain* or sprain* or strain*)):ab

#60 (longissimus* near/3 (ach* or injur* or pain* or sprain* or strain*)):ti OR (longissimus* near/3 (ach* or injur* or pain* or sprain* or strain*)):ab

#61 ((erector near/2 spin*) near/3 (ach* or injur* or pain* or sprain* or strain*)):ti OR ((erector near/2 spin*) near/3 (ach* or injur* or pain* or sprain* or strain*)):ab

#62 (synovial* near/2 cyst*):ti OR (synovial* near/2 cyst*):ab

#63 (thoracolumbar* near/3 (pain* or facet* or (nerve* near/2 root*) or osteoarthr* or radicul* or stenos* or spondylo* or zygapohys* or injur* or trauma* or discomfort* or dysfunction* or sore* or herniat*)):ti OR (thoracolumbar* near/3 (pain* or facet* or (nerve* near/2 root*) or osteoarthr* or radicul* or stenos* or spondylo* or zygapohys* or injur* or trauma* or discomfort* or dysfunction* or sore* or herniat*)):ab

#64 (thoraco-lumbar* near/3 (pain* or facet* or (nerve* near/2 root*) or osteoarthr* or radicul* or stenos* or spondylo* or zygapohys* or injur* or trauma* or discomfort* or dysfunction* or sore* or herniat*)):ti OR (thoraco-lumbar* near/3 (pain* or facet* or (nerve* near/2 root*) or osteoarthr* or radicul* or stenos* or spondylo* or zygapohys* or injur* or trauma* or discomfort* or dysfunction* or sore* or herniat*)):ab

#65 (curvatur* near/2 (spine* or spinal*)):ti OR (curvatur* near/2 (spine* or spinal*)):ab

#66 ((pathol* near/2 (lumbar* or (low* near/2 back) or low-back* or (lower* near/2 back) or lower-back* or thoracolumbar* or thoraco-lumbar* or intervertebral* or lumbosacral* or lumbo-sacral* or sacral* or sacro-iliac* or sacroiliac*))):ti OR ((pathol* near/2 (lumbar* or (low* near/2 back) or low-back* or (lower* near/2 back) or lower-back* or thoracolumbar* or thoraco-lumbar* or intervertebral* or lumbosacral* or lumbo-sacral* or sacral* or sacro-iliac* or sacroiliac*))):ab

#67 MeSH descriptor: [Acupuncture] this term only

#68 MeSH descriptor: [Acupuncture Therapy] this term only

#69 MeSH descriptor: [Acupuncture Analgesia] this term only

#70 MeSH descriptor: [Acupuncture Points] this term only

#71 MeSH descriptor: [Acupuncture, Ear] this term only

#72 MeSH descriptor: [Meridians] this term only

#73 MeSH descriptor: [Moxibustion] this term only

#74 MeSH descriptor: [Trigger Points] this term only

#75 MeSH descriptor: [Dry Needling] this term only

#76 MeSH descriptor: [Electroacupuncture] this term only

#77 MeSH descriptor: [Auriculotherapy] this term only

#78 (acupunct*):ti OR (acupunct*):ab

#79 (electroacupunct*):ti OR (electroacupunct*):ab

#80 (electro-acupunct*):ti OR (electro-acupunct*):ab

#81 (meridian*):ti OR (meridian*):ab

#82 (moxibust*):ti OR (moxibust*):ab

#83 (dry near/2 needl*):ti OR (dry near/2 needl*):ab

#84 (trigger near/2 point*):ti OR (trigger near/2 point*):ab

#85 (tender near/2 point*):ti OR (tender near/2 point*):ab

#86 (neuroreflexotherap*):ti OR (neuroreflexotherap*):ab

#87 (pharmacopunct*):ti OR (pharmacopunct*):ab

#88 (acupotomy*):ti OR (acupotomy*):ab

#89 (acupotomies):ti OR (acupotomies):ab

#90 (auriculotherap*):ti OR (auriculotherap*):ab

#91 ((needl* near/2 (subcutan* or body*))):ti OR ((needl* near/2 (subcutan* or body*))):ab

#92 ((Ching Lo or Jing Luo or Jingluo)):ti OR ((Ching Lo or Jing Luo or Jingluo)):ab

#93 (Korean Constitutional):ti OR (Korean Constitutional):ab

#94 (French Energetic):ti OR (French Energetic):ab

#95 (artemisia vulgaris):ti OR (artemisia vulgaris):ab

#96 (Lemington Five Elements):ti OR (Lemington Five Elements):ab

#97 ((intramuscul* near/2 stimul*)):ti OR ((intramuscul* near/2 stimul*)):ab

#98 ((Shiatsu or Shiatzu or Zhi Ya or Chih Ya)):ti OR ((Shiatsu or Shiatzu or Zhi Ya or Chih Ya)):ab

#99 #1 or #2 or #3 or #4 or #5 or #6 or #7 or #8 or #9 or #10 or #11 or #12 or #13 or #14 or #15 or #16 or #17 or #18 or #19 or #20 or #21 or #22 or #23 or #24 or #25 or #26 or #27 or #28 or #29 or #30 or #31 or #32 or #33 or #34 or #35 or #36 or #37 or #38 or #39 or #40 or #41 or #42 or #43 or #44 or #45 or #46 or #47 or #48 or #49 or #50 or #51 or #52 or #53 or #54 or #55 or #56 or #57 or #58 or #59 or #60 or #61 or #62 or #63 or #64 or #65 or #66

#100 #67 or #68 or #69 or #70 or #71 or #72 or #73 or #74 or #75 or #76 or #77 or #78 or #79 or #80 or #81 or #82 or #83 or #84 or #85 or #86 or #87 or #88 or #89 or #90 or #91 or #92 or #93 or #94 or #95 or #96 or #97 or #98

#101 #99 AND #100 with Cochrane Library publication date Between Aug 2019 and Mar 2022

**E. Database:**  World Health Organization International Clinical Trials Registry Platform (ICTRP)

https://trialsearch.who.int

**Years of search:** August 1, 2017 – March 2022

**Date search run:**  March 9, 2022 **Number of records retrieved:** 164

**Search Strategy:**

[Basic search option]

- low back pain AND acupuncture [OR]
- low back pain AND electroacupuncture [OR]
- low back pain AND dry needling [OR]
- low back pain AND moxibustion [OR]
- lumbar stenosis AND acupuncture [OR]
- lumbar stenosis AND electroacupuncture [OR]
- lumbar stenosis AND dry needling [OR]
- lumbar stenosis AND moxibustion [OR]
- lumbar pain AND acupuncture [OR]
- lumbar pain AND electroacupuncture [OR]
- lumbar pain AND dry needling [OR]
- lumbar pain AND moxibustion

Records selected via ‘date of registration’ August 1, 2017 - current.

**F. Database:**  CNKI (China National Knowledge Infrastructure: China Academic Journals Database) https://oversea.cnki.net/index/

**Years of search:** 2017 – 2022

**Date search run:**  March 9, 2022 **Number of records retrieved:** 481

**Search Strategy:**

[basic search option – limit to *academic journals* – limit to *title, keyword, abstract* – limit to *Chinese language* – limit to *2017-2022*]

- (low back pain or lumbar pain) AND acupuncture AND random* [OR]
- (low back pain or lumbar pain) AND electroacupuncture AND random* [OR]
- (low back pain or lumbar pain) AND dry needling AND random*

**G. Database:**  WanFang MED Online: Medical Literature http://med.wanfangdata.com.cn/

**Years of search:** 2017 – 2022

**Date search run:**  March 9, 2022 **Number of records retrieved:** 74

**Search Strategy:**

[Basic search option]

- (low back pain and acupuncture) OR
- (lumbar pain or lumbar stenosis or lumbar disk or lumbar radiculopathy) and (acupuncture) OR
- (low back pain and electroacupuncture) OR
- (lumbar pain or lumbar stenosis or lumbar disk or lumbar radiculopathy) and (electroacupuncture) OR
- (low back pain and dry needling) OR
- (lumbar pain or lumbar stenosis or lumbar disk or lumbar radiculopathy) and (dry needling)

Limit to CHINESE language

Limit by date 2017- 2022

**Online Resource 2.** Description of all included randomized controlled trials (RCTs) (n=37)

| **Brinkhaus 2006[1]** (2000) | |
| --- | --- |
| Methods | **Study design:** RCT  **Setting:** Germany (high income)  **Needling therapies groups:** 1  **Comparison groups:** 2 |
| Participants | **Number of participants:** 301 (A1: 147, C1: 75, C2: 79)  **Mean age, years (SD):** A1: 59.1 (8.8); C1: 58.2 (9.4); C2: 58.9 (9.5)  **Gender (female)**^†^**:** A1: 63.7%; C1: 75.3%; C2: 68.4%  **Chronic LBP type:** nonspecific (primary)  **Mean chronic LBP duration, years (SD):** A1: 14.7 (11.0); C1:13.6 (10.5); C2: 15.8 (11.8)  **Leg pain:** NR |
| Interventions | **Intervention:** needling therapies (A1)  **Rationale:** NR  **Materials:** sterile, disposable, 1-time needles  **Procedures:** all patients treated with selection of local (at least 4 bilaterally from: BL20-34, BL50-54, GB30; GV 3-6; Huatojiaji, Shiqizhuixia) and distant points (at least 2 bilaterally from: SMI3, BL40, BL60, BL62, KI3, KI7, GB31, GB34, GB41, LI3, GV14, GV20); physicians instructed to achieve de qi (an irradiating feeling), if possible; needles were stimulated manually at least once during each session  **Format:** individual  **Duration:** 30min, 12 sessions, 8 weeks; (2 session per week for the first 4 weeks, 1 session per week for the remaining 4 weeks)  **Location:** NR  **Provider:** needling therapies physician  **Mode of delivery:** in-person  **Tailoring:** patients experiencing local or pseudoradicular symptoms, at least 2 local points were acupunctured; other needling therapies points (ear and trigger points) could be chosen individually  **Modifications:** none  **Adherence:** NR |
| Comparisons | **Comparisons assessed:**   1. needling therapies (A1) vs. minimal (sham) needling therapies (C1) 2. needling therapies (A1) vs. no treatment (C2)   **Intervention:** minimal (sham) needling therapies (C1)  **Procedure:** superficial needling of at least 6 of 10 predefined non-acupuncture points bilaterally; points were not in the area of the low back where patients were experiencing pain; de qi and manual stimulation of the needles were avoided  **Materials:** fine needles (length, 20-40 mm)  **Format:** individual  **Duration:** 30 min, 12 sessions, 8 weeks (2 session per week for the first 4 weeks, 1 session per week for the remaining 4 weeks)  **Location:** NR  **Provider:** needling therapies physician  **Mode of delivery:** in-person  **Tailoring:** none  **Modifications:** none  **Intervention:** no treatment (C2)  **Procedure:** n/a  **Materials:** n/a  **Format:** n/a  **Duration:** n/a  **Location:** n/a  **Provider:** n/a  **Mode of delivery:** n/a  **Tailoring:** n/a  **Modifications:** n/a |
| Outcomes | Pain (VAS 0-100, Pain Disability Index (PDI) 0-70)  Function (Funktionsfragebogen Hannover-Rucken (FFbH), 0-100)  Health-related quality of life (SF-36 0-100)  Depression (General Depression Scale (0-60)  Adverse events (measured for all patients at the end of week 8)  **Follow-up (post-intervention):** short term (closest to 3 months), intermediate term (closest to 6 months), or long term (closest to 12 months); C2 only followed for 8 weeks |
| Risk of bias | High (refer to Appendix 2 for details) |
| **Cherkin 2009[2]** (2001) | |
| Methods | **Study design:** RCT  **Setting:** United States (high income)  **Needling therapies groups:** 2  **Comparison groups:** 2 |
| Participants | **Number of participants:** 638 (A1: 157, A2: 158, C1: 162, C2: 161)  **Mean age, years (SD):** A1: 47 (13); A2: 49 (13); C1: 47 (14); C2: 46 (13)  **Gender (female)**^†^**:** A1: 68%; A2: 56%; C1: 60%; C2: 64%  **Chronic LBP type:** nonspecific (primary)  **Mean chronic LBP duration, years (SD):** symptoms for >1 year: A1: 69%; A2: 74%; C1: 60%; C2: 70%  **Leg pain:** Mixed with and without leg pain: reported pain below the knee: A1: 21%; A2: 22%; C1: 21%; C2: 21% |
| Interventions | **Intervention:** individualized needling therapies (A1)  **Rationale:** addresses importance of needle placement and skin penetration in eliciting effects; based on participant needs  **Materials:** sterile disposable 32-gauge needles (0.25mm) at least 1.5 inches in length  **Procedures:** seventy-four distinct points were used, half on the ‘Bladder meridian’ that includes points on the back and legs  **Format:** individual  **Duration:** 10 sessions (2x per week for 3 weeks, then weekly for 4 weeks), 18 min per session (range 15-20), 7 weeks  **Location:** research clinic  **Provider:** licensed acupuncturists  **Mode of delivery:** in-person  **Tailoring:** individualized treatment prescribed based on TCM diagnostic evaluation at beginning of each visit  **Modifications:** treatment could include any needling therapies points that could be needled with the participant lying prone; no constraints on number of needles, depth of insertion, or needle manipulation  **Adherence:** 84% (adherence defined as completion of 8 or more of the 10 possible visits)  **Intervention:** standardized needling therapies (A2)  **Rationale:** prescription considered effective by experts for chronic LBP  **Materials:** sterile disposable 32-gauge needles (0.25mm) at least 1.5 inches in length  **Procedures:** 8 acupuncture points commonly used for chronic low back pain (Du 3, Bladder 23–bilateral, low back ashi point, Bladder 40–bilateral, Kidney 3–bilateral) on the low back and lower leg. Stimulation by twirling the needles at 10 minutes and again just prior to needle removal. Therapists manipulated the needles to elicit “de qi”  **Format:** individual  **Duration:** 20 min, 10 sessions, 7 weeks (2x per week for 3 weeks, then weekly for 4 weeks)  **Location:** research clinic  **Provider:** licensed acupuncturists  **Mode of delivery:** in-person  **Tailoring:** none  **Modifications:** none  **Adherence:** 87% (adherence defined as completion of 8 or more of the 10 possible visits) |
| Comparisons | **Comparisons assessed:**   1. individualized needling therapies (A1) vs. simulated (sham) needling therapies (C1) 2. individualized needling therapies (A1) vs. usual care (C2) 3. standard needling therapies (A2) vs. simulated (sham) needling therapies 4. standard needling therapies (A2) vs. usual care (C2)   **Intervention:** simulated (sham) needling therapies (C1)  **Procedure:** using a toothpick in a needle guide tube; same point selection as standardized needling therapies group; insertion simulated (skin held taut around each needling therapies point, needle guide tube containing a toothpick placed against the skin, toothpick tapped and twisted then quickly withdrawn while holding finders against the skin for a few additional seconds); all points stimulated (point touched with the toothpick and rotated clockwise and counter clockwise) at 10 minutes and just before ‘removal’; simulated removal after 20 minutes (skin around needling therapies point stretched, cotton ball firmly placed on skin, skin lightly touched with toothpick followed by quick withdrawal)  **Materials:** toothpick, needle guide tube  **Format:** individual  **Duration:** 30 min, number of sessions not reported  **Location:** research clinic  **Provider:** licensed acupuncturists  **Mode of delivery:** in-person  **Tailoring:** none  **Modifications:** none  **Intervention:** usual care (C2)  **Procedure:** participants received no trial-related care; just the care, if any, that they and their physicians chose (mostly medications, primary care, and physical therapy visits)  **Materials:** n/a  **Format:** n/a  **Duration:** n/a  **Location:** n/a  **Provider:** n/a  **Mode of delivery:** n/a  **Tailoring:** n/a  **Modifications:** n/a |
| Outcomes | Pain (NRS 0-10)  Function (RMDQ 0-23)  Adverse events (participants were asked about adverse experiences at each clinic visit and at 8-week follow-up)  **Follow-up (post-intervention):** short term (closest to 3 months), intermediate term (closest to 6 months), long term (closest to 12 months) |
| Risk of bias | Unclear (refer to Appendix 2 for details) |
| **Cho 2013[3]** (2002) | |
| Methods | **Study design:** RCT  **Setting:** Korea (high income)  **Needling therapies groups:** 1  **Comparison groups:** 1 |
| Participants | **Number of participants:** 130 (A1: 65, C1: 65)  **Mean age, years (SD):** A1: 42.4 (14.6); C1: 41.8 (13.6)  **Gender (female)**^†^**:** A1: 82.5%; C1: 86.4%  **Chronic LBP type:** nonspecific (primary)  **Mean chronic LBP duration, years (SD):** A1: 42.4 (14.6), C1: 41.75 (13.6)  **Leg pain:** NR |
| Interventions | **Intervention:** needling therapies (A1)  **Rationale:** “real needling therapies” may be superior to “sham needling therapies”  **Materials:** sterile, disposable stainless steel needles (40 × 0.25 mm; Dongbang Acupuncture, Kyunggi-do, Korea), exercise manual  **Procedures:** individualized needling therapies treatment; providers selected one of 3 pre-specified groups of needling therapies points based on 3 types of meridian patterns (1. Gallbladder meridian pattern: GB12, GB26, GB30, GB34, GB41; 2. Bladder meridian pattern: BL23, BL24, BL25, BL37, BL40; 3. Mixed meridian pattern: ST4, ST36, SP13, SP14, GV3, GV4, GV5, GV24, GV26); needles inserted perpendicular to a depth of 5 to 20 mm depending on the needling therapies point, which was followed by manual stimulation by bidirectional rotation to induce Deqi sensation (dull, localized, and aching sensation); participants also given an exercise manual and instructed to perform exercises daily and maintain correct posture  **Format:** individual  **Duration:** 20 min, 12 sessions, 2x per week for 6 weeks  **Location:** hospital  **Provider:** licensed Korean medicine doctors  **Mode of delivery:** in-person, home exercise  **Tailoring:** other needling therapies points could be used according to diagnosis  **Modifications:** NR  **Adherence:** NR (participants were asked to complete more than 80% of the 12 possible treatments) |
| Comparisons | **Comparisons assessed:** needling therapies (A1) + home exercise vs. sham needling therapies (C1) + home exercise  **Intervention:** sham needling therapies (C1)  **Procedure:** 8 predefined points in lower back unrelated to traditional needling therapies points (1cm below BL39, 1cm lateral to BL18, 1cm lateral to BL20, 2cm above GB30 bilaterally); participants also given an exercise manual and instructed to perform exercises daily and maintain correct posture  **Materials:** semi-blunt nonpenetrating sham needle (Acuprime, Exeter, UK), exercise manual  **Format:** individual  **Duration:** 20 min, 12 sessions, 2x per week for 6 weeks  **Location:** hopsital  **Provider:** licensed Korean medicine doctors  **Mode of delivery:** in-person, home exercise  **Tailoring:** NR  **Modifications:** NR |
| Outcomes | Pain (VAS 0-10)  Function (ODI 0-100)  Health-related quality of life (SF-36 0-100)  Depression (BDI 0-63)  Adverse events (participants asked about adverse events at each visit)  **Follow-up (post-intervention):** short term (closest to 3 months), intermediate term (closest to 6 months) |
| Risk of bias | Unclear (refer to Appendix 2 for details) |
| **de Castro Moura 2019[4]** (1167) | |
| Methods | **Study design:** RCT  **Setting:** Brazil (upper-middle income)  **Needling therapies groups:** 1  **Comparison groups:** 2 |
| Participants | **Number of participants:** 110 (A1: 37, C1: 36, C2: 37)  **Mean age, years (SD):** A1: 47.5 (13.9), C1: 51.0 (14.9), C2: 46.2 (15.7)  **Gender (female)** ^†^**:** A1: 81.1%, C1: 75.0%, C2: 81.1%  **Chronic LBP type:** nonspecific (primary)  **Mean chronic LBP duration, months (SD):** A1: 47.5 (13.9), C1: 51.0 (14.9), C2: 46.2 (15.7)  **Leg pain:** NR |
| Interventions | **Intervention:** auricular needling therapies (A1)  **Rationale:** stimuli in the ear activates meridians (energy channels) to increase the flow of *Qi* (vital energy) and *Xue* (blood) to promote pain relief; or auricular needling therapies mechanisms are related to the neuroendocrine, immune, and autonomic nervous systems, which jointly contribute to relieve pain  **Materials:** semipermanent needles, 0.20 x 1.5 mm (Complementar Agulhas®), microporous tape  **Procedures:** acupoints were located with an Acu-Treat (DongBang®) localization device and needles were inserted and attached with micropore; standardized TCM point selection: Shenmen (TF4); kidney (CO10); sympathetic nervous system (AH6a); points of restoration of the energy balance, corresponding to an organ and a viscus; and cervical vertebrae (AH13), thoracic vertebrae (AH11), and/or lumbosacral vertebrae (AH9)  **Format:** individual  **Duration:** 5 sessions, 6 weeks  **Location:** NR  **Provider:** ‘professional specialized in needling therapies’  **Mode of delivery:** in-person  **Tailoring:** none  **Modifications:** NR  **Adherence:** NR |
| Comparisons | **Comparisons assessed:**   1. auricular needling therapies (A1) vs. placebo auricular needling therapies (C1) 2. auricular needling therapies (A1) vs. no intervention (C2)   **Intervention:** placebo auricular needling therapies (C1)  **Procedure:** single auricular point selected - eye point (LO5); point located in center of the earlobe and distant from points applied in the treatment group with no relationship to the focus of the trial  **Materials:** sterilized and disposable semi-permanent auricular needle (0.20 x 1.5 mm)  **Format:** individual  **Duration:** 5 sessions, 6 weeks  **Location:** NR  **Provider:** ‘professional specialized in needling therapies’  **Mode of delivery:** in-person  **Tailoring:** none  **Modifications:** NR  **Intervention:** no intervention (C2)  **Procedure:** participants received no orientation and were submitted to no intervention during the evaluation period  **Materials:** n/a  **Format:** n/a  **Duration:** n/a  **Location:** n/a  **Provider:** n/a  **Mode of delivery:** n/a  **Tailoring:** n/a  **Modifications:** n/a |
| Outcomes | Pain (Brief Pain Inventory, subscale 1 0-10)  Function (Brief Pain Inventory, subscale 2 0-10)  **Follow-up (post-intervention):** short term (closest to 3 months) |
| Risk of bias | High (refer to Appendix 2 for details) |
| Notes | Same group of participants as reference 32 |
| **de Castro Moura 2019[5]** (32) | |
| Methods | **Study design:** RCT  **Setting:** Brazil (upper-middle income)  **Needling therapies groups:** 2  **Comparison groups:** 1 |
| Participants | **Number of participants:** 111 (A1: 37, A2: 37, C1: 37)  **Mean age, years (SD):** A1: 47.5 (13.9), A2: 50.2 (13.7), C1: 46.2 (15.7)  **Gender (female)**^†^**:** A1: 81.1%, A2: 78.9%, C1: 81.1%  **Chronic LBP type:** nonspecific (primary)  **Mean chronic LBP duration, months (SD):** A1: 132.2 (133.9), A2: 94.8 (95.7), C1: 125.1 (126.8)  **Leg pain:** NR |
| Interventions | **Intervention:** Chinese ear acupuncture (A1)  **Rationale:** stimuli in the ear activates meridians (energy channels) to increase the flow of *Qi* (vital energy) and *Xue* (blood) to promote pain relief  **Materials:** semipermanent needles, microporous tape  **Procedures:** semipermanent needles inserted and fixed with microporous tape and worn until following appointment (1 week); alternation ear at each session  **Format:** individual  **Duration:** 5 sessions, 5 weeks  **Location:** NR  **Provider:** ‘needling therapies expert’  **Mode of delivery:** in-person  **Tailoring:** needling therapies points varied by participant  **Modifications:** NR  **Adherence:** NR  **Intervention:** French ear acupuncture (A2)  **Rationale:** effects of stimulating auricular points are explained by reflexology and other neurophysiological mechanisms  **Materials:** semipermanent needles, microporous tape  **Procedures:** five fixed points, always in the same order: Zero, Cervical Column, Thoracic Column, Lumbar Column and Synthesis. Semipermanent needles inserted and fixed with microporous tape and worn until following appointment (1 week); alternation ear at each session the ear in each session.  **Format:** individual  **Duration:** 5 sessions, 5 weeks  **Location:** NR  **Provider:** ‘needling therapies expert’  **Mode of delivery:** in-person  **Tailoring:** none  **Modifications:** NR  **Adherence:** NR |
| Comparisons | **Comparisons assessed:**   1. Chinese ear acupuncture (A1) vs. no treatment/waitlist (C1) 2. French ear acupuncture (A2) vs. no treatment/waitlist (C1)   **Intervention:** no treatment/waitlist (C1)  **Procedure:** n/a  **Materials:** n/a  **Format:** n/a  **Duration:** n/a  **Location:** n/a  **Provider:** n/a  **Mode of delivery:** n/a  **Tailoring:** n/a  **Modifications:** n/a |
| Outcomes | Pain (Brief Pain Inventory 0-10)  Function (RMDQ 0-24)  **Follow-up (post-intervention):** immediate term (closest to 2 weeks), short term (closest to 3 months) |
| Risk of bias | High (refer to Appendix 2 for details) |
| Notes | Same group of participants as reference 1167 |
| **Depaoli Lemos 2021[6]** (1152) | |
| Methods | **Study design:** RCT  **Setting:** Brazil (upper-middle income)  **Needling therapies groups:** 1  **Comparison groups:** 1 |
| Participants | **Number of participants:** 32 (A1: 16, C1: 16)  **Mean age, years (SD):** A1: 45.4 (13.5), C1: 50.8 (12.9)  **Gender (female)**^†^**:** A1: 62.5%, C1: 68.8%  **Chronic LBP type:** nonspecific (primary)  **Mean chronic LBP duration, years (SD):** A1: 7.37 (5.5), C1: 5.0 (3.6)  **Leg pain:** none |
| Interventions | **Intervention:** electroacupuncture + kinesiotherapy (A1)  **Rationale:** electroacupuncture modulates pain through changes in opioids, serotonin, and norepinephrine leading to therapeutic effects such as analgesia  **Materials:** electrocostimulator (brand Sikuro®, model DS100jr; ANVISA MS registration number 80470920001), 25 × 40 mm needles (brand Gold Dragon®)  **Procedures:** kinesiotherapy: stretching and strengthening exercises: supine bridge, single leg supine bridge, side bridge, prone plank. Electroacupuncture: after kinesiotherapy, an acupuncture electrostimulator was used; needles were used for puncture at bladder meridian points B22 (L1) and B26 (L5)  **Format:** individual  **Duration:** 20min (electroacupuncture alone), 10 sessions, 4 weeks  **Location:** NR  **Provider:** NR  **Mode of delivery:** in-person  **Tailoring:** NR  **Modifications:** NR  **Adherence:** NR |
| Comparisons | **Comparisons assessed:** electroacupuncture + kinesiotherapy (A1) vs. kinesiotherapy (C1)  **Intervention:** kinesiotherapy (C1)  **Procedure:** kinesiotherapy: stretching and strengthening exercises: supine bridge, single leg supine bridge, side bridge, prone plank  **Materials:** none  **Format:** individual  **Duration:** 10 sessions, 4 weeks  **Location:** NR  **Provider:** NR  **Mode of delivery:** in-person  **Tailoring:** NR  **Modifications:** NR |
| Outcomes | Pain (VAS 0-10)  Function (RMDQ 0-24)  **Follow-up (post-intervention):** immediate term (closest to 2 weeks), short term (closest to 3 months) |
| Risk of bias | High (refer to Appendix 4 for details) |
| **Haake 2007[7]** (2003) | |
| Methods | **Study design:** RCT  **Setting:** Germany (high income)  **Needling therapies groups:** 1  **Comparison groups:** 1 |
| Participants | **Number of participants:** 774 (A1: 387, C1: 387)  **Mean age, years (SD):** A1: 49.6 (14.6), C1: 49.2 (14.8)  **Gender (female)**^†^**:** A1: 57.4%, C1: 63.8%  **Chronic LBP type:** nonspecific (primary)  **Mean chronic LBP duration, years (SD):** A1: 8.1 (7.7), C1: 7.7 (8.2)  **Leg pain:** NR |
| Interventions | **Intervention:** verum needling therapies (A1)  **Rationale:** ‘verum needling therapies has never been directly compared with sham needling therapies’  **Materials:** sterile, disposable needles (Asiamed, Pullach near Munich, Germany)  **Procedures:** needling fixed points and additional points (from a prescribed list) chosen individually on the basis of traditional Chinese medicine diagnosis (14-20 needles), including tongue diagnosis. Induction of de Qi was elicited by manual stimulation  **Format:** individual  **Duration:** 30 min, 5 sessions, 6 weeks  **Location:** outpatient practices  **Provider:** physicians of various practices with needling therapies training  **Mode of delivery:** in-person  **Tailoring:** five additional sessions were offered to patients who had a partial response to treatment (10%- 50% reduction in pain intensity). The number of patients who received the 5 additional sessions was 232 (59.9%) in the verum group, and 209 (54.3%) in the sham group.  **Modifications:** NR  **Adherence:** NR |
| Comparisons | **Comparisons assessed:** verum needling therapies (A1) vs. sham needling therapies (C1)  **Intervention:** sham needling therapies (C1)  **Procedure: s**ham needling therapies on either side of the lateral part of the back and on the lower limbs was also standardized, avoiding all known verum points or meridians. As  with verum needling therapies, 14 to 20 needles were inserted, but superficially (1-3 mm) and without stimulation  **Materials:** sterile, disposable needles (Asiamed, Pullach near Munich, Germany)  **Format:** individual  **Duration:** 30 min, 5 sessions, 6 weeks  **Location:** outpatient practices  **Provider:** physicians of various practices with needling therapies training  **Mode of delivery:** in-person  **Tailoring:** NR  **Modifications:** NR |
| Outcomes | Pain (Von Korff Pain Scale 0-70)  Function (Hanover Functional Ability Questionnaire 0-100)  Health-related quality of life (SF-36 0-100)  Adverse events (recorded when reported by participants)  **Follow-up (post-intervention):** immediate term (closest to 2 weeks), short term (closest to 3 months), intermediate term (closest to 6 months) |
| Risk of bias | Low (refer to Appendix 2 for details) |
| **Huang 2019[8]** (52) | |
| Methods | **Study design:** RCT  **Setting:** China (upper-middle income)  **Needling therapies groups:** 1  **Comparison groups:** 1 |
| Participants | **Number of participants:** 46 (A1: 23, C1: 23)  **Mean age, years (SD):** A1: 63.0 (14.0), C1: 63.0 (11.0)  **Gender (female)**^†^**:** A1: 69.6%), C1: 65.2%  **Chronic LBP type:** single level lumbar disc herniation: A1: 47.8%, C1: 34.8%; multilevel disc herniation: A1: 52.2%, C1: 65.2%  **Mean chronic LBP duration, weeks (SD):** A1: 36.5 (14.0); C1: 35.6 (26.4)  **Leg pain:** 100% 'discogenic sciatica' |
| Interventions | **Intervention:** needling therapies (A1)  **Rationale:** to relieve inflammatory and neuropathic pain  **Materials:** disposable Hwato needles (Suzhou Medical Appliance Factory in China)  **Procedures:** penetrating needle needling therapies to 4 TCM points bilaterally (BL25, BL23, BL40, BL57); needles inserted approximately 40-70mm into BL25 until de qi occurred and radiated down to the lower limb; needles inserted approximately 30mm for all other points; twirling, lifting, and thrusting manipulations were performed tenderly and evenly three times in order to reach de qi sensation  **Format:** individual  **Duration:** 30 min, 12 sessions, 4 weeks  **Location:** hospital  **Provider:** experienced acupuncturists  **Mode of delivery:** in-person  **Tailoring:** NR  **Modifications:** NR  **Adherence:** 100% |
| Comparisons | **Comparisons assessed:** needling therapies (A1) vs. sham needling therapies (C1)  **Intervention:** sham needling therapies (C1)  **Procedure:** received treatment on the same acupoints. Procedures were similar with the needling therapies group but without skin penetration and manipulations to reach the de qi sensation  **Materials:** blunt-tipped needles (size ¼ 0.30 mm 25 mm)  **Format:** individual  **Duration:** 30 min, 12 sessions, 4 weeks  **Location:** hospital  **Provider:** experienced acupuncturists  **Mode of delivery:** in-person  **Tailoring:** NR  **Modifications:** NR |
| Outcomes | Pain (VAS (low back pain and leg pain) 0-100)  Function (ODI 0-100)  Health-related quality of life (SF-36 0-100)  Adverse events (recorded when reported by participants)  **Follow-up (post-intervention):** immediate term (closest to 2 weeks), short term (closest to 3 months), intermediate term (closest to 6 months) |
| Risk of bias | Low (refer to Appendix 2 for details) |
| **Kerr 2003[9] (**2004) | |
| Methods | **Study design:** RCT  **Setting:** Ireland (high income)  **Needling therapies groups:** 1  **Comparison groups:** 1 |
| Participants | **Number of participants:** 46 (A1: 26, C1: 20)  **Mean age, years (SD):** A1: 42.6 (11.5), C1: 42.8 (12.0)  **Gender (female)**^†^**:** A1: 43.3%, C1: 43.3%  **Chronic LBP type:** nonspecific (primary)  **Mean chronic LBP duration, months (SD):** A1: 86.1 (84.9), C1: 72.8 (77.4)  **Leg pain:** NR |
| Interventions | **Intervention:** needling therapies (A1)  **Rationale:** ‘needling therapies has been advocated as an effective treatment of low back pain’  **Materials:** Seirin needling therapies needles (No. 8; 0.30 × 50 mm; c-type)  **Procedures:** penetrating needle acupuncture using set needling therapies points (bilateral BL23, BL25, GB30, BL40, KI3, and GV4); eleven needles used for each session; needles were inserted until the sensation of ‘ch’I’ was produced  **Format:** individual  **Duration:** 30 min, 6 sessions, 6 weeks  **Location:** NR  **Provider:** physiotherapist trained in needling therapies  **Mode of delivery:** in-person  **Tailoring:** NR  **Modifications:** NR  **Adherence:** NR |
| Comparisons | **Comparisons assessed:** needling therapies (A1) vs. placebo TENS (C1)  **Intervention:** placebo TENS (C1)  **Procedure:** treatment carried out with patient lying in the prone position for 30 minutes. A non-functioning TENS machine was attached to 4 electrodes placed over the lumbar spine, and the unit was placed so as to make it difficult to interfere with the apparatus  **Materials:** non-functioning TENS machine  **Format:** individual  **Duration:** 30 min, 6 sessions, 6 weeks  **Location:** NR  **Provider:** principal investigator  **Mode of delivery:** in-person  **Tailoring:** none  **Modifications:** none |
| Outcomes | Pain (VAS 0-100)  Health-related quality of life (SF-36 0-100)  Adverse events (all patients asked about adverse events at 6 months)  **Follow-up (post-intervention):** immediate term (closest to 2 weeks) |
| Risk of bias | High (refer to Appendix 2 for details) |
| **Kim 2020[10]** (542) | |
| Methods | **Study design:** RCT  **Setting:** United States (high income)  **Needling therapies groups:** 1  **Comparison groups:** 2 |
| Participants | **Number of participants:** 55 (A1: 18, C1: 18, C2: 19)  **Mean age, years (SD):** A1: 41.3 (14.0), C1: 41.8 (12.2), C2: 41.7 (12.3)  **Gender (female)**^†^**:** A1: 61.1%, C1: 38.9%, C2: 57.9%  **Chronic LBP type:** nonspecific (primary)  **Mean chronic LBP duration, years (SD):** A1: 7.6 (7.2), C1: 10.6 (10.8), C2: 6.0 (5.4)  **Leg pain:** NR |
| Interventions | **Intervention:** verum needling therapies (A1)  **Rationale:** needling therapies may improve tactile acuity and relieve pain  **Materials:** verum needling therapies needles (0.20–0.25 mm diameter, 25–50 mm length, stainless steel; Asiamed)  **Procedures:** acupoint locations selected based on the standardized needling therapies protocol for cLBP (GV-3, BL-23 bilateral, BL-40 bilateral, KI-3 bilateral), combined with 2–3 bilateral ah-shi (tender by palpation) points over the lower back/buttocks. Needles inserted 10–40 mm deep, depending on location, and retained for 20 min, with manual needle stimulation (2 Hz) via twirling at 10 min and again just before needle removal  **Format:** individual  **Duration:** 20 min, 6 sessions, 4 weeks  **Location:** hospital  **Provider:** acupuncturists  **Mode of delivery:** in-person  **Tailoring:** 2–3 bilateral ah-shi (tender by palpation) points over the lower back/buttocks  **Modifications:** NR  **Adherence:** NR |
| Comparisons | **Comparisons assessed:**   1. verum needling therapies (A1) vs. sham needling therapies (C1) 2. verum needling therapies (A1) vs. mock laser needling therapies (C2)   **Intervention:** sham needling therapies (C1)  **Procedure:** same acupoints were used as verum group without skin penetration  (i.e. stimulation restricted to cutaneous levels).  **Materials:** non-inserted Streitberger sham needles  **Format:** individual  **Duration:** 20 min, 6 sessions, 4 weeks  **Location:** hospital  **Provider:** acupuncturists  **Mode of delivery:** in-person  **Tailoring:** NR  **Modifications:** NR  **Intervention:** mock laser needling therapies (C2)  **Procedure:** waved deactivated laser needling therapies device over acupoints for at least 15 sec per point  **Materials:** deactivated laser needling therapies device (Vita-Laser 650, Lhasa OMS)  **Format:** individual  **Duration:** 20 min, 6 sessions, 4 weeks  **Location:** hospital  **Provider:** acupuncturists  **Mode of delivery:** in-person  **Tailoring:** NR  **Modifications:** NR |
| Outcomes | Pain (pain bothersomeness, scale not specified 0-10)  **Follow-up (post-intervention):** short term (closest to 3 months) |
| Risk of bias | High (refer to Appendix 4 for details) |
| **Kong 2020[11]** (41) | |
| Methods | **Study design:** RCT  **Setting:** United States (high income)  **Needling therapies groups:** 1  **Comparison groups:** 1 |
| Participants | **Number of participants:** 121 (A1: 59, C1: 62)  **Mean age, years (SD):** A1: 46.8 (11.9), C1: 45.6 (12.8)  **Gender (female)**^†^**:** A1: 61.0%, C1: 53.2%  **Chronic LBP type:** NR (assumed nonspecific primary)  **Mean chronic LBP duration, years (SD):** NR  **Leg pain:** None |
| Interventions | **Intervention:** electroacupuncture verum needling therapies (A1)  **Rationale:** electroacupuncture may lead to stronger analgesia than manual needling therapies  **Materials:** acupuncture needle, acupuncture stimulator  **Procedures:** body needling therapies using both local anatomy driven points (Bladder back Shu points in the affected dermatome; optional periosteal needle in posterior superior iliac spine and deep muscular point in affected gluteal region for patient with radiating pain to buttocks/hips) and distal points (KI-3, KI-7, HR-3, SI-3, BL-40, GV-3 and GV-20; optional addition of KI-10, BL-10, and BL-60 for those who do not improve after 4 sessions); de qi sensation elicited upon insertion; electrical stimulation (2Hz, intensity set to elicit visible muscular twitching) for 20-25 minutes on local points and 10 minutes over distal points. Each participant also received 30 minutes of heat applied to the back using a heat lamp and standardized penetrating auricular needling therapies (4, fixed, pain specific points)  **Format:** individual  **Duration:** 45 min, 12 sessions, 6 weeks  **Location:** NR  **Provider:** acupuncturists  **Mode of delivery:** in-person  **Tailoring:** standardized penetrating auricular needling therapies (4, fixed, pain specific points)  **Modifications:** NR  **Adherence:** 96% (allowed up to 3 missed sessions total (25%), two of which may be consecutive. 52 attended the assessment visits and were included in the ITT analysis (50 participants had 9 treatment sessions) |
| Comparisons | **Comparisons assessed:** electroacupuncture verum needling therapies (A1) vs. sham electroacupuncture (C1)  **Intervention:** sham electroacupuncture (C1)  **Procedure:** non-penetrating needling therapies using 8 sham points (four on the trunk and four on the upper/lower extremity) located at least 2 cm away from known meridians, away from the participants’ area of maximum pain and away from the center of the low back; points hooked up to needling therapies stimulator with broken wires for 20-25 minutes; each participant also received 10 minutes of low intensity heat applied to the calves and sham non-penetrating auricular needling therapies (2 square pieces of tape placed in locations non known to have any specific effect)  **Materials:** needling therapies stimulator with broken wires  **Format:** individual  **Duration:** 45 min, 12 sessions, 6 weeks  **Location:** NR  **Provider:** acupuncturists  **Mode of delivery:** in-person  **Tailoring:** NR  **Modifications:** NR |
| Outcomes | Pain (National Institutes of Health PROMIS pain intensity instrument 0-100)  Function (RMDQ 0-24)  Adverse events (assessed at the end of each treatment)  **Follow-up (post-intervention):** short term (closest to 3 months) |
| Risk of bias | High (refer to Appendix 2 for details) |
| **Koppenhaver 2021[12]** (166) | |
| Methods | **Study design:** RCT  **Setting:** United States (high income)  **Needling therapies groups:** 1  **Comparison groups:** 1 |
| Participants | **Number of participants:** 60 (A1: 30, C1: 30)  **Mean age, years (SD):** A1: 32.2 (8.2), C1: 32.3 (6.6)  **Gender (female)**^†^**:** A1: 46.7%, C1: 33.3%  **Chronic LBP type:** nonspecific (primary)  **Mean chronic LBP duration, months (SD):** A1: 84.6 (90.7); C1: 68.7 (73.5)  **Leg pain:** none |
| Interventions | **Intervention:** dry needling (A1)  **Rationale:** inserting needles into painful areas of muscle perceived to have motor abnormalities may restore normal muscle function and relieve pain  **Materials:** seirin J-Type or Myotech needle or myotech needle  **Procedures:** treatment was applied to four sites on the most symptomatic side, to both the lumbar multifidi and erector spinae muscles, at the two most symptomatic levels (between L3, L4, and L5). If unable to determine the most symptomatic levels or areas, treatment was performed to the middle of the muscle belly at the L4 and L5 levels  **Format:** individual  **Duration:** 1 session, 1 week  **Location:** NR  **Provider:** physical therapist  **Mode of delivery:** in-person  **Tailoring:** treatment provided to most symptomatic side  **Modifications:** NR  **Adherence:** 100% |
| Comparisons | **Comparisons assessed:** dry needling (A1) vs. sham dry needling (C1)  **Intervention:** sham dry needling (C1)  **Procedure:** identical to dry needling treatment. The needle was placed in the guide tube to mimic insertion and then was rocked and twisted against the skin so as to simulate dry needling treatment without actually piercing the skin  **Materials:** seirin J-Type or Myotech needle or myotech needle with the tip cut off that did not pierce the skin  **Format:** individual  **Duration:** 1 session, 1 week  **Location:** NR  **Provider:** physical therapist  **Mode of delivery:** in-person  **Tailoring:** NR  **Modifications:** NR |
| Outcomes | Pain (NRS 0-10)  Function (ODI 0-100)  Adverse events (recorded when participants reported adverse events)  **Follow-up (post-intervention):** immediate term (closest to 2 weeks) |
| Risk of bias | High (refer to Appendix 2 for details) |
| **Li 2019[13]** (565) | |
| Methods | **Study design:** RCT  **Setting:** China (upper-middle income)  **Needling therapies groups:** 1  **Comparison groups:** 1 |
| Participants | **Number of participants:** 60 (A1: 30, C1: 30)  **Mean age, years (SD):** A1: 49 (12),C1: 50 (12)  **Gender (female)**^†^**:** A1: 26.7%, C1: 30%  **Chronic LBP type:** nonspecific (primary)  **Mean chronic LBP duration, months (SD):** A1: 23.8 (8.8); C1: 24.0 (8.9)  **Leg pain:** none |
| Interventions | **Intervention:** needling therapies + oral celecoxib (A1)  **Rationale:** improve function and relieve pain  **Materials:** needling therapies needles, celecoxib 200 mg  **Procedures:** needling therapies applied at Yaoda (Extra), Wushu (GB 27), Qiayao (Extra); oral celecoxib 200 mg  **Format:** individual  **Duration:** 20 min, 3 sessions, 3 days  **Location:** hospital  **Provider:** NR  **Mode of delivery:** in-person  **Tailoring:** NR  **Modifications:** NR  **Adherence:** NR |
| Comparisons | **Comparisons assessed:** needling therapies + oral celecoxib (A1) vs. oral celecoxib (C1)  **Intervention:** oral celecoxib 200 mg (C1)  **Procedure:** n/a  **Materials:** celecoxib, 200 mg  **Format:** individual  **Duration:** once/day for 3 days  **Location:** hospital  **Provider:** NR  **Mode of delivery:** in-person  **Tailoring:** n/a  **Modifications:** n/a |
| Outcomes | Pain (VAS 0-10)  Function (Japanese Orthopaedic Association Score 0-29)  **Follow-up (post-intervention):** immediate term (closest to 2 weeks) |
| Risk of bias | High (refer to Appendix 4 for details) |
| **Li 2022[14]** (116) | |
| Methods | **Study design:** RCT  **Setting:** China (upper-middle income)  **Needling therapies groups:** 1  **Comparison groups:** 1 |
| Participants | **Number of participants:** 75 (A1: 38, C1: 37)  **Mean age, years (SD):** A1: 42.8 (9.7), C1:41.7 (9.5)  **Gender (female)**^†^**:** A1: 42.9%, C1: 37.1%  **Chronic LBP type:** nonspecific (primary)  **Mean chronic LBP duration, months (SD):** A1: 42.7 (8.9), C1: 41.9 (9.2)  **Leg pain:** none |
| Interventions | **Intervention:** fire needle needling therapies + core stability training (A1)  **Rationale:** analgesia  **Materials:** sterile disposable steel needles (0.35mmX25mm, 0.35mmX40mm, 0.35mmX75mm)  **Procedures:** needles burned to white color before inserting. Yaoyangguan (GV3), bilateral Shenshu (BL23), Weizhong (BL40), Zhibian, Jiaji and ashi points in the prone position; after 10 minutes, in the sitting position for Jingming and Zhiyin  **Format:** individual  **Duration:** 12 sessions, 4 weeks  **Location:** hospital  **Provider:** NR  **Mode of delivery:** in-person  **Tailoring:** NR  **Modifications:** NR  **Adherence:** NR |
| Comparisons | **Comparisons assessed:** fire needle needling therapies + core stability training (A1) vs. core stability training (C1)  **Intervention:** core stability training (C1)  **Procedure:** 8 different bridge style exercises, each last 10-15 seconds for 20 repetitions  **Materials:** n/a  **Format:** individual  **Duration:** 30 min, 12 sessions, 4 weeks  **Location:** hospital  **Provider:** NR  **Mode of delivery:** in-person  **Tailoring:** NR  **Modifications:** NR |
| Outcomes | Pain (VAS 0-10)  Function (ODI 0-50)  **Follow-up (post-intervention):** immediate term (closest to 2 weeks) |
| Risk of bias | High (refer to Appendix 2 for details) |
| **Martin-Corrales 2020[15]** (23) | |
| Methods | **Study design:** RCT  **Setting:** Spain (high income)  **Needling therapies groups:** 1  **Comparison groups:** 1 |
| Participants | **Number of participants:** 46 (A1: 23, C1: 23)  **Mean age, years (SD):** A1: 49 (11.4), C1: 47.6 (11.1)  **Gender (female)**^†^**:** A1: 56.5%, C1: 60.9%  **Chronic LBP type:** NR (assumed nonspecific primary)  **Mean chronic LBP duration, years (SD):** NR  **Leg pain:** NR |
| Interventions | **Intervention:** dry needling therapy + exercise (A1)  **Rationale:** mechanically disrupt tissue without the use of anaesthetic to treat myofascial trigger points  **Materials:** sterile, disposable 0.30 - 0.60-mm needling therapies needle (Agu-punt, Barcelona, Spain)  **Procedures:** participants underwent a single session of dry needling therapy. Needling procedure started with systematic manual flat palpation of the gluteus medius to determine the presence or absence of perceived myofascial trigger points. Technique included insertion of needling therapies needle into the gluteus medius muscles bilaterally. The needles were directed into the gluteus medius muscle to the depth of the pelvis. Then, needling was applied on the basis of the technique described by Hong (1994). Both groups also received the same four-week exercise program. The exercise program consisted of two weekly  supervised group sessions of an hour and involved specific gluteus medius exercises including double leg bridge, traditional hip claim exercise, prone heel squeeze, and side-lying hip abduction with internal rotation exercises. Each exercise was performed on three sets of 10 repetitions bilaterally  **Format:** individual  **Duration:** 1 session, 4 weeks (4-week length of the program includes exercise but only one session of needling therapies administered before exercise program)  **Location:** physiotherapy and pain clinic  **Provider:** physical therapist  **Mode of delivery:** in-person  **Tailoring:** NR  **Modifications:** NR  **Adherence:** NR |
| Comparisons | **Comparisons assessed:** dry needling therapy + exercise (A1) vs. sham dry needling + exercise (C1)  **Intervention:** sham dry needling + exercise (C1)  **Procedure:** Park sham needles cause a pricking sensation on the skin as the real needle,  but do not penetrate the skin as the shaft telescoped into the handle. The sham needle process was similar to that for dry needling group.  **Materials:** sham dry needles (Park Sham Device, AcuPrime, UK)  **Format:** individual  **Duration:** 1 session, 4 weeks (4-week length of the program includes exercise but only one session of sham needling therapies administered before exercise program)  **Location:** physiotherapy and pain clinic  **Provider:** physical therapist  **Mode of delivery:** in-person  **Tailoring:** NR  **Modifications:** NR |
| Outcomes | Pain (VAS 0-10)  Function (RMDQ 0-24)  Fear avoidance (FABQ-Work, FABQ-PA 0-96)  **Follow-up (post-intervention):** immediate term (closest to 2 weeks), short term (closest to 3 months) |
| Risk of bias | Unclear (refer to Appendix 2 for details) |
| **Meng 2003[16]** (2012) | |
| Methods | **Study design:** RCT  **Setting:** United States (high income)  **Needling therapies groups:** 1  **Comparison groups:** 1 |
| Participants | **Number of participants:** 55 (A1: 31, C1: 24)  **Mean age, years (SD):** A1: 72 (5), C1: 70 (6)  **Gender (female)**^†^**:** A1: 58%, C1: 62.5%  **Chronic LBP type:** NR (assumed nonspecific primary)  **Mean chronic LBP duration, years (SD):** A1: 12 (16), C1: 12 (14)  **Leg pain:** none |
| Interventions | **Intervention:** needling therapies + standard therapy (usual care) (A1)  **Rationale:** needling therapies stimulates release of endorphins leading to analgesia  **Materials:** disposable, sterile, 30-guage needles, electrical stimulation machine (details not reported)  **Procedures:** aseptic technique, 30-guage needles with electrical stimulation at 4-6Hz with a pulse duration of 0.5ms; point selection based on 10 standardized points (Shenshu (UB23), Qihai shu (UB24), Dachangshu (UB25), Pangguangshu (UB28), Yoayangguan (Du3), Mingmen (Du4)) with the option of four additional points to treat concomitant buttock or leg pain; de qi response at all points was verified  **Format:** individual  **Duration:** 20 min/session, 10 sessions, 5 weeks  **Location:** NR  **Provider:** anaesthetist certified in needling therapies  **Mode of delivery:** in-person  **Tailoring:** a maximum of four additional needles was allowed: UB36, 54, 37, 40, GB30, 31 to individualize the regimen to treat each subject's specific symptoms  **Modifications:** NR  **Adherence:** NR |
| Comparisons | **Comparisons assessed:** needling therapies + standard therapy (A1) vs. standard therapy (C1)  **Intervention:** standard therapy (C1)  **Procedure:** as directed by primary physician. NSAIDS, aspirin, and non-narcotic analgesic medications were allowed. Patients were also allowed to continue back exercises (i.e., physical therapy or home exercises). Prohibited therapies were narcotic medications, muscle relaxants, TENS, epidural steroid injections and trigger point injections  **Materials:** n/a  **Format:** individual  **Duration:** n/a  **Location:** n/a  **Provider:** primary physician  **Mode of delivery:** in-person  **Tailoring:** n/a  **Modifications:** NR |
| Outcomes | Pain (VAS): Authors do not describe scale. Based on how outcomes are reported they may have scaled the measure from 0-5  Function (RMDQ 0-24)  Medication use (medication diary)  Adverse events (recorded when reported by participants)  **Follow-up (post-intervention):** immediate (closest to 2 weeks), short term (closest to 3 months) |
| Risk of bias | High (refer to Appendix 2 for details) |
| **Molsberger 2002[17]** (2007) | |
| Methods | **Study design:** RCT  **Setting:** Germany (high income)  **Needling therapies groups:** 1  **Comparison groups:** 2 |
| Participants | **Number of participants:** 186 (A1: 65, C1: 60, C2: 61)  **Mean age, years (SD):** A1: 49 (8), C1: 49 (7), C2: 50 (6)  **Gender (female)**^†^**:** A1: 44.6%, C1: 53.3%, C2: 41%  **Chronic LBP type:** nonspecific (primary)  **Mean chronic LBP duration, years (SD):** A1: 11.5 (9.2), C1: 8.1 (5.7), C2: 9.9 (7.7)  **Leg pain:** none |
| Interventions | **Intervention:** verum needling therapies + conventional orthopedic therapy (A1)  **Rationale:** needling specific needling therapies points reduces pain  **Materials:** verum needling therapies needles  **Procedures:** standard points in the lumbar region (adjacent points) were urinary bladder 23, 25, and gallbladder 30; standard points on the lower extremity (distal points) were urinary bladder 40, 60 and gallbladder 34. Additionally up to four points of maximum pain ‘Ahshi points’ (locus dolendi, trigger points), which were often close but not necessarily identical to Bl 54, 31, 32 were needled. Depending on the site of the needle and the type of pain reported by the patient, needle insertion ranged from 1 to 10 mm and needle manipulation was mild to strong. Always a numb, warm feeling around the needling therapies point (Deqi) was achieved.  **Format:** individual  **Duration:** 30 min/session, 12 sessions, 4 weeks  **Location:** clinic  **Provider:** medical doctor  **Mode of delivery:** in-person  **Tailoring:** Depending on the site of the needle and the type of pain reported by the patient, needle insertion ranged from 1 to 10 mm and needle manipulation was mild to strong.  **Modifications:** NR  **Adherence:** NR |
| Comparisons | **Comparisons assessed:**   1. verum needling therapies + conventional orthopedic therapy (A1) vs. conventional orthopedic therapy alone (C1) 2. verum needling therapies + conventional orthopedic therapy (A1) vs. sham needling therapies + conservative orthopedic therapy (C2)   **Intervention:** conventional orthopedic therapy alone (C1)  **Procedure:** conventional conservative orthopedic treatment only. Participants received physiotherapy, physical exercise, back school, mud packs, infrared heat therapy. On demand they received 50 mg diclofenac up to three times a day. Injections or cortison application of any kind were not allowed. Other than that, information and handling of these patients was identical to those of the other two groups.  **Materials:** n/a  **Format:** individual  **Duration:** ‘standardized, daily basis’  **Location:** clinic  **Provider:** medical doctor  **Mode of delivery:** in-person  **Tailoring:** 50 mg diclofenac up to three times a day  **Modifications:** NR  **Intervention:** sham needling therapies + conservative orthopedic therapy (C2)  **Procedure:** sham needling therapies was standardized to ten needles applied superficially (depth of needle insertion was less than 1 cm) at defined non-needling therapies points of the lumbar region, and five needles on either side of the back. Other than the application of sham needling therapies, information and handling of these patients was identical to those of the verum group.  **Materials:** needling therapies needles  **Format:** individual  **Duration:** 30 min/session, 12 sessions, 4 weeks  **Location:** clinic  **Provider:** medical doctor  **Mode of delivery:** in-person  **Tailoring:** none  **Modifications:** NR |
| Outcomes | Pain (VAS 0-100)  Adverse events (procedure not reported)  **Follow-up (post-intervention):** immediate term (closest to 2 weeks), short term (closest to 3 months) |
| Risk of bias | High (refer to Appendix 2 for details) |
| **Moslemi 2020[18]** (305) | |
| Methods | **Study design:** RCT  **Setting:** Iran (lower-middle income)  **Needling therapies groups:** 1  **Comparison groups:** 1 |
| Participants | **Number of participants:** 38 (A1: 19, C1: 19)  **Mean age, years (SD):** A1: 38.5 (9.5), C1: 42 (8.3)  **Gender (female)**^†^**:** NR  **Chronic LBP type:** nonspecific (primary)  **Mean chronic LBP duration, years (SD):** NR  **Leg pain:** NR |
| Interventions | **Intervention:** electroacupuncture + routine physiotherapy (A1)  **Rationale:** stimulates endorphins and increases blood circulation, which reduces muscle spasm and pain  **Materials:** NR  **Procedures:** electroacupuncture including needle insertion into needling therapies points and trigger points of the gluteus maximus; electrical stimulation administered at 2 Hz using continuous mode for 20 minutes. The needles were inserted into the needling therapies points and the trigger points of the gluteus maximus muscle, which were previously found by touch and using ultrasonography. Physiotherapy included the use of TENS and pulsed ultrasonography; timeline (occurring before or after the needling therapies) is unclear  **Format:** individual  **Duration:** 20 min per session, 4 sessions, 2 weeks (electroacupuncture); 24 minutes per session, 10 sessions, 2 weeks (physiotherapy)  **Location:** NR  **Provider:** NR  **Mode of delivery:** in-person  **Tailoring:** NR  **Modifications:** NR  **Adherence:** NR |
| Comparisons | **Comparisons assessed:** electroacupuncture + routine physiotherapy (A1) vs. routine physiotherapy (C1)  **Intervention:** routine physiotherapy (C1)  **Procedure:** Physiotherapy included the use of TENS (20 min) and pulsed ultrasonography (4 min)  **Materials:** TENS, ultrasonography  **Format:** individual  **Duration:** 24 minutes per session, 10 sessions, 2 weeks (physiotherapy)  **Location:** NR  **Provider:** NR  **Mode of delivery:** in-person  **Tailoring:** NR  **Modifications:** NR |
| Outcomes | Pain (VAS 0-10)  Function (ODI 0-50)  **Follow-up (post-intervention):** immediate term (closest to 2 weeks) |
| Risk of bias | High (refer to Appendix 4 for details) |
| **Pan 2019[19]** (1528) | |
| Methods | **Study design:** RCT  **Setting:** China (upper-middle)  **Needling therapies groups:** 1  **Comparison groups:** 1 |
| Participants | **Number of participants:** 100 (A1: 50, C1: 50)  **Mean age, years (SD):** A1: 43.3 (11.6), C1: 44.5 (12.6)  **Gender (female)**^†^**:** A1: 38%, C1: 42%  **Chronic LBP type:** lumbar disc hernation  **Mean chronic LBP duration, years (SD):** A1: 3.8 (2.9), C1: 3.6 (2.8)  **Leg pain:** 100% |
| Interventions | **Intervention:** needling therapies + medication and exercise (A1)  **Rationale:** needle therapies are a well-accepted and effective treatment  **Materials:** 0.30 mm x 60 mm and 0.30 mm x 80 mm needling therapies needles  **Procedures:** needling therapies in addition to treatment received by control group. Manual stimulation. Needling therapies points: jiaji, shenyu, ashi, zhibian, chengfu, yinmen, huantiao, xuanzhong  **Format:** individual  **Duration:** 30 min, 15 sessions, 3 weeks  **Location:** hospital  **Provider:** NR  **Mode of delivery:** in-person  **Tailoring:** NR  **Modifications:** NR  **Adherence:** NR |
| Comparisons | **Comparisons assessed:** needling therapies + medication and exercise (A1) vs. medication and exercise (C1)  **Intervention:** medication and exercise (C1)  **Procedure:** nimesulli capsules orally and with SET for weak-link muscles  **Materials:** nimesulli capsules orally and with sling exercise training for weak-link muscles  **Format:** individual  **Duration:** 20 minutes per session, 5 sessions per week for 3 weeks  **Location:** hospital  **Provider:** NR  **Mode of delivery:** in-person  **Tailoring:** NR  **Modifications:** NR |
| Outcomes | Pain (VAS 0-10, McGill Pain Questionnaire)  Function (ODI 0-50, Japanese Orthopaedic Association Scale 0-29)  **Follow-up (post-intervention):** immediate term (closest to 2 weeks) |
| Risk of bias | High (refer to Appendix 2 for details) |
| **Ran 2021[20]** (1335) | |
| Methods | **Study design:** RCT  **Setting:** China (upper-middle)  **Needling therapies groups:** 1  **Comparison groups:** 1 |
| Participants | **Number of participants:** 280 (A1: 140, C1: 140)  **Mean age, years (SD):** A1: 50.3 (3.6), C1: 50.5 (3.7)  **Gender (female)**^†^**:** A1: 30%, C1: 29.9%  **Chronic LBP type:** nonspecific (primary)  **Mean chronic LBP duration, years (SD):** A1: 2.2 (0.7), C1: 2.1 (0.6)  **Leg pain:** NR |
| Interventions | **Intervention:** needling therapies (Shiqizui points) + conventional needling therapies (A1)  **Rationale:** stimulates local metabolism, depress or delay physiological changes, improve local disc microcirculation, lead to analgesia  **Materials:** 0.30 mm x 40 mm and 0.30 mm x 50 mm needling therapies needles  **Procedures:** needling therapies and moxibustion on the basis of conventional needling therapies Shiqizui points in addition to interventions control group received  **Format:** individual  **Duration:** 30 min, 24 sessions, 4 weeks  **Location:** public hospital  **Provider:** NR  **Mode of delivery:** in-person  **Tailoring:** NR  **Modifications:** NR  **Adherence:** NR |
| Comparisons | **Comparisons assessed:** needling therapies (Shiqizui points) + conventional needling therapies (A1) vs. conventional needling therapies (C1)  **Intervention:** conventional needling therapies (C1)  **Procedure:** conventional needling therapies treatment  **Materials:** 0.30 mm x 40 mm and 0.30 mm x 50 mm needling therapies needles  **Format:** individual  **Duration:** 24 sessions, 4 weeks  **Location:** public hospital  **Provider:** NR  **Mode of delivery:** in-person  **Tailoring:** NR  **Modifications:** NR |
| Outcomes | Pain (VAS 0-10)  Function (ODI 0-45)  **Follow-up (post-intervention):** immediate term (closest to 2 weeks) |
| Risk of bias | High (refer to Appendix 2 for details) |
| **Ren 2021[21]** (1327) | |
| Methods | **Study design:** RCT  **Setting:** China (upper-middle)  **Needling therapies groups:** 1  **Comparison groups:** 1 |
| Participants | **Number of participants:** 96 (A1: 48, C1: 48)  **Mean age, years (SD):** A1: 39.1 (6,1), C1: 38.8 (6)  **Gender (female)**^†^**:** A1: 35.4%, C1: 31.2%  **Chronic LBP type:** nonspecific (primary)  **Mean chronic LBP duration, months (SD):** A1: 15.2 (5.1) , C1: 15 (5)  **Leg pain:** NR |
| Interventions | **Intervention:** needling therapies (meridian-tendon dialectical theory) + traditional acupoint needling therapies + core stability exercise (A1)  **Rationale:** improve blood and qi circulation and lead to analgesia  **Materials:** 0.30 mm x 50 mm needling therapies needles  **Procedures:** needling therapies based on meridian-tendon dialectical theory in addition to same interventions control group received. Based on patients' pain points.  **Format:** individual  **Duration:** 30 min, 40 sessions, 8 weeks  **Location:** public hospital  **Provider:** acupuncturist  **Mode of delivery:** in-person  **Tailoring:** based on patients' pain points  **Modifications:** NR  **Adherence:** NR |
| Comparisons | **Comparisons assessed:** needling therapies (meridian-tendon dialectical theory) + traditional acupoint needling therapies + core stability exercise (A1) vs. traditional acupoint needling therapies + core stability exercise (C1)  **Intervention:** traditional acupoint needling therapies + core stability exercise (C1)  **Procedure:** core stability training and needling therapies based on traditional acupoint selection and needling therapies method  **Materials:** NR  **Format:** individual  **Duration:** 30 min, 40 sessions, 8 weeks  **Location:** public hospital  **Provider:** acupuncturist  **Mode of delivery:** in-person  **Tailoring:** NR  **Modifications:** NR |
| Outcomes | Pain (VAS 0-10)  Function (RMDQ 0-24)  **Follow-up (post-intervention):** immediate term (closest to 2 weeks) |
| Risk of bias | High (refer to Appendix 2 for details) |
| **Shi 2021[22]** (1376) | |
| Methods | **Study design:** RCT  **Setting:** China (upper-middle)  **Needling therapies groups:** 1  **Comparison groups:** 1 |
| Participants | **Number of participants:** 72 (A1: 36, C1: 36)  **Mean age, years (SD):** A1: 36.1 (6.2), C1: 35.9 (7.3)  **Gender (female)**^†^**:** A1: 52.8%, C1: 50%  **Chronic LBP type:** nonspecific (primary)  **Mean chronic LBP duration, months (SD):** A1: 10.1 ( 3.4), C1: 11.2 (3.2)  **Leg pain:** NR |
| Interventions | **Intervention:** abdominal needling therapies + waist and back needling therapies (A1)  **Rationale:** improve low back pain and function in a safe and efficient manner  **Materials:** 0.25 mm x 40 mm needling therapies needles  **Procedures:** patients were instructed to lie on the back. The needling therapies points on the abdomen such as Qihaici, Guanyuanci, Tianshuci and Shufenci were selected and the same palpation method was used to determine the abdominal tendon lesions, and 2 to 6 lesions were selected each time, and the needle was retained for 10 min after getting Qi.  **Format:** individual  **Duration:** 10 min, 20 sessions, 4 weeks  **Location:** hospital  **Provider:** NR  **Mode of delivery:** in-person  **Tailoring:** varied by patient according to abdominal tendon lesions  **Modifications:** NR  **Adherence:** NR |
| Comparisons | **Comparisons assessed:** abdominal needling therapies + waist and back needling therapies (A1) vs. waist and back needling therapies (C1)  **Intervention:** waist and back needling therapies (C1)  **Procedure:** placed in the prone position and the lateral lying position, and the focal points of the waist and back tendons were determined by palpation of the tendons. The needles were kept for 30min after getting Qi.  **Materials:** 0.35 mm x 50 mm and 0.35 mm x 75 mm needling therapies needles  **Format:** individual  **Duration:** 30 minutes, 20 sessions, 4 weeks  **Location:** hospital  **Provider:** NR  **Mode of delivery:** in-person  **Tailoring:** varied by patient according to waist and back tendon lesions  **Modifications:** NR |
| Outcomes | Pain (VAS 0-10)  Function (ODI 0-50)  **Follow-up (post-intervention):** immediate term (closest to 2 weeks) |
| Risk of bias | High (refer to Appendix 2 for details) |
| **Sung 2020[23]** (296) | |
| Methods | **Study design:** RCT  **Setting:** South Korea (high income)  **Needling therapies groups:** 1  **Comparison groups:** 1 |
| Participants | **Number of participants:** 38 (A1: 19, C1: 19)  **Mean age, years (SD):** A1: 56.6 (14.6), C1: 44.1 (14.1)  **Gender (female)**^†^**:** A1: 73.7%, C1: 57.9%  **Chronic LBP type:** nonspecific (primary)  **Mean chronic LBP duration, years (SD):** NR  **Leg pain:** none |
| Interventions | **Intervention:** thread embedding needling therapies + needling therapies (A1)  **Rationale:** the thread dissolves in the subcutaneous tissue or muscle. This may produce an inflammatory response, resulting in tissue recovery.  **Materials:** 40mm length (Hyundae Meditech Co., Weonju, South Korea)  **Procedures:** Thread embedding needling therapies treatment by polydioxanone sutures of 29 gauge and 40mm length on the muscles of the lumbar region. Thread embedding needling therapies was performed with, a 4cm perpendicular insertion at the bilateral EX-B2 of the L4–5 and L5–S1 for multifidus muscle stimulation, a 4cm transverse insertion at the bilateral 3 to 4cm of L3 and S1 spinous processes toward L1 for spinal erector muscle stimulation, and a 4cm transverse insertion at the bilateral 3 to 4cm of L4 transverse process toward iliac crest for lumbar quadrate muscle stimulation. Lumbar-local needling therapies points were GV3 and bilateral BL23, BL24, BL25, BL26, and EX-B5 while distal needling therapies points were bilateral BL40 and BL60.  **Format:** individual  **Duration:** 16 sessions, 8 weeks  **Location:** hospital  **Provider:** Korean medical doctors  **Mode of delivery:** in-person  **Tailoring:** NR  **Modifications:** NR  **Adherence:** 100% |
| Comparisons | **Comparisons assessed:** thread embedding needling therapies + needling therapies (A1) vs. needling therapies (C1)  **Intervention:** needling therapies (C1)  **Procedure:** lumbar-local needling therapies points were GV3 and bilateral BL23, BL24, BL25, BL26, and EX-B5 while distal needling therapies points were bilateral BL40 and BL60  **Materials:** sterilized stainless steel needles of 0.25mm width and 40mm length (DB108C; Dongbang Medical Co., Boryung-si, South Korea)  **Format:** individual  **Duration:** 16 sessions, 8 weeks  **Location:** hospital  **Provider:** Korean medical doctors  **Mode of delivery:** in-person  **Tailoring:** NR  **Modifications:** NR |
| Outcomes | Pain (VAS 0-100)  Function (RMDQ 0-24)  Health-related quality of life (EQ-5D-3L 0-1)  General health (EQ-VAS)  Global overall improvement (Patient Global Impression of Change)  Adverse events (recorded when reported by participants)  **Follow-up (post-intervention):** immediate term (closest to 2 weeks) |
| Risk of bias | Unclear (refer to Appendix 2 for details) |
| **Tabatabaiee 2019[24]** (108) | |
| Methods | **Study design:** RCT  **Setting:** Iran (low income)  **Needling therapies groups:** 1  **Comparison groups:** 1 |
| Participants | **Number of participants:** 32 (A1: 16, C1: 16)  **Mean age, years (SD):** A1: 31.3 (6.1), C1: 30.3 (5.5)  **Gender (female)**^†^**:** A1: 66.6%, C1: 60%  **Chronic LBP type:** NR  **Mean chronic LBP duration, months (SD):** A1: 5.22 (1.59), C1: 6.10 (1.72)  **Leg pain:** with (23%) and without leg pain |
| Interventions | **Intervention:** ultrasound guided dry-needling (A1)  **Rationale:** ‘affect deep target tissue that is not manually palpable’  **Materials:** 0.35 × 100-mm sterile needling therapies needle (Maanshan Bond Medical, Instrument Co, China), ultrasound  **Procedures:** needling therapies needle was inserted through the skin beside the ultrasound transducer using a guide tube. The guide tube was then removed, and the needle was moved toward the piriformis muscle while the tip was observed using ultrasound. The dynamic needling technique was performed by slowly moving the needle in and out of the piriformis muscle with the goal of eliciting a local twitch response, a quick contraction of the local muscle fibers in response to mechanical perturbation, detected by ultrasound visualization  **Format:** individual  **Duration:** 3 sessions, 1 week  **Location:** NR  **Provider:** physiotherapist  **Mode of delivery:** in-person  **Tailoring:** NR  **Modifications:** NR  **Adherence:** 93.75% |
| Comparisons | **Comparisons assessed:** ultrasound guided dry-needling (A1) vs. no treatment (C1)  **Intervention:** no treatment (C1)  **Procedure:** n/a  **Materials:** n/a  **Format:** n/a  **Duration:** n/a  **Location:** n/a  **Provider:** n/a  **Mode of delivery:** n/a  **Tailoring:** n/a  **Modifications:** n/a |
| Outcomes | Pain (VAS 0-10)  Function (ODI 0-100)  **Follow-up (post-intervention):** immediate term (closest to 2 weeks) |
| Risk of bias | High (refer to Appendix 2 for details) |
| **Ushinohama 2016[25]** (2009) | |
| Methods | **Study design:** RCT  **Setting:** Brazil (upper-middle)  **Needling therapies groups:** 1  **Comparison groups:** 1 |
| Participants | **Number of participants:** 80 (A1: 40, C1: 40)  **Mean age, years (SD):** A1: 37.9 (7.7), C1: 34.3 (8.9)  **Gender (female)**^†^**:** A1: 67.5%, C1: 57.5  **Chronic LBP type:** nonspecific (primary)  **Mean chronic LBP duration, months (SD):** A1: 46.3 (38.3); C1: 39.5 (35.4)  **Leg pain:** none |
| Interventions | **Intervention:** ear needling therapies (A1)  **Rationale:** “French version is based on the assumption that the human body is represented in the outer ear as an inverted fetus and that there is a relationship between anatomical sites and specific points in the ear’  **Materials:** disposable Dong Bang needles (0.15×30 mm)  **Procedures:** received ear needling therapies in three points: point 29 (analgesic point), point 40 (shenmen point), and point 55 (low back point)  **Format:** individual  **Duration:** 20 minutes, 1 session  **Location:** NR  **Provider:** ‘experienced therapist’  **Mode of delivery:** in-person  **Tailoring:** none  **Modifications:** none  **Adherence:** 100% |
| Comparisons | **Comparisons assessed:** ear needling therapies (A1) vs. placebo ultrasound (C1)  **Intervention:** placebo ultrasound (C1)  **Procedure:** the ultrasound machine was turned on, but not activated (i.e. no vibration was transmitted to the skin). The head of the ultrasound was placed in light contact with the skin of the painful lower back region and was kept in constant circular motion for minimal interference with the painful area  **Materials:** ultrasound (Sonopulse III, Ibramed, Brazil)  **Format:** individual  **Duration:** 20 minutes, 1 session  **Location:** NR  **Provider:** ‘experienced therapist’  **Mode of delivery:** in-person  **Tailoring:** none  **Modifications:** none |
| Outcomes | Pain (NPRS 0-10)  Adverse events (methods not reported)  **Follow-up (post-intervention):** immediate term (closest to 2 weeks) |
| Risk of bias | High (refer to Appendix 2 for details) |
| **Wang 2020a[26]** (1429) | |
| Methods | **Study design:** RCT  **Setting:** China (upper-middle)  **Needling therapies groups:** 1  **Comparison groups:** 1 |
| Participants | **Number of participants:** 92 (A1: 46, C1: 46)  **Mean age, years (SD):** A1: 43.8 (5.3), C1: 43.6 (4.3)  **Gender (female)**^†^**:** A1: 26.1%, C1: 31.1%  **Chronic LBP type:** lumbar disc herniation  **Mean chronic LBP duration, years (SD):** A1: 5.2 (1.1), C1: 5.09 (1.1)  **Leg pain:** 100% |
| Interventions | **Intervention:** fire needle needling therapies + conventional needling therapies with electronic stimulation (A1)  **Rationale:** stimulate local tissues  **Materials:** 0.40 mm x 35 mm needling therapies needles  **Procedures:** fire needle treatment in addition to intervention received by the control group. Heated needles. Six needling therapies points: bilateral Jiaji points (3 points each side). Needles in and out without being retained  **Format:** individual  **Duration:** 4 sessions, 2 weeks  **Location:** hospital  **Provider:** NR  **Mode of delivery:** in-person  **Tailoring:** NR  **Modifications:** NR  **Adherence:** NR |
| Comparisons | **Comparisons assessed:** fire needle needling therapies + conventional needling therapies with electronic stimulation (A1) vs. conventional needling therapies with electronic stimulation (C1)  **Intervention:** conventional needling therapies with electronic stimulation (C1)  **Procedure:** conventional needling therapies (dachangyu, jizhong, shenyu, weizhong and ashi points with selected yanglingquan, huantiao, chengshan, zhiian, zusanli and liangqiu points based on patients’ condition) and electronic stimulation with 10-16 Hz  **Materials:** NR  **Format:** individual  **Duration:** one dose per day, 7 days as one section, rest for 2 days before the next section  **Location:** hospital  **Provider:** NR  **Mode of delivery:** in-person  **Tailoring:** optional yanglingquan, huantiao, chengshan, zhiian, zusanli and liangqiu points based on patients’ condition  **Modifications:** NR |
| Outcomes | Pain (VAS 0-10)  Function (Japanese Orthopaedic Association Score 0-29)  **Follow-up (post-intervention):** immediate term (closest to 2 weeks) |
| Risk of bias | High (refer to Appendix 2 for details) |
| **Wang 2020b[27]** (1456) | |
| Methods | **Study design:** RCT  **Setting:** China (upper middle income)  **Needling therapies groups:** 1  **Comparison groups:** 1 |
| Participants | **Number of participants:** 80 (A1: 40, C1: 40)  **Mean age, years (SD):** A1: 49 (10), C1: 51 (9)  **Gender (female)**^†^**:** A1: 60%, C1: 72.5%  **Chronic LBP type:** lumbar disc herniation  **Mean chronic LBP duration, months (SD):** A1: 4.4 (2.2), C1: 4.7 (2.4)  **Leg pain:** 100% |
| Interventions | **Intervention:** needling therapies at neck acupoints + conventional needling therapies (A1)  **Rationale:** tendon softening and bone strengthening, and simultaneous treatment of neck and waist  **Materials:** 0.30 mm x 40 mm and 0.30 mm x 50 mm needling therapies needles  **Procedures:** needling therapies at neck acupoints based on the theory of "tendon softening and bone strengthening, and simultaneous treatment of neck and waist" in addition conventional needling therapies as received by control group. Manual stimulation applied. 5 needling therapies points: bilateral fengchi, jinbailao, tianzhu, ashi  **Format:** individual  **Duration:** 30 min, 10 sessions, 2 weeks  **Location:** hospital  **Provider:** NR  **Mode of delivery:** in-person  **Tailoring:** NR  **Modifications:** NR  **Adherence:** NR |
| Comparisons | **Comparisons assessed:** needling therapies at neck acupoints + conventional needling therapies (A1) vs. conventional needling therapies (C1)  **Intervention:** conventional needling therapies (C1)  **Procedure:** needling jiaji, weizhong, huantiao, yanglingquan and ashi points  **Materials:** 0.30 mm x 40 mm and 0.30 mm x 50 mm needling therapies needles  **Format:** individual  **Duration:** 30 minutes, 10 sessions, 2 weeks  **Location:** hospital  **Provider:** NR  **Mode of delivery:** in-person  **Tailoring:** NR  **Modifications:** NR |
| Outcomes | Pain (VAS 0-100)  Function (Japanese Orthopaedic Association Score 0-29)  **Follow-up (post-intervention):** immediate term (closest to 2 weeks), short term (closest to 3 months) |
| Risk of bias | High (refer to Appendix 2 for details) |
| **Weiß 2013[28]** (1153) | |
| Methods | **Study design:** RCT  **Setting:** Germany (high income)  **Acupuncture groups:** 1  **Comparison groups:** 1 |
| Participants | **Number of participants:** 160 (A1: 80, C1: 80)  **Mean age, years (SD):** A1: 49.8 (7.9), C1: 51.7 (7.4)  **Gender (female)**^†^**:** A1: 46%, C1: 39.1  **Chronic LBP type:** NR (assumed nonspecific primary)  **Mean chronic LBP duration, months (SD):** A1: 11.1 (7.6), C1: 11.6 (9.3)  **Leg pain:** NR |
| Interventions | **Intervention:** acupuncture + standardized rehabilitation (A1)  **Rationale:** relieve pain  **Materials:** two different types of sterile disposable needles: VQ-3210 (0.25 · 25 mm) and VQ-3205 (0.25 · 13 mm), Tuina massage and a magnet lamp (TDP-lamp CQ-35, Chongqing Xinfeng Medical Instruments Co. Ltd., Chongqing, China)  **Procedures:** fixed positions of the needles were not mandated so that the therapists were not restricted in their treatment options. Each patient was treated individually according to the opinion of the TCM physician. Patients were advised to rest for 30 minutes after acupuncture. All patients also participated in a standardized 21-day inpatient rehabilitation  program according to current German guidelines.  **Format:** individual  **Duration:** 30 minutes, 6 sessions, 3 weeks + 30 minutes of rest following acupuncture  **Location:** inpatient rehabilitation clinic  **Provider:** traditional Chinese medicine practitioner  **Mode of delivery:** in-person  **Tailoring:** each patient was treated individually according to the opinion of the TCM physician  **Modifications:** NR  **Adherence:** NR |
| Comparisons | **Comparisons assessed:** acupuncture + standardized rehabilitation (A1) vs. standardized rehabilitation (C1)  **Intervention:** standardized rehabilitation (C1)  **Procedure:** standardized 21-day inpatient rehabilitation program according to current German guidelines  **Materials:** NR  **Format:** NR  **Duration:** ‘twice weekly on a fixed schedule’  **Location:** inpatient rehabilitation clinic  **Provider:** traditional Chinese medicine practitioner  **Mode of delivery:** in-person  **Tailoring:** NR  **Modifications:** NR |
| Outcomes | Pain (Self-developed questionnaire measuring quality, intensity and duration of pain)  Health-related quality of life (SF-36 0-100)  Adverse events (self-developed questionnaire)  **Follow-up (post-intervention):** immediate (closest to 2 weeks), short term (closest to 3 months) |
| Risk of bias | High (refer to Appendix 2 for details) |
| **Witt 2006[29]** (2010) | |
| Methods | **Study design:** RCT  **Setting:** Germany (high income)  **Acupuncture groups:** 1  **Comparison groups:** 1 |
| Participants | **Number of participants:** 3093 (A1: 1549, C1: 1544)  **Mean age, years (SD):** A1: 53.1 (13.5), C1: 52.6 (13.2)  **Gender (female)**^†^**:** A1: 57.7%, C1: 56.9%  **Chronic LBP type:** nonspecific (primary)  **Mean chronic LBP duration, years (SD):** A1: 7.2 (8.0), C1: 7.2 (7.8)  **Leg pain:** none |
| Interventions | **Intervention:** acupuncture (A1)  **Rationale:** relieve pain  **Materials:** disposable one-time needles  **Procedures:** needle acupuncture (with disposable one-time needles and manual stimulation). Patients were allowed to use additional conventional treatments as needed  **Format:** individual  **Duration:** 15 sessions (each patient received a maximum of 15 acupuncture sessions), 12 weeks  **Location:** multicentre  **Provider:** physician  **Mode of delivery:** in-person  **Tailoring:** acupuncture points and the number of needles used to the discretion of each physician  **Modifications:** NR  **Adherence:** most patients (74%) received 5–10 sessions, whereas 21% received more than 10 sessions and 5% received fewer than five sessions |
| Comparisons | **Comparisons assessed:** acupuncture (A1) vs. waitlist control group (C1)  **Intervention:** waitlist control group (C1)  **Procedure:** received no treatment for the first 3 months and acupuncture treatment in the last 3 months of the trial  **Materials:** n/a  **Format:** n/a  **Duration:** n/a  **Location:** n/a  **Provider:** n/a  **Mode of delivery:** n/a  **Tailoring:** n/a  **Modifications:** n/a |
| Outcomes | Pain (Low Back Pain Rating Scale 0-130)  Function (Hannover Functional Ability Questionnaire 0-100)  Health-related quality of life (SF-36 0-100)  Adverse events (evaluated using patient and physician questionnaires after 3 months)  **Follow-up(post-intervention):** short term (closest to 3 months) |
| Risk of bias | High (refer to Appendix 2 for details) |
| **Yeung 2003[30]** (2013) | |
| Methods | **Study design:** RCT  **Setting:** China (upper middle)  **Acupuncture groups:** 1  **Comparison groups:** 1 |
| Participants | **Number of participants:** 52 (A1: 26, C1: 26)  **Mean age, years (SD):** A1: 50.4 (16.3), C1: 55.6 (10.4)  **Gender (female)**^†^**:** A1: 84.6%, C1: 80.8%  **Chronic LBP type:** prolapsed intervertebral disc: A1: 12%, C1: 0%  **Percentage chronic LBP duration, months:** A1: 6 months: 7.7%, 7-12 months: 19.2%, 13-18 months: 11.5%, 9-24 months: 7.7%, > 25 months: 53.8%; C1: 6 months: 15.4%, 7-12 months: 7.7%; 13-18 months: 7.7%, 19-24 months: 11.5%, > 25 months: 57.7%  **Leg pain:** A1: 53.8%; C1: 53.8% |
| Interventions | **Intervention:** electroacupuncture + exercise (A1)  **Rationale:** analgesia  **Materials:** sterilized disposable number 30 (0.3mm x 40mm), electrical stimulator (Shanhai Medical Technology Co., Shanghai)  **Procedures:** electroacupuncture; participants in prone position, needles inserted into pre-specified points on most painful side: UB23, UB25, UB40, SP6. Needles manipulated until de qi obtained; needles coupled to an electrical stimulator at a frequency of 2 Hz for 30 minutes; intensity of stimulation set at level that patient could tolerate often with evoked visible muscle contractions; current was applied with biphasic waveform with 0.5 ms pulse width to foru selected acupoints in two pairs (UB-23/UB-25 pair and UB40/SP6 pair). Standard group exercise program consisting of back strengthening and stretching exercises; advice on anatomy, biomechanics, back care, postural correction, lifting and ergonomic advice, behavioural modification; home exercises (patients were instructed to perform the designated types of back exercise every day over the period of the trial)  **Format:** individual (needling therapies), group (exercise)  **Duration:** 30 min, 12 sessions, 4 weeks (needling therapies), 1-hour, 4 sessions, 4 weeks (group exercise), 15min daily home exercise for 4 weeks  **Location:** outpatient clinic  **Provider:** physiotherapist  **Mode of delivery:** in-person  **Tailoring:** postural correction, lifting and ergonomic advice, behavioural modification  **Modifications:** NR  **Adherence:** NR |
| Comparisons | **Comparisons assessed:** electroacupuncture + exercise (A1) vs. exercise (C1)  **Intervention:** exercise (C1)  **Procedure:** standard group exercise program consisting of back strengthening and stretching exercises; advice on anatomy, biomechanics, back care, postural correction, lifting and ergonomic advice, behavioural modification; home exercises (patients were instructed to perform the designated types of back exercise every day over the period of the trial)  **Materials:** n/a  **Format:** group, individual  **Duration:** 1-hour, 4 sessions, 4 weeks (group exercise), 15min daily home exercise for 4 weeks  **Location:** outpatient clinic  **Provider:** physiotherapist  **Mode of delivery:** in-person, home  **Tailoring:** postural correction, lifting and ergonomic advice, behavioural modification  **Modifications:** NR |
| Outcomes | Pain (NRS 0-10)  Function (Aberdeen LBP Scale 0-100)  Adverse events (methods not reported)  **Follow-up (post-intervention):** immediate term (closest to 2 weeks), short term (closest to 3 months) |
| Risk of bias | High (refer to Appendix 2 for details) |
| **Yu 2020[31]** (183) | |
| Methods | **Study design:** RCT  **Setting:** United States  **Needling therapies groups:** 2  **Comparison groups:** 2 |
| Participants | **Number of participants:** 55 (A1: 14, A2: 14, C1: 14, C2: 13)  **Mean age, years (SD):** A1: 43.0 (11.1), A2: 35.0 (13.2), C1: 40.0 (13.5), C2: 39.5 (14.4)  **Gender (female)**^†^**:** A1: 67% A2: 67%, C1: 61.5%, C2: 53.9%  **Chronic LBP type:** nonspecific (primary)  **Mean chronic LBP duration, years (SD):** NR  **Leg pain:** NR |
| Interventions | **Intervention:** needling therapies + structured education (A1)  **Rationale:** analgesia through opioidergic descending pain modulation system  **Materials:** needling therapies needles (additional details not reported)  **Procedures:** 7 needling therapies points included Yaoyangguan (GV3), bilateral Shenshu (BL23), bilateral Weizhong (BL40), bilateral Taixi (KI3), and 1-3 ashi points bilaterally on the lower back and legs. Additional stimulation was applied to elicit deqi by twirling the needles at 10 min and again just prior to needle removal. Structured interaction with the acupuncturist. The acupuncturist’s interaction with the subject was structured with respect to both content (conversations) and style (five primary behaviors: (1) exuding a warm, friendly manner; (2) active listening (such as repeating patient’s words, asking for clarifications); (3) empathy (such as saying “I can understand how diffcult cLBP must be for you”); (4) 20 s of thoughtful silence while taking the patient’s pulse or pondering the treatment plan; and (5) communication of confidence and positive expectation)  **Format:** individual  **Duration:** 25 min, 6 sessions, 4 weeks  **Location:** NR  **Provider:** acupuncturist  **Mode of delivery:** in-person  **Tailoring:** NR  **Modifications:** NR  **Adherence:** NR  **Intervention:** needling therapies + limited education (A2)  **Rationale:** analgesia through opioidergic descending pain modulation system  **Materials:** needling therapies needles (additional details not reported)  **Procedures:** 7 needling therapies points included Yaoyangguan (GV3), bilateral Shenshu (BL23), bilateral Weizhong (BL40), bilateral Taixi (KI3), and 1-3 ashi points bilaterally on the lower back and legs. Additional stimulation was applied to elicit deqi by twirling the needles at 10 min and again just prior to needle removal. Limited education included acupuncturist reading trial information to the patient and aimed to converse with patients as little as possible.  **Format:** individual  **Duration:** 25 min, 6 sessions, 4 weeks  **Location:** NR  **Provider:** acupuncturist  **Mode of delivery:** in-person  **Tailoring:** NR  **Modifications:** NR  **Adherence:** NR |
| Comparisons | **Comparisons assessed:**   1. needling therapies + structured education (A1) vs. sham needling therapies + structured education (C1) 2. needling therapies + limited education (A2) vs. sham needling therapies + limited education (C2)   **Intervention:** sham needling therapies + structured education (C1)  **Procedure:** 12 sham needling therapies points. Nonacupoints were located based on (1) relative closeness to the real points selected and (2) convenience of administration. Instead of penetrating the skin, the point of the Streitberger needle retracts up the handle shaft when the acupuncturist presses it against the skin. Structured interaction with the acupuncturist. The acupuncturist’s interaction with the subject was structured with respect to both content (conversations) and style (five primary behaviors: (1) exuding a warm, friendly manner; (2) active listening (such as repeating patient’s words, asking for clarifications); (3) empathy (such as saying “I can understand how diffcult cLBP must be for you”); (4) 20 s of thoughtful silence while taking the patient’s pulse or pondering the treatment plan; and (5) communication of confidence and positive expectation).  **Materials:** Streitberger placebo needling therapies needle  **Format:** individual  **Duration:** 25 min, 6 sessions, 4 weeks  **Location:** NR  **Provider:** acupuncturist  **Mode of delivery:** in-person  **Tailoring:** NR  **Modifications:** NR  **Intervention:** sham needling therapies + limited education (C2)  **Procedure:** 12 sham needling therapies points. Nonacupoints were located based on (1) relative closeness to the real points selected and (2) convenience of administration. Instead of penetrating the skin, the point of the Streitberger needle retracts up the handle shaft when the acupuncturist presses it against the skin. Limited education included acupuncturist reading trial information to the patient and aimed to converse with patients as little as possible.  **Materials:** Streitberger placebo needling therapies needle  **Format:** individual  **Duration:** 25 min, 6 sessions, 4 weeks  **Location:** NR  **Provider:** acupuncturist  **Mode of delivery:** in-person  **Tailoring:** NR  **Modifications:** NR |
| Outcomes | Pain (VAS 0-10)  Depression (Beck Depression Inventory 0-63)  **Follow-up (post-intervention):** immediate term (closest to 2 weeks) |
| Risk of bias | High (refer to Appendix 2 for details) |
| **Yu 2021[32]** (187) | |
| Methods | **Study design:** RCT  **Setting:** China  **Needling therapies groups:** 1  **Comparison groups:** 1 |
| Participants | **Number of participants:** 60 (A1: 30, C1: 30)  **Mean age, years (SD):** A1: 40.1 (13.8), C1: 40.9 (14.5)  **Gender (female)** ^†^**:** A1: 46.7%, C1: 50%  **Chronic LBP type:** lumbar disc herniation  **Mean chronic LBP duration, months (SD):** A1: 12.8 (9.4); C1: 10.8 (7.1)  **Leg pain:** 100% |
| Interventions | **Intervention:** electroacupuncture + caudal epidural injection (A1)  **Rationale:** reduce pan and improve function  **Materials:** NR  **Procedures:** bilateral Jiaji (EX-82) and adjunct points Guanyuanshu (BL26), Shenshu (BL23), Chengfu (BL36 ), Huantiao (GB30), Zhibian (BL54) on the affected side were stimulated with EA (2 Hz/16 Hz, 5 - 8 mA). Ultrasound guided caudal epidural injection (1ml steroid, 5ml lidocaine and 10ml sodium chloride solution)  **Format:** individual  **Duration:** 30 min, 12 sessions, 4 weeks  **Location:** hospital  **Provider:** NR  **Mode of delivery:** in-person  **Tailoring:** NR  **Modifications:** NR  **Adherence:** NR |
| Comparisons | **Comparisons assessed:** electroacupuncture + caudal epidural injection (A1) vs. caudal epidural injection (C1)  **Intervention:** caudal epidural injection (C1)  **Procedure:** ultrasound guided caudal epidural injection; 1ml steroid, 5ml lidocaine and 10ml sodium chloride solution  **Materials:** 1ml steroid, 5ml lidocaine and 10ml sodium chloride solution  **Format:** individual  **Duration:** 2 sessions, 4 weeks  **Location:** hopsital  **Provider:** NR  **Mode of delivery:** in-person  **Tailoring:** NR  **Modifications:** NR |
| Outcomes | Pain (0-10)  **Follow-up (post-intervention):** immediate term (closest to 2 weeks) |
| Risk of bias | High (refer to Appendix 2 for details) |
| **Yuan 2016[33]** (2011) | |
| Methods | **Study design:** RCT  **Setting:** China (upper middle)  **Needling therapies groups:** 1  **Comparison groups:** 2 |
| Participants | **Number of participants:** 150 (A1: 50, C1: 50, C2: 50)  **Mean age, years (SD):** A1: 47.2 (13.8), C1: 45.5 (13.2), C2: 44.0 (13.1)  **Gender (female)**^†^**:** A1: 52.8%, C1: 70%, C2: 68%  **Chronic LBP type:** nonspecific (primary)  **Mean chronic LBP duration, months (SD):** A1: 12.7 (7.3), C1: 13.5, (7.5) C2: 11.0 (7.8)  **Leg pain:** none |
| Interventions | **Intervention:** needling therapies (A1)  **Rationale:** correct unbalance qi and energy and lead to analgesia  **Materials:** needling therapies needles  **Procedures:** five needling therapies points including Shenshu (BL23), Dachangshu (BL25), Weizhong (BL40), Yaoyangguan (GV3) and Huantiao (GB30) were selected as fixed needling therapies points. Meanwhile, several points were selected for needling therapies treatment from 10 needling therapies points including Wangu (SI4), Daimai (GB26), Yanglingquan (GB34), Zulinqi (GB41), Dicang (ST4), Zusanli (ST36), Mingmen (GV4), Xuanshu (GV5), Shenting (GV24) and Shuigou (GV26) according to the specific condition. Manual stimulation. Needle depth: 5-20mm  **Format:** individual  **Duration:** 20 min, 21 sessions, 6 weeks  **Location:** hospital  **Provider:** NR  **Mode of delivery:** in-person  **Tailoring:** all of the patients were permitted to take NSAIDs for relieving low back pain if necessary  **Modifications:** NR  **Adherence:** NR |
| Comparisons | **Comparisons assessed:**   1. needling therapies (A1) vs. placebo-needling therapies (C1) 2. needling therapies (A1) vs. no treatment (C2)   **Intervention:** placebo-needling therapies (C1)  **Procedure:** therapy by selecting the same 5 needling therapies points as that of needling therapies group and using retractable needles. The course of treatment was also same as that of the needling therapies group.  **Materials:** retractable needles  **Format:** individual  **Duration:** 20 min, 21 sessions, 6 weeks  **Location:** hospital  **Provider:** NR  **Mode of delivery:** in-person  **Tailoring:** all of the patients were permitted to take NSAIDs for relieving low back pain if necessary  **Modifications:** NR  **Intervention:** no treatment (C2)  **Procedure:** n/a  **Materials:** n/a  **Format:** n/a  **Duration:** n/a  **Location:** n/a  **Provider:** n/a  **Mode of delivery:** n/a  **Tailoring:** all of the patients were permitted to take NSAIDs for relieving low back pain if necessary  **Modifications:** n/a |
| Outcomes | Pain (VAS 0-100)  Function (ODI 0-100)  Adverse events (no methods reported)  **Follow-up (post-intervention):** immediate term (closest to 2 weeks), short term (closest to 3 months) |
| Risk of bias | Moderate (refer to Appendix 2 for details) |
| **Zaringhalam 2010[34]** (705) | |
| Methods | **Study design:** RCT  **Setting:** Iran (low income)  **Needling therapies groups:** 2  **Comparison groups:** 2 |
| Participants | **Number of participants:** 84 (A1: 21, A2: 21, C1: 21, C2: 21)  **Mean age, years (SD):** A1: 54.2 (5.4), A2: 54.2 (5.6), C1: 54.3 (4.2), C2: 55.1 (3.3)  **Gender (female)**^†^**:** A1: 0%, A2: 0%, C1: 0%, C2: 0%  **Chronic LBP type:** NR (assumed nonspecific primary)  **Mean chronic LBP duration, years (SD not reported): Years:** A1: 7.1, A2: 6.9, C1: 7; C2: 6.7  **Leg pain:** none |
| Interventions | **Intervention:** electroacupuncture (A1)  **Rationale:** pain relief  **Materials:** disposable, stainless 30-gauge needles  **Procedures:** needles bilaterally in the following acupoints: Shenshu (BL23), Dachangshu (BL25), Panguanshu (BL28), Ciliao (BL32), Kunlun (BL60), Huantiao (GB30) and Yanglingquan (GB34) coupled with electrical stimulation at 4-6 Hz with pulse  duration of 0.5 ms  **Format:** individual  **Duration:** 25 min, 10 sessions, 5 weeks  **Location:** NR  **Provider:** acupuncturist  **Mode of delivery:** in-person  **Tailoring:** NR  **Modifications:** NR  **Adherence:** NR  **Intervention:** electroacupuncture + baclofen (A2)  **Rationale:** pain relief  **Materials:** disposable, stainless 30-gauge needles  **Procedures:** needles bilaterally in the following acupoints: Shenshu (BL23), Dachangshu (BL25), Panguanshu (BL28), Ciliao (BL32), Kunlun (BL60), Huantiao (GB30) and Yanglingquan (GB34) coupled with electrical stimulation at 4-6 Hz with pulse  duration of 0.5 ms. Baclofen administered (30 mg/day)  **Format:** individual  **Duration:** 25 min, 10 sessions, 5 weeks  **Location:** NR  **Provider:** acupuncturist  **Mode of delivery:** in-person  **Tailoring:** NR  **Modifications:** NR  **Adherence:** NR |
| Comparisons | **Comparisons assessed:**   1. electroacupuncture (A1) vs. no treatment/waitlist (C1) 2. electroacupuncture + baclofen (A2) vs. baclofen (C2)   **Intervention:** no treatment/waitlist (C1)  **Procedure:** n/a  **Materials:** n/a  **Format:** n/a  **Duration:** n/a  **Location:** n/a  **Provider:** n/a  **Mode of delivery:** n/a  **Tailoring:** n/a  **Modifications:** n/a  **Intervention:** baclofen (C2)  **Procedure:** orally administered 30 mg/day (15 mg *bid*)  **Materials:** n/a  **Format:** n/a  **Duration:** daily for 5 weeks  **Location:** NR  **Provider:** NR  **Mode of delivery:** n/a  **Tailoring:** NR  **Modifications:** NR |
| Outcomes | Pain (VAS 0-100)  Function (RMDQ 0-24)  **Follow-up (post-intervention):** immediate term (closest to 2 weeks), short term (closest to 3 months) |
| Risk of bias | High (refer to Appendix 2 for details) |
| **Zhai 2019[35]** (1606) | |
| Methods | **Study design:** RCT  **Setting:** China (upper-middle)  **Needling therapies groups:** 1  **Comparison groups:** 1 |
| Participants | **Number of participants:** 101 (A1: 50, C1: 51)  **Mean age, years (SD):** A1: 53.5 (12.2), C1: 52.3 (11.5)  **Gender (female)**^†^**:** A1: 42%, C1: 39.2%  **Chronic LBP type:** lumbar disc herniation  **Mean chronic LBP duration, years (SD):** A1: 5.13, C1: 5.40  **Leg pain:** 100% |
| Interventions | **Intervention:** needling therapies + conventional western medicine and drug cupping (A1)  **Rationale:** Chinese medicine is one of the effective treatments  **Materials:** 116mm needling therapies needles  **Procedures:** deep needling therapies on Jiaji points in addition to intervention received by control group. Manual stimulation  **Format:** individual  **Duration:** 20 min, 40 sessions, 8 weeks  **Location:** hospital  **Provider:** NR  **Mode of delivery:** in-person  **Tailoring:** NR  **Modifications:** NR  **Adherence:** NR |
| Comparisons | **Comparisons assessed:** needling therapies + conventional western medicine and drug cupping (A1) vs. conventional western medicine and drug cupping (C1)  **Intervention:** conventional western medicine and drug cupping (C1)  **Procedure:** conventional western medicine and drug cupping (bamboo cup boiled with Chinese medicines and then applied to pain location)  **Materials:** Chinese medicines  **Format:** individual  **Duration:** 20 minutes, 8 sessions, 2 months  **Location:** hospital  **Provider:** NR  **Mode of delivery:** in-person  **Tailoring:** NR  **Modifications:** NR |
| Outcomes | Pain (VAS 0-10)  Function (ODI 0-100, Japanese Orthopaedic Association Scale 0-29)  Health-related quality of life (WHOQOL 0-100)  **Follow-up (post-intervention):** immediate term (closest to 2 weeks) |
| Risk of bias | High (refer to Appendix 2 for details) |
| **Zhu 2020[36]** (1460) | |
| Methods | **Study design:** RCT  **Setting:** China  **Needling therapies groups:** 1  **Comparison groups:** 1 |
| Participants | **Number of participants:** 82 (A1: 41, C1: 41)  **Mean age, years (SD):** A1: 51.9 (15.6), C1: 51.8 (15.6)  **Gender (female)**^†^**:** A1: 43.9%, C1: 48.8%  **Chronic LBP type:** lumbar disc herniation  **Mean chronic LBP duration, years (SD):** A1: 5.9 (1.3), C1: 5.9 (1.3)  **Leg pain:** 100% |
| Interventions | **Intervention:** needling therapies + manipulation (A1)  **Rationale:** needling therapies is effective for LDH  **Materials:** needling therapies needles, electromagnetic wave therapy device  **Procedures:** needling therapies in addition to manipulation received by control group. Fourteen points: mingmen, yangguan, bilateral zhibian, bilateral fengshi, bilateral weizhong, bilateral yanglingquan, bilateral xuanzhong, bilateral qunlun  **Format:** individual  **Duration:** 30 min, 20 sessions, 4 weeks  **Location:** hospital  **Provider:** NR  **Mode of delivery:** in-person  **Tailoring:** NR  **Modifications:** NR  **Adherence:** NR |
| Comparisons | **Comparisons assessed:** needling therapies + manipulation (A1) vs. manipulation (C1)  **Intervention:** manipulation (C1)  **Procedure:** bone setting manipulation  **Materials:** NR  **Format:** individual  **Duration:** 14 sessions, 4 weeks  **Location:** hospital  **Provider:** NR  **Mode of delivery:** in-person  **Tailoring:** NR  **Modifications:** NR |
| Outcomes | Pain (VAS 0-10)  Function (Japanese Orthopaedic Association Score 0-29)  **Follow-up (post-intervention):** immediate term (closest to 2 weeks) |
| Risk of bias | High (refer to Appendix 2 for details) |
| **Zhu 2021[37]** (1387) | |
| Methods | **Study design:** RCT  **Setting:** China (upper middle)  **Needling therapies groups:** 1  **Comparison groups:** 1 |
| Participants | **Number of participants:** 92 (A1: 46, C1: 46)  **Mean age, years (SD):** A1: 53.4 (6.1), C1: 51.4 (5.8)  **Gender (female)**^†^**:** A1: 47.8%, C1: 43.5  **Chronic LBP type:** nonspecific (primary)  **Mean chronic LBP duration, years (SD):** NR  **Leg pain:** NR |
| Interventions | **Intervention:** warming needle moxibustion + joint loosening therapy (A1)  **Rationale:** needling therapies is effective for chronic low back pain  **Materials:** 50 mm to 100 mm needling therapies needles  **Procedures:** warming- needle moxibustion in addition to joint loosening therapy. Twelve needling therapies points: bilateral Dachangyu, Shenyu, Ciliao, Huantiaoxue, Yanglingquan, ashi point  **Format:** individual  **Duration:** 30 min, 14 sessions, 4 weeks  **Location:** hospital  **Provider:** NR  **Mode of delivery:** in-person  **Tailoring:** NR  **Modifications:** NR  **Adherence:** NR |
| Comparisons | **Comparisons assessed:** warming needle moxibustion + joint loosening therapy (A1) vs. joint loosening therapy (C1)  **Intervention:** joint loosening therapy (C1)  **Procedure:** passive mobilization/manipulation  **Materials:** N/A  **Format:** individual  **Duration:** 20 minutes, 20 sessions, 20 days  **Location:** hospital  **Provider:** NR  **Mode of delivery:** in-person  **Tailoring:** Adjusted based on patients’ pain tolerance  **Modifications:** NR |
| Outcomes | Pain (NRS 0-10)  Function (ODI 0-50)  **Follow-up (post-intervention):** immediate term (closest to 2 weeks) |
| Risk of bias | High (refer to Appendix 4 for details) |

First author last name and year (reference ID)

^†^We used the terms ‘female or male’ to describe gender because these were the terms used by trial authors; however, we recognize that gender is a social construct and sex is a biological construct.

**C1:** Comparison treatment group 1, **A1:** Education or advice treatment group 1, **FABQ-PA:** Fear Avoidance Beliefs Questionnaire Physical Activities subscale, **FABQ-W:** Fear Avoidance Beliefs Questionnaire Work subscale, **LBP:** low back pain, **NPRS:** numeric pain rating scale, **n/a:** not applicable; **NR:** not reported; **ODI:** Oswestry Disability Index, PCS: Pain Catastrophizing Scale, PHI: Physical impairment, QofL: Quality of life, **RCT:** randomized controlled trial, **RMDQ:** Roland-Morris Disability Questionnaire, **SD:** standard deviation, **SF-36:** 36-item Short Form Health Survey, **TSK:** Tampa Scale of Kinesiophobia, **VAS:** Visual Analogue Scale, **WHOQOL-BREF:** World Health Organization Quality of Life abbreviated questionnaire, **WI:** Waddell Disability Index

**Online Resource 3.** List of excluded studies with reasons for exclusion (n=72)

| Reason | Study |
| --- | --- |
| Ineligible publication type  n = 7 | 1. Fuentes, Roselyn Clemente, Organ, Brooke, Creech, Julie, Broszko, Christine M, Nashelsky, Joan. RefID: 549, Acupuncture for low back pain. 2020.[38] 2. Kim, J, Mawla, I, Lee, J, Gerber, J, Chan, S-T, Kim, H, Loggia, M, Edwards, R, Wasan, A, Kong, J, et al. RefID: 1093, Resting state functional brain connectivity predicts clinical improvements in chronic low back pain following acupuncture. 2020.[39] 3. Shojaei, M, Shahsavari, S, Gholami, A. RefID: 964, Comparing two combined treatment method for chronic LBP: conventional physiotherapy with lllt and conventional physiotherapy with dry needling: randomized controlled study. 2017.[40] 4. Spaar A. RefID: 422, Acupuncture treatment for chronic low back pain. 2009.[41] 5. Thomas, K J, MacPherson, H, Ratcliffe, J, Thorpe, L, Brazier, J, Campbell, M, Fitter, M, Roman, M, Walters, S, Nicholl, J P. RefID: 765, Longer term clinical and economic benefits of offering acupuncture care to patients with chronic low back pain.[42] 6. Yu, C, Jia, D, Wan, Y, Jiang, H, Liu, Y, Shang, H, Liang, Y, Li, Y, Cao, H, Liu, C. RefID: 1110, The clinical study of Chinese Tuina versus acupuncture in the treatment of chronic nonspecific low back pain (CNLBP). 2019.[43] 7. RefID: 1124, Efficacy of acupuncture for sciatica: a randomized controlled trial ChiCTR2100044585.[44] |
| Ineligible study design  n = 5 | 1. Carmenaty, I, Montoya, E. RefID: 938, Acupuncture and lumbosacral spine pain. 1991.[45] 2. González, JL, Abd, J, Carmona, A, Carmenaty, I. RefID: 1165, Acupuntura, moxa y ventosa en el tratamiento de la radiculitis lumbosacra crónica Acupuncture, moxibustion and cupping for chronic lumbosacral radiculitis. 1994.[46] 3. González, JL, Martínez, H, López, EM, Carmenaty, I. RefID: 955, Comparative study between acupuncture, laser and diathermy for treatment of chronic lumbosacral spine pain. 1990.[47] 4. Kizhakkeveettil, Anupama, Rose, Kevin A, Kadar, Gena E, Hurwitz, Eric L. RefID: 584, An Exploratory Analysis of Gender as a Potential Modifier of Treatment Effect Among Patients in a Randomized Controlled Trial of Integrative Acupuncture and Spinal Manipulation for Low Back Pain. 2019.[48] 5. Lv J, Liu Y, Wang Z. RefID: 1648, [[Therapeutic effect of mainly comprehensive treatment of electroacupuncture combined with manual fast needle strong stimulation in the treatment of lumbar disc herniation with numbness as its main symptom](https://oversea.cnki.net/kns/Detail?sfield=fn&QueryID=0&CurRec=1&recid=&FileName=HBZY201907023&DbName=CJFDLAST2019&DbCode=CJFD&yx=Y&pr=&URLID=13.1067.R.20190904.0950.046)] (article in Chinese). 2019.[49] |
| Ineligible population  n = 49 | 1. Bao D, Wu Z, Pan F. RefID: 1614, [Clinical study of acupuncture combined with Yishen Huoxue Tang for lumbar disc herniation with kidney deficiency and blood stasis type] (article in Chinese). 2019.[50] 2. Carlsson, C P, Sjolund, B H. RefID: 783, Acupuncture for chronic low back pain: a randomized placebo-controlled study with long-term follow-up. 2001.[51] 3. Chen L., Deng H., Houle T., Zhang Y., Ahmed S., Zhang W., Sullivan S., Opalacz A., Roth S., Filatava E.J., Stabach K., Vo T., Malarick C., Kim H., You Z., Shen S., Mao J. RefID: 136, Comparison between acupuncture therapy and gabapentin for chronic pain: a pilot study. 2021.[52] 4. Chen M.-R., Wang P., Cheng G., Guo X., Wei G.-W., Cheng X.-H. RefID: 225, The warming acupuncture for treatment of sciatica in 30 cases. 2009.[53] 5. Cherkin, Daniel C, Sherman, Karen J, Avins, Andrew L, Erro, Janet H, Ichikawa, Laura, Barlow, William E, Delaney, Kristin, Hawkes, Rene, Hamilton, Luisa, Pressman, Alice, Khalsa, Partap S, Deyo, Richard A. RefID: 746, A randomized trial comparing acupuncture, simulated acupuncture, and usual care for chronic low back pain. 2009.[2] 6. Cherkin, D C, Eisenberg, D, Sherman, K J, Barlow, W, Kaptchuk, T J, Street, J, Deyo, R A. RefID: 786, Randomized trial comparing traditional Chinese medical acupuncture, therapeutic massage, and self-care education for chronic low back pain. 2001.[54] 7. Clark N.G., Hill C.J., Koppenhaver S.L., Massie T., Cleland J.A. RefID: 197, The effects of dry needling to the thoracolumbar junction multifidi on measures of regional and remote flexibility and pain sensitivity: A randomized controlled trial. 2021.[55] 8. Coan R.M., Wong G., Liang Ku S., Chan Y.C., Wang L., Ozer F.T. RefID: 287, The acupuncture treatment of low back pain: A randomized controlled study. 1980.[56] 9. de Castro Moura, C, Iunes, DH, Ruginsk, SG, Salgado Souza, VH, Bacelar de Assis, B, de Cássia Lopes Chaves, E. RefID: 968, Action of ear acupuncture in people with chronic pain in the spinal column: a randomized clinical trial. 2018.[57] 10. Fang F, Yanxia W. RefID: 1658, [Clinical observation on lumbar three needles in the treatment of lumbar disc protrusion and imaging changes] (article in Chinese). 2018.[58] 11. Griswold, D, Gargano, F, Learman, KE. RefID: 960, A randomized clinical trial comparing non-thrust manipulation with segmental and distal dry needling on pain, disability, and rate of recovery for patients with non-specific low back pain. 2019.[59] 12. Guo, Yong-Hong. RefID: 774, [Prolapse of lumbar intervertebral disc treated by needling the side of abdomen and fumigation with Chinese herbs] (article in Chinese). 2003.[60] 13. Heo, In, Shin, Byung-Cheul, Cho, Jae-Heung, Ha, In-Hyuk, Hwang, Eui-Hyoung, Lee, Jun-Hwan, Kim, Koh-Woon, Kim, Me-riong, Jung, So-Young, Kwon, Ojin, Kim, Nam-Kwen, Son, Dong-Wuk, Shin, Kyung-Min. RefID: 75, Multicentre randomised controlled clinical trial of electroacupuncture with usual care for patients with non-acute pain after back surgery. 2021.[61] 14. Hong Q, Liu X, Wang G. RefID: 1348, [Effect of acupuncture combined with medicine on lumbar disc herniation] (article in Chinese). 2021.[62] 15. Hu X.-W., Deng C.-Y., Qiu F.-F., Geng L., Yu X.-J., Tang L.-M., Huang P., Nie X.-F. RefID: 340, Clinical effect of long-snake moxibustion on lumbago of cold-dampness type. 2020.[63] 16. Johnston K., Bonjour T., Powell J., April M.D. RefID: 150, Battlefield Acupuncture Versus Standard Pharmacologic Treatment of Low Back Pain in the Emergency Department: A Randomized Controlled Trial. 2021.[64] 17. Kumnerddee, Wipoo. RefID: 734, Effectiveness comparison between Thai traditional massage and Chinese acupuncture for myofascial back pain in Thai military personnel: a preliminary report. 2009.[65] 18. Lai P, Deng H, Wu J, Chai T. RefID: 1688, [Clinical observation of electrothermic – needling moxibustion treating NLBP of cold – dampness pattern] (article in Chinese). 2019.[66] 19. Lee, Jeungchan, Eun, Seulgi, Kim, Jieun, Lee, Jun-Hwan, Park, Kyungmo. RefID: 625, Differential Influence of Acupuncture Somatosensory and Cognitive/Affective Components on Functional Brain Connectivity and Pain Reduction During Low Back Pain State. 2019.[67] 20. Lehmann, Thomas R, Russell, Daniel W, Spratt, Kevin F, Colby, Hutha, Liu, King Y, Fairchild, Mary Lou, Christensen, Stanley. RefID: 807, Efficacy of electroacupuncture and TENS in the rehabilitation of chronic low back pain patients. 1986.[68] 21. Leibing, Eric, Leonhardt, Urs, Koster, Georg, Goerlitz, Anke, Rosenfeldt, Joerg Andre, Hilgers, Reinhard, Ramadori, Giuliano. RefID: 781, Acupuncture treatment of chronic low-back pain -- a randomized, blinded, placebo-controlled trial with 9-month follow-up. 2002.[69] 22. Li C., Li T., Ma X., Ni C., Wei X., Zhang S. RefID: 151, A randomized clinical study on acupuncture therapy for relieving sciatica caused by lumbar disc herniation. 2021.[70] 23. Li H, Zhang J. RefID: 1656, [Curactive effect observation on electroacupuncture at acupoints of lumbar-abdomen for nonspecific low back pain] (article in Chinese). 2018.[71] 24. Lin, Mu-Lien, Lin, Mu-Hung, Fen, Jun-Jeng, Lin, Wei-Tso, Lin, Chii-Wann, Chen, Po-Quang. RefID: 722, A comparison between pulsed radiofrequency and electro-acupuncture for relieving pain in patients with chronic low back pain. 2010.[72] 25. Loizidis T., Nikodelis T., Bakas E., Kollias I. RefID: 247, The effects of dry needling on pain relief and functional balance in patients with sub-chronic low back pain. 2020.[73] 26. Lu, et al. RefID: 1571, [Therapeutic effects of Zhuifengtougu capsule combined with acupuncture on lumbago] (article in Chinese). 2019.[74] 27. Lu Y, Zhang J, Lin X. RefID: 1657, [Clinical observation on treatment of lumbar intervertebral disc protrusion with Jiaji point combined with salvianolate] (article in Chinese). 2018.[75] 28. Lucy Chen, Hao Deng, Timothy Houle, Yi Zhang, Ahmed, Shihab, Vivian Zhang, Sullivan, Shelly, Opalaze, Arissa, Roth, Sarah, Falacov, Jen, Clair, Kristan St, Vo, Tran, Malarick, Charline, Hyangin Kim, Zerong You, Shiqian Shen, Jianren Mao, Chen, Lucy, Deng, Hao, Houle, Timothy. RefID: 4, A randomized trial to assess the immediate impact of acupuncture on quantitative sensory testing, pain, and functional status. 2019.[76] 29. Lv L, Sux X, Lv Z, Yang Z, Sun X, Ma X. RefID: 1461, [Effect of sputum scupuncture on low back pain in pilots and on patients’ satisfaction and compliance: a multicentre controlled study] (article in Chinese). 2020.[77] 30. Mahmoudzadeh A., Rezaeian Z.S., Karimi A., Dommerholt J. RefID: 407, The effect of dry needling on the radiating pain in subjects with discogenic low-back pain: A randomized control trial. 2016.[78] 31. Molsberger, Albrecht F, Mau, Jochen, Pawelec, Danuta B, Winkler, Janos. RefID: 778, Does acupuncture improve the orthopedic management of chronic low back pain--a randomized, blinded, controlled trial with 3 months follow up. 2002.[17] 32. Qian Z, Song M, Qian J, Ll X, Cui Y. RefID: 1652, [Comparison of effect of electroacupuncture and McKenzie therapy on residual symptoms of patients with lumbar disc herniation after intervertebral foramoscopy] (article in Chinese). 2019.[79] 33. Qin Z., Ding Y., Xu C., Kwong J.S.W., Ji Y., Wu A., Wu J., Liu Z. RefID: 323, Acupuncture vs Noninsertive Sham Acupuncture in Aging Patients with Degenerative Lumbar Spinal Stenosis: A Randomized Controlled Trial. 2020.[80] 34. Quan J. RefID: 1749, [Effect of warm acupuncture combined with massage and hot compress on patients with chronic low back pain due to kidney yang deficiency] (article in Chinese). 2020.[81] 35. Sun, Xu, Ma, Xin-Ping, Lv, Lin-Ya, Yang, Jie, Liu, Hai-Fei, Meng, Qing-Yang, Zheng, Jun, Ma, Xin-Ying. RefID: 515, [Intervention with thumbtack needling on spinal low back pain in Air Force crew: a randomized controlled trial]. 2020.[82] 36. Thomas, M, Lundberg, T. RefID: 798, Importance of modes of acupuncture in the treatment of chronic nociceptive low back pain. 1994.[83] 37. Wang Y., Dai S., Yang A. RefID: 172, Effect evaluation of acupuncture combined with nerve block treatment on patients with lumbar spondylolisthesis. 2021.[84] 38. Wang G, Guo J, Hu J, Zhang F. RefID: 1526, [Clinical study on the treatment of lumbar disc herniation by He’s Santong method of acupuncture] (article in Chinese). 2019.[85] 39. Wang-Price, Sharon, Zafereo, Jason, Couch, Zach, Brizzolara, Kelli, Heins, Taylor, Smith, Lindsey. RefID: 554, Short-term effects of two deep dry needling techniques on pressure pain thresholds and electromyographic amplitude of the lumbosacral multifidus in patients with low back pain - a randomized clinical trial. 2020.[86] 40. White, Peter, Bishop, Felicity L, Prescott, Phil, Scott, Clare, Little, Paul, Lewith, George. RefID: 693, Practice, practitioner, or placebo? A multifactorial, mixed-methods randomized controlled trial of acupuncture. 2012.[87] 41. Xi S, Wang L. RefID: 1391, [Effects of oblique round needle combined with needle-warming moxibustion on serum levels of TXB2 and TNF- α] (article in Chinese). 2020.[88] 42. Yang Y, Wang Y, Zeng Y. RefID: 1653, [Randomized parallel controlled study on the treatment of lumbar intervertebral disc herniation (Fenghan Shibi) with Traditional Chinese Medicine Rehuangbao plus electroacupuncture combined with diclofenac sodium] (article in Chinese). 2018.[89] 43. Yu N, Liu Y, Xiong S, Xu C, Lu J. RefID: 1732, [Observation on therapeutic effect of acupuncture at abdomen acupoints plus tuina for lumbar intervertebral disc herniation] (article in Chinese). 2019.[90] 44. Zhang M, Nie W, Guo Y, Ding Y. RefID: 1213, [Clinical observation of mild to moderate lumbodorsal fasciitis treated with distal acupoints along meridian plus exercising combined with penetration needling on yang meridians of back] (article in Chinese). 2019.[91] 45. Zhang J., Wu Y., Li S., Sun Y. RefID: 383, Pestle needling at Yaoyangguan-Bazhen points for intractable lumbodynia after lumbar disc herniation surgery: A randomized controlled trial. 2019.[92] 46. Zhu Y, Zhang J, Wu Y. RefID: 1669, [Therapeutic observation of Qi-guiding needling plus electroacupuncture for intractable low back pain after lumbar disc herniation surgery and the change of infrared thermal image] (article in Chinese). 2016.[93] 47. Zhuang Z, Wang D. RefID: 1682, [Effect of electroacupuncture at Baihui and Shenmen on pain in lumbar intervertebral disc herniation] (article in Chinese). 2009.[94] 48. Zou, Zhen. RefID: 745, Fifty-two cases of the piriformis syndrome treated by centro-square needling. 2009.[95] |
| Ineligible intervention  n = 2 | 1. Hu M, Jin L, Wang J. RefID: 1562, [Clinical study of decoction for lumbar obstruction and releasing tendon combined with small needle knife in the treatment of senile lumbar disc herniation] (article in Chinese). 2019.[96] 2. Song J, Liu Z. RefID: 1449, [Effect of acupuncture plus kinesiotherapy on analgesia, lumbar function and inflammatory cytokines in patients with lumbar intervertebral disc herniation] (article in Chinese). 2020.[97] |
| Ineligible comparison  n = 10 | 1. Arriaga-Pizano L, Gómez-Jiménez DC, Flores-Mejía LA, et al. Low back pain in athletes can be controlled with acupuncture by a catecholaminergic pathway: clinical trial. 2020.[98] 2. Gang, Wang, Qian, Gao, Jun, Li, Yuling, Tian, Jingshan, Hou. RefID: 993, Impact of Needle Diameter on Long-Term Dry Needling Treatment of Chronic Lumbar Myofascial Pain Syndrome. 2016. [99] 3. Goo, Bonhyuk, Kim, Jung-Hyun, Kim, Eun-Jung, Lee, Hyun-Jong, Kim, Jae-Soo, Nam, Dongwoo, Park, Yeon-Cheol, Kim, Tae-Hun, Baek, Yong-Hyeon, Nam, Sang-Soo, Seo, Byung-Kwan. RefID: 105, Thread embedding acupuncture for herniated intervertebral disc of the lumbar spine: A multicenter, randomized, patient-assessor-blinded, controlled, parallel, clinical trial. 2022.[100] 4. Klassen E., Wiebelitz K.-R., Beer A.-M. RefID: 389, Classical Massage and Acupuncture in Chronic Back Pain - Non-Inferiority Randomised Trial. 2019.[101] 5. Leite, Paula M S, Mendonca, Andreza R C, Maciel, Leonardo Y S, Poderoso-Neto, Mauricio L, Araujo, Carla C A, Gois, Hilda C J, Souza, Jersica H S, DeSantana, Josimari M. RefID: 631, Does Electroacupuncture Treatment Reduce Pain and Change Quantitative Sensory Testing Responses in Patients with Chronic Nonspecific Low Back Pain? A Randomized Controlled Clinical Trial. 2018.[102] 6. Luo, Yong, Yang, Min, Liu, Tao, Zhong, Xiaolong, Tang, Wen, Guo, Mingyang, Hu, Yonghe. RefID: 562, Effect of hand-ear acupuncture on chronic low-back pain: a randomized controlled trial. 2019.[103] 7. Sator-Katzenschlager, S. M., Scharbert, G., Kozek-Langenecker, S. A., Szeles, J. C., Finster, G., Schiesser, A. W., Heinze, G., Kress, H. G. RefID: 2008, The short- and long-term benefit in chronic low back pain through adjuvant electrical versus manual auricular acupuncture. 2004.[104] 8. Tsui, Margaret L K, Cheing, Gladys L Y. RefID: 768, The effectiveness of electroacupuncture versus electrical heat acupuncture in the management of chronic low-back pain. 2004.[105] 9. Wang, Xu, Zhu, Jun-Song. RefID: 535, [Effect of Cangguitanxue acupuncture combined with suspension exercise therapy on chronic low back pain] (article in Chinese). 2020.[106] 10. Yeh B.-Y., Liu G.-H., Lee T.-Y., Wong A.M.-K., Chang H.-H., Chen Y.-S. RefID: 268, Efficacy of Electronic Acupuncture Shoes for Chronic Low Back Pain: Double-Blinded Randomized Controlled Trial. 2020.[107] |
| Ineligible outcome  n =1 | 1. Lv et al. RefID: 1663, [Scalp acupuncture combined with electroacupuncture in treatment of discogenic low back pain: clinical trial] (article in Chinese). 2017.[108] |

**Online Resource 4.** Risk of bias assessment for the included trials (n=37)

| Trial | Random allocation | Concealed allocation | Participant blinding | Provider blinding | Assessor blinding | Drop-outs | Intention-to-treat analysis | Selective reporting | Similar groups (baseline) | Co-interventions | Compliance | Timing of outcomes | Other biases | Overall ROB Rating |
| --- | --- | --- | --- | --- | --- | --- | --- | --- | --- | --- | --- | --- | --- | --- |
|  | Selection bias | | Performance bias | | Detection bias | Attrition bias | | Reporting bias | Selection bias | Other biases | | | |
| Brinkhaus 2006 (2000) | 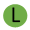 | 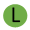 | 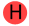 | 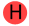 | 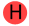 | 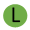 | 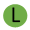 | 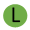 | 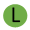 | 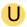 | 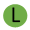 | 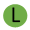 | 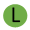 | High |
| Cherkin 2009 (2001) | 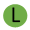 | 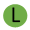 | 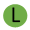 | 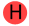 | 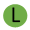 | 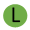 | 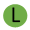 | 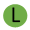 | 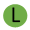 | 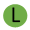 | 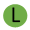 | 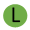 | 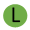 | Unclear |
| Cho 2013 (2002) | 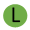 | 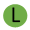 | 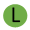 | 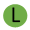 | 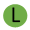 | 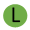 | 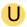 | 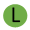 | 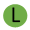 | 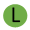 | 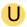 | 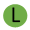 | 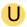 | Unclear |
| de Castro Moura 2019 (32) | 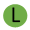 | 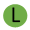 | 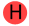 | 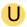 | 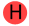 | 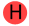 | 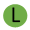 | 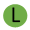 | 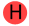 | 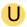 | 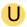 | 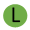 | 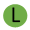 | High |
| de Castro Moura 2019 (1167) | 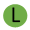 | 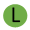 | 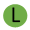 | 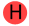 | 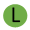 | 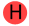 | 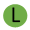 | 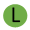 | 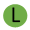 | 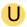 | 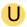 | 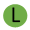 | 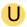 | High |
| Depaoli 2021 (1152) | 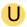 | 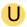 | 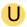 | 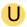 | 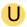 | 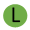 | 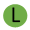 | 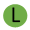 | 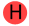 | 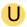 | 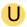 | 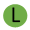 | 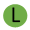 | High |
| Haake 2007 (2003) | 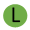 | 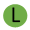 | 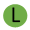 | 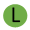 | 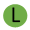 | 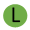 | 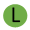 | 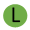 | 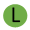 | 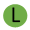 | 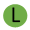 | 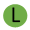 | 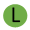 | Low |
| Huang 2019 (52) | 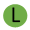 | 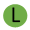 | 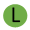 | 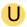 | 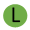 | 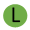 | 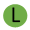 | 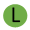 | 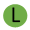 |  |  |  |  | Low |
| Kerr 2003 (2004) |  |  |  |  |  |  |  |  |  |  |  |  |  | High |
| Kim 2020 (542) |  |  |  |  |  |  |  |  |  |  |  |  |  | High |
| Kong 2020 (41) |  |  |  |  |  |  |  |  |  |  |  |  |  | High |
| Koppenhaver 2021 (166) |  |  |  |  |  |  |  |  |  |  |  |  |  | High |
| Li 2019 (565) |  |  |  |  |  |  |  |  |  |  |  |  |  | High |
| Li 2022 (116) |  |  |  |  |  |  |  |  |  |  |  |  |  | High |
| Martin-Corrales 2020 (23) |  |  |  |  |  |  |  |  |  |  |  |  |  | Unclear |
| Meng 2003 (2012) |  |  |  |  |  |  |  |  |  |  |  |  |  | High |
| Molsberger 2002 (2007) |  |  |  |  |  |  |  |  |  |  |  |  |  | High |
| Moslemi 2020 (305) |  |  |  |  |  |  |  |  |  |  |  |  |  | High |
| Pan 2019 (1528) |  |  |  |  |  |  |  |  |  |  |  |  |  | High |
| Ran 2021 (1335) |  |  |  |  |  |  |  |  |  |  |  |  |  | High |
| Ren 2021 (1327) |  |  |  |  |  |  |  |  |  |  |  |  |  | High |
| Shi 2021 (1376) |  |  |  |  |  |  |  |  |  |  |  |  |  | High |
| Sung 2020 (296) |  |  |  |  |  |  |  |  |  |  |  |  |  | Unclear |
| Tabatabaiee 2019 (108) |  |  |  |  |  |  |  |  |  |  |  |  |  | High |
| Ushinohama 2016 (2009) |  |  |  |  |  |  |  |  |  |  |  |  |  | High |
| Wang 2020 (1429) |  |  |  |  |  |  |  |  |  |  |  |  |  | High |
| Wang 2020 (1456) |  |  |  |  |  |  |  |  |  |  |  |  |  | High |
| Weib 2013 (1153) |  |  |  |  |  |  |  |  |  |  |  |  |  | High |
| Witt 2006 (2010) |  |  |  |  |  |  |  |  |  |  |  |  |  | High |
| Yeung 2003 (2013) |  |  |  |  |  |  |  |  |  |  |  |  |  | High |
| Yu 2020 (183) |  |  |  |  |  |  |  |  |  |  |  |  |  | High |
| Yu 2021 (187) |  |  |  |  |  |  |  |  |  |  |  |  |  | High |
| Yuan 2016 (2011) |  |  |  |  |  |  |  |  |  |  |  |  |  | Unclear |
| Zaringhalam 2010 (705) |  |  |  |  |  |  |  |  |  |  |  |  |  | High |
| Zhai 2019 (1606) |  |  |  |  |  |  |  |  |  |  |  |  |  | High |
| Zhu 2020 (1460) |  |  |  |  |  |  |  |  |  |  |  |  |  | High |
| Zhu 2021 (1387) |  |  |  |  |  |  |  |  |  |  |  |  |  | High |

Risk of bias assessment conducted using ROB1 criteria.

First author last name and year (reference ID number).

H=high risk of bias, L=low risk of bias, ROB=risk of bias, U=Unclear

Risk of bias graph: review authors’ judgements about each risk of bias item presented as percentages across all included trials

**Online Resource 5.** GRADE evidence profile tables

GRADE evidence profile 1 table: ***What are the benefits and harms of needling therapies in the management of community-dwelling adults (including older adults aged 60 years and over) with chronic primary low back pain (with or without leg pain) compared to sham?***

| **Certainty assessment** | | | | | | | **№ of patients** | | **Effect** | | **Certainty** | **Importance** |
| --- | --- | --- | --- | --- | --- | --- | --- | --- | --- | --- | --- | --- |
| **№ of studies** | **Study design** | **Risk of bias** | **Inconsistency** | **Indirectness** | **Imprecision** | **Other considerations** | **Needling therapies** | **Sham** | **Relative (95% CI)** | **Absolute (95% CI)** |
| **ALL ADULTS** | | | | | | | | | | | | |
| **Pain (follow-up: closest to 2 weeks; assessed with: VAS, NRS, Von Korff Pain Scale; benefit indicated by lower values; Scale from: 0 to 10)** | | | | | | | | | | | | |
| 7^1,2,3,4,5,6,7,a,b^ | randomised trials | very serious^c^ | not serious^d^ | not serious^e^ | not serious^f^ | none | 581 | 582 | - | MD **0.41 lower** (0.72 lower to 0.1 lower) | ⨁⨁◯◯ Low | CRITICAL |
| **Pain in people without leg pain (follow-up: closest to 2 weeks; assessed with: VAS, NRS; benefit indicated by lower values; Scale from: 0 to 10)** | | | | | | | | | | | | |
| 3^1,3,5,g^ | randomised trials | very serious^c^ | very serious^h^ | not serious^e^ | serious^i^ | none | 138 | 138 | - | MD **0.41 lower** (1.31 lower to 0.49 higher) | ⨁◯◯◯ Very low | CRITICAL |
| **Pain in people with unclassified presence of leg pain (follow-up: closest to 2 weeks; assessed with: VAS, Von Korff Pain Scale; benefit indicated by lower values; Scale from: 0 to 10)** | | | | | | | | | | | | |
| 4^2,4,6,7,a^ | randomised trials | serious^j^ | not serious^k^ | not serious^e^ | not serious^f^ | none | 443 | 444 | - | MD **0.42 lower** (0.75 lower to 0.09 lower) | ⨁⨁⨁◯ Moderate | CRITICAL |
| **Pain in people with radicular leg pain (follow-up: closest to 2 weeks; assessed with: VAS, 0-100; benefit indicated by lower values)** | | | | | | | | | | | | |
| 1^8,l,m,n^ | randomised trials | not serious^o^ | not serious^p^ | serious^q^ | very serious^r^ | none | Between-group MD (95% CI) of within-group MDs: -6.85 (-16.82 to 3.11) (46 participants total). | | | | ⨁◯◯◯ Very low | CRITICAL |
|  | | | | | | | | | | | | |
| **Pain stratified by gender (follow-up: closest to 2 weeks)** | | | | | | | | | | | | |
| 0 |  |  |  |  |  |  |  |  |  |  |  | CRITICAL |
| **Pain stratified by race/ethnicity (follow-up: closest to 2 weeks)** | | | | | | | | | | | | |
| 0 |  |  |  |  |  |  |  |  |  |  |  | CRITICAL |
| **Pain in low to lower-middle income countries (follow-up: closest to 2 weeks)** | | | | | | | | | | | | |
| 0 |  |  |  |  |  |  |  |  |  |  |  | CRITICAL |
| **Pain in people treated with needling therapies type TCM (follow-up: closest to 2 weeks; assessed with: VAS, Von Korff Pain Scale; benefit indicated by lower values; Scale from: 0 to 10)** | | | | | | | | | | | | |
| 5^1,2,3,6,7,a,b^ | randomised trials | serious^j^ | not serious^s^ | not serious^e^ | not serious^f^ | none | 528 | 529 | - | MD **0.46 lower** (0.87 lower to 0.06 lower) | ⨁⨁⨁◯ Moderate | CRITICAL |
| **Pain in people treated with needling therapies type myofascial (follow-up: closest to 2 weeks; assessed with: VAS, NRS; benefit indicated by lower values; Scale from: 0 to 10)** | | | | | | | | | | | | |
| 2^4,5^ | randomised trials | very serious^t^ | not serious^u^ | not serious^e^ | very serious^r^ | none | 53 | 53 | - | MD **0.3 lower** (1.06 lower to 0.45 higher) | ⨁◯◯◯ Very low | CRITICAL |
| **Pain in people treated with needling therapies with manual stimulation (follow-up: closest to 2 weeks; assessed with: VAS, NRS; benefit indicated by lower values; Scale from: 0 to 10)** | | | | | | | | | | | | |
| 5^1,2,3,5,6,a,v^ | randomised trials | very serious^t^ | not serious^w^ | not serious^e^ | serious^i^ | none | 188 | 184 | - | MD **0.43 lower** (1.01 lower to 0.14 higher) | ⨁◯◯◯ Very low | CRITICAL |
|  | | | | | | | | | | | | |
|  | | | | | | | | | | | | |
| **Pain in people treated with needling therapies without stimulation (follow-up: closest to 2 weeks; assessed with: VAS, Von Korff Pain Scale; benefit indicated by lower values; Scale from: 0 to 10)** | | | | | | | | | | | | |
| 2^4,7^ | randomised trials | not serious^x^ | not serious^k^ | not serious^e^ | not serious^f^ | none | 393 | 398 | - | MD **0.4 lower** (0.75 lower to 0.06 lower) | ⨁⨁⨁⨁ High | CRITICAL |
| **Pain after removing high risk of bias studies (follow-up: closest to 2 weeks; assessed with: VAS, Von Korff Pain Scale; benefit indicated by lower values; Scale from: 0 to 10)** | | | | | | | | | | | | |
| 3^1,4,7,v^ | randomised trials | not serious^x^ | serious^y^ | not serious^e^ | serious^z^ | none | 443 | 448 | - | MD **0.68 lower** (1.26 lower to 0.1 lower) | ⨁⨁◯◯ Low | CRITICAL |
| **Pain (follow-up: closest to 3 months; assessed with: VAS, NRS, Von Korff Pain Scale; benefit indicated by lower values; Scale from: 0 to 10)** | | | | | | | | | | | | |
| 9^1,3,4,7,9,10,11,12,13,aa,ab,ac^ | randomised trials | very serious^c^ | very serious^ad^ | not serious^e^ | not serious^ae^ | none | 1044 | 847 | - | MD **0.42 lower** (0.88 lower to 0.05 higher) | ⨁◯◯◯ Very low | CRITICAL |
| **Pain in people without leg pain (follow-up: closest to 3 months; assessed with: VAS, NRS; benefit indicated by lower values; Scale from: 0 to 10)** | | | | | | | | | | | | |
| 4^1,3,9,13,ab,af^ | randomised trials | very serious^t^ | not serious^k^ | not serious^e^ | not serious^ae^ | none | 255 | 194 | - | MD **0.38 lower** (0.86 lower to 0.1 higher) | ⨁⨁◯◯ Low | CRITICAL |
| **Pain in people with radicular leg pain (follow-up: closest to 3 months; assessed with: VAS, 0-100; benefit indicated by lower values)** | | | | | | | | | | | | |
| 1^8,l,m,n^ | randomised trials | not serious^o^ | not serious^p^ | serious^q^ | very serious^r^ | none | Between-group MD (95% CI) of within-group MDs: -6.06 (-18.50 to 6.38) (46 participants total) | | | | ⨁◯◯◯ Very low | CRITICAL |
|  | | | | | | | | | | | | |
| **Pain in people with and without leg pain (follow-up: closest to 3 months; assessed with: NRS; benefit indicated by lower values; Scale from: 0 to 10)** | | | | | | | | | | | | |
| 1^10,aa^ | randomised trials | very serious^t^ | not serious^p^ | serious^q^ | not serious^ae^ | none | 299 | 159 | - | MD **0.35 higher** (0.13 lower to 0.83 higher) | ⨁◯◯◯ Very low | CRITICAL |
| **Pain in people with unclassified presence of leg pain (follow-up: closest to 3 months; assessed with: VAS, BPI, Von Korff Pain Scale; benefit indicated by lower values; Scale from: 0 to 10)** | | | | | | | | | | | | |
| 4^4,7,11,12^ | randomised trials | very serious^c^ | very serious^ag^ | not serious^e^ | serious^ah^ | none | 490 | 494 | - | MD **0.96 lower** (1.81 lower to 0.12 lower) | ⨁◯◯◯ Very low | CRITICAL |
| **Pain stratified by gender (follow-up: closest to 3 months)** | | | | | | | | | | | | |
| 0 |  |  |  |  |  |  |  |  |  |  |  | CRITICAL |
| **Pain stratified by race/ethnicity (follow-up: closest to 3 months)** | | | | | | | | | | | | |
| 0 |  |  |  |  |  |  |  |  |  |  |  | CRITICAL |
| **Pain in people from low to lower-middle income countries (follow-up: closest to 3 months)** | | | | | | | | | | | | |
| 0 |  |  |  |  |  |  |  |  |  |  |  | CRITICAL |
| **Pain in people treated with needling therapies type TCM (follow-up: closest to 3 months; assessed with: NRS, VAS, BPI, Von Korff Pain Scale; benefit indicated by lower values; Scale from: 0 to 10)** | | | | | | | | | | | | |
| 7^1,3,7,10,11,12,13,ai^ | randomised trials | very serious^c^ | serious^aj^ | not serious^e^ | not serious^ae^ | none | 881 | 754 | - | MD **0.17 lower** (0.57 lower to 0.22 higher) | ⨁◯◯◯ Very low | CRITICAL |
|  | | | | | | | | | | | | |
|  | | | | | | | | | | | | |
| **Pain in people treated with needling therapies type myofascial (follow-up: closest to 3 months; assessed with: VAS; benefit indicated by lower values; Scale from: 0 to 10)** | | | | | | | | | | | | |
| 1^4^ | randomised trials | very serious^t^ | not serious^p^ | serious^q^ | very serious^r^ | none | 23 | 23 | - | MD **1.96 lower** (2.79 lower to 1.13 lower) | ⨁◯◯◯ Very low | CRITICAL |
| **Pain in people treated with needling therapies type mixed (TCM, myofascial) (follow-up: closest to 3 months; assessed with: VAS; benefit indicated by lower values; Scale from: 0 to 10)** | | | | | | | | | | | | |
| 1^9,af^ | randomised trials | very serious^t^ | not serious^p^ | serious^q^ | serious^i^ | none | 140 | 70 | - | MD **0.92 lower** (1.76 lower to 0.08 lower) | ⨁◯◯◯ Very low | CRITICAL |
| **Pain in people treated with needling therapies with electrical stimulation (follow-up: closest to 3 months; assessed with: PROMIS, 0-100; benefit indicated by lower values)** | | | | | | | | | | | | |
| 1^14^ | randomised trials | very serious^t^ | not serious^p^ | serious | very serious^r^ | none | Between-group MD (95% CI) of within-group MDs: -2.09 (-4.27 to 0.09) (121 participants total) | | | | ⨁◯◯◯ Very low | CRITICAL |
| **Pain in people treated with needling therapies with manual stimulation (follow-up: closest to 3 months; assessed with: VAS, NRS; benefit indicated by lower values; Scale from: 0 to 10)** | | | | | | | | | | | | |
| 5^1,3,9,11,13,ab,ai^ | randomised trials | very serious^t^ | not serious^ak^ | not serious^e^ | serious^ah^ | none | 312 | 253 | - | MD **0.57 lower** (1.08 lower to 0.06 lower) | ⨁◯◯◯ Very low | CRITICAL |
| **Pain in people treated with needling therapies without stimulation (follow-up: closest to 3 months; assessed with: VAS, BPI, Von Korff Pain Scale; benefit indicated by lower values; Scale from: 0 to 10)** | | | | | | | | | | | | |
| 3^4,7,12^ | randomised trials | very serious^c^ | very serious^al^ | not serious^e^ | serious^z^ | none | 433 | 435 | - | MD **0.83 lower** (2.01 lower to 0.34 higher) | ⨁◯◯◯ Very low | CRITICAL |
|  | | | | | | | | | | | | |
| **Pain in people treated with needling therapies (stimulation not reported) (follow-up: closest to 3 months; assessed with: NRS; benefit indicated by lower values; Scale from: 0 to 10)** | | | | | | | | | | | | |
| 1^10,aa^ | randomised trials | very serious^t^ | not serious^p^ | not serious | not serious^ae^ | none | 299 | 159 | - | MD **0.35 higher** (0.13 lower to 0.83 higher) | ⨁⨁◯◯ Low | CRITICAL |
| **Pain after removing high risk of bias studies (follow-up: closest to 3 months; assessed with: VAS, NRS, Von Korff Pain Scale; benefit indicated by lower values; Scale from: 0 to 10)** | | | | | | | | | | | | |
| 5^1,4,7,10,11,aa,ai^ | randomised trials | very serious^am^ | very serious^an^ | not serious^e^ | serious^z^ | none | 802 | 667 | - | MD **0.55 lower** (1.21 lower to 0.1 higher) | ⨁◯◯◯ Very low | CRITICAL |
| **Pain (follow-up: closest to 6 months; assessed with: VAS, NRS, Von Korff Pain Scale; benefit indicated by lower values; Scale from: 0 to 10)** | | | | | | | | | | | | |
| 4^7,9,10,11,aa,ao^ | randomised trials | very serious^c^ | not serious^ap^ | not serious^e^ | not serious^ae^ | none | 859 | 658 | - | MD **0.21 lower** (0.58 lower to 0.16 higher) | ⨁⨁◯◯ Low | CRITICAL |
| **Pain in people with radicular leg pain (follow-up: closest to 6 months; assessed with: VAS, 0-100; benefit indicated by lower values)** | | | | | | | | | | | | |
| 1^8,l,m,n^ | randomised trials | not serious^o^ | not serious^p^ | serious^q^ | very serious^r^ | none | Between-group MD (95% CI) of within-group MDs: -7.01 (-17.50 to 3.48) (46 participants total) | | | | ⨁◯◯◯ Very low | CRITICAL |
| **Pain in people without leg pain (follow-up: closest to 6 months; assessed with: VAS; benefit indicated by lower values; Scale from: 0 to 10)** | | | | | | | | | | | | |
| 1^9^ | randomised trials | very serious^t^ | not serious^p^ | serious^q^ | serious^i^ | none | 140 | 70 | - | MD **0.37 lower** (1.23 lower to 0.49 higher) | ⨁◯◯◯ Very low | CRITICAL |
|  | | | | | | | | | | | | |
| **Pain in people with and without leg pain (follow-up: closest to 6 months; assessed with: NRS; benefit indicated by lower values; Scale from: 0 to 10)** | | | | | | | | | | | | |
| 1^10,aa^ | randomised trials | very serious^t^ | not serious^p^ | serious^q^ | not serious^ae^ | none | 285 | 153 | - | MD **0.25 higher** (0.27 lower to 0.77 higher) | ⨁◯◯◯ Very low | CRITICAL |
| **Pain in people with unclassified presence of leg pain (follow-up: closest to 6 months; assessed with: VAS, Von Korff Pain Scale; benefit indicated by lower values; Scale from: 0 to 10)** | | | | | | | | | | | | |
| 2^7,11^ | randomised trials | not serious^x^ | not serious^k^ | not serious^e^ | not serious^f^ | none | 434 | 435 | - | MD **0.51 lower** (0.92 lower to 0.1 lower) | ⨁⨁⨁⨁ High | CRITICAL |
| **Pain stratified by gender (follow-up: closest to 6 months)** | | | | | | | | | | | | |
| 0 |  |  |  |  |  |  |  |  |  |  |  | CRITICAL |
| **Pain stratified by race/ethnicity (follow-up: closest to 6 months)** | | | | | | | | | | | | |
| 0 |  |  |  |  |  |  |  |  |  |  |  | CRITICAL |
| **Pain in people in low to lower-middle income countries (follow-up: closest to 6 months)** | | | | | | | | | | | | |
| 0 |  |  |  |  |  |  |  |  |  |  |  | CRITICAL |
| **Pain in people treated with needling therapies type TCM (follow-up: closest to 6 months; assessed with: VAS, NRS, Von Korff Pain Scale; benefit indicated by lower values; Scale from: 0 to 10)** | | | | | | | | | | | | |
| 3^7,10,11,aa,ao^ | randomised trials | very serious^c^ | serious^aq^ | not serious^e^ | not serious^ae^ | none | 719 | 588 | - | MD **0.18 lower** (0.63 lower to 0.28 higher) | ⨁◯◯◯ Very low | CRITICAL |
|  | | | | | | | | | | | | |
|  | | | | | | | | | | | | |
|  | | | | | | | | | | | | |
| **Pain in people treated with needling therapies mixed type (TCM, myofascial) (follow-up: closest to 6 months; assessed with: VAS; benefit indicated by lower values; Scale from: 0 to 10)** | | | | | | | | | | | | |
| 1^9^ | randomised trials | very serious^t^ | not serious^p^ | serious^q^ | serious^i^ | none | 140 | 70 | - | MD **0.37 lower** (1.23 lower to 0.49 higher) | ⨁◯◯◯ Very low | CRITICAL |
| **Pain in people treated with needling therapies with manual stimulation (follow-up: closest to 6 months; assessed with: VAS; benefit indicated by lower values; Scale from: 0 to 10)** | | | | | | | | | | | | |
| 2^9,11,ao^ | randomised trials | very serious^t^ | not serious^k^ | not serious^e^ | serious^i^ | none | 197 | 129 | - | MD **0.54 lower** (1.17 lower to 0.08 higher) | ⨁◯◯◯ Very low | CRITICAL |
| **Pain in people treated with needling therapies without stimulation (follow-up: closest to 6 months; assessed with: Von Korff Pain Scale; benefit indicated by lower values; Scale from: 0 to 10)** | | | | | | | | | | | | |
| 1^7^ | randomised trials | not serious^o^ | not serious^p^ | serious^q^ | not serious^ae^ | none | 377 | 376 | - | MD **0.45 lower** (0.91 lower to 0.01 higher) | ⨁⨁⨁◯ Moderate | CRITICAL |
| **Pain in people treated with needling therapies (stimulation not reported) (follow-up: closest to 6 months; assessed with: NRS; benefit indicated by lower values; Scale from: 0 to 10)** | | | | | | | | | | | | |
| 1^10,aa^ | randomised trials | very serious^t^ | not serious^p^ | serious^q^ | not serious^ae^ | none | 285 | 153 | - | MD **0.25 higher** (0.27 lower to 0.77 higher) | ⨁◯◯◯ Very low | CRITICAL |
|  | | | | | | | | | | | | |
|  | | | | | | | | | | | | |
|  | | | | | | | | | | | | |
| **Pain in people after removing high risk of bias studies (follow-up: closest to 6 months; assessed with: VAS, NRS, Von Korff Pain Scale; benefit indicated by lower values; Scale from: 0 to 10)** | | | | | | | | | | | | |
| 3^7,10,11,aa,ao^ | randomised trials | very serious^am^ | not serious^aq^ | not serious^e^ | not serious^ae^ | none | 719 | 588 | - | MD **0.18 lower** (0.63 lower to 0.28 higher) | ⨁⨁◯◯ Low | CRITICAL |
| **Pain (follow-up: closest to 12 months; assessed with: VAS, NRS; benefit indicated by lower values; Scale from: 0 to 10)** | | | | | | | | | | | | |
| 2^9,10,aa^ | randomised trials | very serious^t^ | not serious^ar^ | not serious^e^ | not serious^ae^ | none | 428 | 222 | - | MD **0.02 lower** (0.51 lower to 0.47 higher) | ⨁⨁◯◯ Low | CRITICAL |
| **Pain in people without leg pain (follow-up: closest to 12 months; assessed with: VAS; benefit indicated by lower values; Scale from: 0 to 10)** | | | | | | | | | | | | |
| 1^9^ | randomised trials | very serious^t^ | not serious^p^ | serious^q^ | serious^i^ | none | 140 | 70 | - | MD **0.57 lower** (1.43 lower to 0.29 higher) | ⨁◯◯◯ Very low | CRITICAL |
| **Pain in people with and without leg pain (follow-up: closest to 12 months; assessed with: NRS; benefit indicated by lower values; Scale from: 0 to 10)** | | | | | | | | | | | | |
| 1^10,aa^ | randomised trials | very serious^t^ | not serious^p^ | serious^q^ | not serious^ae^ | none | 288 | 152 | - | MD **0.2 higher** (0.33 lower to 0.73 higher) | ⨁◯◯◯ Very low | CRITICAL |
| **Pain stratified by gender (follow-up: closest to 12 months)** | | | | | | | | | | | | |
| 0 |  |  |  |  |  |  |  |  |  |  |  | CRITICAL |
|  | | | | | | | | | | | | |
| **Pain stratified by race/ethnicity (follow-up: closest to 12 months)** | | | | | | | | | | | | |
| 0 |  |  |  |  |  |  |  |  |  |  |  | CRITICAL |
| **Pain in people in low to lower-middle income countries (follow-up: closest to 12 months)** | | | | | | | | | | | | |
| 0 |  |  |  |  |  |  |  |  |  |  |  | CRITICAL |
| **Pain in people treated with needling therapies type TCM (follow-up: closest to 12 months; assessed with: VAS; benefit indicated by lower values; Scale from: 0 to 10)** | | | | | | | | | | | | |
| 1^10,aa^ | randomised trials | very serious^t^ | not serious^p^ | serious^q^ | not serious^ae^ | none | 288 | 152 | - | MD **0.2 higher** (0.33 lower to 0.73 higher) | ⨁◯◯◯ Very low | CRITICAL |
| **Pain in people treated with needling therapies type mixed (TCM, myofascial) (follow-up: closest to 12 months; assessed with: VAS; benefit indicated by lower values; Scale from: 0 to 10)** | | | | | | | | | | | | |
| 1^9^ | randomised trials | very serious^t^ | not serious^p^ | serious^q^ | serious^i^ | none | 140 | 70 | - | MD **0.57 lower** (1.43 lower to 0.29 higher) | ⨁◯◯◯ Very low | CRITICAL |
| **Pain in people treated with needling therapies with manual stimulation (follow-up: closest to 12 months; assessed with: VAS; benefit indicated by lower values; Scale from: 0 to 10)** | | | | | | | | | | | | |
| 1^9^ | randomised trials | very serious^t^ | not serious^p^ | serious^q^ | serious^i^ | none | 140 | 70 | - | MD **0.57 lower** (1.43 lower to 0.29 higher) | ⨁◯◯◯ Very low | CRITICAL |
|  | | | | | | | | | | | | |
|  | | | | | | | | | | | | |
|  | | | | | | | | | | | | |
|  | | | | | | | | | | | | |
| **Pain in people treated with needling therapies (stimulation not reported) (follow-up: closest to 12 months; assessed with: NRS; benefit indicated by lower values; Scale from: 0 to 10)** | | | | | | | | | | | | |
| 1^10,aa^ | randomised trials | very serious^t^ | not serious^p^ | serious^q^ | not serious^ae^ | none | 288 | 152 | - | MD **0.2 higher** (0.33 lower to 0.73 higher) | ⨁◯◯◯ Very low | CRITICAL |
| **Pain after removing high risk of bias studies (follow-up: closest to 12 months; assessed with: NRS; benefit indicated by lower values; Scale from: 0 to 10)** | | | | | | | | | | | | |
| 1^10,aa^ | randomised trials | very serious^t^ | not serious^p^ | serious^q^ | not serious^ae^ | none | 288 | 152 | - | MD **0.2 higher** (0.33 lower to 0.73 higher) | ⨁◯◯◯ Very low | CRITICAL |
| **Function (follow-up: closest to 2 weeks; assessed with: RMDQ, ODI, Hannover; benefit indicated by lower values)** | | | | | | | | | | | | |
| 4^1,4,5,7,as^ | randomised trials | very serious^c^ | serious^at^ | not serious^e^ | serious^au^ | none | 478 | 473 | - | SMD **0.22 lower** (0.54 lower to 0.11 higher) | ⨁◯◯◯ Very low | CRITICAL |
| **Function in people without leg pain (follow-up: closest to 2 weeks; assessed with: ODI, Hannover; benefit indicated by lower values)** | | | | | | | | | | | | |
| 2^1,5,af^ | randomised trials | very serious^t^ | serious^aj^ | not serious^e^ | very serious^r^ | none | 80 | 80 | - | SMD **0.48 lower** (0.92 lower to 0.05 lower) | ⨁◯◯◯ Very low | CRITICAL |
|  | | | | | | | | | | | | |
|  | | | | | | | | | | | | |
|  | | | | | | | | | | | | |
| **Function in people with unclassified presence of leg pain (follow-up: closest to 2 weeks; assessed with: RMDQ, Hannover; benefit indicated by lower values)** | | | | | | | | | | | | |
| 2^4,7^ | randomised trials | not serious^x^ | serious^av^ | not serious^e^ | very serious^aw^ | none | 398 | 393 | - | SMD **0.03 lower** (0.37 lower to 0.31 higher) | ⨁◯◯◯ Very low | CRITICAL |
| **Function in people with radicular leg pain (follow-up: closest to 2 weeks; assessed with: ODI, 0-100; benefit indicated by lower values)** | | | | | | | | | | | | |
| 1^8,l,m,n^ | randomised trials | not serious^o^ | not serious^p^ | serious^q^ | very serious^r^ | none | Between-group MD (95% CI) of within-group MDs: -4.52 (-13.05 to 4.01) (46 participants total) | | | | ⨁◯◯◯ Very low | CRITICAL |
| **Function stratified by gender (follow-up: closest to 2 weeks)** | | | | | | | | | | | | |
| 0 |  |  |  |  |  |  |  |  |  |  |  | CRITICAL |
| **Function stratified by race/ethnicity (follow-up: closest to 2 weeks)** | | | | | | | | | | | | |
| 0 |  |  |  |  |  |  |  |  |  |  |  | CRITICAL |
| **Function in people in low to lower-middle countries (follow-up: closest to 2 weeks)** | | | | | | | | | | | | |
| 0 |  |  |  |  |  |  |  |  |  |  |  | CRITICAL |
| **Function in people treated with needling therapies type TCM (follow-up: closest to 2 weeks; assessed with: ODI, Hannover)** | | | | | | | | | | | | |
| 2^1,7,ax^ | randomised trials | not serious^x^ | very serious^ay^ | not serious^e^ | serious^au^ | none | 425 | 429 | - | SMD **0.37 lower** (0.91 lower to 0.17 higher) | ⨁◯◯◯ Very low | CRITICAL |
| **Function in people treated with needling therapies type myofascial (follow-up: closest to 2 weeks; assessed with: RMDQ, ODI)** | | | | | | | | | | | | |
| 2^4,5^ | randomised trials | very serious^t^ | not serious^az^ | not serious^e^ | very serious^r^ | none | 53 | 53 | - | SMD **0**  (0.5 lower to 0.5 higher) | ⨁◯◯◯ Very low | CRITICAL |
|  | | | | | | | | | | | | |
| **Function in people treated with needling therapies type mixed (TCM, myofascial) (follow-up: closest to 2 weeks; assessed with: RMDQ, 0-24; benefit indicated by lower values)** | | | | | | | | | | | | |
| 1^14^ | randomised trials | very serious^t^ | not serious^p^ | serious^q^ | very serious^r^ | none | Between-group MD (95% CI) of within-group MDs: −2.11 (−3.75 to −0.47) (121 participants total) | | | | ⨁◯◯◯ Very low | CRITICAL |
| **Function in people treated with needling therapies with electrical stimulation (follow-up: closest to 2 weeks; assessed with: RMDQ, 0-24; benefit indicated by lower values)** | | | | | | | | | | | | |
| 1^14^ | randomised trials | very serious^t^ | not serious^p^ | serious^q^ | very serious^r^ | none | Between-group MD (95% CI) of within-group MDs: −2.11 (−3.75 to −0.47) (121 participants total) | | | | ⨁◯◯◯ Very low | CRITICAL |
| **Function in people treated with needling therapies with manual stimulation (follow-up: closest to 2 weeks; assessed with: ODI; benefit indicated by lower values)** | | | | | | | | | | | | |
| 2^1,5,ax^ | randomised trials | very serious^t^ | serious^aj^ | not serious^e^ | very serious^r^ | none | 80 | 80 | - | SMD **0.48 lower** (0.92 lower to 0.05 lower) | ⨁◯◯◯ Very low | CRITICAL |
| **Function in people treated with needling therapies without stimulation (follow-up: closest to 2 weeks; assessed with: RMDQ, Hannover; benefit indicated by lower values)** | | | | | | | | | | | | |
| 2^4,7^ | randomised trials | not serious^x^ | serious^av^ | not serious^e^ | very serious^aw^ | none | 398 | 393 | - | SMD **0.03 lower** (0.37 lower to 0.31 higher) | ⨁◯◯◯ Very low | CRITICAL |
| **Function after removing high risk of bias studies (follow-up: closest to 2 weeks; assessed with: RMDQ, ODI, Hannover; benefit indicated by lower values)** | | | | | | | | | | | | |
| 3^1,4,7,ax^ | randomised trials | not serious^x^ | very serious^ba^ | not serious^e^ | very serious^bb^ | none | 448 | 443 | - | SMD **0.21 lower** (0.64 lower to 0.23 higher) | ⨁◯◯◯ Very low | CRITICAL |
|  | | | | | | | | | | | | |
|  | | | | | | | | | | | | |
|  | | | | | | | | | | | | |
| **Function (follow-up: closest to 3 months; assessed with: RMDQ, ODI, BPI, Hannover; benefit indicated by lower values)** | | | | | | | | | | | | |
| 7^1,4,7,9,10,11,12,aa,ax^ | randomised trials | very serious^c^ | not serious^bc^ | not serious^e^ | not serious^bd^ | none | 911 | 841 | - | SMD **0.03 lower** (0.17 lower to 0.11 higher) | ⨁⨁◯◯ Low | CRITICAL |
| **Function in people with radicular leg pain (follow-up: closest to 3 months; assessed with: ODI, 0-100; benefit indicated by lower values)** | | | | | | | | | | | | |
| 1^8,l,m,n^ | randomised trials | not serious^o^ | not serious^p^ | serious^q^ | very serious^r^ | none | Between-group MD (95% CI) of within-group MDs: -3.04 (-12.34 to 6.25) (46 participants total) | | | | ⨁◯◯◯ Very low | CRITICAL |
| **Function in people without leg pain (follow-up: closest to 3 months; assessed with: ODI, Hannover; benefit indicated by lower values)** | | | | | | | | | | | | |
| 2^1,9^ | randomised trials | very serious^t^ | not serious^k^ | not serious^e^ | serious^i^ | none | 120 | 190 | - | SMD **0.19 lower** (0.42 lower to 0.04 higher) | ⨁◯◯◯ Very low | CRITICAL |
| **Function in people either with or without leg pain (follow-up: closest to 3 months; assessed with: RMDQ; benefit indicated by lower values)** | | | | | | | | | | | | |
| 1^10,aa^ | randomised trials | very serious^t^ | not serious^p^ | serious^q^ | serious^be^ | none | 299 | 159 | - | SMD **0.18 higher** (0.01 lower to 0.37 higher) | ⨁◯◯◯ Very low | CRITICAL |
| **Function in people with unclassifed presence of leg pain (follow-up: closest to 3 months; assessed with: RMDQ, ODI, BPI, Hannover; benefit indicated by lower values)** | | | | | | | | | | | | |
| 4^4,7,11,12^ | randomised trials | serious^j^ | not serious^k^ | not serious^e^ | serious^bf^ | none | 492 | 492 | - | SMD **0.13 lower** (0.26 lower to 0.01 lower) | ⨁⨁◯◯ Low | CRITICAL |
|  | | | | | | | | | | | | |
| **Function stratified by gender (follow-up: closest to 3 months)** | | | | | | | | | | | | |
| 0 |  |  |  |  |  |  |  |  |  |  |  | CRITICAL |
| **Function stratified by race/ethnicity (follow-up: closest to 3 months)** | | | | | | | | | | | | |
| 0 |  |  |  |  |  |  |  |  |  |  |  | CRITICAL |
| **Function in people in low to lower-middle income countries (follow-up: closest to 3 months)** | | | | | | | | | | | | |
| 0 |  |  |  |  |  |  |  |  |  |  |  | CRITICAL |
| **Function in people treated with needling therapies type TCM (follow-up: closest to 3 months; assessed with: RMDQ, ODI, BPI, Hannover; benefit indicated by lower values)** | | | | | | | | | | | | |
| 5^1,7,10,11,12,aa,ax^ | randomised trials | very serious^c^ | serious^bg^ | not serious^e^ | not serious^bh^ | none | 818 | 678 | - | SMD **0**  (0.17 lower to 0.17 higher) | ⨁◯◯◯ Very low | CRITICAL |
| **Function in people treated with needling therapies type myofascial (follow-up: closest to 3 months; assessed with: RMDQ; benefit indicated by lower values)** | | | | | | | | | | | | |
| 1^4^ | randomised trials | very serious^t^ | not serious^p^ | serious^q^ | very serious^r^ | none | 23 | 23 | - | SMD **0.09 higher** (0.49 lower to 0.66 higher) | ⨁◯◯◯ Very low | CRITICAL |
| **Function in people treated with needling therapies type mixed (TCM, myofascial) (follow-up: closest to 3 months; assessed with: Hannover; benefit indicated by lower values)** | | | | | | | | | | | | |
| 1^9^ | randomised trials | very serious^t^ | not serious^p^ | serious^q^ | serious^i^ | none | 70 | 140 | - | SMD **0.2 lower** (0.49 lower to 0.08 higher) | ⨁◯◯◯ Very low | CRITICAL |
|  | | | | | | | | | | | | |
|  | | | | | | | | | | | | |
|  | | | | | | | | | | | | |
| **Function in people treated with needling therapies with manual stimulation (follow-up: closest to 3 months; assessed with: ODI, Hannover; benefit indicated by lower values)** | | | | | | | | | | | | |
| 3^1,9,11,ax^ | randomised trials | very serious^t^ | not serious^k^ | not serious^e^ | serious^bf^ | none | 177 | 249 | - | SMD **0.17 lower** (0.37 lower to 0.02 higher) | ⨁◯◯◯ Very low | CRITICAL |
| **Function in people treated with needling therapies without stimulation (follow-up: closest to 3 months; assessed with: RMDQ, BPI, Hannover; benefit indicated by lower values)** | | | | | | | | | | | | |
| 3^4,7,12^ | randomised trials | not serious^x^ | not serious^bi^ | not serious^e^ | serious^bf^ | none | 435 | 433 | - | SMD **0.07 lower** (0.3 lower to 0.17 higher) | ⨁⨁⨁◯ Moderate | CRITICAL |
| **Function in people treated with needling therapies (stimulation not reported) (follow-up: closest to 3 months; assessed with: RMDQ; benefit indicated by lower values)** | | | | | | | | | | | | |
| 1^10,aa^ | randomised trials | very serious^t^ | not serious^p^ | serious^q^ | serious^be^ | none | 299 | 159 | - | SMD **0.18 higher** (0.01 lower to 0.37 higher) | ⨁◯◯◯ Very low | CRITICAL |
| **Function after removing high risk of bias studies (follow-up: closest to 3 months; assessed with: RMDQ, ODI, Hannover; benefit indicated by lower values)** | | | | | | | | | | | | |
| 5^1,4,7,10,11,aa,ax^ | randomised trials | very serious^am^ | serious^bj^ | not serious^e^ | not serious^bd^ | none | 805 | 664 | - | SMD **0.02 lower** (0.18 lower to 0.15 higher) | ⨁◯◯◯ Very low | CRITICAL |
|  | | | | | | | | | | | | |
|  | | | | | | | | | | | | |
|  | | | | | | | | | | | | |
|  | | | | | | | | | | | | |
| **Function (follow-up: closest to 6 months; assessed with: RMDQ, ODI, Hannover; benefit indicated by lower values)** | | | | | | | | | | | | |
| 4^7,9,10,11,aa,ax^ | randomised trials | very serious^c^ | not serious^s^ | not serious^e^ | serious^bf^ | none | 788 | 729 | - | SMD **0.1 lower** (0.22 lower to 0.02 higher) | ⨁◯◯◯ Very low | CRITICAL |
| **Function in people with radicular leg pain (follow-up: closest to 6 months; assessed with: ODI, 0-100; benefit indicated by lower values)** | | | | | | | | | | | | |
| 1^8,l,m,n^ | randomised trials | not serious^o^ | not serious^p^ | serious^q^ | very serious^r^ | none | Between-group MD (95% CI) of within-group MDs: 0.09 (-10.80 to 10.98) (46 participants total) | | | | ⨁◯◯◯ Very low | CRITICAL |
| **Function in people without leg pain (follow-up: closest to 6 months; assessed with: Hannover; benefit indicated by lower values)** | | | | | | | | | | | | |
| 1^9^ | randomised trials | very serious^t^ | not serious^p^ | serious^q^ | very serious^bk^ | none | 70 | 140 | - | SMD **0.09 lower** (0.38 lower to 0.2 higher) | ⨁◯◯◯ Very low | CRITICAL |
| **Function in people with and without leg pain (follow-up: closest to 6 months; assessed with: RMDQ; benefit indicated by lower values)** | | | | | | | | | | | | |
| 1^10,aa^ | randomised trials | very serious^t^ | not serious^p^ | serious^q^ | serious^be^ | none | 285 | 153 | - | SMD **0.06 higher** (0.14 lower to 0.26 higher) | ⨁◯◯◯ Very low | CRITICAL |
| **Function in people with unclassified presence of leg pain (follow-up: closest to 6 months; assessed with: ODI, Hannover; benefit indicated by lower values)** | | | | | | | | | | | | |
| 2^7,11^ | randomised trials | not serious^x^ | not serious^k^ | not serious^e^ | not serious^bl^ | none | 433 | 436 | - | SMD **0.21 lower** (0.34 lower to 0.07 lower) | ⨁⨁⨁⨁ High | CRITICAL |
|  | | | | | | | | | | | | |
| **Function stratified by gender (follow-up: closest to 6 months)** | | | | | | | | | | | | |
| 0 |  |  |  |  |  |  |  |  |  |  |  | CRITICAL |
| **Function stratified by race/ethnicity (follow-up: closest to 6 months)** | | | | | | | | | | | | |
| 0 |  |  |  |  |  |  |  |  |  |  |  | CRITICAL |
| **Function in people in low to lower-middle income countries (follow-up: closest to 6 months)** | | | | | | | | | | | | |
| 0 |  |  |  |  |  |  |  |  |  |  |  | CRITICAL |
| **Function in people treated with needling therapies type TCM (follow-up: closest to 6 months; assessed with: RMDQ, ODI, Hannover; benefit indicated by lower values)** | | | | | | | | | | | | |
| 3^7,10,11,aa,ax^ | randomised trials | serious^j^ | not serious^bc^ | not serious^e^ | serious^bf^ | none | 718 | 589 | - | SMD **0.09 lower** (0.25 lower to 0.06 higher) | ⨁⨁◯◯ Low | CRITICAL |
| **Function in people treated with needling therapies type mixed (TCM, myofascial) (follow-up: closest to 6 months; assessed with: Hannover; benefit indicated by lower values)** | | | | | | | | | | | | |
| 1^9^ | randomised trials | very serious^t^ | not serious^p^ | serious^q^ | very serious^bk^ | none | 70 | 140 | - | SMD **0.09 lower** (0.38 lower to 0.2 higher) | ⨁◯◯◯ Very low | CRITICAL |
| **Function in people treated with needling therapies with manual stimulation (follow-up: closest to 6 months; assessed with: ODI, Hannover; benefit indicated by lower values)** | | | | | | | | | | | | |
| 2^9,11,ax^ | randomised trials | very serious^t^ | not serious^k^ | not serious^e^ | serious^i^ | none | 127 | 199 | - | SMD **0.15 lower** (0.37 lower to 0.08 higher) | ⨁◯◯◯ Very low | CRITICAL |
|  | | | | | | | | | | | | |
|  | | | | | | | | | | | | |
| **Function in people treated with needling therapies without stimulation (follow-up: closest to 6 months; assessed with: Hannover; benefit indicated by lower values)** | | | | | | | | | | | | |
| 1^7^ | randomised trials | not serious^o^ | not serious^p^ | serious^q^ | not serious^bl^ | none | 376 | 377 | - | SMD **0.2 lower** (0.34 lower to 0.06 lower) | ⨁⨁⨁◯ Moderate | CRITICAL |
| **Function in people treated with needling therapies (stimulation not reported) (follow-up: closest to 6 months; assessed with: RMDQ; benefit indicated by lower values)** | | | | | | | | | | | | |
| 1^10,aa^ | randomised trials | very serious^t^ | not serious^p^ | serious^q^ | serious^be^ | none | 285 | 153 | - | SMD **0.06 higher** (0.14 lower to 0.26 higher) | ⨁◯◯◯ Very low | CRITICAL |
| **Function after removing high risk of bias studies (follow-up: closest to 6 months; assessed with: RMDQ, ODI, Hannover; benefit indicated by lower values)** | | | | | | | | | | | | |
| 3^7,10,11,aa,ax^ | randomised trials | serious^bm^ | not serious^bc^ | not serious^e^ | serious^bf^ | none | 718 | 589 | - | SMD **0.09 lower** (0.25 lower to 0.06 higher) | ⨁⨁◯◯ Low | CRITICAL |
| **Health-related quality of life (follow-up: closest to 2 weeks; assessed with: SF-36; benefit indicated by higher values; Scale from: 0 to 100)** | | | | | | | | | | | | |
| 1^6,ax^ | randomised trials | very serious^t^ | not serious^p^ | serious^q^ | very serious^r^ | none | 26 | 20 | - | MD **6.4 higher** (6.42 lower to 19.22 higher) | ⨁◯◯◯ Very low | CRITICAL |
| **Health-related quality of life in people with radicular leg pain (follow-up: closest to 2 weeks; assessed with: SF-36; benefit indicated by higher values)** | | | | | | | | | | | | |
| 1^8,l,m,n^ | randomised trials | not serious^o^ | not serious^p^ | serious^q^ | very serious^r^ | none | No significant difference between groups for mean change from baseline on any of the subscales (46 participants total). | | | | ⨁◯◯◯ Very low | CRITICAL |
| **Health-related quality of life in people with unclassified presence of leg pain (follow-up: closest to 2 weeks; assessed with: SF-36; benefit indicated by higher values; Scale from: 0 to 100)** | | | | | | | | | | | | |
| 1^6^ | randomised trials | very serious^t^ | not serious^p^ | serious^q^ | very serious^r^ | none | 26 | 20 | - | MD **6.4 higher** (6.42 lower to 19.22 higher) | ⨁◯◯◯ Very low | CRITICAL |
| **Health-related quality of life stratified by gender (follow-up: closest to 2 weeks)** | | | | | | | | | | | | |
| 0 |  |  |  |  |  |  |  |  |  |  |  | CRITICAL |
| **Health-related quality of life stratified by race-ethnicity (follow-up: closest to 2 weeks)** | | | | | | | | | | | | |
| 0 |  |  |  |  |  |  |  |  |  |  |  | CRITICAL |
| **Health-related quality of life in people in low to lower-middle income countries (follow-up: closest to 2 weeks)** | | | | | | | | | | | | |
| 0 |  |  |  |  |  |  |  |  |  |  |  | CRITICAL |
| **Health-related quality of life in people treated with needling therapies type TCM (follow-up: closest to 2 weeks; assessed with: SF-36; benefit indicated by higher values; Scale from: 0 to 100)** | | | | | | | | | | | | |
| 1^6,bn^ | randomised trials | very serious^t^ | not serious^p^ | serious^q^ | very serious^r^ | none | 26 | 20 | - | MD **6.4 higher** (6.42 lower to 19.22 higher) | ⨁◯◯◯ Very low | CRITICAL |
| **Health-related quality of life in people treated with needling therapies with manual stimulation (follow-up: closest to 2 weeks; assessed with: SF-36; benefit indicated by higher values; Scale from: 0 to 100)** | | | | | | | | | | | | |
| 1^6,bn^ | randomised trials | very serious^t^ | not serious^p^ | serious^q^ | very serious^r^ | none | 26 | 20 | - | MD **6.4 higher** (6.42 lower to 19.22 higher) | ⨁◯◯◯ Very low | CRITICAL |
|  | | | | | | | | | | | | |
|  | | | | | | | | | | | | |
| **Health-related quality of life after removing high risk of bias studies (follow-up: closest to 2 weeks)** | | | | | | | | | | | | |
| 1^8,l,m,n^ | randomised trials | not serious^o^ | not serious^p^ | serious^q^ | very serious^r^ | none | No improvement in needling therapies versus sham group (43 participants total) | | | | ⨁◯◯◯ Very low | CRITICAL |
| **Health-related quality of life (follow-up: closest to 3 months; assessed with: SF-36; benefit indicated by higher values; Scale from: 0 to 100)** | | | | | | | | | | | | |
| 1^11,bo^ | randomised trials | very serious^t^ | not serious^p^ | serious^q^ | very serious^r^ | none | 57 | 59 | - | MD **7.78 higher** (1.41 higher to 14.15 higher) | ⨁◯◯◯ Very low | CRITICAL |
| **Health -related quality of life (follow-up: closest to 3 months; assessed with: SF-36 (PCS); benefit indicated by higher values)** | | | | | | | | | | | | |
| 2^7,9^ | randomised trials | serious^j^ | very serious^bp^ | serious^q^ | serious^bq^ | none | 510 | 442 | - | SMD **0.25 higher** (0.07 lower to 0.56 higher) | ⨁◯◯◯ Very low | CRITICAL |
| **Health-related quality of life in people without leg pain (follow-up: closest to 3 months; assessed with: SF-36 (PCS); benefit indicated by higher values)** | | | | | | | | | | | | |
| 1^9^ | randomised trials | very serious^t^ | not serious^p^ | serious^q^ | serious^i^ | none | 140 | 70 | - | SMD **0.43 higher** (0.14 higher to 0.72 higher) | ⨁◯◯◯ Very low | CRITICAL |
| **Health-related quality of life in people with unclassified presence of leg pain (follow-up: closest to 3 months; assessed with: SF-36 (PCS); benefit indicated by higher values)** | | | | | | | | | | | | |
| 1^7^ | randomised trials | not serious^o^ | not serious^p^ | serious^q^ | serious^br^ | none | 370 | 372 | - | SMD **0.11 higher** (0.03 lower to 0.25 higher) | ⨁⨁◯◯ Low | CRITICAL |
|  | | | | | | | | | | | | |
| **Health-related quality of life in people treated with needling therapies type TCM (follow-up: closest to 3 months; assessed with: SF-36 (PCS); benefit indicated by higher values)** | | | | | | | | | | | | |
| 1^7,bs^ | randomised trials | not serious^o^ | not serious^p^ | serious^q^ | serious^br^ | none | 370 | 372 | - | SMD **0.11 higher** (0.03 lower to 0.25 higher) | ⨁⨁◯◯ Low | CRITICAL |
| **Health-related quality of life in people treated with needling therapies type mixed (TCM, myofascial) (follow-up: closest to 3 months; assessed with: SF-36 (PCS); benefit indicated by higher values)** | | | | | | | | | | | | |
| 1^9^ | randomised trials | very serious^t^ | not serious^p^ | serious^q^ | serious^i^ | none | 140 | 70 | - | SMD **0.43 higher** (0.14 higher to 0.72 higher) | ⨁◯◯◯ Very low | CRITICAL |
| **Health-related quality of life in people treated with needling therapies with manual stimulation (follow-up: closest to 3 months; assessed with: SF-36 (PCS); benefit indicated by higher values)** | | | | | | | | | | | | |
| 1^9^ | randomised trials | very serious^t^ | not serious^p^ | serious^q^ | serious^i^ | none | 140 | 70 | - | SMD **0.43 higher** (0.14 higher to 0.72 higher) | ⨁◯◯◯ Very low | CRITICAL |
| **Health-related quality of life in people treated with needling therapies without stimulation (follow-up: closest to 3 months; assessed with: SF-36 (PCS); benefit indicated by higher values)** | | | | | | | | | | | | |
| 1^7^ | randomised trials | not serious^o^ | not serious^p^ | serious^q^ | serious^br^ | none | 370 | 372 | - | SMD **0.11 higher** (0.03 lower to 0.25 higher) | ⨁⨁◯◯ Low | CRITICAL |
|  | | | | | | | | | | | | |
|  | | | | | | | | | | | | |
|  | | | | | | | | | | | | |
| **Health-related quality of life after removing high risk of bias studies (follow-up: closest to 3 months; assessed with: SF-36 (PCS); benefit indicated by higher values)** | | | | | | | | | | | | |
| 1^7^ | randomised trials | not serious^o^ | not serious^p^ | serious^q^ | serious^br^ | none | 370 | 372 | - | SMD **0.11 higher** (0.03 lower to 0.25 higher) | ⨁⨁◯◯ Low | CRITICAL |
| **Health-related quality of life (follow-up: closest to 3 months; assessed with: SF-36 (MCS); benefit indicated by higher values)** | | | | | | | | | | | | |
| 2^7,9^ | randomised trials | serious^j^ | not serious^k^ | serious^q^ | not serious^bt^ | none | 510 | 442 | - | SMD **0.01 higher** (0.12 lower to 0.14 higher) | ⨁⨁◯◯ Low | CRITICAL |
| **Health-related quality of life in people without leg pain (follow-up: closest to 3 months; assessed with: SF-36 (MCS); benefit indicated by higher values)** | | | | | | | | | | | | |
| 1^9^ | randomised trials | very serious^t^ | not serious^p^ | serious^q^ | very serious^bu^ | none | 140 | 70 | - | SMD **0.04 lower** (0.33 lower to 0.25 higher) | ⨁◯◯◯ Very low | CRITICAL |
| **Health-related quality of life in people with unclassified presence of leg pain (follow-up: closest to 3 months; assessed with: SF-36 (MCS); benefit indicated by higher values)** | | | | | | | | | | | | |
| 1^7^ | randomised trials | not serious^o^ | not serious^p^ | serious^q^ | not serious^bt^ | none | 370 | 372 | - | SMD **0.03 higher** (0.12 lower to 0.17 higher) | ⨁⨁⨁◯ Moderate | CRITICAL |
|  | | | | | | | | | | | | |
|  | | | | | | | | | | | | |
|  | | | | | | | | | | | | |
| **Health-related quality of life in people treated with needling therapies type TCM (follow-up: closest to 3 months; assessed with: SF-36 (MCS); benefit indicated by higher values)** | | | | | | | | | | | | |
| 1^7^ | randomised trials | not serious^o^ | not serious^p^ | serious^q^ | not serious^bt^ | none | 370 | 372 | - | SMD **0.03 higher** (0.12 lower to 0.17 higher) | ⨁⨁⨁◯ Moderate | CRITICAL |
| **Health-related quality of life in people treated with needling therapies type mixed (TCM, myofascial) (follow-up: closest to 3 months; assessed with: SF-36 (MCS); benefit indicated by higher values)** | | | | | | | | | | | | |
| 1^9^ | randomised trials | very serious^t^ | not serious^p^ | serious^q^ | very serious^bu^ | none | 140 | 70 | - | SMD **0.04 lower** (0.33 lower to 0.25 higher) | ⨁◯◯◯ Very low | CRITICAL |
| **Health-related quality of life in people treated with needling therapies with manual stimulation (follow-up: closest to 3 months; assessed with: SF-36 (MCS); benefit indicated by higher values)** | | | | | | | | | | | | |
| 1^9^ | randomised trials | very serious^t^ | not serious^p^ | serious^q^ | very serious^bu^ | none | 140 | 70 | - | SMD **0.04 lower** (0.33 lower to 0.25 higher) | ⨁◯◯◯ Very low | CRITICAL |
| **Health-related quality of life in people treated with needling therapies without stimulation (follow-up: closest to 3 months; assessed with: SF-36 (MCS); benefit indicated by higher values)** | | | | | | | | | | | | |
| 1^7^ | randomised trials | not serious^o^ | not serious^p^ | serious^q^ | not serious^bt^ | none | 370 | 372 | - | SMD **0.03 higher** (0.12 lower to 0.17 higher) | ⨁⨁⨁◯ Moderate | CRITICAL |
|  | | | | | | | | | | | | |
|  | | | | | | | | | | | | |
|  | | | | | | | | | | | | |
| **Health-related quality of life after removing high risk of bias studies (follow-up: closest to 3 months; assessed with: SF-36 (MCS); benefit indicated by higher values)** | | | | | | | | | | | | |
| 1^7^ | randomised trials | not serious^o^ | not serious^p^ | serious^q^ | not serious^bt^ | none | 370 | 372 | - | SMD **0.03 higher** (0.12 lower to 0.17 higher) | ⨁⨁⨁◯ Moderate | CRITICAL |
| **Health-related quality of life stratified by gender (follow-up: closest to 3 months)** | | | | | | | | | | | | |
| 0 |  |  |  |  |  |  |  |  |  |  |  | CRITICAL |
| **Health-related quality of life stratified by race/ethnicity (follow-up: closest to 3 months)** | | | | | | | | | | | | |
| 0 |  |  |  |  |  |  |  |  |  |  |  | CRITICAL |
| **Health-related quality of life in people in low to lower-middle income countries (follow-up: closest to 3 months)** | | | | | | | | | | | | |
| 0 |  |  |  |  |  |  |  |  |  |  |  | CRITICAL |
| **Health-related quality of life (follow-up: closest to 6 months; assessed with: SF-36; benefit indicated by higher values; Scale from: 0 to 100)** | | | | | | | | | | | | |
| 1^11,bo^ | randomised trials | very serious^t^ | not serious^p^ | serious^q^ | very serious^r^ | none | 57 | 59 | - | MD **3.39 higher** (2.98 lower to 9.76 higher) | ⨁◯◯◯ Very low | CRITICAL |
| **Health-related quality of life (follow-up: closest to 6 months; assessed with: SF-36 (PCS); benefit indicated by higher values)** | | | | | | | | | | | | |
| 2^7,9^ | randomised trials | serious^j^ | not serious^k^ | serious^q^ | not serious^bl^ | none | 513 | 442 | - | SMD **0.2 higher** (0.07 higher to 0.32 higher) | ⨁⨁◯◯ Low | CRITICAL |
|  | | | | | | | | | | | | |
|  | | | | | | | | | | | | |
| **Health-related quality of life in people without leg pain (follow-up: closest to 6 months; assessed with: SF-36 (PCS); benefit indicated by higher values)** | | | | | | | | | | | | |
| 1^9^ | randomised trials | very serious^t^ | not serious^p^ | serious^q^ | serious^i^ | none | 140 | 70 | - | SMD **0.16 higher** (0.12 lower to 0.45 higher) | ⨁◯◯◯ Very low | CRITICAL |
| **Health-related quality of life in people with unclassified presence of leg pain (follow-up: closest to 6 months; assessed with: SF-36 (PCS); benefit indicated by higher values)** | | | | | | | | | | | | |
| 1^7^ | randomised trials | not serious^o^ | not serious^p^ | serious^q^ | not serious^bl^ | none | 373 | 372 | - | SMD **0.2 higher** (0.06 higher to 0.35 higher) | ⨁⨁⨁◯ Moderate | CRITICAL |
| **Health-related quality of life in people treated with needling therapies type TCM (follow-up: closest to 6 months; assessed with: SF-36 (PCS); benefit indicated by higher values)** | | | | | | | | | | | | |
| 1^7^ | randomised trials | not serious^o^ | not serious^p^ | serious^q^ | not serious^bl^ | none | 373 | 372 | - | SMD **0.2 higher** (0.06 higher to 0.35 higher) | ⨁⨁⨁◯ Moderate | CRITICAL |
| **Health-related quality of life in people treated with needling therapies type mixed (TCM, myofascial) (follow-up: closest to 6 months; assessed with: SF-36 (PCS); benefit indicated by higher values)** | | | | | | | | | | | | |
| 1^9^ | randomised trials | very serious^t^ | not serious^p^ | serious^q^ | serious^i^ | none | 140 | 70 | - | SMD **0.16 higher** (0.12 lower to 0.45 higher) | ⨁◯◯◯ Very low | CRITICAL |
|  | | | | | | | | | | | | |
|  | | | | | | | | | | | | |
|  | | | | | | | | | | | | |
| **Health-related quality of life in people treated with needling therapies with manual stimulation (follow-up: closest to 6 months; assessed with: SF-36 (PCS); benefit indicated by higher values)** | | | | | | | | | | | | |
| 1^9^ | randomised trials | very serious^t^ | not serious^p^ | serious^q^ | serious^i^ | none | 140 | 70 | - | SMD **0.16 higher** (0.12 lower to 0.45 higher) | ⨁◯◯◯ Very low | CRITICAL |
| **Health-related quality of life in people treated with needling therapies (without stimulation) (follow-up: closest to 6 months; assessed with: SF-36 (PCS); benefit indicated by higher values)** | | | | | | | | | | | | |
| 1^7^ | randomised trials | not serious^o^ | not serious^p^ | serious^q^ | not serious^bl^ | none | 373 | 372 | - | SMD **0.2 higher** (0.06 higher to 0.35 higher) | ⨁⨁⨁◯ Moderate | CRITICAL |
| **Health-related quality of life after removing high risk of bias studies (follow-up: closest to 6 months; assessed with: SF-36 (PCS); benefit indicated by higher values)** | | | | | | | | | | | | |
| 1^7^ | randomised trials | not serious^o^ | not serious^p^ | serious^q^ | not serious^bl^ | none | 373 | 372 | - | SMD **0.2 higher** (0.06 higher to 0.35 higher) | ⨁⨁⨁◯ Moderate | CRITICAL |
| **Health-related quality of life (follow-up: closest to 6 months; assessed with: SF-36 (MCS); benefit indicated by higher values)** | | | | | | | | | | | | |
| 2^7,9^ | randomised trials | serious^j^ | very serious^bv^ | serious^q^ | serious^br^ | none | 513 | 442 | - | SMD **0.1 higher** (0.18 lower to 0.39 higher) | ⨁◯◯◯ Very low | CRITICAL |
|  | | | | | | | | | | | | |
|  | | | | | | | | | | | | |
|  | | | | | | | | | | | | |
| **Health-related quality of life in people without leg pain (follow-up: closest to 6 months; assessed with: SF-36 (MCS); benefit indicated by higher values)** | | | | | | | | | | | | |
| 1^9^ | randomised trials | very serious^t^ | not serious^p^ | serious^q^ | serious^i^ | none | 140 | 70 | - | SMD **0.28 higher** (0.01 lower to 0.57 higher) | ⨁◯◯◯ Very low | CRITICAL |
| **Health-related quality of life in people with unclassified presence of leg pain (follow-up: closest to 6 months; assessed with: SF-36 (MCS); benefit indicated by higher values)** | | | | | | | | | | | | |
| 1^7^ | randomised trials | not serious^o^ | not serious^p^ | serious^q^ | not serious^bt^ | none | 373 | 372 | - | SMD **0.02 lower** (0.16 lower to 0.13 higher) | ⨁⨁⨁◯ Moderate | CRITICAL |
| **Health-related quality of life in people treated with needling therapies type TCM (follow-up: closest to 6 weeks; assessed with: SF-36 (MCS); benefit indicated by higher values)** | | | | | | | | | | | | |
| 1^7^ | randomised trials | not serious^o^ | not serious^p^ | serious^q^ | not serious^bt^ | none | 373 | 372 | - | SMD **0.02 lower** (0.16 lower to 0.13 higher) | ⨁⨁⨁◯ Moderate | CRITICAL |
| **Health-related quality of life in people treated with needling therapies type mixed (TCM, myofascial) (follow-up: closest to 6 months; assessed with: SF-36 (MCS); benefit indicated by higher values)** | | | | | | | | | | | | |
| 1^9^ | randomised trials | very serious^t^ | not serious^p^ | serious^q^ | serious^i^ | none | 140 | 70 | - | SMD **0.28 higher** (0.01 lower to 0.57 higher) | ⨁◯◯◯ Very low | CRITICAL |
|  | | | | | | | | | | | | |
|  | | | | | | | | | | | | |
|  | | | | | | | | | | | | |
| **Health-related quality of life in people treated with needling therapies with manual stimulation (follow-up: closest to 6 months; assessed with: SF-36 (MCS); benefit indicated by higher values)** | | | | | | | | | | | | |
| 1^9^ | randomised trials | very serious^t^ | not serious^p^ | serious^q^ | serious^i^ | none | 140 | 70 | - | SMD **0.28 higher** (0.01 lower to 0.57 higher) | ⨁◯◯◯ Very low | CRITICAL |
| **Health-related quality of life in people treated with needling therapies without stimulation (follow-up: closest to 6 months; assessed with: SF-36 (MCS); benefit indicated by higher values)** | | | | | | | | | | | | |
| 1^7^ | randomised trials | not serious^o^ | not serious^p^ | serious^q^ | not serious^bt^ | none | 373 | 372 | - | SMD **0.02 lower** (0.16 lower to 0.13 higher) | ⨁⨁⨁◯ Moderate | CRITICAL |
| **Health-related quality of life after removing high risk of bias studies (follow-up: closest to 6 months; assessed with: SF-36 (MCS); benefit indicated by higher values)** | | | | | | | | | | | | |
| 1^7^ | randomised trials | not serious^o^ | not serious^p^ | serious^q^ | not serious^bt^ | none | 373 | 372 | - | SMD **0.02 lower** (0.16 lower to 0.13 higher) | ⨁⨁⨁◯ Moderate | CRITICAL |
| **Health-related quality of life stratified by gender** | | | | | | | | | | | | |
| 0 |  |  |  |  |  |  |  |  |  |  |  | CRITICAL |
| **Health-related quality of life stratified by race/ethnicity** | | | | | | | | | | | | |
| 0 |  |  |  |  |  |  |  |  |  |  |  | CRITICAL |
| **Health-related quality of life in people in low to lower-middle income countries** | | | | | | | | | | | | |
| 0 |  |  |  |  |  |  |  |  |  |  |  | CRITICAL |
|  | | | | | | | | | | | | |
|  | | | | | | | | | | | | |
| **Depression (follow-up: closest to 2 weeks; assessed with: General Depression Scale; benefit indicated by lower values; Scale from: 0 to 60)** | | | | | | | | | | | | |
| 1^9^ | randomised trials | very serious^t^ | not serious^p^ | serious^q^ | serious^i^ | none | 140 | 70 | - | MD **2.5 lower** (5.23 lower to 0.23 higher) | ⨁◯◯◯ Very low | CRITICAL |
| **Depression in people without leg pain (follow-up: closest to 2 weeks; assessed with: General Depression Scale; benefit indicated by lower values; Scale from: 0 to 60)** | | | | | | | | | | | | |
| 1^9^ | randomised trials | very serious^t^ | not serious^p^ | serious^q^ | serious^i^ | none | 140 | 70 | - | MD **2.5 lower** (5.23 lower to 0.23 higher) | ⨁◯◯◯ Very low | CRITICAL |
| **Depression stratified by gender (follow-up: closest to 2 weeks)** | | | | | | | | | | | | |
| 0 |  |  |  |  |  |  |  |  |  |  |  | CRITICAL |
| **Depression stratified by race/ethnicity (follow-up: closest to 2 weeks)** | | | | | | | | | | | | |
| 0 |  |  |  |  |  |  |  |  |  |  |  | CRITICAL |
| **Depression in people in low to lower-middle income countries (follow-up: closest to 2 weeks)** | | | | | | | | | | | | |
| 0 |  |  |  |  |  |  |  |  |  |  |  | CRITICAL |
| **Depression in people treated with needling therapies type mixed (TCM, myofascial) (follow-up: closest to 2 weeks; assessed with: General Depression Scale; benefit indicated by lower values; Scale from: 0 to 60)** | | | | | | | | | | | | |
| 1^9^ | randomised trials | very serious^t^ | not serious^p^ | serious^q^ | serious^i^ | none | 140 | 70 | - | MD **2.5 lower** (5.23 lower to 0.23 higher) | ⨁◯◯◯ Very low | CRITICAL |
|  | | | | | | | | | | | | |
|  | | | | | | | | | | | | |
| **Depression in people treated with needling therapies with manual stimulation (follow-up: closest to 2 weeks; assessed with: General Depression Scale; benefit indicated by lower values; Scale from: 0 to 60)** | | | | | | | | | | | | |
| 1^9^ | randomised trials | very serious^t^ | not serious^p^ | serious^q^ | serious^i^ | none | 140 | 70 | - | MD **2.5 lower** (5.23 lower to 0.23 higher) | ⨁◯◯◯ Very low | CRITICAL |
| **Depression (follow-up: closest to 3 months; assessed with: BDI, General Depression Scale; benefit indicated by lower values)** | | | | | | | | | | | | |
| 2^9,11^ | randomised trials | very serious^t^ | not serious^ak^ | not serious^e^ | serious^i^ | none | 197 | 129 | - | SMD **0.17 lower** (0.44 lower to 0.1 higher) | ⨁◯◯◯ Very low | CRITICAL |
| **Depression in people without leg pain (follow-up: closest to 3 months; assessed with: General Depression Scale; benefit indicated by lower values)** | | | | | | | | | | | | |
| 1^9^ | randomised trials | very serious^t^ | not serious^p^ | serious^q^ | very serious^aw^ | none | 140 | 70 | - | SMD **0.05 lower** (0.34 lower to 0.23 higher) | ⨁◯◯◯ Very low | CRITICAL |
| **Depression in people with unclassified presence of leg pain (follow-up: closest to 3 months; assessed with: BDI; benefit indicated by lower values)** | | | | | | | | | | | | |
| 1^11^ | randomised trials | very serious^t^ | not serious^p^ | serious^q^ | very serious^r^ | none | 57 | 59 | - | SMD **0.33 lower** (0.7 lower to 0.03 higher) | ⨁◯◯◯ Very low | CRITICAL |
|  | | | | | | | | | | | | |
|  | | | | | | | | | | | | |
|  | | | | | | | | | | | | |
| **Depression in people treated with needling therapies type TCM (follow-up: closest to 3 months; assessed with: BDI; benefit indicated by lower values)** | | | | | | | | | | | | |
| 1^11^ | randomised trials | very serious^t^ | not serious^p^ | serious^q^ | very serious^r^ | none | 57 | 59 | - | SMD **0.33 lower** (0.7 lower to 0.03 higher) | ⨁◯◯◯ Very low | CRITICAL |
| **Depression in people treated with needling therapies type mixed (TCM, myofascial) (follow-up: closest to 3 months; assessed with: General Depression Scale; benefit indicated by lower values)** | | | | | | | | | | | | |
| 1^9^ | randomised trials | very serious^t^ | not serious^p^ | serious^q^ | very serious^aw^ | none | 140 | 70 | - | SMD **0.05 lower** (0.34 lower to 0.23 higher) | ⨁◯◯◯ Very low | CRITICAL |
| **Depression in people treated with needling therapies with manual stimulation (follow-up: closest to 3 months; assessed with: BDI, General Depression Scale; benefit indicated by lower values)** | | | | | | | | | | | | |
| 2^9,11^ | randomised trials | very serious^t^ | not serious^ak^ | not serious^e^ | serious^i^ | none | 197 | 129 | - | SMD **0.17 lower** (0.44 lower to 0.1 higher) | ⨁◯◯◯ Very low | CRITICAL |
| **Depression stratified by gender (follow-up: closest to 3 months)** | | | | | | | | | | | | |
| 0 |  |  |  |  |  |  |  |  |  |  |  | CRITICAL |
| **Depression stratified by race/ethnicity** | | | | | | | | | | | | |
| 0 |  |  |  |  |  |  |  |  |  |  |  | CRITICAL |
| **Depression in people in low to lower-middle income countries (follow-up: closest to 3 months)** | | | | | | | | | | | | |
| 0 |  |  |  |  |  |  |  |  |  |  |  | CRITICAL |
|  | | | | | | | | | | | | |
|  | | | | | | | | | | | | |
|  | | | | | | | | | | | | |
| **Depression after removing high risk of bias studies (follow-up: closest to 3 months; assessed with: BDI; benefit indicated by lower values)** | | | | | | | | | | | | |
| 1^11^ | randomised trials | very serious^t^ | not serious^p^ | serious^q^ | very serious^r^ | none | 57 | 59 | - | SMD **0.33 lower** (0.7 lower to 0.03 higher) | ⨁◯◯◯ Very low | CRITICAL |
| **Depression (follow-up: closest to 6 months; assessed with: BDI, General Depression Scale; benefit indicated by lower values)** | | | | | | | | | | | | |
| 2^9,11^ | randomised trials | very serious^t^ | not serious^k^ | not serious^e^ | serious^i^ | none | 197 | 129 | - | SMD **0.1 lower** (0.33 lower to 0.12 higher) | ⨁◯◯◯ Very low | CRITICAL |
| **Depression in people without leg pain (follow-up: closest to 6 months; assessed with: General Depression Scale; benefit indicated by lower values)** | | | | | | | | | | | | |
| 1^9^ | randomised trials | very serious^t^ | not serious^p^ | serious^q^ | very serious^aw^ | none | 140 | 70 | - | SMD **0.06 lower** (0.35 lower to 0.22 higher) | ⨁◯◯◯ Very low | CRITICAL |
| **Depression in people with unclassified presence of leg pain (follow-up: closest to 6 months; assessed with: BDI; benefit indicated by lower values)** | | | | | | | | | | | | |
| 1^11^ | randomised trials | very serious^t^ | not serious^p^ | serious^q^ | very serious^r^ | none | 57 | 59 | - | SMD **0.17 lower** (0.53 lower to 0.2 higher) | ⨁◯◯◯ Very low | CRITICAL |
| **Depression stratified by gender (follow-up: closest to 6 months)** | | | | | | | | | | | | |
| 0 |  |  |  |  |  |  |  |  |  |  |  | CRITICAL |
| **Depression stratified by race/ethnicity (follow-up: closest to 6 months)** | | | | | | | | | | | | |
| 0 |  |  |  |  |  |  |  |  |  |  |  | CRITICAL |
| **Depression in low to lower-middle income countries (follow-up: closest to 6 months)** | | | | | | | | | | | | |
| 0 |  |  |  |  |  |  |  |  |  |  |  | CRITICAL |
| **Depression in people treated with needling therapies type TCM (follow-up: closest to 6 months; assessed with: BDI; benefit indicated by lower values)** | | | | | | | | | | | | |
| 1^11^ | randomised trials | very serious^t^ | not serious^p^ | serious^q^ | very serious^r^ | none | 57 | 59 | - | SMD **0.17 lower** (0.53 lower to 0.2 higher) | ⨁◯◯◯ Very low | CRITICAL |
| **Depression in people treated with needling therapies type mixed (TCM, myofascial) (follow-up: closest to 6 months; assessed with: General Depression Scale; benefit indicated by lower values)** | | | | | | | | | | | | |
| 1^9^ | randomised trials | very serious^t^ | not serious^p^ | serious^q^ | very serious^aw^ | none | 140 | 70 | - | SMD **0.06 lower** (0.35 lower to 0.22 higher) | ⨁◯◯◯ Very low | CRITICAL |
| **Depression in people treated with needling therapies with manual stimulation (follow-up: closest to 6 months; assessed with: BDI, General Depression Scale; benefit indicated by lower values)** | | | | | | | | | | | | |
| 2^9,11^ | randomised trials | very serious^t^ | not serious^k^ | not serious^e^ | serious^i^ | none | 197 | 129 | - | SMD **0.1 lower** (0.33 lower to 0.12 higher) | ⨁◯◯◯ Very low | CRITICAL |
| **Depression after removing high risk of bias studies (follow-up: closest to 6 months; assessed with: BDI; benefit indicated by lower values)** | | | | | | | | | | | | |
| 1^11^ | randomised trials | very serious^t^ | not serious^p^ | serious^q^ | very serious^r^ | none | 57 | 59 | - | SMD **0.17 lower** (0.53 lower to 0.2 higher) | ⨁◯◯◯ Very low | CRITICAL |
|  | | | | | | | | | | | | |
| **Other psychological functioning (fear avoidance, catastrophizing, anxiety, self-efficacy)** | | | | | | | | | | | | |
| 0 |  |  |  |  |  |  |  |  |  |  |  | CRITICAL |
| **Social participation** | | | | | | | | | | | | |
| 0 |  |  |  |  |  |  |  |  |  |  |  | CRITICAL |
| **Adverse events/harms during intervention period** | | | | | | | | | | | | |
| 6^1,5,8,9,10,14,bw,bx^ | randomised trials | very serious^c^ | very serious^by^ | not serious^e^ | serious^bz^ | none | 66/617 (10.7%) | 35/397 (8.8%) | **OR 1.62** (0.67 to 3.90) | **47 more per 1,000** (from 27 fewer to 186 more) | ⨁◯◯◯ Very low | CRITICAL |
| **Adverse events/harms in people with radicular leg pain during intervention period** | | | | | | | | | | | | |
| 1^8,ca^ | randomised trials | not serious^cb^ | not serious^p^ | serious^q^ | very serious^r^ | none | 2/23 (8.7%) | 0/23 (0.0%) | **OR 5.47** (0.25 to 120.37) | **0 fewer per 1,000** (from 0 fewer to 0 fewer) | ⨁◯◯◯ Very low | CRITICAL |
| **Adverse events/harms in people with and without leg pain during intervention period** | | | | | | | | | | | | |
| 1^10,cc^ | randomised trials | very serious^t^ | not serious^p^ | serious^q^ | serious^bz^ | none | 12/315 (3.8%) | 0/162 (0.0%) | **OR 13.39** (0.79 to 227.53) | **0 fewer per 1,000** (from 0 fewer to 0 fewer) | ⨁◯◯◯ Very low | CRITICAL |
| **Adverse events/harms in people without leg pain during intervention period** | | | | | | | | | | | | |
| 4^1,5,9,14,cd,ce^ | randomised trials | very serious^t^ | very serious^h^ | not serious^e^ | serious^bz^ | none | 52/279 (18.6%) | 35/212 (16.5%) | **OR 1.24** (0.50 to 3.04) | **32 more per 1,000** (from 75 fewer to 210 more) | ⨁◯◯◯ Very low | CRITICAL |
| **Adverse events/harms stratified by gender** | | | | | | | | | | | | |
| 0 |  |  |  |  |  |  |  |  |  |  |  | CRITICAL |
| **Adverse events/harms stratified by race/ethnicity** | | | | | | | | | | | | |
| 0 |  |  |  |  |  |  |  |  |  |  |  | CRITICAL |
| **Adverse events/harms in people in low to lower-middle income countries** | | | | | | | | | | | | |
| 0 |  |  |  |  |  |  |  |  |  |  |  | CRITICAL |
| **Adverse events/harms in people treated with needling therapies type TCM during intervention period** | | | | | | | | | | | | |
| 3^1,8,10,bw,cf^ | randomised trials | very serious^c^ | serious^cg^ | not serious^e^ | serious^bz^ | none | 22/388 (5.7%) | 9/235 (3.8%) | **OR 2.77** (0.39 to 19.97) | **61 more per 1,000** (from 23 fewer to 405 more) | ⨁◯◯◯ Very low | CRITICAL |
| **Adverse events/harms in people treated with needling therapies type myofascial during intervention period** | | | | | | | | | | | | |
| 1^5,ch^ | randomised trials | very serious^t^ | not serious^p^ | serious^q^ | very serious^r^ | none | 5/30 (16.7%) | 4/30 (13.3%) | **OR 1.30** (0.31 to 5.40) | **33 more per 1,000** (from 88 fewer to 320 more) | ⨁◯◯◯ Very low | CRITICAL |
| **Adverse events/harms in people treated with needling therapies type mixed (TCM, myofascial) during intervention period** | | | | | | | | | | | | |
| 2^9,14,ci^ | randomised trials | very serious^t^ | very serious^cj^ | not serious^e^ | serious^bz^ | none | 39/199 (19.6%) | 22/132 (16.7%) | **OR 1.43** (0.24 to 8.50) | **56 more per 1,000** (from 121 fewer to 463 more) | ⨁◯◯◯ Very low | CRITICAL |
| **Adverse events/harms in people treated with needling therapies with manual stimulation during intervention period** | | | | | | | | | | | | |
| 3^1,5,9,ck,cl^ | randomised trials | very serious^t^ | not serious^k^ | not serious^e^ | very serious^cm^ | none | 28/220 (12.7%) | 25/150 (16.7%) | **OR 0.76** (0.42 to 1.36) | **35 fewer per 1,000** (from 89 fewer to 47 more) | ⨁◯◯◯ Very low | CRITICAL |
|  | | | | | | | | | | | | |
|  | | | | | | | | | | | | |
| **Adverse events/harms in people treated with needling therapies with electrical stimulation during intervention period** | | | | | | | | | | | | |
| 1^14,cn^ | randomised trials | very serious^t^ | not serious^p^ | serious^q^ | very serious^r^ | none | 24/59 (40.7%) | 10/62 (16.1%) | **OR 3.57** (1.52 to 8.37) | **246 more per 1,000** (from 65 more to 456 more) | ⨁◯◯◯ Very low | CRITICAL |
| **Adverse events/harms in people treated with needling therapies without stimulation during intervention period** | | | | | | | | | | | | |
| 1^8,ca,co^ | randomised trials | not serious^cb^ | not serious^p^ | serious^q^ | very serious^r^ | none | 2/23 (8.7%) | 0/23 (0.0%) | **OR 5.47** (0.25 to 120.37) | **0 fewer per 1,000** (from 0 fewer to 0 fewer) | ⨁◯◯◯ Very low | CRITICAL |
| **Adverse events/harms in people treated with needling therapies (stimulation not reported) during intervention period** | | | | | | | | | | | | |
| 1^10,cc^ | randomised trials | very serious^t^ | serious^p^ | serious^q^ | serious^bz^ | none | 12/315 (3.8%) | 0/162 (0.0%) | **OR 13.39** (0.79 to 227.53) | **0 fewer per 1,000** (from 0 fewer to 0 fewer) | ⨁◯◯◯ Very low | CRITICAL |
| **Adverse events/harms after removing high risk of bias studies during intervention period** | | | | | | | | | | | | |
| 3^1,8,10,cf,co^ | randomised trials | very serious^t^ | serious^cg^ | not serious^e^ | serious^bz^ | none | 22/388 (5.7%) | 9/235 (3.8%) | **OR 2.77** (0.39 to 19.97) | **61 more per 1,000** (from 23 fewer to 405 more) | ⨁◯◯◯ Very low | CRITICAL |
| **OLDER ADULTS (aged 60 years or more)** | | | | | | | | | | | | |
| **Pain (people with radicular leg pain, high-income country) (follow-up: closest to 2 weeks; assessed with: VAS, 0-100; benefit indicated by lower values)** | | | | | | | | | | | | |
| 1^8,l,m,n^ | randomised trials | not serious^o^ | not serious^p^ | serious^q^ | very serious^r^ | none | Between-group MD (95% CI) of within-group MDs: -6.85 (-16.82 to 3.11) (46 participants total) | | | | ⨁◯◯◯ Very low | CRITICAL |
|  | | | | | | | | | | | | |
|  | | | | | | | | | | | | |
| **Pain (people with radicular leg pain, high-income country) (follow-up: closest to 3 months; assessed with: VAS, 0-100; benefit indicated by lower values)** | | | | | | | | | | | | |
| 1^8,l,m,n^ | randomised trials | not serious^o^ | not serious^p^ | serious^q^ | very serious^r^ | none | Between-group MD (95% CI) of within-group MDs: -6.06 (-18.50 to 6.38) (46 participants total) | | | | ⨁◯◯◯ Very low | CRITICAL |
| **Pain (people with radicular leg pain, high-income country) (follow-up: closest to 6 months; assessed with: VAS, 0-100; benefit indicated by lower values)** | | | | | | | | | | | | |
| 1^8,l,m,n^ | randomised trials | not serious^o^ | not serious^p^ | serious^q^ | very serious^r^ | none | Between-group MD (95% CI) of within-group MDs: -7.01 (-17.50 to 3.48) (46 participants total) | | | | ⨁◯◯◯ Very low | CRITICAL |
| **Pain stratified by gender** | | | | | | | | | | | | |
| 0 |  |  |  |  |  |  |  |  |  |  |  | CRITICAL |
| **Pain stratified by race/ethnicity** | | | | | | | | | | | | |
| 0 |  |  |  |  |  |  |  |  |  |  |  | CRITICAL |
| **Pain in people in low to lower-middle income countries** | | | | | | | | | | | | |
| 0 |  |  |  |  |  |  |  |  |  |  |  | CRITICAL |
| **Function (people with radicular leg pain, high-income country) (follow-up: closest to 2 weeks; assessed with: ODI, 0-100; benefit indicated by lower values)** | | | | | | | | | | | | |
| 1^8,l,m,n^ | randomised trials | not serious^o^ | not serious^p^ | serious^q^ | very serious^r^ | none | Between-group MD (95% CI) of within-group MDs: -4.52 (-13.05 to 4.01) (46 participants total) | | | | ⨁◯◯◯ Very low | CRITICAL |
| **Function (people with radicular leg pain, high-income country) (follow-up: closest to 3 months; assessed with: ODI, 0-100; benefit indicated by lower values)** | | | | | | | | | | | | |
| 1^8,l,m,n^ | randomised trials | not serious^o^ | not serious^p^ | serious^q^ | very serious^r^ | none | Between-group MD (95% CI) of within-group MDs: -3.04 (-12.34 to 6.25) (46 participants total) | | | | ⨁◯◯◯ Very low | CRITICAL |
| **Function (people with radicular leg pain, high-income country) (follow-up: closest to 6 months; assessed with: ODI, 0-100; benefit indicated by lower values)** | | | | | | | | | | | | |
| 1^8,l,m,n^ | randomised trials | not serious^o^ | not serious^p^ | serious^q^ | very serious^r^ | none | Between-group MD (95% CI) of within-group MDs: 0.09 (-10.80 to 10.98) (46 participants total) | | | | ⨁◯◯◯ Very low | CRITICAL |
| **Function stratified by gender** | | | | | | | | | | | | |
| 0 |  |  |  |  |  |  |  |  |  |  |  | CRITICAL |
| **Function stratified by race/ethnicity** | | | | | | | | | | | | |
| 0 |  |  |  |  |  |  |  |  |  |  |  | CRITICAL |
|  | | | | | | | | | | | | |
| **Function in people in low to lower-middle income countries** | | | | | | | | | | | | |
| 0 |  |  |  |  |  |  |  |  |  |  |  | CRITICAL |
| **Health-related quality of life (people with radicular leg pain, high-income country) (follow-up: closest to 2 weeks; assessed with: SF-36, 0-100; benefit indicated by higher values)** | | | | | | | | | | | | |
| 1^8,l,m,n^ | randomised trials | not serious^o^ | not serious^p^ | serious^q^ | very serious^r^ | none | No improvement in needling therapies versus sham group (46 participants total) | | | | ⨁◯◯◯ Very low | CRITICAL |
| **Health-related quality of life stratified by gender** | | | | | | | | | | | | |
| 0 |  |  |  |  |  |  |  |  |  |  |  | CRITICAL |
| **Health-related quality of life stratified by race/ethnicity** | | | | | | | | | | | | |
| 0 |  |  |  |  |  |  |  |  |  |  |  | CRITICAL |
| **Health-related quality of life in people in low to lower-middle income countries** | | | | | | | | | | | | |
| 0 |  |  |  |  |  |  |  |  |  |  |  | CRITICAL |
| **Adverse events/harms (people with radicular leg pain, high-income country)** | | | | | | | | | | | | |
| 1^8,l,m^ | randomised trials | not serious^o^ | not serious^p^ | serious^q^ | very serious^r^ | none | No serious adverse events occurred during 4-week trial; 2 of 46 participants total (4.3%) had subcutaneous hematoma after needling (both from needling therapies group) (46 participants total) | | | | ⨁◯◯◯ Very low | CRITICAL |
| **Adverse events/harms stratified by gender** | | | | | | | | | | | | |
| 0 |  |  |  |  |  |  |  |  |  |  |  | CRITICAL |
| **Adverse events/harms stratified by race/ethnicity** | | | | | | | | | | | | |
| 0 |  |  |  |  |  |  |  |  |  |  |  | CRITICAL |
| **Adverse events/harms in people in low to lower-middle income countries** | | | | | | | | | | | | |
| 0 |  |  |  |  |  |  |  |  |  |  |  | CRITICAL |
| **Psychological functioning (i.e., depression, fear avoidance, anxiety, catastrophizing, self-efficacy)** | | | | | | | | | | | | |
| 0 |  |  |  |  |  |  |  |  |  |  |  | CRITICAL |
|  | | | | | | | | | | | | |
| **Change in use of medications** | | | | | | | | | | | | |
| 0 |  |  |  |  |  |  |  |  |  |  |  | CRITICAL |
| **Falls** | | | | | | | | | | | | |
| 0 |  |  |  |  |  |  |  |  |  |  |  | CRITICAL |

**BDI:** Beck Depression Inventory; **BPI:** Brief Pain Inventory; **CI:** confidence interval; **MD:** mean difference; **MCS:** Mental Component Summary; **n/a:** not applicable; **OR:** odds ratio; **NRS:** numerical rating scale; **ODI:** Oswestry Disability Index; **OIS:** Optimal Information Size; **PCS:** Physical Component Summary; **RMDQ:** Roland Morris Disability Questionnaire; **SF-36:** Short Form Health Survey – 36-item; **SMD:** standardised mean difference; **TCM:** Traditional Chinese Medicine; **VAS:** Visual Analogue Scale

The following was used to guide the ratings.

**Risk of bias:** *Not serious:* all or most of the weight (>50%) comes from overall low risk of bias trial(s). *Serious:* some of the weight (<50%) comes from overall low risk of bias trial(s). *Very serious:* all or most of the weight (>50%) comes from overall high or unclear risk of bias trial(s).

**Inconsistency:** *Not serious:* high extent of similarity of point estimates and overlap of confidence intervals; statistical heterogeneity (I^2^) is between 0% and 40%, which might not be important. *Serious:* some extent of similarity of point estimates and overlap of confidence intervals; statistical heterogeneity (I^2^) is between 30% and 60%, which could not be explained due to small subgroups and may represent moderate heterogeneity. *Very serious:* little or no similarity of point estimates and overlap of confidence intervals; statistical heterogeneity (I^2^) is between 50% and 90% or 75% and 100%, which could not be explained due to small subgroups and may represent substantial or considerable heterogeneity, respectively.

**Indirectness:** *Not serious:* trial(s) were conducted in different countries or settings. *Serious:* trial(s) were conducted from a single country/setting. *Very serious:* evidence is not directly related to PICO question.

**Imprecision:** *Not serious:* Optimal Information Size (OIS) was reached (i.e., sample sizes with at least 200 participants per group may provide prognostic balance); and the entire confidence interval lies on one side of the threshold that may be considered clinically important (≥10% scale range or SMD ≥0.2 for continuous variables, ≥10% for binary variables), such that the clinical course of action would not differ if the upper versus the lower boundary of the confidence interval represented the truth. *Serious:* OIS would not have been reached (sample sizes with less than 200 participants per group); if the OIS was reached, the clinical course of action might differ if the upper versus the lower boundary of the confidence interval represented the truth. *Very serious:* similar to ‘serious’ but to a greater extent (e.g., very small sample sizes and confidence intervals crossing appreciable benefit and harm).

**Other considerations:** *Not serious:* Publication bias is undetected. *Serious/very serious:* Publication bias is strongly suspected.

#### Explanations

a. Yu 2020 assessed two comparisons (both included in meta-analysis).

b. Two trials were not included in the meta-analysis because they reported within-group change scores. Huang 2019: 46 participants total, rated as overall low risk of bias. Needling therapies made little or no difference to back pain: between-group MD of within-group MDs: -6.85, 95% CI -16.82 to 3.11 (VAS 0-100). Ushinohama 2016: 80 participants total; rated as overall high risk of bias. Small statistically significant difference between groups for median change in pain (p=0.032; effect size=0.21) favouring needling therapies.

c. Risk of bias: We downgraded twice because most of the weight (>50%) comes from high or unclear (i.e., some concerns) risk of bias trials.

d. Inconsistency: We did not down grade. The point estimates are similar with overlapping confidence intervals; statistical heterogeneity is between 0% and 40%, which might not be important (i.e., I2 = 9%).

e. Indirectness: We did not downgrade because the trials were conducted in different countries (high or upper-middle income).

f. Imprecision: We did not downgrade. The point estimate did not reach the pre-specified threshold for what may be considered clinically important (MD ≥ 1). The confidence interval does not cross the null or the boundary for what may be considered appreciable benefit (MD = -1).

g. One trial was not included in the meta-analysis because it only reported a within-group change score (Ushinohama 2016: 80 participants total; rated as overall high risk of bias). Small statistically significant difference between groups for median change in pain (p=0.032; effect size=0.21) favouring needling therapies.

h. Inconsistency: We downgraded twice. There is some similarity between confidence intervals and overlapping confidence intervals; statistical heterogeneity is between 50% and 90% (i.e., I2 = 69%). This could not be explained due to small subgroups and may represent substantial heterogeneity.

i. Imprecision: We downgraded once. The sample size is small (OIS would not have been achieved).

j. Risk of bias: We downgraded once because some of the weight (<50%) comes from high or unclear (i.e., some concerns) risk of bias studies.

k. Inconsistency: We did not downgrade. There is similarity between some or all point estimates and confidence intervals overlap; statistical heterogeneity is between 0% and 40%, which might not be important (i.e., I2 = 0%).

l. Treated with needling therapies type TCM.

m. Treated with needling therapies with manual stimulation.

n. Huang 2019 did not report follow-up scores (compared within-group changes between the 2 groups).

o. Risk of bias: We did not downgrade because all of the weight comes from low risk of bias trials.

p. Inconsistency: We did not downgrade; however, there are no other trials with which to compare findings.

q. Indirectness: We downgraded once; trial(s) conducted in one country (high or upper-middle income).

r. Imprecision: We downgraded twice. The sample size is small (OIS would not have been achieved).

s. Inconsistency: We did not downgrade. Some or all of the point estimates are similar with overlapping confidence intervals; statistical heterogeneity is between 0% and 40%, which might not be important (i.e., I2 = 18%).

t. Risk of bias: We downgraded twice because all of the weight comes from high or unclear (i.e., some concerns) risk of bias trials.

u. Inconsistency: We did not downgrade because statistical heterogeneity is between 0% and 40%, which might not be important (i.e., I2 = 32%).

v. One trial was not included in the meta-analysis because it reported a within-group change score (Huang 2019: 46 participants total; rated as overall low risk of bias). Needling therapies made little or no difference to back pain: between-group MD of within-group MDs: -6.85, 95% CI -16.82 to 3.11 (VAS 0-100).

w. Inconsistency: We did not downgrade. The point estimates are similar with overlapping confidence intervals; statistical heterogeneity is between 0% and 40%, which might not be important (i.e., I2 = 31%).

x. Risk of bias: We did not downgrade because most of the weight (>50%) comes from low risk of bias trials.

y. Inconsistency: We downgraded once. The point estimates are similar with overlapping confidence intervals; statistical heterogeneity is between 30% and 60% (i.e., I2 = 52%). This could not be explained due to small subgroups and may represent moderate heterogeneity.

z. Imprecision: We downgraded once. The point estimate did not reach the pre-specified threshold for what may be considered clinically important (MD ≥ 1). The confidence interval crosses the null. The lower boundary crosses the threshold for what may be considered appreciable benefit (-1).

aa. Cherkin 2009 assessed two comparisons (both included in meta-analysis).

ab. Kim 2020 assessed two comparisons (both included in meta-analysis).

ac. Two trials were not included in the meta-analysis because they included within-group change scores. Huang 2019: 46 participants total, rated as overall low risk of bias. Needling therapies made little or no difference to back pain: between-group MD of within-group MDs: -6.06 (-18.50 to 6.38) (VAS 0-100). Kong 2020: 121 participants total, rated as overall high risk of bias. No statistically significant difference between groups for mean change from baseline.

ad. Inconsistency: We downgraded twice. The point estimates vary and have some non-overlapping confidence intervals; statistical heterogeneity is between 50% and 90% (i.e., I2 = 68%). This could not be explained due to small subgroups and may represent substantial heterogeneity.

ae. Imprecision: We did not downgrade. The point estimate did not reach the threshold for what may be considered clinically important (MD ≥ 1). The confidence interval crosses the null but not the boundaries for appreciable benefit (MD = -1) or harm (MD = +1).

af. One trial was not included in the meta-analysis because it included a within-group change score. Kong 2020: 121 participants total, rated as high overall risk of bias. No statistically significant difference between groups for mean change from baseline.

ag. Inconsistency: We downgraded twice. The point estimates vary and have some non-overlapping confidence intervals; statistical heterogeneity is between 75% and 100% (i.e., I2 = 78%). This could not be explained due to small subgroups and may represent considerable heterogeneity.

ah. Imprecision: We downgraded once. The point estimate did not reach the pre-specified threshold for what may be considered clinically important (MD ≥ 1). The confidence interval does not cross the null; the lower boundary crosses the threshold for what may be considered appreciable benefit (MD = -1).

ai. One trial was not included in the meta-analysis because it reported a within-group change score (Huang 2019: 46 participants total; rated as overall low risk of bias). Needling therapies made little or no difference to back pain: between-group MD of within-group MDs: -6.06 (-18.50 to 6.38) (VAS 0-100).

aj. Inconsistency: We downgraded once. The point estimates vary and have some overlapping confidence intervals; statistical heterogeneity is between 30% and 60% (i.e., I2 = 45%). This could not be explained due to small subgroups and may represent moderate heterogeneity.

ak. Inconsistency: We did not downgrade. There is similarity between some point estimates and overlapping confidence intervals; statistical heterogeneity is between 0% and 40%, which might not be important (i.e., I2 = 28%).

al. Inconsistency: We downgraded twice. The point estimates vary and have some non-overlapping confidence intervals; statistical heterogeneity is between 75% and 100% (i.e., I2 = 83%). This could not be explained due to small subgroups and may represent considerable heterogeneity.

am. Risk of bias: We downgraded twice because most of the weight (>50%) comes from unclear (i.e., some concerns) risk of bias studies.

an. Inconsistency: We downgraded twice. The point estimates vary and have some non-overlapping confidence intervals. Statistical heterogeneity is between 75% and 100% (i.e., I2 = 82%); this could not be explained due to small subgroups and may represent considerable heterogeneity.

ao. One trial was not included in the meta-analysis because it reported a within-group change score (Huang 2019: 46 participants total; rated as overall low risk of bias). Needling therapies made little or no difference to back pain: between-group MD of within-group MDs: -7.01 (-17.50 to 3.48) (VAS 0-100).

ap. Inconsistency: We did not downgrade. The point estimates are similar with overlapping confidence intervals; statistical heterogeneity is between 0% and 40%, which might not be important (i.e., I2 = 27%).

aq. Inconsistency: We downgraded once. There is some similarity between point estimates and overlapping confidence intervals. Statistical heterogeneity is between 30% and 60% (i.e., I2 = 44%); this could not be explained due to small subgroups and may represent moderate heterogeneity.

ar. Inconsistency: We did not downgrade. There is similarity between point estimates and overlapping confidence intervals. Statistical heterogeneity is between 0% and 40%, which might not be important (i.e., I2 = 16%).

as. Two trials were not included in the meta-analysis because they included within-group change scores. Huang 2019: 46 participants total, rated as overall low risk of bias. No significant difference between groups for mean change from baseline. Kong 2020: 121 participants total, rated as overall high risk of bias. No statistically significant difference between groups for mean change from baseline.

at. Inconsistency: We downgraded once. There is some similarity between point estimates and overlapping confidence intervals. Statistical heterogeneity is between 50% and 90% (i.e., I2 = 66%). This could not be explained due to small subgroups and may represent substantial heterogeneity.

au. Imprecision: We downgraded once. The point estimate reached the pre-specified threshold for what may be considered clinically important (SMD ≥ 0.2). The confidence interval crosses the null.

av. Inconsistency: We downgraded once. The point estimates differ with overlapping confidence intervals. Statistical heterogeneity is between 30% and 60% (i.e., I2 = 42%); this could not be explained due to small subgroups and may represent moderate heterogeneity.

aw. Imprecision: We downgraded twice. The point estimate did not reach the pre-specified threshold for what may be considered clinically important (SMD ≥ 0.2). The lower boundary of the 95% CI crosses the threshold for what may be considered appreciable benefit (-0.2), and the upper boundary crosses the threshold for what may be considered appreciable harm (+0.2).

ax. One trial was not included in the meta-analysis because it reported a within-group change score (Huang 2019: 46 participants total; rated as overall low risk of bias). No significant difference between groups for mean change from baseline.

ay. Inconsistency: We downgraded twice. The point estimates vary with little overlap in confidence intervals. Statistical heterogeneity is between 75% and 100% (i.e., I2 = 84%); this could not be explained due to small subgroups and may represent considerable heterogeneity.

az. Inconsistency: We did not downgrade. The point estimates are similar with overlapping confidence intervals. Statistical heterogeneity is between 0% and 40%, which might not be important (i.e., I2 = 40%).

ba. Inconsistency: We downgraded twice. The point estimates vary with little overlap in confidence intervals. Statistical heterogeneity is between 75% and 100% (i.e., I2 = 77%); this could not be explained due to small subgroups and may represent considerable heterogeneity.

bb. Imprecision: We downgraded twice. The point estimate reached the pre-specified threshold for what may be considered clinically important (SMD ≥ 0.2). The upper boundary of the 95% CI crosses the threshold for what may be considered appreciable harm (+0.2).

bc. Inconsistency: We did not downgrade. There is some similarity in point estimates and overlapping confidence intervals. Statistical heterogeneity is between 0% and 40%, which might not be important (i.e., I2 = 38%).

bd. Imprecision: We did not downgrade. The point estimate did not reach the pre-specified threshold for what may be considered clinically important (SMD ≥ 0.2). The upper and lower boundaries of the 95% CI do not cross the threshold for what may be considered appreciable benefit (-0.2) or harm (+0.2).

be. Imprecision: We downgraded once. The point estimate did not reach the pre-specified threshold for what may be considered clinically important (SMD ≥ 0.2). The upper boundary of the 95% CI crosses the threshold for what may be considered appreciable harm (+0.2), but the lower boundary does not cross the threshold for what may be considered appreciable benefit (-0.2).

bf. Imprecision: We downgraded once. The point estimate did not reach the pre-specified threshold for what may be considered clinically important (SMD ≥ 0.2). The lower boundary of the 95%

CI crosses the threshold for what may be considered appreciable benefit (-0.2), but the upper boundary does not cross the threshold for what may be considered appreciable harm (+0.2).

bg. Inconsistency: We downgraded once. There is some similarity between point estimates and overlapping confidence intervals. Statistical heterogeneity is between 30% and 60% (i.e., I2 = 51%). This could not be explained due to small subgroups and may represent moderate heterogeneity.

bh. Imprecision: We did not downgrade. The point estimate did not reach the pre-specified threshold for what may be considered clinically important (SMD ≥ 0.2). The upper and lower boundaries of the 95% CI do not cross the threshold for what may be considered appreciable benefit (-0.2) or harm (+0.2).

bi. Inconsistency: We did not downgrade. There is some similarity between point estimates and overlapping confidence intervals. Statistical heterogeneity is between 0% and 40%, which might not be important (i.e., I2 = 31%).

bj. Inconsistency: We downgraded once. There is some similarity between point estimates and overlapping confidence intervals. Statistical heterogeneity is between 30% and 60% (i.e., I2 = 46%); this could not be explained due to small subgroups and may represent moderate heterogeneity.

bk. Imprecision: We downgraded twice. The point estimate did not reach the pre-specified threshold for what may be considered clinically important (SMD ≥ 0.2). The lower boundary of the 95% CI crosses the threshold for what may be considered appreciable benefit (-0.2), and the upper boundary crosses the threshold for what may be considered appreciable harm (+0.2).

bl. Imprecision: We did not downgrade. The point estimate reached the threshold for what may be considered appreciable benefit (SMD ≥ 0.2). The confidence interval does not cross the null.

bm. Risk of bias: We downgraded once because some of the weight (<50%) comes from unclear (i.e., some concerns) risk of bias trials.

bn. One trial was not included in the meta-analysis because it reported a within-group change score (Huang 2019: 46 participants total; rated as overall low risk of bias). No significant difference between groups for mean change from baseline on any of the subscales.

bo. Cho 2013: Participants had an unknown presence of leg pain, and received needling therapies type TCM with manual stimulation. The trial did not stratify results based on gender, age, or race/ethnicity.

bp. Inconsistency: We downgraded twice. The point estimates varied with little overlap in the confidence intervals. Statistical heterogeneity is between 50% and 90% (i.e., I2 = 74%); this could not be explained due to small subgroups and may represent substantial heterogeneity.

bq. Imprecision: We downgraded once. The point estimate reached the pre-specified threshold for what may be considered clinically important (SMD ≥ 0.2). The confidence interval crosses the null.

br. Imprecision: We downgraded once. The point estimate did not reach the pre-specified threshold for what may be considered clinically important (SMD ≥ 0.2). The upper boundary of the 95% CI crosses the threshold for what may be considered appreciable benefit (+0.2), but the lower boundary does not cross the threshold for what may be considered appreciable harm (-0.2).

bs. One trial was not included in the meta-analysis due to missing data (Cherkin 2009: 638 participants total, rated as overall unclear risk of bias). Clinically unimportant (MD<10, scale 0-100) but statistically significant difference between groups for mean change in PCS and MCS (p<0.001) favouring needling therapies.

bt. Imprecision: We did not downgrade. The point estimate did not reach the pre-specified threshold for what may be considered clinically important (SMD ≥ 0.2). The upper and lower boundaries of the 95% CI do not cross the threshold for what may be considered appreciable benefit (+0.2) or harm (-0.2).

bu. Imprecision: We downgraded twice. The point estimate did not reach the pre-specified threshold for what may be considered clinically important (SMD ≥ 0.2). The lower boundary of the 95% CI crosses the threshold for what may be considered appreciable harm (-0.2), and the upper boundary crosses the threshold for what may be considered appreciable benefit (+0.2).

bv. Inconsistency: We downgraded twice. The point estimates differed with little overlap in confidence intervals. Statistical heterogeneity is between 50% and 90% (i.e., I2 = 70%). This could not be explained due to small subgroups and may represent substantial heterogeneity.

bw. Three trials were not included in the meta-analysis due to missing data. Cho 2013 (ID#: 2002): 130 participants total, rated as overall unclear risk of bias. Authors reported no serious events; 10 minor to moderate adverse events in needling therapies group (none persisted more than 1 week): pain; bruising at needling therapies site; pain, numbness or other bothersomeness in leg; shoulder pain. Haake 2007 (ID#: 2003): 774 participants total, rated as overall low risk of bias. Authors reported 476 clinically relevant adverse effects by 257 patients (22.6%) with no significant difference between groups. Molsberger 2002 (ID#: 2007): 186 participants total, rated as overall high risk of bias. Authors reported no important adverse events or side effects were observed in any group.

bx. Minor adverse events: Brinkhaus 2006: hematoma, bleeding in both groups. Cherkin 2009: mostly short-term pain with individualized or standardized needling therapies (1 participant reported pain lasting 1 month). Huang 2019: subcutaneous hematoma after needling therapies. Kong 2020: minor pain, bruising, skin rash, and slight bleeding at needle site; mild reaction to prone position included nausea, dizziness, and mild back ache in both groups. Koppenhaver 2021: pain during treatment, dizziness, unspecified emotional change. Yuan 2016: transient worsening back pain, needling therapies point bruise, back and leg numbness and discomfort, shoulder pain (up to 1 week) in both groups.

by. Inconsistency: We downgraded twice. The point estimates vary with little overlap in the confidence intervals. Statistical heterogeneity is between 50% and 90% (i.e., I2 = 63%). This could not be explained due to small subgroups and may represent substantial heterogeneity.

bz. Imprecision: We downgraded once. The point estimate reached the pre-specified threshold for what may be considered clinically important (OR ≥ 1.10). The lower boundary of the 95% CI crosses the threshold for what may be considered appreciable benefit (0.90).

ca. Minor adverse events: Huang 2019: subcutaneous hematoma after needling.

cb. Risk of bias: We did not downgrade because all of the weight comes from low risk of bias trials.

cc. Minor adverse events: Cherkin 2009: mostly short-term pain with individualized or standardized needling therapies (1 participant reported pain lasting 1 month).

cd. Molsberger 2002 (ID#: 2007) was not included in meta-analysis due to missing data, 186 participants total, rated as overall high risk of bias. Authors reported no important adverse events or side effects were observed in any group.

ce. Minor adverse events: Brinkhaus 2006: hematoma, bleeding in both groups. Kong 2020: minor pain, bruising, skin rash, and slight bleeding at needle site; mild reaction to prone position included nausea, dizziness, and mild back ache in both groups. Koppenhaver 2021: pain during treatment, dizziness, unspecified emotional change. Yuan 2016: transient worsening back pain, needling therapies point bruise, back and leg numbness and discomfort, shoulder pain (up to 1 week) in both groups.

cf. Minor adverse events: Cherkin 2009: mostly short-term pain with individualized or standardized needling therapies (1 participant reported pain lasting 1 month). Huang 2019: subcutaneous hematoma after needling therapies. Yuan 2016: transient worsening back pain, needling therapies point bruise, back and leg numbness and discomfort, shoulder pain (up to 1 week) in both groups.

cg. Inconsistency: We downgraded once. There is some similarity between point estimates and overlapping confidence intervals. Statistical heterogeneity is between 30% and 60% (i.e., I2 = 57%). This could not be explained due to small subgroups and may represent moderate heterogeneity.

ch. Minor adverse events: Koppenhaver 2021: pain during treatment, dizziness, unspecified emotional change.

ci. Minor adverse events: Brinkhaus 2006: hematoma, bleeding in both groups. Cherkin 2009: mostly short-term pain with individualized or standardized needling therapies (1 participant reported pain lasting 1 month). Kong 2020: minor pain, bruising, skin rash, and slight bleeding at needle site; mild reaction to prone position included nausea, dizziness, and mild back ache in both groups.

cj. Inconsistency: We downgraded twice. The point estimates are in different directions with no overlap in confidence intervals. Statistical heterogeneity is between 50% and 90% (i.e., I2 = 89%). This could not be explained due to small subgroups and may represent substantial heterogeneity.

ck. Two studies were not included in the meta-analysis due to missing data. Cho 2013 (ID#: 2002): 130 participants total, rated as overall unclear risk of bias, authors reported no serious events; 10 minor to moderate adverse events in needling therapies group (none persisted more than 1 week) including pain, bruising at needling therapies site. Molsberger 2002 (ID#: 2007): 186 participant total, rated as overall high risk of bias, authors reported no important adverse events or side effects were observed in any group.

cl. Minor adverse events: Brinkhaus 2006: hematoma, bleeding in both groups. Koppenhaver 2021: pain during treatment, dizziness, unspecified emotional change. Yuan 2016: transient worsening back pain, needling therapies point bruise, back and leg numbness and discomfort, shoulder pain (up to 1 week) in both groups.

cm. Imprecision: We downgraded once. The point estimate reached the pre-specified threshold for what may be considered clinically important (OR ≥ 0.90). The upper boundary of the 95% CI crosses the threshold for what may be considered appreciable harm (1.10), but the lower boundary does not cross the threshold for what may be considered appreciable harm (0.90).

cn. Minor adverse events: Kong 2020: minor pain, bruising, skin rash, and slight bleeding at needle site; mild reaction to prone position included nausea, dizziness, and mild back ache in both groups.

co. One trial was not included in the meta-analysis due to missing data. Haake 2007 (ID#: 2003): 774 participants total, rated as overall low risk of bias; authors reported 476 clinically relevant adverse effects by 257 patients (22.6%) with no significant difference between groups.

#### References

1.Yuan Q, Liu L,Ma J,Wu W,Ye M,Zhang Y. [A clinical study of acupuncture therapy for treatment of chronic nonspecific low back pain] . 2016.

2.Yu S, Ortiz A,Gollub RL,et al. Acupuncture treatment modulates the connectivity of key regions of the descending pain modulation and reward systems in patients with chronic low back pain. 2020.

3.Molsberger AF, Mau J,Pawelec DB,Winkler J. Does acupuncture improve the orthopedic management of chronic low back pain--a randomized, blinded, controlled trial with 3 months follow up. 2002.

4.Martín-Corrales C, Bautista IV,Méndez-Mera JE,et al. Benefits of Adding Gluteal Dry Needling to a Four-Week Physical Exercise Program in a Chronic Low Back Pain Population. A Randomized Clinical Trial. 2020.

5.Koppenhaver SL, Weaver AM,Randall TL,et al. Effect of dry needling on lumbar muscle stiffness in patients with low back pain: a double blind, randomized controlled trial using shear wave elastography. . 2021.

6.Kerr DP, Walsh DM,Baxter D. Acupuncture in the management of chronic low back pain: a blinded randomized controlled trial. 2003.

7.Haake M, Muller HH,Schade-Brittinger C,et al.. German Acupuncture Trials (GERAC) for chronic low back pain: randomized, multicenter, blinded, parallel-group trial with 3 groups. 2007.

8.Huang Z, Liu S,Zhou J,Yao Q,Liu Z. Efficacy and Safety of Acupuncture for Chronic Discogenic Sciatica, a Randomized Controlled Sham Acupuncture Trial. 2019.

9.Brinkhaus B, Witt CM,Jena S,et al. Acupuncture in patients with chronic low back pain: a randomized controlled trial. 2006.

10.Cherkin DC, Sherman KJ,Avins AL,et al. A randomized trial comparing acupuncture, simulated acupuncture, and usual care for chronic low back pain. 2009.

11.Cho YJ, Song YK,Cha YY,et al. Acupuncture for chronic low back pain: a multicenter, randomized, patient-assessor blind, sham-controlled clinical trial. 2013.

12.de Castro Moura C, de Cássia Lopes Chaves E,Couto Machado Chianca T,Ruginsk SG,Alves Nogueira D,Iunes DH. Effects of auricular acupuncture on chronic pain in people with back musculoskeletal disorders: a randomized clinical trial. 2019.

13.Kim H, Mawla I,Lee J,et al. Reduced tactile acuity in chronic low back pain is linked with structural neuroplasticity in primary somatosensory cortex and is modulated by acupuncture therapy. 2020.

14.Kong JT, Puetz C,Tian L,et al. Effect of Electroacupuncture vs Sham Treatment on Change in Pain Severity Among Adults With Chronic Low Back Pain: a Randomized Clinical Trial. 2020.

15. Ushinohama A, et al. Effect of a single session of ear acupuncture on pain intensity and postural control in individuals with chronic low back pain: a randomized controlled trial. 2016.

GRADE evidence profile table 2: ***What are the benefits and harms of needling therapies in the management of community-dwelling adults (including older adults aged 60 years and over) with chronic primary low back pain (with or without leg pain) compared to no intervention or interventions where the effect of needling therapies could be isolated?***

| **Certainty assessment** | | | | | | | **№ of patients** | | **Effect** | | **Certainty** | **Importance** |
| --- | --- | --- | --- | --- | --- | --- | --- | --- | --- | --- | --- | --- |
| **№ of studies** | **Study design** | **Risk of bias** | **Inconsistency** | **Indirectness** | **Imprecision** | **Other considerations** | **Needling therapies** | **No treatment** | **Relative (95% CI)** | **Absolute (95% CI)** |
| **ALL ADULTS** | | | | | | | | | | | | |
| **Pain (follow-up: closest to 2 weeks; assessed with: VAS, NRS, Pain Scale; benefit indicated by lower values; Scale from: 0 to 10)** | | | | | | | | | | | | |
| 21^1,2,3,4,5,6,7,8,9,10,11,12,13,14,15,16,17,18,19,20,21,a,b^ | randomised trials | very serious^c^ | not serious^d^ | not serious^e^ | not serious^f^ | none | 859 | 858 | - | MD **1.21 lower** (1.5 lower to 0.92 lower) | ⨁⨁◯◯ Low | CRITICAL |
| **Pain (mixed females and males) (follow-up: closest to 2 weeks; assessed with: VAS, NRS, Pain Scale; benefit indicated by lower values; Scale from: 0 to 10)** | | | | | | | | | | | | |
| 19^1,2,3,4,6,7,8,9,10,11,12,13,14,15,17,18,19,20,21,b^ | randomised trials | very serious^c^ | not serious^d^ | not serious^e^ | not serious^f^ | none | 800 | 799 | - | MD **1.22 lower** (1.48 lower to 0.97 lower) | ⨁⨁◯◯ Low | CRITICAL |
| **Pain in males (follow-up: closest to 2 weeks; assessed with: VAS; benefit indicated by lower values; Scale from: 0 to 10)** | | | | | | | | | | | | |
| 1^16,a^ | randomised trials | very serious^c^ | not serious^g^ | serious^h^ | very serious^i^ | none | 40 | 40 | - | MD **1.99 lower** (2.86 lower to 1.12 lower) | ⨁◯◯◯ Very low | CRITICAL |
|  | | | | | | | | | | | | |
|  | | | | | | | | | | | | |
|  | | | | | | | | | | | | |
| **Pain in people (gender not reported) (follow-up: closest to 2 weeks; assessed with: VAS; benefit indicated by lower values; Scale from: 0 to 10)** | | | | | | | | | | | | |
| 1^5^ | randomised trials | very serious^c^ | not serious^g^ | serious^h^ | very serious^i^ | none | 19 | 19 | - | MD **0.3 higher** (0.1 higher to 0.5 higher) | ⨁◯◯◯ Very low | CRITICAL |
| **Pain in people without leg pain (follow-up: closest to 2 weeks; assessed with: VAS, Pain Scale; benefit indicated by lower values; Scale from: 0 to 10)** | | | | | | | | | | | | |
| 8^1,2,3,4,10,16,20,21,a^ | randomised trials | very serious^c^ | not serious^d^ | not serious^e^ | not serious^f^ | none | 272 | 271 | - | MD **1.83 lower** (2.76 lower to 0.91 lower) | ⨁⨁◯◯ Low | CRITICAL |
| **Pain in people with radicular leg pain (follow-up: closest to 2 weeks; assessed with: VAS; benefit indicated by lower values; Scale from: 0 to 10)** | | | | | | | | | | | | |
| 6^6,12,13,15,17,18^ | randomised trials | very serious^c^ | not serious^d^ | not serious^j^ | not serious^k^ | none | 257 | 257 | - | MD **0.75 lower** (0.95 lower to 0.55 lower) | ⨁⨁◯◯ Low | CRITICAL |
| **Pain in people either with or without leg pain (follow-up: closest to 2 weeks; assessed with: VAS, NRS; benefit indicated by lower values; Scale from: 0 to 10)** | | | | | | | | | | | | |
| 3^7,11,14^ | randomised trials | very serious^c^ | not serious^d^ | not serious^e^ | serious^l^ | none | 181 | 181 | - | MD **1.32 lower** (1.49 lower to 1.16 lower) | ⨁◯◯◯ Very low | CRITICAL |
|  | | | | | | | | | | | | |
|  | | | | | | | | | | | | |
|  | | | | | | | | | | | | |
| **Pain in people with unclassified presence of leg pain (follow-up: closest to 2 weeks; assessed with: VAS, NRS; benefit indicated by lower values; Scale from: 0 to 10)** | | | | | | | | | | | | |
| 4^5,8,9,19,b^ | randomised trials | very serious^c^ | serious^m^ | not serious^e^ | serious^l^ | none | 149 | 149 | - | MD **0.68 lower** (1.44 lower to 0.08 higher) | ⨁◯◯◯ Very low | CRITICAL |
| **Pain in people in high to upper-middle income countries (follow-up: closest to 2 weeks; assessed with: VAS, NRS, Pain Scale; benefit indicated by lower values; Scale from: 0 to 10)** | | | | | | | | | | | | |
| 18^1,2,3,4,6,7,8,9,10,12,13,14,15,17,18,19,20,21,b^ | randomised trials | very serious^c^ | not serious^d^ | not serious^j^ | not serious^f^ | none | 785 | 784 | - | MD **1.2 lower** (1.46 lower to 0.94 lower) | ⨁⨁◯◯ Low | CRITICAL |
| **Pain in people in low to lower-middle income countries (follow-up: closest to 2 weeks; assessed with: VAS; benefit indicated by lower values; Scale from: 0 to 10)** | | | | | | | | | | | | |
| 3^5,11,16,a^ | randomised trials | very serious^c^ | serious^n^ | not serious^o^ | very serious^i^ | none | 74 | 74 | - | MD **1.38 lower** (3.02 lower to 0.26 higher) | ⨁◯◯◯ Very low | CRITICAL |
| **Pain stratified by race/ethnicity (follow-up: closest to 2 weeks)** | | | | | | | | | | | | |
| 0 |  |  |  |  |  |  |  |  |  |  |  |  |
| **Pain in people treated with needling therapies type TCM (follow-up: closest to 2 weeks; assessed with: VAS, NRS, Pain Scale; benefit indicated by lower values; Scale from: 0 to 10)** | | | | | | | | | | | | |
| 19^1,2,3,4,6,7,8,9,10,12,13,14,15,16,17,18,19,20,21,a,b^ | randomised trials | very serious^c^ | not serious^d^ | not serious^e^ | not serious^f^ | none | 825 | 824 | - | MD **1.24 lower** (1.49 lower to 0.99 lower) | ⨁⨁◯◯ Low | CRITICAL |
|  | | | | | | | | | | | | |
| **Pain in people treated with needling therapies type myofascial (follow-up: closest to 2 weeks; assessed with: VAS; benefit indicated by lower values; Scale from: 0 to 10)** | | | | | | | | | | | | |
| 1^11^ | randomised trials | very serious^c^ | not serious^g^ | serious^h^ | very serious^i^ | none | 15 | 15 | - | MD **2.17 lower** (3.49 lower to 0.85 lower) | ⨁◯◯◯ Very low | CRITICAL |
| **Pain in people treated with needling therapies (type not reported) (follow-up: closest to 2 weeks; assessed with: VAS; benefit indicated by lower values; Scale from: 0 to 10)** | | | | | | | | | | | | |
| 1^5^ | randomised trials | very serious^c^ | not serious^g^ | serious^h^ | very serious^i^ | none | 19 | 19 | - | MD **0.3 higher** (0.1 higher to 0.5 higher) | ⨁◯◯◯ Very low | CRITICAL |
| **Pain in people treated with needling therapies with manual stimulation (follow-up: closest to 2 weeks; assessed with: VAS; benefit indicated by lower values; Scale from: 0 to 10)** | | | | | | | | | | | | |
| 8^2,6,8,9,13,17,20,21^ | randomised trials | very serious^c^ | not serious^d^ | not serious^e^ | not serious^f^ | none | 362 | 363 | - | MD **1.38 lower** (1.84 lower to 0.92 lower) | ⨁⨁◯◯ Low | CRITICAL |
| **Pain in people treated with needling therapies with electrical stimulation (follow-up: closest to 2 weeks; assessed with: VAS, NRS, Pain Scale; benefit indicated by lower values; Scale from: 0 to 10)** | | | | | | | | | | | | |
| 5^1,4,5,14,16,a^ | randomised trials | very serious^c^ | not serious^d^ | not serious^e^ | serious^l^ | none | 125 | 124 | - | MD **1.21 lower** (2.22 lower to 0.21 lower) | ⨁◯◯◯ Very low | CRITICAL |
|  | | | | | | | | | | | | |
|  | | | | | | | | | | | | |
|  | | | | | | | | | | | | |
| **Pain in people treated with needling therapies with heat stimulation (follow-up: closest to 2 weeks; assessed with: VAS; benefit indicated by lower values; Scale from: 0 to 10)** | | | | | | | | | | | | |
| 1^12^ | randomised trials | very serious^c^ | not serious^g^ | serious^p^ | very serious^i^ | none | 46 | 45 | - | MD **1.23 lower** (1.6 lower to 0.86 lower) | ⨁◯◯◯ Very low | CRITICAL |
| **Pain in people treated with needling therapies with mixed stimulation methods (follow-up: closest to 2 weeks; assessed with: VAS, NRS; benefit indicated by lower values; Scale from: 0 to 10)** | | | | | | | | | | | | |
| 4^7,15,18,19^ | randomised trials | very serious^c^ | not serious^d^ | not serious^j^ | not serious^f^ | none | 257 | 257 | - | MD **1.11 lower** (1.43 lower to 0.79 lower) | ⨁⨁◯◯ Low | CRITICAL |
| **Pain in people treated with needling therapies without stimulation (follow-up: closest to 2 weeks; assessed with: VAS; benefit indicated by lower values; Scale from: 0 to 10)** | | | | | | | | | | | | |
| 2^3,11,q^ | randomised trials | very serious^c^ | not serious^d^ | not serious^e^ | very serious^i^ | none | 50 | 50 | - | MD **1.28 lower** (2.69 lower to 0.13 higher) | ⨁◯◯◯ Very low | CRITICAL |
| **Pain in people treated with needling therapies with threading stimulation (follow-up: closest to 2 weeks; assessed with: VAS; benefit indicated by lower values; Scale from: 0 to 10)** | | | | | | | | | | | | |
| 1^10,r^ | randomised trials | very serious^c^ | not serious^g^ | serious^p^ | very serious^i^ | none | 19 | 19 | - | MD **0.78 lower** (2.16 lower to 0.6 higher) | ⨁◯◯◯ Very low | CRITICAL |
|  | | | | | | | | | | | | |
|  | | | | | | | | | | | | |
|  | | | | | | | | | | | | |
| **Pain in people after removing high risk of bias studies (follow-up: closest to 2 weeks; assessed with: VAS; benefit indicated by lower values; Scale from: 0 to 10)** | | | | | | | | | | | | |
| 2^10,20^ | randomised trials | very serious^c^ | not serious^d^ | not serious^j^ | very serious^i^ | none | 69 | 69 | - | MD **1.79 lower** (3.59 lower to 0.02 higher) | ⨁◯◯◯ Very low | CRITICAL CRITICAL |
| **Pain (follow-up: closest to 3 months; assessed with: VAS, NRS, BPI, Pain Scale; benefit indicated by lower values; Scale from: 0 to 10)** | | | | | | | | | | | | |
| 9^1,4,13,14,16,20,21,22,23,a,s^ | randomised trials | very serious^c^ | not serious^d^ | not serious^e^ | not serious^f^ | none | 420 | 342 | - | MD **1.56 lower** (2.18 lower to 0.95 lower) | ⨁⨁◯◯ Low | CRITICAL |
| **Pain (mixed females and males) (follow-up: closest to 3 months; assessed with: VAS, NRS, BPI, Pain Scale; benefit indicated by lower values; Scale from: 0 to 10)** | | | | | | | | | | | | |
| 8^1,4,13,14,20,21,22,23,s^ | randomised trials | very serious^c^ | not serious^d^ | not serious^e^ | not serious^f^ | none | 380 | 302 | - | MD **1.57 lower** (2.28 lower to 0.86 lower) | ⨁⨁◯◯ Low | CRITICAL |
| **Pain in males (follow-up: closest to 3 months; assessed with: VAS; benefit indicated by lower values; Scale from: 0 to 10)** | | | | | | | | | | | | |
| 1^16,a^ | randomised trials | very serious^c^ | not serious^g^ | serious^h^ | very serious^i^ | none | 40 | 40 | - | MD **1.54 lower** (2.48 lower to 0.61 lower) | ⨁◯◯◯ Very low | CRITICAL |
| **Pain in females (follow-up: closest to 3 months)** | | | | | | | | | | | | |
| 0 |  |  |  |  |  |  |  |  |  |  |  |  |
|  | | | | | | | | | | | | |
| **Pain stratified by race/ethnicity (follow-up: closest to 3 months)** | | | | | | | | | | | | |
| 0 |  |  |  |  |  |  |  |  |  |  |  |  |
| **Pain in people with radicular leg pain (follow-up: closest to 3 months; assessed with: VAS; benefit indicated by lower values; Scale from: 0 to 10)** | | | | | | | | | | | | |
| 1^13^ | randomised trials | very serious^c^ | not serious^g^ | serious^p^ | very serious^i^ | none | 40 | 40 | - | MD **0.61 lower** (0.91 lower to 0.31 lower) | ⨁◯◯◯ Very low | CRITICAL |
| **Pain in people without leg pain (follow-up: closest to 3 months; assessed with: VAS, Pain Scale; benefit indicated by lower values; Scale from: 0 to 10)** | | | | | | | | | | | | |
| 6^1,4,16,20,21,23,a^ | randomised trials | very serious^c^ | not serious^d^ | not serious^e^ | not serious^f^ | none | 317 | 239 | - | MD **1.89 lower** (2.55 lower to 1.22 lower) | ⨁⨁◯◯ Low | CRITICAL |
| **Pain in people either with or without leg pain (follow-up: closest to 3 months; assessed with: NRS; benefit indicated by lower values; Scale from: 0 to 10)** | | | | | | | | | | | | |
| 1^14^ | randomised trials | very serious^c^ | not serious^g^ | serious^p^ | very serious^i^ | none | 26 | 26 | - | MD **1.81 lower** (3.03 lower to 0.59 lower) | ⨁◯◯◯ Very low | CRITICAL |
| **Pain in people with unclassified presence of leg pain (follow-up: closest to 3 months; assessed with: BPI; benefit indicated by lower values; Scale from: 0 to 10)** | | | | | | | | | | | | |
| 1^22,s^ | randomised trials | very serious^c^ | not serious^g^ | serious^p^ | very serious^i^ | none | 37 | 37 | - | MD **0.05 higher** (1.4 lower to 1.5 higher) | ⨁◯◯◯ Very low | CRITICAL |
|  | | | | | | | | | | | | |
| **Pain in people in high to upper-middle income countries (follow-up: closest to 3 months; assessed with: VAS, NRS, BPI, Pain Scale; benefit indicated by lower values; Scale from: 0 to 10)** | | | | | | | | | | | | |
| 8^1,4,13,14,20,21,22,23,s^ | randomised trials | very serious^c^ | not serious^d^ | not serious^j^ | not serious^f^ | none | 380 | 302 | - | MD **1.57 lower** (2.28 lower to 0.86 lower) | ⨁⨁◯◯ Low | CRITICAL |
| **Pain in people in low to lower-middle income countries (follow-up: closest to 3 months; assessed with: VAS; benefit indicated by lower values; Scale from: 0 to 10)** | | | | | | | | | | | | |
| 1^16^ | randomised trials | very serious^c^ | not serious^g^ | serious^h^ | very serious^i^ | none | 40 | 40 | - | MD **1.54 lower** (2.48 lower to 0.61 lower) | ⨁◯◯◯ Very low | CRITICAL |
| **Pain in people treated with needling therapies type TCM (follow-up: closest to 3 months; assessed with: VAS, NRS, BPI, Pain Scale; benefit indicated by lower values; Scale from: 0 to 10)** | | | | | | | | | | | | |
| 8^1,4,13,14,16,20,21,22,a,s^ | randomised trials | very serious^c^ | not serious^d^ | not serious^e^ | not serious^f^ | none | 280 | 268 | - | MD **1.45 lower** (2.07 lower to 0.83 lower) | ⨁⨁◯◯ Low | CRITICAL |
| **Pain in people treated with needling therapies type mixed (TCM, myofascial) (follow-up: closest to 3 months; assessed with: VAS; benefit indicated by lower values; Scale from: 0 to 10)** | | | | | | | | | | | | |
| 1^23^ | randomised trials | very serious^c^ | not serious^g^ | serious^p^ | serious^l^ | none | 140 | 74 | - | MD **2.41 lower** (3.15 lower to 1.67 lower) | ⨁◯◯◯ Very low | CRITICAL |
|  | | | | | | | | | | | | |
|  | | | | | | | | | | | | |
|  | | | | | | | | | | | | |
| **Pain in people treated with needling therapies with manual stimulation (follow-up: closest to 3 months; assessed with: VAS; benefit indicated by lower values; Scale from: 0 to 10)** | | | | | | | | | | | | |
| 4^13,20,21,23^ | randomised trials | very serious^c^ | not serious^d^ | not serious^e^ | serious^t^ | none | 277 | 200 | - | MD **1.69 lower** (2.9 lower to 0.48 lower) | ⨁◯◯◯ Very low | CRITICAL |
| **Pain in people treated with needling therapies with electrical stimulation (follow-up: closest to 3 months; assessed with: VAS, NRS, Pain Scale; benefit indicated by lower values; Scale from: 0 to 10)** | | | | | | | | | | | | |
| 4^1,4,14,16,a^ | randomised trials | very serious^c^ | not serious^d^ | not serious^e^ | serious^l^ | none | 106 | 105 | - | MD **1.65 lower** (2.29 lower to 1.02 lower) | ⨁◯◯◯ Very low | CRITICAL |
| **Pain in people treated with needling therapies (no stimulation) (follow-up: closest to 3 months; assessed with: BPI; benefit indicated by lower values; Scale from: 0 to 10)** | | | | | | | | | | | | |
| 1^22,s,u^ | randomised trials | very serious^c^ | not serious^g^ | serious^p^ | very serious^i^ | none | 37 | 37 | - | MD **0.05 higher** (1.4 lower to 1.5 higher) | ⨁◯◯◯ Very low | CRITICAL |
| **Pain after removing high risk of bias studies (follow-up: closest to 3 months; assessed with: VAS; benefit indicated by lower values; Scale from: 0 to 10)** | | | | | | | | | | | | |
| 1^20^ | randomised trials | very serious^c^ | not serious^g^ | serious^p^ | very serious^i^ | none | 50 | 50 | - | MD **0.92 lower** (1.89 lower to 0.05 higher) | ⨁◯◯◯ Very low | CRITICAL |
| **Function (follow-up: closest to 2 weeks; assessed with: RMDQ, ODI, JOA, Aberdeen; benefit indicated by lower values)** | | | | | | | | | | | | |
| 19^1,2,3,4,5,6,7,8,9,10,11,12,13,14,16,17,18,19,20,a,v^ | randomised trials | very serious^c^ | not serious^w^ | not serious^e^ | not serious^f^ | none | 770 | 771 | - | SMD **1.39 lower** (2 lower to 0.77 lower) | ⨁⨁◯◯ Low | CRITICAL |
| **Function (mixed females and males) (follow-up: closest to 2 weeks; assessed with: RMDQ, ODI, JOA, Aberdeen; benefit indicated by lower values)** | | | | | | | | | | | | |
| 17^1,2,3,4,6,7,8,9,10,11,12,13,14,17,18,19,20,v^ | randomised trials | very serious^c^ | not serious^w^ | not serious^e^ | not serious^f^ | none | 711 | 712 | - | SMD **1.66 lower** (2.29 lower to 1.04 lower) | ⨁⨁◯◯ Low | CRITICAL |
| **Function in males (follow-up: closest to 2 weeks; assessed with: RMDQ; benefit indicated by lower values)** | | | | | | | | | | | | |
| 1^16,a^ | randomised trials | very serious^c^ | not serious^g^ | serious^h^ | very serious^i^ | none | 40 | 40 | - | SMD **1.01 lower** (1.48 lower to 0.55 lower) | ⨁◯◯◯ Very low | CRITICAL |
| **Function (gender not reported) (follow-up: closest to 2 weeks; assessed with: ODI; benefit indicated by lower values)** | | | | | | | | | | | | |
| 1^5^ | randomised trials | very serious^c^ | not serious^g^ | serious^h^ | very serious^i^ | none | 19 | 19 | - | SMD **2.93 higher** (1.98 higher to 3.87 higher) | ⨁◯◯◯ Very low | CRITICAL |
| **Function in people with radicular leg pain (follow-up: closest to 2 weeks; assessed with: ODI, JOA; benefit indicated by lower values)** | | | | | | | | | | | | |
| 5^6,12,13,17,18^ | randomised trials | very serious^c^ | not serious^w^ | not serious^j^ | not serious^f^ | none | 226 | 228 | - | SMD **2.03 lower** (3.05 lower to 1 lower) | ⨁⨁◯◯ Low | CRITICAL |
|  | | | | | | | | | | | | |
|  | | | | | | | | | | | | |
|  | | | | | | | | | | | | |
| **Function in people either with or without leg pain (follow-up: closest to 2 weeks; assessed with: ODI, Aberdeen; benefit indicated by lower values)** | | | | | | | | | | | | |
| 3^7,11,14^ | randomised trials | very serious^c^ | serious^x^ | not serious^e^ | very serious^y^ | none | 181 | 181 | - | SMD **1.99 lower** (4.9 lower to 0.92 higher) | ⨁◯◯◯ Very low | CRITICAL |
| **Function in people without leg pain (follow-up: closest to 2 weeks; assessed with: RMDQ, ODI, JOA; benefit indicated by lower values)** | | | | | | | | | | | | |
| 7^1,2,3,4,10,16,20,a^ | randomised trials | very serious^c^ | not serious^w^ | not serious^e^ | not serious^f^ | none | 214 | 213 | - | SMD **1.02 lower** (1.42 lower to 0.61 lower) | ⨁⨁◯◯ Low | CRITICAL |
| **Function in people with unclassified presence of leg pain (follow-up: closest to 2 weeks; assessed with: RMDQ, ODI; benefit indicated by lower values)** | | | | | | | | | | | | |
| 4^5,8,9,18,v^ | randomised trials | very serious^c^ | serious^z^ | not serious^e^ | very serious^y^ | none | 149 | 149 | - | SMD **0.8 lower** (2.74 lower to 1.15 higher) | ⨁◯◯◯ Very low | CRITICAL |
| **Function in people in high to upper-middle income countries (follow-up: closest to 2 weeks; assessed with: RMDQ, ODI, JOA, Aberdeen; benefit indicated by lower values)** | | | | | | | | | | | | |
| 16^1,2,3,4,6,7,8,9,10,12,13,14,17,18,19,20,v^ | randomised trials | very serious^c^ | not serious^w^ | not serious^j^ | not serious^f^ | none | 696 | 697 | - | SMD **1.75 lower** (2.39 lower to 1.1 lower) | ⨁⨁◯◯ Low | CRITICAL |
|  | | | | | | | | | | | | |
|  | | | | | | | | | | | | |
|  | | | | | | | | | | | | |
|  | | | | | | | | | | | | |
| **Function in people in low to lower-middle income countries (follow-up: closest to 2 weeks; assessed with: RMDQ, ODI; benefit indicated by lower values)** | | | | | | | | | | | | |
| 3^5,11,16,a^ | randomised trials | very serious^c^ | serious^aa^ | not serious^o^ | very serious^i^ | none | 74 | 74 | - | SMD **0.11 higher** (1.44 lower to 1.67 higher) | ⨁◯◯◯ Very low | CRITICAL |
| **Function stratified by race/ethnicity (follow-up: closest to 2 weeks)** | | | | | | | | | | | | |
| 0 |  |  |  |  |  |  |  |  |  |  |  | CRITICAL |
| **Function in people treated with needling therapies type TCM (follow-up: closest to 2 weeks; assessed with: RMDQ, ODI, JOA, Aberdeen; benefit indicated by lower values)** | | | | | | | | | | | | |
| 17^1,2,3,4,6,7,8,9,10,12,13,14,16,17,18,19,20,a,v^ | randomised trials | very serious^c^ | not serious^w^ | not serious^e^ | not serious^f^ | none | 736 | 737 | - | SMD **1.67 lower** (2.26 lower to 1.08 lower) | ⨁⨁◯◯ Low | CRITICAL |
| **Function in people treated with needling therapies type myofascial (follow-up: closest to 2 weeks; assessed with: ODI; benefit indicated by lower values)** | | | | | | | | | | | | |
| 1^11^ | randomised trials | very serious^c^ | not serious^g^ | serious^h^ | very serious^i^ | none | 15 | 15 | - | SMD **0.32 lower** (1.04 lower to 0.4 higher) | ⨁◯◯◯ Very low | CRITICAL |
| **Function in people treated with needling therapies (type not reported) (follow-up: closest to 2 weeks; assessed with: ODI; benefit indicated by lower values)** | | | | | | | | | | | | |
| 1^5^ | randomised trials | very serious^c^ | not serious^g^ | serious^h^ | very serious^i^ | none | 19 | 19 | - | SMD **2.93 higher** (1.98 higher to 3.87 higher) | ⨁◯◯◯ Very low | CRITICAL |
|  | | | | | | | | | | | | |
| **Function in people treated with needling therapies with manual stimulation (follow-up: closest to 2 weeks; assessed with: RMDQ, ODI, JOA; benefit indicated by lower values)** | | | | | | | | | | | | |
| 7^2,6,8,9,13,17,20^ | randomised trials | very serious^c^ | not serious^w^ | not serious^j^ | not serious^f^ | none | 304 | 305 | - | SMD **1.14 lower** (1.57 lower to 0.71 lower) | ⨁⨁◯◯ Low | CRITICAL |
| **Function in people treated with needling therapies with electrical stimulation (follow-up: closest to 2 weeks; assessed with: RMDQ, ODI, Aberdeen; benefit indicated by lower values)** | | | | | | | | | | | | |
| 5^1,4,5,14,16^ | randomised trials | very serious^c^ | serious^ab^ | not serious^e^ | very serious^y^ | none | 125 | 124 | - | SMD **0.38 lower** (1.35 lower to 0.59 higher) | ⨁◯◯◯ Very low | CRITICAL |
| **Function in people treated with needling therapies with heat stimulation (follow-up: closest to 2 weeks; assessed with: JOA; benefit indicated by lower values)** | | | | | | | | | | | | |
| 1^12^ | randomised trials | very serious^c^ | not serious^g^ | serious^p^ | very serious^i^ | none | 45 | 46 | - | SMD **3.44 lower** (4.1 lower to 2.79 lower) | ⨁◯◯◯ Very low | CRITICAL |
| **Function in people treated with needling therapies with mixed stimulation methods (follow-up: closest to 2 weeks; assessed with: ODI, JOA; benefit indicated by lower values)** | | | | | | | | | | | | |
| 3^7,18,19^ | randomised trials | very serious^c^ | not serious^w^ | not serious^j^ | not serious^f^ | none | 227 | 227 | - | SMD **3.73 lower** (4.84 lower to 2.62 lower) | ⨁⨁◯◯ Low | CRITICAL |
|  | | | | | | | | | | | | |
|  | | | | | | | | | | | | |
|  | | | | | | | | | | | | |
| **Function in people treated with needling therapies without stimulation (follow-up: closest to 2 weeks; assessed with: ODI; benefit indicated by lower values)** | | | | | | | | | | | | |
| 2^3,11,v^ | randomised trials | very serious^c^ | serious^ac^ | not serious^e^ | very serious^i^ | none | 50 | 50 | - | SMD **1.32 lower** (3.27 lower to 0.62 higher) | ⨁◯◯◯ Very low | CRITICAL |
| **Function in people treated with needling therapies with threading stimulation (follow-up: closest to 2 weeks; assessed with: RMDQ; benefit indicated by lower values)** | | | | | | | | | | | | |
| 1^10^ | randomised trials | very serious^c^ | not serious^g^ | serious^p^ | very serious^i^ | none | 19 | 19 | - | SMD **0.15 lower** (0.79 lower to 0.49 higher) | ⨁◯◯◯ Very low | CRITICAL |
| **Function after removing high risk of bias studies (follow-up: closest to 2 weeks; assessed with: RMDQ, ODI; benefit indicated by lower values)** | | | | | | | | | | | | |
| 2^10,20^ | randomised trials | very serious^c^ | serious^ad^ | not serious^j^ | very serious^i^ | none | 69 | 69 | - | SMD **0.59 lower** (1.36 lower to 0.19 higher) | ⨁◯◯◯ Very low | CRITICAL |
| **Function (follow-up: closest to 3 months; assessed with: RMDQ, ODI, JOA, BPI, Hannover, Aberdeen; benefit indicated by lower values)** | | | | | | | | | | | | |
| 8^1,4,13,14,16,20,22,23,ae,af^ | randomised trials | very serious^c^ | not serious^w^ | not serious^e^ | not serious^f^ | none | 287 | 352 | - | SMD **0.57 lower** (0.92 lower to 0.22 lower) | ⨁⨁◯◯ Low | CRITICAL |
|  | | | | | | | | | | | | |
|  | | | | | | | | | | | | |
|  | | | | | | | | | | | | |
| **Function (mixed females and males) (follow-up: closest to 3 months; assessed with: RMDQ, ODI, JOA, BPI, Hannover, Aberdeen; benefit indicated by lower values)** | | | | | | | | | | | | |
| 7^1,4,13,14,20,22,23,ae,af^ | randomised trials | very serious^c^ | not serious^w^ | not serious^j^ | not serious^f^ | none | 267 | 332 | - | SMD **0.56 lower** (0.95 lower to 0.17 lower) | ⨁⨁◯◯ Low | CRITICAL |
| **Function in males (follow-up: closest to 3 months; assessed with: RMDQ; benefit indicated by lower values)** | | | | | | | | | | | | |
| 1^16^ | randomised trials | very serious^c^ | not serious^g^ | serious^h^ | very serious^i^ | none | 20 | 20 | - | SMD **0.67 lower** (1.31 lower to 0.04 lower) | ⨁◯◯◯ Very low | CRITICAL |
| **Function in people with radicular leg pain (follow-up: closest to 3 months; assessed with: JOA; benefit indicated by lower values)** | | | | | | | | | | | | |
| 1^13^ | randomised trials | very serious^c^ | not serious^g^ | serious^p^ | very serious^i^ | none | 40 | 40 | - | SMD **1.05 lower** (1.52 lower to 0.58 lower) | ⨁◯◯◯ Very low | CRITICAL |
| **Function in people either with or without leg pain (follow-up: closest to 3 months; assessed with: Aberdeen; benefit indicated by lower values)** | | | | | | | | | | | | |
| 1^14^ | randomised trials | very serious^c^ | not serious^g^ | serious^p^ | very serious^i^ | none | 26 | 26 | - | SMD **0.5 lower** (1.05 lower to 0.05 higher) | ⨁◯◯◯ Very low | CRITICAL CRITICAL |
|  | | | | | | | | | | | | |
|  | | | | | | | | | | | | |
|  | | | | | | | | | | | | |
| **Function in people without leg pain (follow-up: closest to 3 months; assessed with: RMDQ, ODI, Hannover; benefit indicated by lower values)** | | | | | | | | | | | | |
| 5^1,4,16,20,23,af^ | randomised trials | very serious^c^ | not serious^w^ | not serious^e^ | not serious^f^ | none | 184 | 249 | - | SMD **0.65 lower** (0.95 lower to 0.34 lower) | ⨁⨁◯◯ Low | CRITICAL |
| **Function in people with unclassified presence of leg pain (follow-up: closest to 3 months; assessed with: BPI; benefit indicated by lower values)** | | | | | | | | | | | | |
| 1^22,ae^ | randomised trials | very serious^c^ | not serious^g^ | serious^p^ | very serious^i^ | none | 37 | 37 | - | SMD **0.43 higher** (0.03 lower to 0.89 higher) | ⨁◯◯◯ Very low | CRITICAL |
| **Function in people in high to upper-middle income countries (follow-up: closest to 3 months; assessed with: RMDQ, ODI, JOA, BPI, Hannover, Aberdeen; benefit indicated by lower values)** | | | | | | | | | | | | |
| 7^1,4,13,14,20,22,23,ae,af^ | randomised trials | very serious^c^ | not serious^w^ | not serious^j^ | not serious^f^ | none | 267 | 332 | - | SMD **0.56 lower** (0.95 lower to 0.17 lower) | ⨁⨁◯◯ Low | CRITICAL |
| **Function in people in low to lower-middle income countries (follow-up: closest to 3 months; assessed with: RMDQ; benefit indicated by lower values)** | | | | | | | | | | | | |
| 1^16^ | randomised trials | very serious^c^ | not serious^g^ | serious^h^ | very serious^i^ | none | 20 | 20 | - | SMD **0.67 lower** (1.31 lower to 0.04 lower) | ⨁◯◯◯ Very low | CRITICAL |
| **Function stratified by race/ethnicity (follow-up: closest to 3 months)** | | | | | | | | | | | | |
| 0 |  |  |  |  |  |  |  |  |  |  |  | CRITICAL |
|  | | | | | | | | | | | | |
| **Function in people treated with needling therapies type TCM (follow-up: closest to 3 months; assessed with: RMDQ, ODI, JOA, BPI, Hannover, Aberdeen; benefit indicated by lower values)** | | | | | | | | | | | | |
| 7^1,4,13,14,16,20,22,ae,af^ | randomised trials | very serious^c^ | not serious^w^ | not serious^e^ | not serious^f^ | none | 213 | 212 | - | SMD **0.6 lower** (1.04 lower to 0.15 lower) | ⨁⨁◯◯ Low | CRITICAL |
| **Function in people treated with needling therapies type mixed (TCM, myofascial) (follow-up: closest to 3 months; assessed with: Hannover; benefit indicated by lower values)** | | | | | | | | | | | | |
| 1^23^ | randomised trials | very serious^c^ | not serious^g^ | serious^p^ | serious^l^ | none | 74 | 140 | - | SMD **0.48 lower** (0.77 lower to 0.2 lower) | ⨁◯◯◯ Very low | CRITICAL |
| **Function in people treated with needling therapies with manual stimulation (follow-up: closest to 3 months; assessed with: ODI, JOA, Hannover; benefit indicated by lower values)** | | | | | | | | | | | | |
| 3^13,20,23^ | randomised trials | very serious^c^ | not serious^w^ | not serious^j^ | not serious^f^ | none | 164 | 230 | - | SMD **0.58 lower** (0.97 lower to 0.2 lower) | ⨁⨁◯◯ Low | CRITICAL |
| **Function in people treated with needling therapies with electrical stimulation (follow-up: closest to 3 months; assessed with: RMDQ, Aberdeen; benefit indicated by lower values)** | | | | | | | | | | | | |
| 4^1,4,14,16^ | randomised trials | very serious^c^ | not serious^w^ | not serious^e^ | very serious^i^ | none | 86 | 85 | - | SMD **0.82 lower** (1.15 lower to 0.49 lower) | ⨁◯◯◯ Very low | CRITICAL |
|  | | | | | | | | | | | | |
|  | | | | | | | | | | | | |
|  | | | | | | | | | | | | |
|  | | | | | | | | | | | | |
| **Function in people treated with needling therapies without stimulation (follow-up: closest to 3 months; assessed with: BPI; benefit indicated by lower values)** | | | | | | | | | | | | |
| 1^22,ae,ag^ | randomised trials | very serious^c^ | not serious^g^ | serious^p^ | very serious^i^ | none | 37 | 37 | - | SMD **0.43 higher** (0.03 lower to 0.89 higher) | ⨁◯◯◯ Very low | CRITICAL |
| **Function after removing high risk of bias studies (follow-up: closest to 3 months; assessed with: ODI; benefit indicated by lower values)** | | | | | | | | | | | | |
| 1^20^ | randomised trials | very serious^c^ | not serious^g^ | serious^p^ | very serious^i^ | none | 50 | 50 | - | SMD **0.3 lower** (0.69 lower to 0.1 higher) | ⨁◯◯◯ Very low | CRITICAL |
| **Function (follow-up: closest to 6 months; assessed with: Hannover; benefit indicated by lower values; Scale from: 0 to 100)** | | | | | | | | | | | | |
| 1^23,ah^ | randomised trials | very serious^c^ | not serious^g^ | serious^p^ | serious^l^ | none | 74 | 140 | - | MD **8.3 lower** (13.93 lower to 2.67 lower) | ⨁◯◯◯ Very low | CRITICAL |
| **Function stratified by gender (follow-up: closest to 6 months)** | | | | | | | | | | | | |
| 0 |  |  |  |  |  |  |  |  |  |  |  | CRITICAL |
| **Function in people without leg pain (follow-up: closest to 6 months; assessed with: Hannover; benefit indicated by lower values; Scale from: 0 to 100)** | | | | | | | | | | | | |
| 1^23^ | randomised trials | very serious^c^ | not serious^g^ | not serious | serious^l^ | none | 74 | 140 | - | MD **8.3 lower** (13.93 lower to 2.67 lower) | ⨁◯◯◯ Very low | CRITICAL CRITICAL |
|  | | | | | | | | | | | | |
| **Function in people in high to upper-middle income countries (follow-up: closest to 6 months; assessed with: Hannover; benefit indicated by lower values; Scale from: 0 to 100)** | | | | | | | | | | | | |
| 1^23^ | randomised trials | very serious^c^ | not serious^g^ | not serious | serious^l^ | none | 74 | 140 | - | MD **8.3 lower** (13.93 lower to 2.67 lower) | ⨁◯◯◯ Very low | CRITICAL |
| **Function in people in low to lower-middle income countries (follow-up: closest to 6 months)** | | | | | | | | | | | | |
| 0 |  |  |  |  |  |  |  |  |  |  |  | CRITICAL |
| **Function stratified by race/ethnicity (follow-up: closest to 6 months)** | | | | | | | | | | | | |
| 0 |  |  |  |  |  |  |  |  |  |  |  | CRITICAL |
| **Function after removing high risk of bias studies (follow-up: closest to 6 months)** | | | | | | | | | | | | |
| 0 |  |  |  |  |  |  |  |  |  |  |  | CRITICAL |
| **Health-related quality of life (follow-up: closest to 2 weeks; assessed with: EQ-5D; benefit indicated by higher values; Scale from: 0 to 1)** | | | | | | | | | | | | |
| 1^10^ | randomised trials | very serious^c^ | not serious^g^ | serious^p^ | very serious^i^ | none | 19 | 19 | - | MD **0.02 higher** (0.09 lower to 0.14 higher) | ⨁◯◯◯ Very low | CRITICAL |
| **Health-related quality of life in people without leg pain (follow-up: closest to 2 weeks; assessed with: EQ-5D; benefit indicated by higher values; Scale from: 0 to 1)** | | | | | | | | | | | | |
| 1^10^ | randomised trials | very serious^c^ | not serious^g^ | serious^p^ | very serious^i^ | none | 19 | 19 | - | MD **0.02 higher** (0.09 lower to 0.14 higher) | ⨁◯◯◯ Very low | CRITICAL CRITICAL |
|  | | | | | | | | | | | | |
|  | | | | | | | | | | | | |
| **Health-related quality of life in people in high to upper-middle income countries (follow-up: closest to 2 weeks; assessed with: EQ-5D; benefit indicated by higher values; Scale from: 0 to 1)** | | | | | | | | | | | | |
| 1^10^ | randomised trials | very serious^c^ | not serious^g^ | serious^p^ | very serious^i^ | none | 19 | 19 | - | MD **0.02 higher** (0.09 lower to 0.14 higher) | ⨁◯◯◯ Very low | CRITICAL |
| **Health-related quality of life in people in low to lower-middle income countries (follow-up: closest to 2 weeks)** | | | | | | | | | | | | |
| 0 |  |  |  |  |  |  |  |  |  |  |  | CRITICAL |
| **Health-related quality of life stratified by gender (follow-up: closest to 2 weeks)** | | | | | | | | | | | | |
| 0 |  |  |  |  |  |  |  |  |  |  |  | CRITICAL |
| **Health-related quality of life stratified by race/ethnicity (follow-up: closest to 2 weeks)** | | | | | | | | | | | | |
| 0 |  |  |  |  |  |  |  |  |  |  |  | CRITICAL |
| **Health-related quality of life in people treated with needling therapies type TCM (follow-up: closest to 2 weeks; assessed with: EQ-5D; benefit indicated by higher values; Scale from: 0 to 1)** | | | | | | | | | | | | |
| 1^10,ai^ | randomised trials | very serious^c^ | not serious^g^ | serious^p^ | very serious^i^ | none | 19 | 19 | - | MD **0.02 higher** (0.09 lower to 0.14 higher) | ⨁◯◯◯ Very low | CRITICAL |
| **Health-related quality of life (follow-up: closest to 3 months; assessed with: SF-36 (PCS); benefit indicated by higher values; Scale from: 0 to 100)** | | | | | | | | | | | | |
| 1^23,ah,aj^ | randomised trials | very serious^c^ | not serious^g^ | serious^p^ | serious^l^ | none | 140 | 74 | - | MD **6.6 higher** (3.9 higher to 9.3 higher) | ⨁◯◯◯ Very low | CRITICAL |
|  | | | | | | | | | | | | |
|  | | | | | | | | | | | | |
| **Health-related quality of life (follow-up: closest to 3 months; assessed with: SF-36 (MCS); benefit indicated by higher values; Scale from: 0 to 100)** | | | | | | | | | | | | |
| 1^23,ah,ak^ | randomised trials | very serious^c^ | not serious^g^ | serious^p^ | serious^l^ | none | 140 | 74 | - | MD **1.2 higher** (1.86 lower to 4.26 higher) | ⨁◯◯◯ Very low | CRITICAL |
| **Health-related quality of life stratified by gender (follow-up: closest to 3 months)** | | | | | | | | | | | | |
| 0 |  |  |  |  |  |  |  |  |  |  |  | CRITICAL |
| **Health-related quality of life stratified by race/ethnicity (follow-up: closest to 3 months)** | | | | | | | | | | | | |
| 0 |  |  |  |  |  |  |  |  |  |  |  | CRITICAL |
| **Health-related quality of life in people in low to lower-middle income countries (follow-up: closest to 3 months)** | | | | | | | | | | | | |
| 0 |  |  |  |  |  |  |  |  |  |  |  | CRITICAL |
| **Health-related quality of life after removing high risk of bias studies (follow-up: closest to 3 months)** | | | | | | | | | | | | |
| 0 |  |  |  |  |  |  |  |  |  |  |  | CRITICAL |
| **Depression (follow-up: closest to 3 months; assessed with: General Depression Scale; benefit indicated by lower values; Scale from: 0 to 61)** | | | | | | | | | | | | |
| 1^23,ah^ | randomised trials | very serious^c^ | not serious^g^ | serious^p^ | serious^l^ | none | 140 | 74 | - | MD **0.8 lower** (3.6 lower to 2 higher) | ⨁◯◯◯ Very low | CRITICAL |
| **Depression stratified by gender** | | | | | | | | | | | | |
| 0 |  |  |  |  |  |  |  |  |  |  |  | CRITICAL |
| **Depression stratified by race/ethnicity** | | | | | | | | | | | | |
| 0 |  |  |  |  |  |  |  |  |  |  |  | CRITICAL |
|  | | | | | | | | | | | | |
|  | | | | | | | | | | | | |
| **Depression in people in low to lower-middle income countries** | | | | | | | | | | | | |
| 0 |  |  |  |  |  |  |  |  |  |  |  | CRITICAL |
| **Depression after removing high risk of bias studies** | | | | | | | | | | | | |
| 0 |  |  |  |  |  |  |  |  |  |  |  | CRITICAL |
| **Depression in people with leg pain** | | | | | | | | | | | | |
| 0 |  |  |  |  |  |  |  |  |  |  |  | CRITICAL |
| **Other psychological functioning (fear avoidance, catastrophizing, anxiety, self-efficacy)** | | | | | | | | | | | | |
| 0 |  |  |  |  |  |  |  |  |  |  |  | CRITICAL |
| **Social participation** | | | | | | | | | | | | |
| 0 |  |  |  |  |  |  |  |  |  |  |  | CRITICAL |
| **Adverse events/harms during intervention period (needling therapies type TCM)** | | | | | | | | | | | | |
| 3^20,24,25,al,am^ | randomised trials | very serious^c^ | serious^an^ | not serious^j^ | very serious^ao^ | none | 11/113 (9.7%) | 2/110 (1.8%) | **OR 3.12** (0.42 to 23.44) | **36 more per 1,000** (from 10 fewer to 285 more) | ⨁◯◯◯ Very low | CRITICAL |
| **Adverse events/harms in people without leg pain during intervention period** | | | | | | | | | | | | |
| 2^20,24,al,ap^ | randomised trials | very serious^c^ | not serious^aq^ | not serious^j^ | very serious^ao^ | none | 9/90 (10.0%) | 0/90 (0.0%) | **OR 8.77** (1.02 to 75.35) | **0 fewer per 1,000** (from 0 fewer to 0 fewer) | ⨁◯◯◯ Very low | CRITICAL |
| **Adverse events/harms in people with unclassified presence of leg pain during intervention period** | | | | | | | | | | | | |
| 1^25,ar^ | randomised trials | very serious^c^ | not serious^g^ | serious^p^ | very serious^ao^ | none | 2/23 (8.7%) | 2/20 (10.0%) | **OR 0.86** (0.11 to 6.72) | **13 fewer per 1,000** (from 88 fewer to 327 more) | ⨁◯◯◯ Very low | CRITICAL |
| **Adverse events/harms stratified by gender during intervention period** | | | | | | | | | | | | |
| 0 |  |  |  |  |  |  |  |  |  |  |  | CRITICAL |
| **Adverse events/harms stratified by race/ethnicity during intervention period** | | | | | | | | | | | | |
| 0 |  |  |  |  |  |  |  |  |  |  |  | CRITICAL |
| **Adverse events/harms in people in low to lower-middle income countries during intervention period** | | | | | | | | | | | | |
| 0 |  |  |  |  |  |  |  |  |  |  |  | CRITICAL |
| **Adverse events/harms in people treated with needling therapies with manual stimulation during intervention period** | | | | | | | | | | | | |
| 2^20,25,al,as^ | randomised trials | very serious^c^ | serious^at^ | not serious^j^ | very serious^ao^ | none | 10/73 (13.7%) | 2/70 (2.9%) | **OR 3.59** (0.14 to 94.80) | **67 more per 1,000** (from 24 fewer to 707 more) | ⨁◯◯◯ Very low | CRITICAL |
| **Adverse events/harms in people treated with needling therapies (stimulation not reported) during intervention period** | | | | | | | | | | | | |
| 1^24,au^ | randomised trials | very serious^c^ | not serious^g^ | serious^p^ | very serious^ao^ | none | 1/40 (2.5%) | 0/40 (0.0%) | **OR 3.08** (0.12 to 77.80) | **0 fewer per 1,000** (from 0 fewer to 0 fewer) | ⨁◯◯◯ Very low | CRITICAL |
| **Adverse events/harms after removing high risk of bias studies during intervention period** | | | | | | | | | | | | |
| 1^20,av^ | randomised trials | very serious^c^ | not serious^g^ | serious^p^ | very serious^ao^ | none | 8/50 (16.0%) | 0/50 (0.0%) | **OR 20.20** (1.13 to 360.28) | **0 fewer per 1,000** (from 0 fewer to 0 fewer) | ⨁◯◯◯ Very low | CRITICAL |
|  | | | | | | | | | | | | |
|  | | | | | | | | | | | | |
|  | | | | | | | | | | | | |
| **OLDER ADULTS (aged 60 years or more)** | | | | | | | | | | | | |
| **Pain (follow-up: closest to 2 weeks; assessed with: Pain Scale; benefit indicated by lower values; Scale from: 0 to 10)** | | | | | | | | | | | | |
| 1^4,aw,ax^ | randomised trials | very serious^c^ | not serious^g^ | serious^p^ | very serious^i^ | none | 24 | 23 | - | MD **0.9 lower** (1.53 lower to 0.27 lower) | ⨁◯◯◯ Very low | CRITICAL |
| **Pain (follow-up: closest to 3 months; assessed with: Pain Scale; benefit indicated by lower values; Scale from: 0 to 10)** | | | | | | | | | | | | |
| 1^4,aw,ax^ | randomised trials | very serious^c^ | not serious^g^ | serious^p^ | very serious^i^ | none | 24 | 23 | - | MD **1.1 lower** (1.62 lower to 0.58 lower) | ⨁◯◯◯ Very low | CRITICAL |
| **Pain stratified by gender** | | | | | | | | | | | | |
| 0 |  |  |  |  |  |  |  |  |  |  |  | CRITICAL |
| **Pain stratified by race/ethnicity** | | | | | | | | | | | | |
| 0 |  |  |  |  |  |  |  |  |  |  |  | CRITICAL |
| **Pain in people in low to lower-middle income countries** | | | | | | | | | | | | |
| 0 |  |  |  |  |  |  |  |  |  |  |  | CRITICAL |
| **Function (follow-up: closest to 2 weeks; assessed with: RMDQ; benefit indicated by lower values)** | | | | | | | | | | | | |
| 1^4,ax^ | randomised trials | very serious^c^ | not serious^g^ | serious^p^ | very serious^i^ | none | 24 | 23 | - | SMD **1.1 lower** (1.71 lower to 0.48 lower) | ⨁◯◯◯ Very low | CRITICAL |
|  | | | | | | | | | | | | |
| **Function (follow-up: closest to 3 months; assessed with: RMDQ; benefit indicated by lower values)** | | | | | | | | | | | | |
| 1^4,ax^ | randomised trials | very serious^c^ | not serious^g^ | serious^p^ | very serious^i^ | none | 24 | 23 | - | SMD **1.04 lower** (1.66 lower to 0.43 lower) | ⨁◯◯◯ Very low | CRITICAL |
| **Function stratified by gender** | | | | | | | | | | | | |
| 0 |  |  |  |  |  |  |  |  |  |  |  | CRITICAL |
| **Function stratified by race/ethnicity** | | | | | | | | | | | | |
| 0 |  |  |  |  |  |  |  |  |  |  |  | CRITICAL |
| **Function in people in low to lower-middle income countries** | | | | | | | | | | | | |
| 0 |  |  |  |  |  |  |  |  |  |  |  | CRITICAL |
| **Health-related quality of life** | | | | | | | | | | | | |
| 0 |  |  |  |  |  |  |  |  |  |  |  | CRITICAL |
| **Adverse events/harms** | | | | | | | | | | | | |
| 0 |  |  |  |  |  |  |  |  |  |  |  | CRITICAL |
| **Psychological functioning (depression, fear avoidance, catastrophizing, anxiety, self-efficacy)** | | | | | | | | | | | | |
| 0 |  |  |  |  |  |  |  |  |  |  |  | CRITICAL |
| **Change in use of medications** | | | | | | | | | | | | |
| 0 |  |  |  |  |  |  |  |  |  |  |  | CRITICAL |
| **Falls** | | | | | | | | | | | | |
| 0 |  |  |  |  |  |  |  |  |  |  |  | CRITICAL |

**BPI:** Brief Pain Inventory; **CI:** confidence interval; **EQ-5D:** EuroQol 5 Dimensions; **JOA:** Japanese Orthopedic Association; **MD:** mean difference; **MCS:** Mental Component Summary; **OIS:** Optimal Information Size; **OR:** odds ratio; **NRS:** numerical rating scale; **ODI:** Oswestry Disability Index; **PCS:** Physical Component Summary; **RMDQ:** Roland Morris Disability Questionnaire; **SF-36:** Short Form Health Survey – 36-item; **SMD:** standardised mean difference; **TCM:** Traditional Chinese Medicine; **VAS:** Visual Analogue Scale

The following was used to guide the ratings.

**Risk of bias:** *Not serious:* all or most of the weight (>50%) comes from overall low risk of bias trial(s). *Serious:* some of the weight (<50%) comes from overall low risk of bias trial(s). *Very serious:* all or most of the weight (>50%) comes from overall high or unclear risk of bias trial(s).

**Inconsistency:** *Not serious:* high extent of similarity of point estimates and overlap of confidence intervals; statistical heterogeneity (I^2^) is between 0% and 40%, which might not be important. *Serious:* some extent of similarity of point estimates and overlap of confidence intervals; statistical heterogeneity (I^2^) is between 30% and 60%, which could not be explained due to small subgroups and may represent moderate heterogeneity. *Very serious:* little or no similarity of point estimates and overlap of confidence intervals; statistical heterogeneity (I^2^) is between 50% and 90% or 75% and 100%, which could not be explained due to small subgroups and may represent substantial or considerable heterogeneity, respectively.

**Indirectness:** *Not serious:* trial(s) were conducted in different countries or settings. *Serious:* trial(s) were conducted from a single country/setting. *Very serious:* evidence is not directly related to PICO question.

**Imprecision:** *Not serious:* Optimal Information Size (OIS) was reached (i.e., sample sizes with at least 200 participants per group may provide prognostic balance); and the entire confidence interval lies on one side of the threshold that may be considered clinically important (≥10% scale range or SMD ≥0.2 for continuous variables, ≥10% for binary variables), such that the clinical course of action would not differ if the upper versus the lower boundary of the confidence interval represented the truth. *Serious:* OIS would not have been reached (sample sizes with less than 200 participants per group); if the OIS was reached, the clinical course of action might differ if the upper versus the lower boundary of the confidence interval represented the truth. *Very serious:* similar to ‘serious’ but to a greater extent (e.g., very small sample sizes and confidence intervals crossing appreciable benefit and harm).

**Other considerations:** *Not serious:* Publication bias is undetected. *Serious/very serious:* Publication bias is strongly suspected.

#### Explanations

a. Zaringhalam 2010 assessed two comparisons (there were 2 comparison groups). Both comparisons included in meta-analysis.

b. Two trials were not included in the meta-analysis because they reported within-group change scores. De Castro Moura 2019 (ID#: 32): 111 participants total, rated as overall high risk of bias. Clinically important (MD≥1, scale 0 to 10) and statistically significant within group mean difference for Chinese auricular acupuncture group: 1.38 (95% CI 0.43; 2.33); no significant within group changes for French auricular acupuncture or comparison group; no statistical comparison between groups. Weiß 2013 (ID#: 1153): 160 participants total, rated as overall high risk of bias. No significant difference between groups in the proportion of participants experiencing improvement in pain while sitting/standing or walking.

c. Risk of bias: We downgraded twice because all of the weight comes from high or unclear (i.e., some concerns) overall risk of bias trials.

d. Inconsistency: We did not downgrade. All or most trials are in the same direction, showing a reduction in pain.

e. Indirectness: We did not downgrade because the trials were conducted in different countries (high to low-income).

f. Imprecision: We did not downgrade. The point estimate reached the pre-specified threshold for what may be considered clinically important (MD ≥ 1 or SMD ≥ 0.2 ). The confidence interval does not cross the null.

g. Inconsistency: We did not downgrade; however, there are no other trials with which to compare findings.

h. Indirectness: We downgraded once; trial(s) conducted in one country (low or lower-middle income).

i. Imprecision: We downgraded twice. The sample size is small (OIS would not have been achieved).

j. Indirectness: We did not downgrade because the trials were conducted in different countries (high or upper-middle income).

k. Imprecision: We did not downgrade. The point estimate did not reach the pre-specified threshold for what may be considered clinically important (MD ≥ 1). The confidence interval does not cross the null.

l. Imprecision: We downgraded once. The sample size is small (OIS would not have been achieved).

m. Inconsistency: We downgraded once. Most trials are in the same direction with similar point estimates. Statistical heterogeneity is between 75% and 100% (i.e., I2 = 97%). This could not be explained due to small subgroups and may represent considerable heterogeneity.

n. Inconsistency: We downgraded once. Most of the trials are in the same direction showing a reduction in pain. Statistical heterogeneity is between 75% and 100% (i.e., I2 = 92%). This could not be explained due to small subgroups and may represent considerable heterogeneity.

o. Indirectness: We did not downgrade because the trials were conducted in different countries (low or lower-middle income).

p. Indirectness: We downgraded once; trial(s) conducted in one country (high or upper-middle income).

q. One trial was not included in the meta-analysis because it reported within-group change scores. De Castro Moura 2019 (ID#: 32): 111 participants total; rated as overall high risk of bias. Clinically important (MD≥1, scale 0 to 10) and statistically significant within group mean difference for Chinese auricular acupuncture group: 1.38 (95% CI 0.43; 2.33); no significant within group changes for French auricular acupuncture or comparison group; no statistical comparison between groups.

r. One trial was not included in the meta-analysis because it reported within-group change scores. Weiß 2013 (ID#: 1153): 160 participants total, rated as overall high risk of bias. No significant difference between groups in the proportion of participants experiencing improvement in pain while sitting/standing or walking.

s. Two trials were not included in the meta-analysis because they reported within-group change scores. De Castro Moura 2019 (ID#: 32): 111 participants total, rated as overall high risk of bias. No significant within group changes needling therapies groups or comparison group; no statistical comparison between groups. Weiß 2013 (ID#: 1153): 160 participants total, rated as overall high risk of bias. Statistically significant difference between proportion of participants experiencing improvement in pain while sitting/standing (p<0.01) but not in pain while walking.

t. Imprecision: We downgraded once. The point estimate reached the pre-specified threshold for what may be considered clinically important (MD ≥ 1). The upper boundary of the 95% CI crosses the threshold for what may be considered appreciable benefit (-1).

u. Use of stimulation was not reported in Weiß 2013 (ID#: 1153).

v. One trial was not included in the meta-analysis because it reported within-group change scores. De Castro Moura 2019 (ID#: 32): 111 participants total; rated as overall high risk of bias. Clinically unimportant (MD<2.4, scale 0 to 24) but statistically significant within group mean difference for Chinese auricular acupuncture group: 1.56 (95% CI 0.10; 3.02); no significant within group changes for French auricular acupuncture or comparison group; no statistical comparison between groups.

w. Inconsistency: We did not downgrade. All or most trials are in the same direction, showing a reduction in functional limitation.

x. Inconsistency: We downgraded once. The results are in the same direction. One point estimate is much larger in magnitude; confidence intervals of the other studies do not overlap with it. Statistical heterogeneity is between 75% and 100% (i.e., I2 = 99%). This could not be explained due to small subgroups and may represent considerable heterogeneity.

y. Imprecision: We downgraded twice. The point estimate reached the pre-specified threshold for what may be considered clinically important (SMD ≥ 0.2). The lower boundary of the 95% CI crosses the threshold for what may be considered appreciable benefit (-0.2), and the upper boundary crosses the threshold for what may be considered appreciable harm (+0.2).

z. Inconsistency: We downgraded once. The point estimates differ with little overlap in confidence intervals. Statistical heterogeneity is between 75% and 100% (i.e., I2 = 98%). This could not be explained due to small subgroups and may represent considerable heterogeneity.

aa. Inconsistency: We downgraded once. Most of the point estimates are in the same direction. Statistical heterogeneity is between 75% and 100% (i.e., I2 = 94%). This could not be explained due to small subgroups and may represent considerable heterogeneity.

ab. Inconsistency: We downgraded once. Most of the trials are in the same direction showing a reduction in functional limitation. Statistical heterogeneity is between 75% and 100% (i.e., I2 = 92%). This could not be explained due to small subgroups and may represent considerable heterogeneity.

ac. Inconsistency: We downgraded once. The point estimates are in the same direction. Statistical heterogeneity is between 75% and 100% (i.e., I2 = 94%). This could not be explained due to small subgroups and may represent considerable heterogeneity.

ad. Inconsistency: We downgraded once. The point estimates are in the same direction with little overlap between confidence intervals. Statistical heterogeneity is between 75% and 100% (i.e., I2 = 76%). This could not be explained due to small subgroups and may represent considerable heterogeneity.

ae. One trial was not included in the meta-analysis because it reported within-group change scores. De Castro Moura 2019 (ID#: 32): 111 participants total; rated as overall high risk of bias. No significant within group changes for needling therapies groups or comparison group; no statistical comparison between groups.

af. One trial was not included in the meta-analysis because it reported within-group change scores. Witt 2006 (ID#: 2010): 3093 participants total; rated as overall high risk of bias. Statistically significant difference between groups for mean percent disability reduction (scale 0 to 100) (22.0; 95% CI 19.3, 24.7; p<0.001) favouring needling therapies.

ag. Use of stimulation was not reported in Witt 2006 (ID#: 2010).

ah. Brinkhaus 2006: participants had no leg pain; in high to upper-middle income country; were treated with mixed needling therapies type (TCM, dry needling) with manual stimulation.

ai. Sung 2020: needling therapies with threading stimulation; rated as overall unclear risk of bias.

aj. One trial was not included in the meta-analysis because it reported within-group change scores. Witt 2006 (ID#: 2010): 3093 participants total; rated as overall high risk of bias. clinically unimportant (PCS: MD <10, scale 0-100) but statistically significant difference between groups for mean point increase in quality of life (4.7; 95% CI 4.0, 5.4; p<0.001) favouring needling therapies.

ak. One trial was not included in the meta-analysis because it reported within-group change scores. Witt 2006 (ID#: 2010): 3093 participants total; rated as overall high risk of bias. Clinically unimportant (MCS: MD<10, scale 0-100) but statistically significant different between groups for mean point increase in quality of life (2.1; 95% CI 1.4, 2.8; p<0.001) favouring needling therapies.

al. One trial was not included in meta-analysis due to missing data. Molsberger 2002 (ID#: 2007): 186 participants total, rated as overall high risk of bias. Authors reported no important adverse events or side effects were observed in any group.

am. Minor adverse events: Kerr 2003: increased tenderness, leg pain for a few days following treatment. Ushinohama 2016: dizziness in one participant (unknown treatment group allocation). Yuan 2016: transient (up to 1 week) worsening back pain, needling therapies point pain and bruising, back and leg numbness and discomfort, shoulder pain, foot pain.

an. Inconsistency: We downgraded once. The point estimates vary and have overlapping confidence intervals. Statistical heterogeneity is between 30% and 60% (i.e., I2 = 41%). This could not be explained due to small subgroups and may represent moderate heterogeneity.

ao. Imprecision: We downgraded twice due to small sample size and number of events.

ap. Minor adverse events: Ushinohama 2016: dizziness in one participant (unknown treatment group allocation). Yuan 2016: transient (up to 1 week) worsening back pain, needling therapies point pain and bruising, back and leg numbness and discomfort, shoulder pain, foot pain.

aq. Inconsistency: We did not downgrade. The point estimates are in the same direction with overlapping confidence intervals. Statistical heterogeneity is between 0% and 40%, which might not be important (i.e., I2 = 0%).

ar. Minor adverse events: Kerr 2003: increased tenderness, leg pain for a few days following treatment.

as. Minor adverse events: Kerr 2003: increased tenderness, leg pain for a few days following treatment. Yuan 2016: transient (up to 1 week) worsening back pain, needling therapies point pain and bruising, back and leg numbness and discomfort, shoulder pain, foot pain.

at. Inconsistency: We downgraded once. The point estimates go in different directions; there is some overlap in confidence intervals. Statistical heterogeneity is between 50% and 90% (i.e., I2 = 71%). This could not be explained due to small subgroups and may represent substantial heterogeneity.

au. Minor adverse events: Ushinohama 2016: dizziness in one participant (unknown treatment group allocation).

av. Minor adverse events: Yuan 2016: transient (up to 1 week) worsening back pain, needling therapies point pain and bruising, back and leg numbness and discomfort, shoulder pain, foot pain.

aw. Meng 2003: Pain Scale range not specified (assumed 0-10).

ax. Meng 2003: Participants had no leg pain, were in a high to upper-middle income country, and were treated with needling therapies type TCM with electrical stimulation.

#### References

1.Depaoli Lemos VJ, Selau RC,Blos C,Baptista Dohnert M,Boff Daitx R,de Almeida Brito V. Electroacupuncture and Transcutaneous Electrical Nerve Stimulation in Chronic Nonspecific Low Back Pain: a Blind Randomized Clinical Trial. 2021.

2.Li YJ, Zhuang WS,Cai XG,Yang Y,Han MM,Zhang DW. [Effect of acupuncture at " three points of ilioumbar" on lumbar function and pain in patients with iliopsoas muscle strain]. 2019.

3.Li SK, Zhao J,Cao XW,Zhu S,Liu ZQ,Fan YX. [Core stability training combined with acupuncture in treatment of chronic nonspecific low back pain: a prospective randomized controlled trial]. 2022.

4.Meng CF, Wang D,Ngeow J,Lao L,Peterson M,Paget S. Acupuncture for chronic low back pain in older patients: a randomized, controlled trial. 2003.

5.Moslemi F, Farokhi ZS. Effects of electroacupuncture on pain, functional disability and ultrasonographic changes of gluteus maximus muscle in non-specific chronic low back pain patients with gluteus maximus muscle trigger points. 2020.

6.Pan J, Yang L,Qiu Y,et al. [Clinical trial on effect of acupuncture combined with sling exercise training on lumbar disc herniation] . 2019.

7.P, Ran. [Effect of acupuncture on pain and lumbar function in patients with discogenic low back pain]. 2021.

8.Ren B, Feng X,Zhang C. [Clinical trial on the analgesic effect of acupuncture based on meridian-tendon dialectical theory in chronic low back pain]. 2021.

9.Shi F, Dong B,Lin X,Fu Y. [Clinical observation on treatment of non-specific low back pain based on meridian theory and acupuncture]. 2021.

10.Sung WS, Hong Y,Jeon SR,et al. Efficacy and safety of thread embedding acupuncture combined with acupuncture for chronic low back pain: A randomized, controlled, assessor-blinded, multicenter clinical trial. 2020.

11.Tabatabaiee A, Takamjani IE,Sarrafzadeh J,Salehi R,Ahmadi M. Ultrasound-guided dry needling decreases pain in patients with piriformis syndrome. 2019.

12.Wang X, Yu X,Huang F,Luo X,Gong Y. [Effect of acupuncture combined with fire acupuncture on pain and serum inflammatory factor in patients with lumbar disc herniation]. 2020a.

13.Wang L, Wang Z,Wang Y,Guo Y. Observations on the efficacy of acupuncture for lumbar intervertebral disc herniation. 2020b.

14.Yeung CK, Leung MC,Chow DH. The use of electro-acupuncture in conjunction with exercise for the treatment of chronic low-back pain. 2003.

15.Yu XJ, Zhang L,Lu WY,Gao Q,Liu L,Wang Y. Effect of electroacupuncture combined with caudal epidural injection on functional rehabilitation of patients with lumbar hernia. 2021.

16.Zaringhalam J, Manaheji H,Rastqar A,Zaringhalam M. Reduction of chronic non-specific low back pain: a randomised controlled clinical trial on acupuncture and baclofen. 2010.

17.Zhai B, Wang C. [Therapeutic effect of drug cupping combined with deep puncture on Jiaji point on lumbar disc herniation and its effect on M-JOA score, ODI index and quality of life]. 2019.

18.Zhu X, Shen X,Kang L,et al.. Clinical trial on acupuncture combined with bone setting manipulation in the treatment of lumbar disc herniation. 2020.

19.Z, Zhu. Clinical trial on warming-needle moxibustion combined with joint loosening for chronic low back pain. 2021.

20.Yuan Q, Liu L,Ma J,Wu W,Ye M,Zhang Y. [A clinical trial of acupuncture therapy for treatment of chronic nonspecific low back pain] . 2016.

21.Molsberger AF, Mau J,Pawelec DB,Winkler J. Does acupuncture improve the orthopedic management of chronic low back pain--a randomized, blinded, controlled trial with 3 months follow up. 2002.

22.de Castro Moura C, de Cássia Lopes Chaves E,Couto Machado Chianca T,Ruginsk SG,Alves Nogueira D,Iunes DH. Effects of auricular acupuncture on chronic pain in people with back musculoskeletal disorders: a randomized clinical trial. 2019.

23.Brinkhaus B, Witt CM,Jena S,et al. Acupuncture in patients with chronic low back pain: a randomized controlled trial. 2006.

24.Ushinohama A, Cunha BP,Costa LO,Barela AM,Freitas PB. Effect of a single session of ear acupuncture on pain intensity and postural control in individuals with chronic low back pain: a randomized controlled trial. 2016.

25.Kerr DP, Walsh DM,Baxter D. Acupuncture in the management of chronic low back pain: a blinded randomized controlled trial. 2003.

GRADE evidence profile table 3: ***What are the benefits and harms of needling therapies in the management of community-dwelling adults (including older adults aged 60 years and over) with chronic primary low back pain (with or without leg pain) compared to usual care?***

| **Certainty assessment** | | | | | | | **№ of patients** | | **Effect** | | **Certainty** | **Importance** |
| --- | --- | --- | --- | --- | --- | --- | --- | --- | --- | --- | --- | --- |
| **№ of studies** | **Study design** | **Risk of bias** | **Inconsistency** | **Indirectness** | **Imprecision** | **Other considerations** | **Needling therapies** | **Usual care** | **Relative (95% CI)** | **Absolute (95% CI)** |
| **ALL ADULTS** | | | | | | | | | | | | |
| **Pain (in people with and without leg pain, in high-income country, treated with needling therapies type TCM) (follow-up: closest to 3 months; assessed with: NRS; benefit indicated by lower values; Scale from: 0 to 10)** | | | | | | | | | | | | |
| 1^1,a^ | randomised trials | very serious^b^ | not serious^c^ | not serious^d^ | serious^e^ | none | 299 | 148 | - | MD **1.35 lower** (1.86 lower to 0.84 lower) | ⨁◯◯◯ Very low | CRITICAL |
| **Pain (in people with and without leg pain, in high-income country, treated with needling therapies type TCM) (follow-up: closest to 6 months; assessed with: NRS; benefit indicated by lower values; Scale from: 0 to 10)** | | | | | | | | | | | | |
| 1^1,a^ | randomised trials | very serious^b^ | not serious^c^ | not serious^d^ | serious^f^ | none | 285 | 145 | - | MD **0.65 lower** (1.17 lower to 0.13 lower) | ⨁◯◯◯ Very low | CRITICAL |
| **Pain (in people with and without leg pain, in high-income country, treated with needling therapies type TCM) (follow-up: closest to 12 months; assessed with: NRS; benefit indicated by lower values; Scale from: 0 to 10)** | | | | | | | | | | | | |
| 1^1,a^ | randomised trials | very serious^b^ | not serious^c^ | not serious^d^ | serious^g^ | none | 288 | 143 | - | MD **0.5 lower** (1.02 lower to 0.02 higher) | ⨁◯◯◯ Very low | CRITICAL |
| **Pain stratified by gender** | | | | | | | | | | | | |
| 0 |  |  |  |  |  |  |  |  |  |  |  | CRITICAL |
| **Pain stratified by race/ethnicity** | | | | | | | | | | | | |
| 0 |  |  |  |  |  |  |  |  |  |  |  | CRITICAL |
| **Pain in people in low to lower-middle income countries** | | | | | | | | | | | | |
| 0 |  |  |  |  |  |  |  |  |  |  |  | CRITICAL |
| **Function (in people with and without leg pain, in high-income country, treated with needling therapies type TCM) (follow-up: closest to 3 months; assessed with: RMDQ; benefit indicated by lower values; Scale from: 0 to 24)** | | | | | | | | | | | | |
| 1^1,a^ | randomised trials | very serious^b^ | not serious^c^ | not serious^d^ | serious^h^ | none | 299 | 148 | - | MD **2.55 lower** (3.7 lower to 1.4 lower) | ⨁◯◯◯ Very low | CRITICAL |
| **Function (in people with and without leg pain, in high-income country, treated with needling therapies type TCM) (follow-up: closest to 6 months; assessed with: RMDQ; benefit indicated by lower values; Scale from: 0 to 24)** | | | | | | | | | | | | |
| 1^1,a^ | randomised trials | very serious^b^ | not serious^c^ | not serious^d^ | serious^i^ | none | 285 | 145 | - | MD **1.65 lower** (2.83 lower to 0.47 lower) | ⨁◯◯◯ Very low | CRITICAL |
| **Function (in people with and without leg pain, in high-income country, treated with needling therapies type TCM) (follow-up: closest to 12 months; assessed with: RMDQ; benefit indicated by lower values; Scale from: 0 to 24)** | | | | | | | | | | | | |
| 1^1,a^ | randomised trials | very serious^b^ | not serious^c^ | not serious^d^ | serious^i^ | none | 288 | 143 | - | MD **1.9 lower** (3.15 lower to 0.65 lower) | ⨁◯◯◯ Very low | CRITICAL |
| **Function stratified by gender** | | | | | | | | | | | | |
| 0 |  |  |  |  |  |  |  |  |  |  |  | CRITICAL |
| **Function stratified by race/ethnicity** | | | | | | | | | | | | |
| 0 |  |  |  |  |  |  |  |  |  |  |  | CRITICAL |
| **Function in people in low to lower-middle income countries** | | | | | | | | | | | | |
| 0 |  |  |  |  |  |  |  |  |  |  |  | CRITICAL |
| **Health-related quality of life** | | | | | | | | | | | | |
| 0 |  |  |  |  |  |  |  |  |  |  |  | CRITICAL |
| **Adverse events/harms** | | | | | | | | | | | | |
| 0 |  |  |  |  |  |  |  |  |  |  |  | CRITICAL |
| **Psychological functioning (depression, fear avoidance, catastrophizing, anxiety, self-efficacy)** | | | | | | | | | | | | |
| 0 |  |  |  |  |  |  |  |  |  |  |  | CRITICAL |
| **Social participation** | | | | | | | | | | | | |
| 0 |  |  |  |  |  |  |  |  |  |  |  | CRITICAL |
| **OLDER ADULTS (aged 60 years or more)** | | | | | | | | | | | | |
| **Pain** | | | | | | | | | | | | |
| 0 |  |  |  |  |  |  |  |  |  |  |  | CRITICAL |
| **Function** | | | | | | | | | | | | |
| 0 |  |  |  |  |  |  |  |  |  |  |  | CRITICAL |
| **Health-related quality of life** | | | | | | | | | | | | |
| 0 |  |  |  |  |  |  |  |  |  |  |  | CRITICAL |
| **Adverse events/harms** | | | | | | | | | | | | |
| 0 |  |  |  |  |  |  |  |  |  |  |  | CRITICAL |
| **Psychological functioning (depression, fear avoidance, catastrophizing, anxiety, self-efficacy)** | | | | | | | | | | | | |
| 0 |  |  |  |  |  |  |  |  |  |  |  | CRITICAL |
| **Change in use of medications** | | | | | | | | | | | | |
| 0 |  |  |  |  |  |  |  |  |  |  |  | CRITICAL |
| **Falls** | | | | | | | | | | | | |
| 0 |  |  |  |  |  |  |  |  |  |  |  | CRITICAL |

**CI:** confidence interval; **MD:** mean difference; **NRS:** numerical rating scale; **RMDQ:** Roland Morris Disability Questionnaire; **TCM:** Traditional Chinese Medicine

The following was used to guide the ratings.

**Risk of bias:** *Not serious:* all or most of the weight (>50%) comes from overall low risk of bias trial(s). *Serious:* some of the weight (<50%) comes from overall low risk of bias trial(s). *Very serious:* all or most of the weight (>50%) comes from overall high or unclear risk of bias trial(s).

**Inconsistency:** *Not serious:* high extent of similarity of point estimates and overlap of confidence intervals; statistical heterogeneity (I^2^) is between 0% and 40%, which might not be important. *Serious:* some extent of similarity of point estimates and overlap of confidence intervals; statistical heterogeneity (I^2^) is between 30% and 60%, which could not be explained due to small subgroups and may represent moderate heterogeneity. *Very serious:* little or no similarity of point estimates and overlap of confidence intervals; statistical heterogeneity (I^2^) is between 50% and 90% or 75% and 100%, which could not be explained due to small subgroups and may represent substantial or considerable heterogeneity, respectively.

**Indirectness:** *Not serious:* trial(s) were conducted in different countries or settings. *Serious:* trial(s) were conducted from a single country/setting. *Very serious:* evidence is not directly related to PICO question.

**Imprecision:** *Not serious:* Optimal Information Size (OIS) was reached (i.e., sample sizes with at least 200 participants per group may provide prognostic balance); and the entire confidence interval lies on one side of the threshold that may be considered clinically important (≥10% scale range or SMD ≥0.2 for continuous variables, ≥10% for binary variables), such that the clinical course of action would not differ if the upper versus the lower boundary of the confidence interval represented the truth. *Serious:* OIS would not have been reached (sample sizes with less than 200 participants per group); if the OIS was reached, the clinical course of action might differ if the upper versus the lower boundary of the confidence interval represented the truth. *Very serious:* similar to ‘serious’ but to a greater extent (e.g., very small sample sizes and confidence intervals crossing appreciable benefit and harm).

**Other considerations:** *Not serious:* Publication bias is undetected. *Serious/very serious:* Publication bias is strongly suspected.

#### Explanations

a. Cherkin 2009 had 2 comparisons (both included in meta-analysis); needling therapies stimulation not reported; rated as overall unclear risk of bias.

b. Risk of bias: We downgraded twice because all of the weight comes from high or unclear (i.e., some concerns) risk of bias studies.

c. Inconsistency: We did not downgrade; however, there are no other studies with which to compare findings.

d. Indirectness: We downgraded once because the trial was conducted in one country (high-income).

e. Imprecision: We downgraded once. The point estimate reached the pre-specified threshold for what may be considered clinically important (MD ≥ 1). The upper boundary of the 95% CI crosses the threshold for what may be considered appreciable benefit (-1).

f. Imprecision: We downgraded once. The point estimate did not reach the pre-specified threshold for what may be considered clinically important (MD ≥ 1). The lower boundary of the 95% CI crosses the threshold for what may be considered appreciable benefit (-1).

g. Imprecision: We downgraded once. The point estimate did not reach the pre-specified threshold for what may be considered clinically important (MD ≥ 1). The lower boundary of the 95% CI crosses the threshold for what may be considered appreciable benefit (-1), but the upper boundary does not cross the threshold for what may be considered appreciable harm (+1).

h. Imprecision: We downgraded once. The point estimate reached the pre-specified threshold for what may be considered clinically important (MD ≥ 2.4). The upper boundary of the 95% CI crosses the threshold for what may be considered appreciable benefit (-2.4).

i. Imprecision: We downgraded once. The point estimate did not reach the pre-specified threshold for what may be considered clinically important (MD ≥ 2.4). The lower boundary of the 95% CI crosses the threshold for what may be considered appreciable benefit (-2.4).

#### References

1.Cherkin DC, Sherman KJ,Avins AL,et al. A randomized trial comparing acupuncture, simulated acupuncture, and usual care for chronic low back pain. 2009.

**Online Resource 6:** GRADE Summary of findings tables

Summary of findings table 1: ***What are the benefits and harms of needling therapies in the management of community-dwelling adults (including older adults aged 60 years and over) with chronic primary low back pain (with or without leg pain) compared to sham?***

| Outcomes | **Anticipated absolute effects^*^** (95% CI) | | Relative effect (95% CI) | № of participants (studies) | Certainty of the evidence (GRADE) | Comments |
| --- | --- | --- | --- | --- | --- | --- |
|  | **Risk with sham** | **Risk with needling therapies** |  |  |  |  |
| **ALL ADULTS** | | | | | | |
| **Pain** 0 to 10; 0 = no pain follow-up: closest to 2 weeks | The mean pain ranged from **1.1 to 7.29** | MD **0.41 lower** (0.72 lower to 0.1 lower) | - | 1163 (7 RCTs)^1,2,3,4,5,6,7,a,b^ | ⨁⨁◯◯ Low^c,d,e,f^ | Needling therapies reduce pain more than sham in the immediate term (may not meet threshold for what may be considered a clinically important amount: MD ≥1). |
| **Pain** 0 to 10; 0 = no pain follow-up: closest to 3 months | The mean pain ranged from **2.3 to 6.93** | MD **0.42 lower** (0.88 lower to 0.05 higher) | - | 1891 (9 RCTs)^1,3,4,7,9,10,11,12,13,aa,ab,ac^ | ⨁◯◯◯ Very low^ad,ae,c,e^ | Needling therapies do not reduce pain more than sham in the short term. |
| **Pain** 0 to 10; 0 = no pain follow-up: closest to 6 months | The mean pain ranged from **3.5 to 6.19** | MD **0.21 lower** (0.58 lower to 0.16 higher) | - | 1517 (4 RCTs)^7,9,10,11,aa,ao^ | ⨁⨁◯◯ Low^ae,ap,c,e^ | Needling therapies do not reduce pain more than sham in the intermediate term. |
| **Pain** 0 to 10; 0 = no pain follow-up: closest to 12 months | The mean pain ranged from **3.4 to 4.49** | MD **0.02 lower** (0.51 lower to 0.47 higher) | - | 650 (2 RCTs)^9,10,aa^ | ⨁⨁◯◯ Low^ae,ar,e,t^ | Needling therapies do not reduce pain more than sham in the long term. |
| **Function** follow-up: closest to 2 weeks | The function ranged from **3.87** (RMDQ, 0-24) **to 64** (Hannover, 0-100) | SMD **0.22 SD lower** (0.54 lower to 0.11 higher) | - | 951 (4 RCTs)^1,4,5,7,as^ | ⨁◯◯◯ Very low^at,au,c,e^ | Needling therapies do not improve function more than sham in the immediate term. |
| **Function** follow-up: closest to 3 months | The function ranged from **2.3** (RMDQ, 0-24) **to 66.8** (Hannover) | SMD **0.03 SD lower** (0.17 lower to 0.11 higher) | - | 1752 (7 RCTs)^1,4,7,9,10,11,12,aa,ax^ | ⨁⨁◯◯ Low^bc,bd,c,e^ | Needling therapies do not improve function more than sham in the short term. |
| **Function** follow-up: closest to 6 months | The function ranged from **6.4** (RMDQ, 0-24) **to 66.8** (Hannover) | SMD **0.1 SD lower** (0.22 lower to 0.02 higher) | - | 1517 (4 RCTs)^7,9,10,11,aa,ax^ | ⨁◯◯◯ Very low^bf,c,e,s^ | Needling therapies do not improve function more than sham in the intermediate term. |
| **Health-related quality of life** (QofL) (total score) 0 to 100; 0 = poor QofL follow-up: closest to 2 weeks | The mean health-related quality of life was **57.5** | MD **6.4 higher** (6.42 lower to 19.22 higher) | - | 46 (1 RCT)^6,ax^ | ⨁◯◯◯ Very low^p,q,r,t^ | Needling therapies do not improve health-related quality of life more than sham in the immediate term. |
| **Health-related quality of life** (QofL) (total score) 0 to 100; 0 = poor QofL follow-up: closest to 3 months | The mean health-related quality of life was **122.56** | MD **7.78 higher** (1.41 higher to 14.15 higher) | - | 116 (1 RCT)^11,bo^ | ⨁◯◯◯ Very low^p,q,r,t^ | Needling therapies improve health-related quality of life more than sham in the short term (may not reach clinically important threshold (MD ≥10). |
| **Health -related quality of life** (QofL) (physical component) 0 to 100; 0 = poor QofL follow-up: closest to 3 months | The mean health-related quality of life physical component ranged from **36.2** (SF-36, 0-100) **to 39.2** (SF-12, 0-100) | SMD **0.25 higher** (0.07 lower to 0.56 higher) | - | 952 (2 RCTs)^7,9^ | ⨁◯◯◯ Very low^bp,bq,j,q^ | Needling therapies do not improve health-related quality of life (PCS) more than sham in the short term. |
| **Health-related quality of life**  (QofL) (mental component) 0 to 100; 0 = poor QofL follow-up: closest to 3 months | The mean health-related quality of life mental component ranged from **50.2** (SF-12, 0-100) **to 51** (SF-36, 0-100) | SMD **0.01 higher** (0.12 lower to 0.14 higher) | - | 952 (2 RCTs)^7,9^ | ⨁⨁◯◯ Low^bt,j,k,q^ | Needling therapies do not improve health-related quality of life (MCS) more than sham in the short term. |
| **Health-related quality of life**  (QofL) Korean version SF-36; 0 = poor QofL follow-up: closest to 6 months | The mean health-related quality of life was **125.87** | MD **3.39 higher** (2.98 lower to 9.76 higher) | - | 116 (1 RCT)^11,bo^ | ⨁◯◯◯ Very low^p,q,r,t^ | Needling therapies do not improve health-related quality of life more than sham in the intermediate term. |
| **Health-related quality of life**  (QofL) (physical component) 0 to 100; 0 = poor QofL follow-up: closest to 6 months | The mean health-related quality of life physical component ranged from **37.6** (SF-36, 0-100) **to 39.5** (SF-12, 0-100) | SMD **0.2 higher** (0.07 higher to 0.32 higher) | - | 955 (2 RCTs)^7,9^ | ⨁⨁◯◯ Low^bl,j,k,q^ | Needling therapies improve health-related quality of life (PCS) more than sham in the intermediate term. |
| **Health-related quality of life**  (QofL) (mental component) 0 to 100; 0 = poor QofL follow-up: closest to 6 months | The mean health-related quality of life mental component ranged from **46.8** (SF-36, 0-100) **to 50.9** (SF-12, 0-100) | SMD **0.1 higher** (0.18 lower to 0.39 higher) | - | 955 (2 RCTs)^7,9^ | ⨁◯◯◯ Very low^br,bv,j,q^ | Needling therapies do not improve health-related quality of life (MCS) more than sham in the intermediate term. |
| **Depression** 0 to 60; 0 = no depression follow-up: closest to 2 weeks | The mean depression was **50.7** | MD **2.5 lower** (5.23 lower to 0.23 higher) | - | 210 (1 RCT)^9^ | ⨁◯◯◯ Very low^i,p,q,t^ | Needling therapies do not reduce depression more than sham in the immediate term. |
| **Depression** follow-up: closest to 3 months | The mean depression ranged from **8.23** (BDI, 0-63**) to 49.4** (General Depression Scale, 0-60) | SMD **0.17 lower** (0.44 lower to 0.1 higher) | - | 326 (2 RCTs)^9,11^ | ⨁◯◯◯ Very low^ak,e,i,t^ | Needling therapies do not reduce depression more than sham in the short term. |
| **Depression** follow-up: closest to 6 months | The mean depression ranged from **7.52** (BDI, 0-63) **to 50.3** (General Depression Scale, 0-60) | SMD **0.1 lower** (0.33 lower to 0.12 higher) | - | 326 (2 RCTs)^9,11^ | ⨁◯◯◯ Very low^e,i,k,t^ | Needling therapies do not reduce depression more than sham in the intermediate term. |
| **Other psychological functioning** (fear avoidance, catastrophizing, anxiety, self-efficacy) | - | **-** | - | (0 studies) | - |  |
| **Social participation** | - | **-** | - | (0 studies) | - |  |
| **Adverse events/harms** | 88 per 1,000 | **135 per 1,000** (61 to 274) | **OR 1.62** (0.67 to 3.90) | 1014 (6 RCTs)^1,5,8,9,10,14,bw,bx^ | ⨁◯◯◯ Very low^by,bz,c,e^ | Needling therapies do not increase adverse events compared to sham during the intervention period. |
| **OLDER ADULTS (aged 60 years or more)** | | | | | | |
| **Pain** 0 to 100; 0 = no pain follow-up: closest to 2 weeks | Between-group MD (95% CI) of within-group MDs: -6.85 (-16.82 to 3.11) (46 participants total) | |  | (1 RCT)^8,l,m,n^ | ⨁◯◯◯ Very low^o,p,q,r^ | Needling therapies do not reduce pain more than sham in the immediate term. |
| **Pain**  0 to 100; 0 = no pain follow-up: closest to 3 months | Between-group MD (95% CI) of within-group MDs: -6.06 (-18.50 to 6.38) (46 participants total) | |  | (1 RCT)^8,l,m,n^ | ⨁◯◯◯ Very low^o,p,q,r^ | Needling therapies do not reduce pain more than sham in the short term. |
| **Pain** 0 to 100; 0 = no pain follow-up: closest to 6 months | Between-group MD (95% CI) of within-group MDs: -7.01 (-17.50 to 3.48) (46 participants total) | |  | (1 RCT)^8,l,m,n^ | ⨁◯◯◯ Very low^o,p,q,r^ | Needling therapies do not reduce pain more than sham in the intermediate term. |
| **Function** 0 to 100; 0 = no disability follow-up: closest to 2 weeks | Between-group MD (95% CI) of within-group MDs: -4.52 (-13.05 to 4.01) (46 participants total) | |  | (1 RCT)^8,l,m,n^ | ⨁◯◯◯ Very low^o,p,q,r^ | Needling therapies do not improve function more than sham in the immediate term. |
| **Function** 0 to 100; 0 = no disability follow-up: closest to 3 months | Between-group MD (95% CI) of within-group MDs: -3.04 (-12.34 to 6.25) (46 participants total) | |  | (1 RCT)^8,l,m,n^ | ⨁◯◯◯ Very low^o,p,q,r^ | Needling therapies do not improve function more than sham in the short term. |
| **Function** 0 to 100; 0 = no disability follow-up: closest to 6 months | Between-group MD (95% CI) of within-group MDs: 0.09 (-10.80 to 10.98) (46 participants total) | |  | (1 RCT)^8,l,m,n^ | ⨁◯◯◯ Very low^o,p,q,r^ | Needling therapies do not improve function more than sham in the immediate term. |
| **Health-related quality of life**  (QofL) 0 to 100; 0 = poor QofL follow-up: closest to 2 weeks | No improvement in needling therapies versus sham group (46 participants total) | |  | (1 RCT)^8,l,m,n^ | ⨁◯◯◯ Very low^o,p,q,r^ | Needling therapies do not improve health-related quality of life more than sham in the immediate term. |
| **Adverse events/harms** | No serious adverse events occurred during 4-week trial; 2 of 46 participants total (4.3%) had subcutaneous hematoma after needling (both from needling therapies group) (46 participants total) | |  | (1 RCT)^8,l,m^ | ⨁◯◯◯ Very low^o,p,q,r^ | Needling therapies may temporarily increase mild adverse events after needling. |
| **Psychological functioning** (depression, fear avoidance, anxiety, catastrophizing, self-efficacy) | - | **-** | - | (0 studies) | - |  |
| **Change in use of medications** | - | **-** | - | (0 studies) | - |  |
| **Falls** | - | **-** | - | (0 studies) | - |  |
| ***The risk in the intervention group** (and its 95% confidence interval) is based on the assumed risk in the comparison group and the **relative effect** of the intervention (and its 95% CI).  **CI:** confidence interval; **MD:** mean difference; **OR:** odds ratio; **SMD:** standardised mean difference | | | | | | |
| **GRADE Working Group grades of evidence** **High certainty:** we are very confident that the true effect lies close to that of the estimate of the effect. **Moderate certainty:** we are moderately confident in the effect estimate: the true effect is likely to be close to the estimate of the effect, but there is a possibility that it is substantially different. **Low certainty:** our confidence in the effect estimate is limited: the true effect may be substantially different from the estimate of the effect. **Very low certainty:** we have very little confidence in the effect estimate: the true effect is likely to be substantially different from the estimate of effect. | | | | | | |

#### Explanations

a. Yu 2020 assessed two comparisons (both included in meta-analysis).

b. Two studies were not included in the meta-analysis because they reported within-group change scores. Huang 2019: 46 participants total, rated as overall low risk of bias. Needling therapies made little or no difference to back pain: between-group MD of within-group MDs: -6.85, 95% CI -16.82 to 3.11 (VAS 0-100). Ushinohama 2016: 80 participants total; rated as overall high risk of bias. Small statistically significant difference between groups for median change in pain (p=0.032; effect size=0.21) favouring needling therapies.

c. Risk of bias: We downgraded twice because most of the weight (>50%) comes from high or unclear (i.e., some concerns) risk of bias studies.

d. Inconsistency: We did not down grade. The point estimates are similar with overlapping confidence intervals; statistical heterogeneity is between 0% and 40%, which might not be important (i.e., I2 = 9%).

e. Indirectness: We did not downgrade because the trials were conducted in different countries (high or upper-middle income).

f. Imprecision: We did not downgrade. The point estimate did not reach the pre-specified threshold for what may be considered clinically important (MD ≥ 1). The confidence interval does not cross the null or the boundary for what may be considered appreciable benefit (MD = -1).

g. One trial was not included in the meta-analysis because it only reported a within-group change score (Ushinohama 2016: 80 participants total; rated as overall high risk of bias). Small statistically significant difference between groups for median change in pain (p=0.032; effect size=0.21) favouring needling therapies.

h. Inconsistency: We downgraded twice. There is some similarity between confidence intervals and overlapping confidence intervals; statistical heterogeneity is between 50% and 90% (i.e., I2 = 69%). This could not be explained due to small subgroups and may represent substantial heterogeneity.

i. Imprecision: We downgraded once. The sample size is small (OIS would not have been achieved).

j. Risk of bias: We downgraded once because some of the weight (<50%) comes from high or unclear (i.e., some concerns) risk of bias studies.

k. Inconsistency: We did not downgrade. There is similarity between some or all point estimates and confidence intervals overlap; statistical heterogeneity is between 0% and 40%, which might not be important (i.e., I2 = 0%).

l. Treated with needling therapies type TCM.

m. Treated with needling therapies with manual stimulation.

n. Huang 2019 did not report follow-up scores (compared within-group changes between the 2 groups).

o. Risk of bias: We did not downgrade because all of the weight comes from low risk of bias studies.

p. Inconsistency: We did not downgrade; however, there are no other studies with which to compare findings.

q. Indirectness: We downgraded once; trial(s) conducted in one country (high or upper-middle income).

r. Imprecision: We downgraded twice. The sample size is small (OIS would not have been achieved).

s. Inconsistency: We did not downgrade. Some or all of the point estimates are similar with overlapping confidence intervals; statistical heterogeneity is between 0% and 40%, which might not be important (i.e., I2 = 18%).

t. Risk of bias: We downgraded twice because all of the weight comes from high or unclear (i.e., some concerns) risk of bias studies.

u. Inconsistency: We did not downgrade because statistical heterogeneity is between 0% and 40%, which might not be important (i.e., I2 = 32%).

v. One trial was not included in the meta-analysis because it reported a within-group change score (Huang 2019: 46 participants total; rated as overall low risk of bias). Needling therapies made little or no difference to back pain: between-group MD of within-group MDs: -6.85, 95% CI -16.82 to 3.11 (VAS 0-100).

w. Inconsistency: We did not downgrade. The point estimates are similar with overlapping confidence intervals; statistical heterogeneity is between 0% and 40%, which might not be important (i.e., I2 = 31%).

x. Risk of bias: We did not downgrade because most of the weight (>50%) comes from low risk of bias studies.

y. Inconsistency: We downgraded once. The point estimates are similar with overlapping confidence intervals; statistical heterogeneity is between 30% and 60% (i.e., I2 = 52%). This could not be explained due to small subgroups and may represent moderate heterogeneity.

z. Imprecision: We downgraded once. The point estimate did not reach the pre-specified threshold for what may be considered clinically important (MD ≥ 1). The confidence interval crosses the null. The lower boundary crosses the threshold for what may be considered appreciable benefit (-1).

aa. Cherkin 2009 assessed two comparisons (both included in meta-analysis).

ab. Kim 2020 assessed two comparisons (both included in meta-analysis).

ac. Two studies were not included in the meta-analysis because they included within-group change scores. Huang 2019: 46 participants total, rated as overall low risk of bias. Needling therapies made little or no difference to back pain: between-group MD of within-group MDs: -6.06 (-18.50 to 6.38) (VAS 0-100). Kong 2020: 121 participants total, rated as overall high risk of bias. No statistically significant difference between groups for mean change from baseline.

ad. Inconsistency: We downgraded twice. The point estimates vary and have some non-overlapping confidence intervals; statistical heterogeneity is between 50% and 90% (i.e., I2 = 68%). This could not be explained due to small subgroups and may represent substantial heterogeneity.

ae. Imprecision: We did not downgrade. The point estimate did not reach the threshold for what may be considered clinically important (MD ≥ 1). The confidence interval crosses the null but not the boundaries for appreciable benefit (MD = -1) or harm (MD = +1).

af. One trial was not included in the meta-analysis because it included a within-group change score. Kong 2020: 121 participants total, rated as high overall risk of bias. No statistically significant difference between groups for mean change from baseline.

ag. Inconsistency: We downgraded twice. The point estimates vary and have some non-overlapping confidence intervals; statistical heterogeneity is between 75% and 100% (i.e., I2 = 78%). This could not be explained due to small subgroups and may represent considerable heterogeneity.

ah. Imprecision: We downgraded once. The point estimate did not reach the pre-specified threshold for what may be considered clinically important (MD ≥ 1). The confidence interval does not cross the null; the lower boundary crosses the threshold for what may be considered appreciable benefit (MD = -1).

ai. One trial was not included in the meta-analysis because it reported a within-group change score (Huang 2019: 46 participants total; rated as overall low risk of bias). Needling therapies made little or no difference to back pain: between-group MD of within-group MDs: -6.06 (-18.50 to 6.38) (VAS 0-100).

aj. Inconsistency: We downgraded once. The point estimates vary and have some overlapping confidence intervals; statistical heterogeneity is between 30% and 60% (i.e., I2 = 45%). This could not be explained due to small subgroups and may represent moderate heterogeneity.

ak. Inconsistency: We did not downgrade. There is similarity between some point estimates and overlapping confidence intervals; statistical heterogeneity is between 0% and 40%, which might not be important (i.e., I2 = 28%).

al. Inconsistency: We downgraded twice. The point estimates vary and have some non-overlapping confidence intervals; statistical heterogeneity is between 75% and 100% (i.e., I2 = 83%). This could not be explained due to small subgroups and may represent considerable heterogeneity.

am. Risk of bias: We downgraded twice because most of the weight (>50%) comes from unclear (i.e., some concerns) risk of bias studies.

an. Inconsistency: We downgraded twice. The point estimates vary and have some non-overlapping confidence intervals. Statistical heterogeneity is between 75% and 100% (i.e., I2 = 82%); this could not be explained due to small subgroups and may represent considerable heterogeneity.

ao. One trial was not included in the meta-analysis because it reported a within-group change score (Huang 2019: 46 participants total; rated as overall low risk of bias). Needling therapies made little or no difference to back pain: between-group MD of within-group MDs: -7.01 (-17.50 to 3.48) (VAS 0-100).

ap. Inconsistency: We did not downgrade. The point estimates are similar with overlapping confidence intervals; statistical heterogeneity is between 0% and 40%, which might not be important (i.e., I2 = 27%).

aq. Inconsistency: We downgraded once. There is some similarity between point estimates and overlapping confidence intervals. Statistical heterogeneity is between 30% and 60% (i.e., I2 = 44%); this could not be explained due to small subgroups and may represent moderate heterogeneity.

ar. Inconsistency: We did not downgrade. There is similarity between point estimates and overlapping confidence intervals. Statistical heterogeneity is between 0% and 40%, which might not be important (i.e., I2 = 16%).

as. Two studies were not included in the meta-analysis because they included within-group change scores. Huang 2019: 46 participants total, rated as overall low risk of bias. No significant difference between groups for mean change from baseline. Kong 2020: 121 participants total, rated as overall high risk of bias. No statistically significant difference between groups for mean change from baseline.

at. Inconsistency: We downgraded once. There is some similarity between point estimates and overlapping confidence intervals. Statistical heterogeneity is between 50% and 90% (i.e., I2 = 66%). This could not be explained due to small subgroups and may represent substantial heterogeneity.

au. Imprecision: We downgraded once. The point estimate reached the pre-specified threshold for what may be considered clinically important (SMD ≥ 0.2). The confidence interval crosses the null.

av. Inconsistency: We downgraded once. The point estimates differ with overlapping confidence intervals. Statistical heterogeneity is between 30% and 60% (i.e., I2 = 42%); this could not be explained due to small subgroups and may represent moderate heterogeneity.

aw. Imprecision: We downgraded twice. The point estimate did not reach the pre-specified threshold for what may be considered clinically important (SMD ≥ 0.2). The lower boundary of the 95% CI crosses the threshold for what may be considered appreciable benefit (-0.2), and the upper boundary crosses the threshold for what may be considered appreciable harm (+0.2).

ax. One trial was not included in the meta-analysis because it reported a within-group change score (Huang 2019: 46 participants total; rated as overall low risk of bias). No significant difference between groups for mean change from baseline.

ay. Inconsistency: We downgraded twice. The point estimates vary with little overlap in confidence intervals. Statistical heterogeneity is between 75% and 100% (i.e., I2 = 84%); this could not be explained due to small subgroups and may represent considerable heterogeneity.

az. Inconsistency: We did not downgrade. The point estimates are similar with overlapping confidence intervals. Statistical heterogeneity is between 0% and 40%, which might not be important (i.e., I2 = 40%).

ba. Inconsistency: We downgraded twice. The point estimates vary with little overlap in confidence intervals. Statistical heterogeneity is between 75% and 100% (i.e., I2 = 77%); this could not be explained due to small subgroups and may represent considerable heterogeneity.

bb. Imprecision: We downgraded twice. The point estimate reached the pre-specified threshold for what may be considered clinically important (SMD ≥ 0.2). The upper boundary of the 95% CI crosses the threshold for what may be considered appreciable harm (+0.2).

bc. Inconsistency: We did not downgrade. There is some similarity in point estimates and overlapping confidence intervals. Statistical heterogeneity is between 0% and 40%, which might not be important (i.e., I2 = 38%).

bd. Imprecision: We did not downgrade. The point estimate did not reach the pre-specified threshold for what may be considered clinically important (SMD ≥ 0.2). The upper and lower boundaries of the 95% CI do not cross the threshold for what may be considered appreciable benefit (-0.2) or harm (+0.2).

be. Imprecision: We downgraded once. The point estimate did not reach the pre-specified threshold for what may be considered clinically important (SMD ≥ 0.2). The upper boundary of the 95% CI crosses the threshold for what may be considered appreciable harm (+0.2), but the lower boundary does not cross the threshold for what may be considered appreciable benefit (-0.2).

bf. Imprecision: We downgraded once. The point estimate did not reach the pre-specified threshold for what may be considered clinically important (SMD ≥ 0.2). The lower boundary of the 95% CI crosses the threshold for what may be considered appreciable benefit (-0.2), but the upper boundary does not cross the threshold for what may be considered appreciable harm (+0.2).

bg. Inconsistency: We downgraded once. There is some similarity between point estimates and overlapping confidence intervals. Statistical heterogeneity is between 30% and 60% (i.e., I2 = 51%). This could not be explained due to small subgroups and may represent moderate heterogeneity.

bh. Imprecision: We did not downgrade. The point estimate did not reach the pre-specified threshold for what may be considered clinically important (SMD ≥ 0.2). The upper and lower boundaries of the 95% CI do not cross the threshold for what may be considered appreciable benefit (-0.2) or harm (+0.2).

bi. Inconsistency: We did not downgrade. There is some similarity between point estimates and overlapping confidence intervals. Statistical heterogeneity is between 0% and 40%, which might not be important (i.e., I2 = 31%).

bj. Inconsistency: We downgraded once. There is some similarity between point estimates and overlapping confidence intervals. Statistical heterogeneity is between 30% and 60% (i.e., I2 = 46%); this could not be explained due to small subgroups and may represent moderate heterogeneity.

bk. Imprecision: We downgraded twice. The point estimate did not reach the pre-specified threshold for what may be considered clinically important (SMD ≥ 0.2). The lower boundary of the 95% CI crosses the threshold for what may be considered appreciable benefit (-0.2), and the upper boundary crosses the threshold for what may be considered appreciable harm (+0.2).

bl. Imprecision: We did not downgrade. The point estimate reached the threshold for what may be considered appreciable benefit (SMD ≥ 0.2). The confidence interval does not cross the null.

bm. Risk of bias: We downgraded once because some of the weight (<50%) comes from unclear (i.e., some concerns) risk of bias studies.

bn. One trial was not included in the meta-analysis because it reported a within-group change score (Huang 2019: 46 participants total; rated as overall low risk of bias). No significant difference between groups for mean change from baseline on any of the subscales.

bo. Cho 2013: Participants had an unknown presence of leg pain, and received needling therapies type TCM with manual stimulation. The trial did not stratify results based on gender, age, or race/ethnicity.

bp. Inconsistency: We downgraded twice. The point estimates varied with little overlap in the confidence intervals. Statistical heterogeneity is between 50% and 90% (i.e., I2 = 74%); this could not be explained due to small subgroups and may represent substantial heterogeneity.

bq. Imprecision: We downgraded once. The point estimate reached the pre-specified threshold for what may be considered clinically important (SMD ≥ 0.2). The confidence interval crosses the null.

br. Imprecision: We downgraded once. The point estimate did not reach the pre-specified threshold for what may be considered clinically important (SMD ≥ 0.2). The upper boundary of the 95% CI crosses the threshold for what may be considered appreciable benefit (+0.2), but the lower boundary does not cross the threshold for what may be considered appreciable harm (-0.2).

bs. One trial was not included in the meta-analysis due to missing data (Cherkin 2009: 638 participants total, rated as overall unclear risk of bias). Clinically unimportant (MD<10, scale 0-100) but statistically significant difference between groups for mean change in PCS and MCS (p<0.001) favouring needling therapies.

bt. Imprecision: We did not downgrade. The point estimate did not reach the pre-specified threshold for what may be considered clinically important (SMD ≥ 0.2). The upper and lower boundaries of the 95% CI do not cross the threshold for what may be considered appreciable benefit (+0.2) or harm (-0.2).

bu. Imprecision: We downgraded twice. The point estimate did not reach the pre-specified threshold for what may be considered clinically important (SMD ≥ 0.2). The lower boundary of the 95% CI crosses the threshold for what may be considered appreciable harm (-0.2), and the upper boundary crosses the threshold for what may be considered appreciable benefit (+0.2).

bv. Inconsistency: We downgraded twice. The point estimates differed with little overlap in confidence intervals. Statistical heterogeneity is between 50% and 90% (i.e., I2 = 70%). This could not be explained due to small subgroups and may represent substantial heterogeneity.

bw. Three studies were not included in the meta-analysis due to missing data. Cho 2013 (ID#: 2002): 130 participants total, rated as overall unclear risk of bias. Authors reported no serious events; 10 minor to moderate adverse events in needling therapies group (none persisted more than 1 week): pain; bruising at needling therapies site; pain, numbness or other bothersomeness in leg; shoulder pain. Haake 2007 (ID#: 2003): 774 participants total, rated as overall low risk of bias. Authors reported 476 clinically relevant adverse effects by 257 patients (22.6%) with no significant difference between groups. Molsberger 2002 (ID#: 2007): 186 participants total, rated as overall high risk of bias. Authors reported no important adverse events or side effects were observed in any group.

bx. Minor adverse events: Brinkhaus 2006: hematoma, bleeding in both groups. Cherkin 2009: mostly short-term pain with individualized or standardized needling therapies (1 participant reported pain lasting 1 month). Huang 2019: subcutaneous hematoma after needling therapies. Kong 2020: minor pain, bruising, skin rash, and slight bleeding at needle site; mild reaction to prone position included nausea, dizziness, and mild back ache in both groups. Koppenhaver 2021: pain during treatment, dizziness, unspecified emotional change. Yuan 2016: transient worsening back pain, needling therapies point bruise, back and leg numbness and discomfort, shoulder pain (up to 1 week) in both groups.

by. Inconsistency: We downgraded twice. The point estimates vary with little overlap in the confidence intervals. Statistical heterogeneity is between 50% and 90% (i.e., I2 = 63%). This could not be explained due to small subgroups and may represent substantial heterogeneity.

bz. Imprecision: We downgraded once. The point estimate reached the pre-specified threshold for what may be considered clinically important (OR ≥ 1.10). The lower boundary of the 95% CI crosses the threshold for what may be considered appreciable benefit (0.90).

ca. Minor adverse events: Huang 2019: subcutaneous hematoma after needling

cb. Risk of bias: We did not downgrade because all of the weight comes from low risk of bias studies.

cc. Minor adverse events: Cherkin 2009: mostly short-term pain with individualized or standardized needling therapies (1 participant reported pain lasting 1 month).

cd. Molsberger 2002 (ID#: 2007) was not included in meta-analysis due to missing data, 186 participants total, rated as overall high risk of bias. Authors reported no important adverse events or side effects were observed in any group.

ce. Minor adverse events: Brinkhaus 2006: hematoma, bleeding in both groups. Kong 2020: minor pain, bruising, skin rash, and slight bleeding at needle site; mild reaction to prone position included nausea, dizziness, and mild back ache in both groups. Koppenhaver 2021: pain during treatment, dizziness, unspecified emotional change. Yuan 2016: transient worsening back pain, needling therapies point bruise, back and leg numbness and discomfort, shoulder pain (up to 1 week) in both groups.

cf. Minor adverse events: Cherkin 2009: mostly short-term pain with individualized or standardized needling therapies (1 participant reported pain lasting 1 month). Huang 2019: subcutaneous hematoma after needling therapies. Yuan 2016: transient worsening back pain, needling therapies point bruise, back and leg numbness and discomfort, shoulder pain (up to 1 week) in both groups.

cg. Inconsistency: We downgraded once. There is some similarity between point estimates and overlapping confidence intervals. Statistical heterogeneity is between 30% and 60% (i.e., I2 = 57%). This could not be explained due to small subgroups and may represent moderate heterogeneity.

ch. Minor adverse events: Koppenhaver 2021: pain during treatment, dizziness, unspecified emotional change.

ci. Minor adverse events: Brinkhaus 2006: hematoma, bleeding in both groups. Cherkin 2009: mostly short-term pain with individualized or standardized needling therapies (1 participant reported pain lasting 1 month). Kong 2020: minor pain, bruising, skin rash, and slight bleeding at needle site; mild reaction to prone position included nausea, dizziness, and mild back ache in both groups.

cj. Inconsistency: We downgraded twice. The point estimates are in different directions with no overlap in confidence intervals. Statistical heterogeneity is between 50% and 90% (i.e., I2 = 89%). This could not be explained due to small subgroups and may represent substantial heterogeneity.

ck. Two studies were not included in the meta-analysis due to missing data. Cho 2013 (ID#: 2002): 130 participants total, rated as overall unclear risk of bias, authors reported no serious events; 10 minor to moderate adverse events in needling therapies group (none persisted more than 1 week) including pain, bruising at needling therapies site. Molsberger 2002 (ID#: 2007): 186 participant total, rated as overall high risk of bias, authors reported no important adverse events or side effects were observed in any group.

cl. Minor adverse events: Brinkhaus 2006: hematoma, bleeding in both groups. Koppenhaver 2021: pain during treatment, dizziness, unspecified emotional change. Yuan 2016: transient worsening back pain, needling therapies point bruise, back and leg numbness and discomfort, shoulder pain (up to 1 week) in both groups.

cm. Imprecision: We downgraded once. The point estimate reached the pre-specified threshold for what may be considered clinically important (OR ≥ 0.90). The upper boundary of the 95% CI crosses the threshold for what may be considered appreciable harm (1.10), but the lower boundary does not cross the threshold for what may be considered appreciable harm (0.90).

cn. Minor adverse events: Kong 2020: minor pain, bruising, skin rash, and slight bleeding at needle site; mild reaction to prone position included nausea, dizziness, and mild back ache in both groups.

co. One trial was not included in the meta-analysis due to missing data. Haake 2007 (ID#: 2003): 774 participants total, rated as overall low risk of bias; authors reported 476 clinically relevant adverse effects by 257 patients (22.6%) with no significant difference between groups.

#### References

1.Yuan Q, Liu L,Ma J,Wu W,Ye M,Zhang Y. [A clinical study of acupuncture therapy for treatment of chronic nonspecific low back pain] . 2016.

2.Yu S, Ortiz A,Gollub RL,et al. Acupuncture treatment modulates the connectivity of key regions of the descending pain modulation and reward systems in patients with chronic low back pain. 2020.

3.Molsberger AF, Mau J,Pawelec DB,Winkler J. Does acupuncture improve the orthopedic management of chronic low back pain--a randomized, blinded, controlled trial with 3 months follow up. 2002.

4.Martín-Corrales C, Bautista IV,Méndez-Mera JE,et al. Benefits of Adding Gluteal Dry Needling to a Four-Week Physical Exercise Program in a Chronic Low Back Pain Population. A Randomized Clinical Trial. 2020.

5.Koppenhaver SL, Weaver AM,Randall TL,et al. Effect of dry needling on lumbar muscle stiffness in patients with low back pain: a double blind, randomized controlled trial using shear wave elastography. . 2021.

6.Kerr DP, Walsh DM,Baxter D. Acupuncture in the management of chronic low back pain: a blinded randomized controlled trial. 2003.

7.Haake M, Muller HH,Schade-Brittinger C,et al.. German Acupuncture Trials (GERAC) for chronic low back pain: randomized, multicenter, blinded, parallel-group trial with 3 groups. 2007.

8.Huang Z, Liu S,Zhou J,Yao Q,Liu Z. Efficacy and Safety of Acupuncture for Chronic Discogenic Sciatica, a Randomized Controlled Sham Acupuncture Trial. 2019.

9.Brinkhaus B, Witt CM,Jena S,et al. Acupuncture in patients with chronic low back pain: a randomized controlled trial. 2006.

10.Cherkin DC, Sherman KJ,Avins AL,et al. A randomized trial comparing acupuncture, simulated acupuncture, and usual care for chronic low back pain. 2009.

11.Cho YJ, Song YK,Cha YY,et al. Acupuncture for chronic low back pain: a multicenter, randomized, patient-assessor blind, sham-controlled clinical trial. 2013.

12.de Castro Moura C, de Cássia Lopes Chaves E,Couto Machado Chianca T,Ruginsk SG,Alves Nogueira D,Iunes DH. Effects of auricular acupuncture on chronic pain in people with back musculoskeletal disorders: a randomized clinical trial. 2019.

13.Kim H, Mawla I,Lee J,et al. Reduced tactile acuity in chronic low back pain is linked with structural neuroplasticity in primary somatosensory cortex and is modulated by acupuncture therapy. 2020.

14.Kong JT, Puetz C,Tian L,et al. Effect of Electroacupuncture vs Sham Treatment on Change in Pain Severity Among Adults With Chronic Low Back Pain: a Randomized Clinical Trial. 2020.

Summary of findings table 2: ***What are the benefits and harms of needling therapies in the management of community-dwelling adults (including older adults aged 60 years and over) with chronic primary low back pain (with or without leg pain) compared to no intervention or interventions where the effect of needling therapies could be isolated?***

| Outcomes | **Anticipated absolute effects^*^** (95% CI) | | Relative effect (95% CI) | № of participants (studies) | Certainty of the evidence (GRADE) | Comments |
| --- | --- | --- | --- | --- | --- | --- |
|  | **Risk with no treatment** | **Risk with needling therapies** |  |  |  |  |
| **ALL ADULTS** | | | | | | |
| **Pain** 0 to 10; 0 = no pain follow-up: closest to 2 weeks | The mean pain ranged from **1.49 to 6.43** | MD **1.21 lower** (1.5 lower to 0.92 lower) | - | 1717 (21 RCTs)^1,2,3,4,5,6,7,8,9,10,11,12,13,14,15,16,17,18,19,20,21,a,b^ | ⨁⨁◯◯ Low^c,d,e,f^ | Needling therapies reduce pain more than no treatment in the immediate term. |
| **Pain** 0 to 10; 0 = no pain follow-up: closest to 3 months | The mean pain ranged from **2.4 to 6.42** | MD **1.56 lower** (2.18 lower to 0.95 lower) | - | 762 (9 RCTs)^1,4,13,14,16,20,21,22,23,a,s^ | ⨁⨁◯◯ Low^c,d,e,f^ | Needling therapies reduce pain more than no treatment in the short term. |
| **Function** follow-up: closest to 2 weeks | The mean function ranged from **4.6** (RMDQ, 0-24) **to 49.7** (ODI, 0-100) | SMD **1.39 lower** (2 lower to 0.77 lower) | - | 1541 (19 RCTs)^1,2,3,4,5,6,7,8,9,10,11,12,13,14,16,17,18,19,20,a,v^ | ⨁⨁◯◯ Low^c,e,f,w^ | Needling therapies improve function more than no treatment in the immediate term. |
| **Function** follow-up: closest to 3 months | The mean function ranged from **2.51** (BPI, 0-10) **to 66.8** (Hannover, 0-100) | SMD **0.57 lower** (0.92 lower to 0.22 lower) | - | 639 (8 RCTs)^1,4,13,14,16,20,22,23,ae,af^ | ⨁⨁◯◯ Low^c,e,f,w^ | Needling therapies improve function more than no treatment in the short term. |
| **Function** 0 to 100; 0 = no disability follow-up: closest to 6 months | The mean function was **66** | MD **8.3 lower** (13.93 lower to 2.67 lower) | - | 214 (1 RCT)^23,ah^ | ⨁◯◯◯ Very low^c,g,l,p^ | Needling therapies improve function more than no treatment in the intermediate term (may not meet clinically important threshold: MD ≥10). |
| **Health-related quality of life** 0 to 1; 0 = poor QofL follow-up: closest to 2 weeks | The mean health-related quality of life was **0.734** | MD **0.02 higher** (0.09 lower to 0.14 higher) | - | 38 (1 RCT)^10^ | ⨁◯◯◯ Very low^c,g,i,p^ | Needling therapies do not improve health-related quality of life compared to no treatment in the immediate term. |
| **Health -related quality of life** (QofL) (physical component) 0 to 100; 0 = poor QofL follow-up: closest to 3 months | The mean health-related quality of life was **33.9** | MD **6.6 higher** (3.9 higher to 9.3 higher) | - | 214 (1 RCT)^23,ah,aj^ | ⨁◯◯◯ Very low^c,g,l,p^ | Needling therapies improve health-related quality of life (PCS) more than no treatment in the short term (may not meet clinically important threshold: MD ≥10). |
| **Health-related quality of life**  (QofL) (mental component) 0 to 100; 0 = poor QofL follow-up: closest to 3 months | The mean health-related quality of life was **49.4** | MD **1.2 higher** (1.86 lower to 4.26 higher) | - | 214 (1 RCT)^23,ah,ak^ | ⨁◯◯◯ Very low^c,g,l,p^ | Needling therapies do not improve health-related quality of life (MCS) compared to no treatment in the short term. |
| **Depression** 0 to 61; 0 = no depression follow-up: closest to 3 months | The mean depression was **49.7** | MD **0.8 lower** (3.6 lower to 2 higher) | - | 214 (1 RCT)^23,ah^ | ⨁◯◯◯ Very low^c,g,l,p^ | Needling therapies do not improve depression compared to no treatment in the short term. |
| **Other psychological functioning** (fear avoidance, catastrophizing, anxiety, self-efficacy) | - | **-** | - | (0 studies) | - |  |
| **Social participation** | - | **-** | - | (0 studies) | - |  |
| **Adverse events/harms** | 18 per 1,000 | **55 per 1,000** (8 to 303) | **OR 3.12** (0.42 to 23.44) | 223 (3 RCTs)^20,24,25,al,am^ | ⨁◯◯◯ Very low^an,ao,c,j^ | Needling therapies do not increase adverse events compared to no treatment during the intervention period. |
| **OLDER ADULTS (aged 60 years or more)** | | | | | | |
| **Pain** 0 to 10; 0 = no pain follow-up: closest to 2 weeks | The mean pain was **2.4** | MD **0.9 lower** (1.53 lower to 0.27 lower) | - | 47 (1 RCT)^4,aw,ax^ | ⨁◯◯◯ Very low^c,g,i,p^ | Needling therapies reduce pain more than no treatment in the immediate term (may not meet clinically important threshold: MD ≥1). |
| **Pain** 0 to 10; 0 = no pain follow-up: closest to 3 months | The mean pain was **2.4** | MD **1.1 lower** (1.62 lower to 0.58 lower) | - | 47 (1 RCT)^4,aw,ax^ | ⨁◯◯◯ Very low^c,g,i,p^ | Needling therapies improve function more than no treatment in the immediate term. |
| **Function** follow-up: closest to 2 weeks | The mean function was **11** | SMD **1.1 lower** (1.71 lower to 0.48 lower) | - | 47 (1 RCT)^4,ax^ | ⨁◯◯◯ Very low^c,g,i,p^ | Needling therapies improve function more than no treatment in the immediate term. |
| **Function** follow-up: closest to 3 months | The mean function was **11.2** | SMD **1.04 lower** (1.66 lower to 0.43 lower) | - | 47 (1 RCT)^4,ax^ | ⨁◯◯◯ Very low^c,g,i,p^ | Needling therapies improve function more than no treatment in the short term. |
| **Health-related quality of life** | - | **-** | - | (0 studies) | - |  |
| **Adverse events/harms** | - | **-** | - | (0 studies) | - |  |
| **Psychological functioning** (depression, fear avoidance, catastrophizing, anxiety, self-efficacy) | - | **-** | - | (0 studies) | - |  |
| **Change in use of medications** | - | **-** | - | (0 studies) | - |  |
| **Falls** | - | **-** | - | (0 studies) | - |  |
| ***The risk in the intervention group** (and its 95% confidence interval) is based on the assumed risk in the comparison group and the **relative effect** of the intervention (and its 95% CI).  **CI:** confidence interval; **MD:** mean difference; **OR:** odds ratio; **SMD:** standardised mean difference | | | | | | |
| **GRADE Working Group grades of evidence** **High certainty:** we are very confident that the true effect lies close to that of the estimate of the effect. **Moderate certainty:** we are moderately confident in the effect estimate: the true effect is likely to be close to the estimate of the effect, but there is a possibility that it is substantially different. **Low certainty:** our confidence in the effect estimate is limited: the true effect may be substantially different from the estimate of the effect. **Very low certainty:** we have very little confidence in the effect estimate: the true effect is likely to be substantially different from the estimate of effect. | | | | | | |

#### Explanations

a. Zaringhalam 2010 assessed two comparisons (there were 2 comparison groups). Both comparisons included in meta-analysis.

b. Two studies were not included in the meta-analysis because they reported within-group change scores. De Castro Moura 2019 (ID#: 32): 111 participants total, rated as overall high risk of bias. Clinically important (MD≥1, scale 0 to 10) and statistically significant within group mean difference for Chinese auricular acupuncture group: 1.38 (95% CI 0.43; 2.33); no significant within group changes for French auricular acupuncture or comparison group; no statistical comparison between groups. Weiß 2013 (ID#: 1153): 160 participants total, rated as overall high risk of bias. No significant difference between groups in the proportion of participants experiencing improvement in pain while sitting/standing or walking.

c. Risk of bias: We downgraded twice because all of the weight comes from high or unclear (i.e., some concerns) overall risk of bias studies.

d. Inconsistency: We did not downgrade. All or most trials are in the same direction, showing a reduction in pain.

e. Indirectness: We did not downgrade because the trials were conducted in different countries (high to low-income).

f. Imprecision: We did not downgrade. The point estimate reached the pre-specified threshold for what may be considered clinically important (MD ≥ 1 or SMD ≥ 0.2 ). The confidence interval does not cross the null.

g. Inconsistency: We did not downgrade; however, there are no other studies with which to compare findings.

h. Indirectness: We downgraded once; trial(s) conducted in one country (low or lower-middle income).

i. Imprecision: We downgraded twice. The sample size is small (OIS would not have been achieved).

j. Indirectness: We did not downgrade because the trials were conducted in different countries (high or upper-middle income).

k. Imprecision: We did not downgrade. The point estimate did not reach the pre-specified threshold for what may be considered clinically important (MD ≥ 1). The confidence interval does not cross the null.

l. Imprecision: We downgraded once. The sample size is small (OIS would not have been achieved).

m. Inconsistency: We downgraded once. Most trials are in the same direction with similar point estimates. Statistical heterogeneity is between 75% and 100% (i.e., I2 = 97%). This could not be explained due to small subgroups and may represent considerable heterogeneity.

n. Inconsistency: We downgraded once. Most of the trials are in the same direction showing a reduction in pain. Statistical heterogeneity is between 75% and 100% (i.e., I2 = 92%). This could not be explained due to small subgroups and may represent considerable heterogeneity.

o. Indirectness: We did not downgrade because the trials were conducted in different countries (low or lower-middle income).

p. Indirectness: We downgraded once; trial(s) conducted in one country (high or upper-middle income).

q. One trial was not included in the meta-analysis because it reported within-group change scores. De Castro Moura 2019 (ID#: 32): 111 participants total; rated as overall high risk of bias. Clinically important (MD≥1, scale 0 to 10) and statistically significant within group mean difference for Chinese auricular acupuncture group: 1.38 (95% CI 0.43; 2.33); no significant within group changes for French auricular acupuncture or comparison group; no statistical comparison between groups.

r. One trial was not included in the meta-analysis because it reported within-group change scores. Weiß 2013 (ID#: 1153): 160 participants total, rated as overall high risk of bias. No significant difference between groups in the proportion of participants experiencing improvement in pain while sitting/standing or walking.

s. Two studies were not included in the meta-analysis because they reported within-group change scores. De Castro Moura 2019 (ID#: 32): 111 participants total, rated as overall high risk of bias. No significant within group changes needling therapies groups or comparison group; no statistical comparison between groups. Weiß 2013 (ID#: 1153): 160 participants total, rated as overall high risk of bias. Statistically significant difference between proportion of participants experiencing improvement in pain while sitting/standing (p<0.01) but not in pain while walking.

t. Imprecision: We downgraded once. The point estimate reached the pre-specified threshold for what may be considered clinically important (MD ≥ 1). The upper boundary of the 95% CI crosses the threshold for what may be considered appreciable benefit (-1).

u. Use of stimulation was not reported in Weiß 2013 (ID#: 1153).

v. One trial was not included in the meta-analysis because it reported within-group change scores. De Castro Moura 2019 (ID#: 32): 111 participants total; rated as overall high risk of bias. Clinically unimportant (MD<2.4, scale 0 to 24) but statistically significant within group mean difference for Chinese auricular acupuncture group: 1.56 (95% CI 0.10; 3.02); no significant within group changes for French auricular acupuncture or comparison group; no statistical comparison between groups.

w. Inconsistency: We did not downgrade. All or most trials are in the same direction, showing a reduction in functional limitation.

x. Inconsistency: We downgraded once. The studies are in the same direction. One point estimate is much larger in magnitude; confidence intervals of the other studies do not overlap with it. Statistical heterogeneity is between 75% and 100% (i.e., I2 = 99%). This could not be explained due to small subgroups and may represent considerable heterogeneity.

y. Imprecision: We downgraded twice. The point estimate reached the pre-specified threshold for what may be considered clinically important (SMD ≥ 0.2). The lower boundary of the 95% CI crosses the threshold for what may be considered appreciable benefit (-0.2), and the upper boundary crosses the threshold for what may be considered appreciable harm (+0.2).

z. Inconsistency: We downgraded once. The point estimates differ with little overlap in confidence intervals. Statistical heterogeneity is between 75% and 100% (i.e., I2 = 98%). This could not be explained due to small subgroups and may represent considerable heterogeneity.

aa. Inconsistency: We downgraded once. Most of the point estimates are in the same direction. Statistical heterogeneity is between 75% and 100% (i.e., I2 = 94%). This could not be explained due to small subgroups and may represent considerable heterogeneity.

ab. Inconsistency: We downgraded once. Most of the trials are in the same direction showing a reduction in functional limitation. Statistical heterogeneity is between 75% and 100% (i.e., I2 = 92%). This could not be explained due to small subgroups and may represent considerable heterogeneity.

ac. Inconsistency: We downgraded once. The point estimates are in the same direction. Statistical heterogeneity is between 75% and 100% (i.e., I2 = 94%). This could not be explained due to small subgroups and may represent considerable heterogeneity.

ad. Inconsistency: We downgraded once. The point estimates are in the same direction with little overlap between confidence intervals. Statistical heterogeneity is between 75% and 100% (i.e., I2 = 76%). This could not be explained due to small subgroups and may represent considerable heterogeneity.

ae. One trial was not included in the meta-analysis because it reported within-group change scores. De Castro Moura 2019 (ID#: 32): 111 participants total; rated as overall high risk of bias. No significant within group changes for needling therapies groups or comparison group; no statistical comparison between groups.

af. One trial was not included in the meta-analysis because it reported within-group change scores. Witt 2006 (ID#: 2010): 3093 participants total; rated as overall high risk of bias. Statistically significant difference between groups for mean percent disability reduction (scale 0 to 100) (22.0; 95% CI 19.3, 24.7; p<0.001) favouring needling therapies.

ag. Use of stimulation was not reported in Witt 2006 (ID#: 2010).

ah. Brinkhaus 2006: participants had no leg pain; in high to upper-middle income country; were treated with mixed needling therapies type (TCM, dry needling) with manual stimulation.

ai. Sung 2020: needling therapies with threading stimulation; rated as overall unclear risk of bias.

aj. One trial was not included in the meta-analysis because it reported within-group change scores. Witt 2006 (ID#: 2010): 3093 participants total; rated as overall high risk of bias. clinically unimportant (PCS: MD <10, scale 0-100) but statistically significant difference between groups for mean point increase in quality of life (4.7; 95% CI 4.0, 5.4; p<0.001) favouring needling therapies.

ak. One trial was not included in the meta-analysis because it reported within-group change scores. Witt 2006 (ID#: 2010): 3093 participants total; rated as overall high risk of bias. Clinically unimportant (MCS: MD<10, scale 0-100) but statistically significant different between groups for mean point increase in quality of life (2.1; 95% CI 1.4, 2.8; p<0.001) favouring needling therapies.

al. One trial was not included in meta-analysis due to missing data. Molsberger 2002 (ID#: 2007): 186 participants total, rated as overall high risk of bias. Authors reported no important adverse events or side effects were observed in any group.

am. Minor adverse events: Kerr 2003: increased tenderness, leg pain for a few days following treatment. Ushinohama 2016: dizziness in one participant (unknown treatment group allocation). Yuan 2016: transient (up to 1 week) worsening back pain, needling therapies point pain and bruising, back and leg numbness and discomfort, shoulder pain, foot pain.

an. Inconsistency: We downgraded once. The point estimates vary and have overlapping confidence intervals. Statistical heterogeneity is between 30% and 60% (i.e., I2 = 41%). This could not be explained due to small subgroups and may represent moderate heterogeneity.

ao. Imprecision: We downgraded twice due to small sample size and number of events.

ap. Minor adverse events: Ushinohama 2016: dizziness in one participant (unknown treatment group allocation). Yuan 2016: transient (up to 1 week) worsening back pain, needling therapies point pain and bruising, back and leg numbness and discomfort, shoulder pain, foot pain.

aq. Inconsistency: We did not downgrade. The point estimates are in the same direction with overlapping confidence intervals. Statistical heterogeneity is between 0% and 40%, which might not be important (i.e., I2 = 0%).

ar. Minor adverse events: Kerr 2003: increased tenderness, leg pain for a few days following treatment.

as. Minor adverse events: Kerr 2003: increased tenderness, leg pain for a few days following treatment. Yuan 2016: transient (up to 1 week) worsening back pain, needling therapies point pain and bruising, back and leg numbness and discomfort, shoulder pain, foot pain.

at. Inconsistency: We downgraded once. The point estimates go in different directions; there is some overlap in confidence intervals. Statistical heterogeneity is between 50% and 90% (i.e., I2 = 71%). This could not be explained due to small subgroups and may represent substantial heterogeneity.

au. Minor adverse events: Ushinohama 2016: dizziness in one participant (unknown treatment group allocation).

av. Minor adverse events: Yuan 2016: transient (up to 1 week) worsening back pain, needling therapies point pain and bruising, back and leg numbness and discomfort, shoulder pain, foot pain.

aw. Meng 2003: Pain Scale range not specified (assumed 0-10)

ax. Meng 2003: Participants had no leg pain, were in a high to upper-middle income country, and were treated with needling therapies type TCM with electrical stimulation.

#### References

1.Depaoli Lemos VJ, Selau RC,Blos C,Baptista Dohnert M,Boff Daitx R,de Almeida Brito V. Electroacupuncture and Transcutaneous Electrical Nerve Stimulation in Chronic Nonspecific Low Back Pain: a Blind Randomized Clinical Trial. 2021.

2.Li YJ, Zhuang WS,Cai XG,Yang Y,Han MM,Zhang DW. [Effect of acupuncture at " three points of ilioumbar" on lumbar function and pain in patients with iliopsoas muscle strain]. 2019.

3.Li SK, Zhao J,Cao XW,Zhu S,Liu ZQ,Fan YX. [Core stability training combined with acupuncture in treatment of chronic nonspecific low back pain: a prospective randomized controlled trial]. 2022.

4.Meng CF, Wang D,Ngeow J,Lao L,Peterson M,Paget S. Acupuncture for chronic low back pain in older patients: a randomized, controlled trial. 2003.

5.Moslemi F, Farokhi ZS. Effects of electroacupuncture on pain, functional disability and ultrasonographic changes of gluteus maximus muscle in non-specific chronic low back pain patients with gluteus maximus muscle trigger points. 2020.

6.Pan J, Yang L,Qiu Y,et al. [Clinical trial on effect of acupuncture combined with sling exercise training on lumbar disc herniation] . 2019.

7.P, Ran. [Effect of acupuncture on pain and lumbar function in patients with discogenic low back pain] . 2021.

8.Ren B, Feng X,Zhang C. [Clinical trial on the analgesic effect of acupuncture based on meridian-tendon dialectical theory in chronic low back pain]. 2021.

9.Shi F, Dong B,Lin X,Fu Y. [Clinical observation on treatment of non-specific low back pain based on meridian theory and acupuncture]. 2021.

10.Sung WS, Hong Y,Jeon SR,et al. Efficacy and safety of thread embedding acupuncture combined with acupuncture for chronic low back pain: A randomized, controlled, assessor-blinded, multicenter clinical trial. 2020.

11.Tabatabaiee A, Takamjani IE,Sarrafzadeh J,Salehi R,Ahmadi M. Ultrasound-guided dry needling decreases pain in patients with piriformis syndrome. 2019.

12.Wang X, Yu X,Huang F,Luo X,Gong Y. [Effect of acupuncture combined with fire acupuncture on pain and serum inflammatory factor in patients with lumbar disc herniation]. 2020a.

13.Wang L, Wang Z,Wang Y,Guo Y. Observations on the efficacy of acupuncture for lumbar intervertebral disc herniation. 2020b.

14.Yeung CK, Leung MC,Chow DH. The use of electro-acupuncture in conjunction with exercise for the treatment of chronic low-back pain. 2003.

15.Yu XJ, Zhang L,Lu WY,Gao Q,Liu L,Wang Y. Effect of electroacupuncture combined with caudal epidural injection on functional rehabilitation of patients with lumbar hernia. 2021.

16.Zaringhalam J, Manaheji H,Rastqar A,Zaringhalam M. Reduction of chronic non-specific low back pain: a randomised controlled clinical trial on acupuncture and baclofen. 2010.

17.Zhai B, Wang C. [Therapeutic effect of drug cupping combined with deep puncture on Jiaji point on lumbar disc herniation and its effect on M-JOA score, ODI index and quality of life]. 2019.

18.Zhu X, Shen X,Kang L,et al.. Clinical trial on acupuncture combined with bone setting manipulation in the treatment of lumbar disc herniation. 2020.

19.Z, Zhu. Clinical trial on warming-needle moxibustion combined with joint loosening for chronic low back pain. 2021.

20.Yuan Q, Liu L,Ma J,Wu W,Ye M,Zhang Y. [A clinical trial of acupuncture therapy for treatment of chronic nonspecific low back pain] . 2016.

21.Molsberger AF, Mau J,Pawelec DB,Winkler J. Does acupuncture improve the orthopedic management of chronic low back pain--a randomized, blinded, controlled trial with 3 months follow up. 2002.

22.de Castro Moura C, de Cássia Lopes Chaves E,Couto Machado Chianca T,Ruginsk SG,Alves Nogueira D,Iunes DH. Effects of auricular acupuncture on chronic pain in people with back musculoskeletal disorders: a randomized clinical trial. 2019.

23.Brinkhaus B, Witt CM,Jena S,et al. Acupuncture in patients with chronic low back pain: a randomized controlled trial. 2006.

24.Ushinohama A, Cunha BP,Costa LO,Barela AM,Freitas PB. Effect of a single session of ear acupuncture on pain intensity and postural control in individuals with chronic low back pain: a randomized controlled trial. 2016.

25.Kerr DP, Walsh DM,Baxter D. Acupuncture in the management of chronic low back pain: a blinded randomized controlled trial. 2003.

Summary of findings table 3: ***What are the benefits and harms of needling therapies in the management of community-dwelling adults (including older adults aged 60 years and over) with chronic primary low back pain (with or without leg pain) compared to usual care?***

| Outcomes | **Anticipated absolute effects^*^** (95% CI) | | Relative effect (95% CI) | № of participants (studies) | Certainty of the evidence (GRADE) | Comments |
| --- | --- | --- | --- | --- | --- | --- |
|  | **Risk with usual care** | **Risk with needling therapies** |  |  |  |  |
| **ALL ADULTS** | | | | | | |
| **Pain** 0 to 10; 0 = no pain follow-up: closest to 3 months | The mean pain was **4.70** | MD **1.35 lower** (1.86 lower to 0.84 lower) | - | 447 (1 RCT)^1,a^ | ⨁◯◯◯ Very low^b,c,d,e^ | Needling therapies reduce pain more than usual care in the short term. |
| **Pain** 0 to 10; 0 = no pain follow-up: closest to 6 months | The mean pain was **4.40** | MD **0.65 lower** (1.17 lower to 0.13 lower) | - | 430 (1 RCT)^1,a^ | ⨁◯◯◯ Very low^b,c,d,f^ | Needling therapies reduce pain more than usual care in the intermediate term (may not meet clinically important threshold: MD ≥1). |
| **Pain** 0 to 10; 0 = no pain follow-up: closest to 12 months | The mean pain was **4.10** | MD **0.5 lower** (1.02 lower to 0.02 higher) | - | 431 (1 RCT)^1,a^ | ⨁◯◯◯ Very low^b,c,d,g^ | Needling therapies do not reduce pain more than usual care in the long term. |
| **Function**  0 to 24; 0 = no disability  follow-up: closest to 3 months | The mean function was **8.90** | MD **2.55 lower** (3.7 lower to 1.4 lower) | - | 447 (1 RCT)^1,a^ | ⨁◯◯◯ Very low^b,c,d,h^ | Needling therapies improve pain more than usual care in the short term. |
| **Function**  0 to 24; 0 = no disability  follow-up: closest to 6 months | The mean function was **8.40** | MD **1.65 lower** (2.83 lower to 0.47 lower) | - | 430 (1 RCT)^1,a^ | ⨁◯◯◯ Very low^b,c,d,i^ | Needling therapies improve function more than usual care in the intermediate term (may not meet clinically important threshold: MD ≥2.4). |
| **Function**  0 to 24; 0 = no disability  follow-up: closest to 12 months | The mean function was **7.90** | MD **1.9 lower** (3.15 lower to 0.65 lower) | - | 431 (1 RCT)^1,a^ | ⨁◯◯◯ Very low^b,c,d,i^ | Needling therapies improve function more than usual care in the long term (may not meet clinically important threshold: MD ≥2.4). |
| **Health-related quality of life** | - | **-** | - | (0 studies) | - |  |
| **Adverse events/harms** | - | **-** | - | (0 studies) | - |  |
| **Psychological functioning** (depression, fear avoidance, catastrophizing, anxiety, self-efficacy) | - | **-** | - | (0 studies) | - |  |
| **Social participation** | - | **-** | - | (0 studies) | - |  |
| **OLDER ADULTS (aged 60 years or more)** | | | | | | |
| **Pain** | - | **-** | - | (0 studies) | - |  |
| **Function** | - | **-** | - | (0 studies) | - |  |
| **Health-related quality of life** | - | **-** | - | (0 studies) | - |  |
| **Adverse events/harms** | - | **-** | - | (0 studies) | - |  |
| **Psychological functioning** (depression, fear avoidance, catastrophizing, anxiety, self-efficacy) | - | **-** | - | (0 studies) | - |  |
| **Change in use of medications** | - | **-** | - | (0 studies) | - |  |
| **Falls** | - | **-** | - | (0 studies) | - |  |
| ***The risk in the intervention group** (and its 95% confidence interval) is based on the assumed risk in the comparison group and the **relative effect** of the intervention (and its 95% CI).  **CI:** confidence interval; **MD:** mean difference | | | | | | |
| **GRADE Working Group grades of evidence** **High certainty:** we are very confident that the true effect lies close to that of the estimate of the effect. **Moderate certainty:** we are moderately confident in the effect estimate: the true effect is likely to be close to the estimate of the effect, but there is a possibility that it is substantially different. **Low certainty:** our confidence in the effect estimate is limited: the true effect may be substantially different from the estimate of the effect. **Very low certainty:** we have very little confidence in the effect estimate: the true effect is likely to be substantially different from the estimate of effect. | | | | | | |

#### Explanations

a. Cherkin 2009 had 2 comparisons (both included in meta-analysis); needling therapies stimulation not reported; rated as overall unclear risk of bias.

b. Risk of bias: We downgraded twice because all of the weight comes from high or unclear (i.e., some concerns) risk of bias studies.

c. Inconsistency: We did not downgrade; however, there are no other studies with which to compare findings.

d. Indirectness: We downgraded once because the trial was conducted in one country (high-income).

e. Imprecision: We downgraded once. The point estimate reached the pre-specified threshold for what may be considered clinically important (MD ≥ 1). The upper boundary of the 95% CI crosses the threshold for what may be considered appreciable benefit (-1).

f. Imprecision: We downgraded once. The point estimate did not reach the pre-specified threshold for what may be considered clinically important (MD ≥ 1). The lower boundary of the 95% CI crosses the threshold for what may be considered appreciable benefit (-1).

g. Imprecision: We downgraded once. The point estimate did not reach the pre-specified threshold for what may be considered clinically important (MD ≥ 1). The lower boundary of the 95% CI crosses the threshold for what may be considered appreciable benefit (-1), but the upper boundary does not cross the threshold for what may be considered appreciable harm (+1).

h. Imprecision: We downgraded once. The point estimate reached the pre-specified threshold for what may be considered clinically important (MD ≥ 2.4). The upper boundary of the 95% CI crosses the threshold for what may be considered appreciable benefit (-2.4).

i. Imprecision: We downgraded once. The point estimate did not reach the pre-specified threshold for what may be considered clinically important (MD ≥ 2.4). The lower boundary of the 95% CI crosses the threshold for what may be considered appreciable benefit (-2.4).

#### References

1.Cherkin DC, Sherman KJ,Avins AL,et al. A randomized trial comparing acupuncture, simulated acupuncture, and usual care for chronic low back pain. 2009.

**Online Resource 7.** Meta-analyses (including subgroup analyses and sensitivity analyses)

**References**

1. Brinkhaus B, Witt CM, Jena S, Linde K, Streng A, Wagenpfeil S, et al. Acupuncture in patients with chronic low back pain: a randomized controlled trial. Arch Intern Med. 2006;166(4):450-7.

2. Cherkin DC, Sherman KJ, Avins AL, Erro JH, Ichikawa L, Barlow WE, et al. A randomized trial comparing acupuncture, simulated acupuncture, and usual care for chronic low back pain. Arch Intern Med. 2009;169(9):858-66.

3. Cho YJ, Song YK, Cha YY, Shin BC, Shin IH, Park HJ, et al. Acupuncture for chronic low back pain: a multicenter, randomized, patient-assessor blind, sham-controlled clinical trial. Spine (Phila Pa 1976). 2013;38(7):549-57.

4. de Castro Moura C, de Cássia Lopes Chaves E, Couto Machado Chianca T, Ruginsk SG, Alves Nogueira D, Iunes DH. Effects of auricular acupuncture on chronic pain in people with back musculoskeletal disorders: a randomized clinical trial. Revista da escola de enfermagem da USP. 2019;53:1‐9.

5. de Castro Moura C, Chaves ECL, Chianca TCM, Ruginsk SG, Nogueira DA, Souza VHS, et al. Contribution of Chinese and French ear acupuncture for the management of chronic back pain: a randomised controlled trial. J Clin Nurs. 2019;28(21‐22):3796‐806.

6. Depaoli Lemos VJ, Selau RC, Blos C, Baptista Dohnert M, Boff Daitx R, de Almeida Brito V. Electroacupuncture and Transcutaneous Electrical Nerve Stimulation in Chronic Nonspecific Low Back Pain: a Blind Randomized Clinical Trial. Muscles, ligaments & tendons journal (MLTJ). 2021;11(4):719‐27.
[truncated: 22,887 more chars]
